# Clinical validation of a next-generation sequencing-based multi-cancer early detection “liquid biopsy” blood test in over 1,000 dogs using an independent testing set: The CANcer Detection in Dogs (CANDiD) study

**DOI:** 10.1371/journal.pone.0266623

**Published:** 2022-04-26

**Authors:** Andi Flory, Kristina M. Kruglyak, John A. Tynan, Lisa M. McLennan, Jill M. Rafalko, Patrick Christian Fiaux, Gilberto E. Hernandez, Francesco Marass, Prachi Nakashe, Carlos A. Ruiz-Perez, Donna M. Fath, Thuy Jennings, Rita Motalli-Pepio, Kate Wotrang, Angela L. McCleary-Wheeler, Susan Lana, Brenda Phillips, Brian K. Flesner, Nicole F. Leibman, Tracy LaDue, Chelsea D. Tripp, Brenda L. Coomber, J. Paul Woods, Mairin Miller, Sean W. Aiken, Amber Wolf-Ringwall, Antonella Borgatti, Kathleen Kraska, Christopher B. Thomson, Alane Kosanovich Cahalane, Rebecca L. Murray, William C. Kisseberth, Maria A. Camps-Palau, Franck Floch, Claire Beaudu-Lange, Aurélia Klajer-Peres, Olivier Keravel, Luc-André Fribourg-Blanc, Pascale Chicha Mazetier, Angelo Marco, Molly B. McLeod, Erin Portillo, Terry S. Clark, Scott Judd, C. Kirk Feinberg, Marie Benitez, Candace Runyan, Lindsey Hackett, Scott Lafey, Danielle Richardson, Sarah Vineyard, Mary Tefend Campbell, Nilesh Dharajiya, Taylor J. Jensen, Dirk van den Boom, Luis A. Diaz, Daniel S. Grosu, Arthur Polk, Kalle Marsal, Susan Cho Hicks, Katherine M. Lytle, Lauren Holtvoigt, Jason Chibuk, Ilya Chorny, Dana W. Y. Tsui

**Affiliations:** 1 PetDx, La Jolla, California, United States of America; 2 Veterinary Specialty Hospital of San Diego, San Diego, California, United States of America; 3 Veterinary Specialty Hospital of North County, San Marcos, California, United States of America; 4 Department of Veterinary Medicine and Surgery, University of Missouri, Columbia, Missouri, United States of America; 5 Department of Clinical Sciences, Colorado State University, Fort Collins, Colorado, United States of America; 6 Department of Clinical Science and Advanced Medicine, University of Pennsylvania, Philadelphia, Pennsylvania, United States of America; 7 The Animal Medical Center, New York, New York, United States of America; 8 Southeast Veterinary Oncology and Internal Medicine, Orange Park, Florida, United States of America; 9 Bridge Animal Referral Center, Edmonds, Washington, United States of America; 10 Department of Biomedical Sciences, Ontario Veterinary College, University of Guelph, Guelph, Ontario, Canada; 11 Institute for Comparative Cancer Investigation at the Mona Campbell Centre for Animal Cancer, Ontario Veterinary College, University of Guelph, Guelph, Ontario, Canada; 12 Department of Veterinary Clinical Sciences, University of Minnesota, College of Veterinary Medicine, Saint Paul, Minnesota, United States of America; 13 Veterinary Specialty Hospital of Hong Kong, Wan Chai, Hong Kong; 14 Department of Veterinary Clinical Sciences, The Ohio State University College of Veterinary Medicine, Columbus, Ohio, United States of America; 15 Oncovet, Villeneuve-D’ascq, France; 16 AniCura TRIOVet, Rennes, France; 17 Clinique Vétérinaire de la Pierre Bleue, Pipriac, France; 18 Eiffelvet, Paris, France; 19 Clinique Vétérinaire SeineVet, Rouen, France; 20 Clinique Vétérinaire Mazetier, Argenteuil, France; 21 Governor Animal Clinic, Inc., San Diego, California, United States of America; 22 City Paws Home Health, Columbus, Ohio, United States of America; 23 VCA Valley Oak Veterinary Center, Chico, California, United States of America; 24 VCA Metroplex Animal Hospital, Irving, Texas, United States of America; 25 Prices Creek Veterinary Services, Lewisburg, Ohio, United States of America; 26 Carlsbad Animal Hospital, Carlsbad, California, United States of America; 27 Oceanside Veterinary Hospital, Oceanside, California, United States of America; 28 Amici Pet Hospital of Little Italy, San Diego, California, United States of America; 29 Colony Veterinary Hospital, San Diego, California, United States of America; 30 Carriage Hills Animal Hospital, Montgomery, Alabama, United States of America; 31 Healthbit.ai Inc., San Diego, California, United States of America; 32 Laboratory Corporation of America, Durham, North Carolina, United States of America; 33 Advisor to PetDx, La Jolla, California, United States of America; 34 Division of Solid Tumor Oncology, Memorial Sloan Kettering Cancer Center, New York, New York, United States of America; Bauer Research Foundation, UNITED STATES

## Abstract

Cancer is the leading cause of death in dogs, yet there are no established screening paradigms for early detection. Liquid biopsy methods that interrogate cancer-derived genomic alterations in cell-free DNA in blood are being adopted for multi-cancer early detection in human medicine and are now available for veterinary use. The CANcer Detection in Dogs (CANDiD) study is an international, multi-center clinical study designed to validate the performance of a novel multi-cancer early detection “liquid biopsy” test developed for noninvasive detection and characterization of cancer in dogs using next-generation sequencing (NGS) of blood-derived DNA; study results are reported here. In total, 1,358 cancer-diagnosed and presumably cancer-free dogs were enrolled in the study, representing the range of breeds, weights, ages, and cancer types seen in routine clinical practice; 1,100 subjects met inclusion criteria for analysis and were used in the validation of the test. Overall, the liquid biopsy test demonstrated a 54.7% (95% CI: 49.3–60.0%) sensitivity and a 98.5% (95% CI: 97.0–99.3%) specificity. For three of the most aggressive canine cancers (lymphoma, hemangiosarcoma, osteosarcoma), the detection rate was 85.4% (95% CI: 78.4–90.9%); and for eight of the most common canine cancers (lymphoma, hemangiosarcoma, osteosarcoma, soft tissue sarcoma, mast cell tumor, mammary gland carcinoma, anal sac adenocarcinoma, malignant melanoma), the detection rate was 61.9% (95% CI: 55.3–68.1%). The test detected cancer signal in patients representing 30 distinct cancer types and provided a Cancer Signal Origin prediction for a subset of patients with hematological malignancies. Furthermore, the test accurately detected cancer signal in four presumably cancer-free subjects before the onset of clinical signs, further supporting the utility of liquid biopsy as an early detection test. Taken together, these findings demonstrate that NGS-based liquid biopsy can offer a novel option for noninvasive multi-cancer detection in dogs.

## Introduction

Cancer is by far the leading cause of death in adult dogs [1]. In many cases, canine cancer is identified only after clinical signs have developed, by which point the disease is often advanced, the ability to provide long-term control is low, and the prognosis is poor. Just as for human cancer patients, early detection and treatment are considered essential for achieving the best possible clinical outcomes for canine cancer patients [2, 3], and studies of cancers detected at earlier stages (including incidentally detected cases) in dogs have demonstrated improved outcomes [4–8]. In humans, organ-based cancer screening programs such as mammograms for women, prostate specific antigen (PSA) testing for men, and colonoscopies are well established and are covered by most insurance policies, as they have been proven to help detect cancers at earlier stages, when treatment is more effective, and a cure is more likely to be achieved [9]. Liquid biopsy methods, which detect blood-based analytes such as cancer-derived DNA, were initially developed as noninvasive alternatives to tissue-based “companion diagnostic” testing [10] for selection of targeted treatments in human cancer patients [11]; liquid biopsy solutions are particularly useful in situations where tissue biopsies were difficult or impossible to obtain, such as in lung cancer [12, 13]. The first FDA approval of a liquid biopsy next-generation sequencing (NGS) based test was issued in August 2020, for a companion diagnostic indication [14].

Liquid biopsy methods are now being adopted in human medicine to simultaneously screen for multiple types of cancer with a simple blood test; these multi-cancer early detection (MCED) tests represent a paradigm shift in cancer screening and promise to significantly increase the number of cases that are detected at earlier stages in human patients [15, 16]. The first MCED test to receive an FDA breakthrough device designation (May 2019) [17], became commercially available for use in humans in June 2021 [18]. Cell-free DNA (cfDNA) based methods have been previously explored in the veterinary space for prognosis in canine lymphoid neoplasia, using cfDNA concentration measurements [19]; for detection of small genomic alterations in canine histiocytic sarcoma, oral malignant melanoma, and multicentric lymphoma, using PCR (polymerase chain reaction) techniques [20]; and for detection of multiple classes of genomic alterations (and demonstration of intra-patient spatial heterogeneity) in tissue and plasma samples from dogs diagnosed with multiple cancer types, using next-generation sequencing [21].

Widespread cancer screening programs do not currently exist for dogs. Routine preventive care, “wellness” physical exams, and commonly available tests (e.g., CBC, chemistry, urinalysis, and basic imaging) are generally unable to detect canine cancers at a preclinical stage. MCED liquid biopsy methods may offer clinical utility as a new screening paradigm in canine patients [21, 22], allowing veterinarians to detect many different cancer types noninvasively before the emergence of clinical signs by using the latest technologies now available for multi-cancer screening in humans.

Beyond its use as a screening tool, liquid biopsy may also provide utility in canine patients as an aid-in-diagnosis for cancer, particularly when traditional diagnostic testing would be challenging. In some cases, cancer may be suspected, but pursuing a diagnosis through established methods (e.g., tissue sampling via surgical biopsy) may be difficult or deemed too risky due to the anatomical location of the mass or other characteristics of the procedure. In other cases, cancer may be high on the list of differential diagnoses based on the clinical presentation, but traditional diagnostic techniques cannot be employed because a specific anatomical location for the suspected cancer is not clinically evident.

Here, results of the CANcer Detection in Dogs (CANDiD) study are reported. CANDiD is an international, multi-center clinical study designed to validate the performance of a novel MCED “liquid biopsy” test using next-generation sequencing of blood-derived DNA, developed for the noninvasive detection and characterization of cancer in dogs.

## Methods

The CANDiD study was based on a prospective sample collection program that enrolled 1,358 client-owned dogs, with and without cancer, at 41 clinical sites across the US, Canada, Brazil, the Netherlands, France, and Hong Kong between November 2019 and August 2021. Collection sites included veterinary specialty practices, university/academic veterinary hospitals, and general practices. All subjects were enrolled under protocols that received Institutional Animal Care and Use Committee (IACUC) or site-specific ethics approval, according to each site’s requirements. All subjects were client-owned, and written informed consent was obtained from all owners.

### Criteria for inclusion, exclusion, and characterization of subjects

Blood samples were prospectively collected from an all-comers cohort of dogs with confirmed or suspected malignancy at the time of enrollment; only subjects in which cancer was definitively diagnosed were ultimately included in the validation. Additionally, blood samples were collected from an all-comers cohort of dogs who were presumed to be cancer-free due to no history of cancer and no suspicion of cancer, based on a thorough clinical history and physical exam by the treating veterinarian at the time of study enrollment; these “presumably cancer-free" subjects were allowed to enroll if they had known or suspected medical conditions other than cancer.

Dogs were eligible for study enrollment if: their owner provided written informed consent for collection and use of their dog’s blood and clinical data; they weighed a minimum of 12 pounds (5.5 kg); they were 1 year of age or older; and they were able, in the professional opinion of the managing veterinarian, to provide the amount of whole blood required for the study (a minimum of 14 mL collected across two specialized tubes, with a minimum of 7 mL collected in each tube). Dogs of all breeds were eligible to enter the study.

Dogs were excluded from enrollment in the study if they: had experienced physical trauma (including injury, surgery, or core needle biopsy for any clinical indication) in the 7 days prior to blood collection (routine blood collection, fine needle aspiration, and cystocentesis were not excluded); were believed to be pregnant; or if collection of a blood sample provided an unacceptable risk to the site staff and/or the dog, in the professional opinion of the managing veterinarian. No subjects were excluded based on known or suspected comorbidities, including acute and chronic inflammatory, infectious, autoimmune, degenerative, or other conditions. Dogs were excluded from enrollment in the cancer-diagnosed cohort if they previously underwent curative-intent surgery for cancer and were considered to be cancer-free at the time of enrollment; or had undergone targeted or non-targeted chemotherapy, immunotherapy, radiation therapy, or experimental treatment for cancer (other than steroidal or non-steroidal anti-inflammatory drugs) within 30 days prior to enrollment.

The rationale for exclusion due to recent trauma was based on knowledge from liquid biopsy testing in humans that tissue disruption (for example as a result of biopsy or surgery) can lead to the release of large amounts of cell-free DNA and/or circulating tumor DNA (ctDNA—representing the fraction of cfDNA originating from tumor cells) into the bloodstream. In cancer-diagnosed subjects, disproportionate disruption of surrounding normal tissue (with release of high amounts of normal cfDNA) could lead to a transient “dilution” of the ctDNA fraction, potentially making cancer detection by liquid biopsy methods more difficult [23]. Alternatively, disproportionate disruption of the tumor tissue could trigger a transient increase in the ctDNA fraction, potentially leading to an artificially inflated rate of detection by liquid biopsy [24, 25]. In short, tissue trauma could confound the measurement of test performance in unpredictable ways. Trauma was also an exclusion criterion for presumably cancer-free subjects, in order to avoid enrollment of subjects where a clinical observation of “trauma” may represent the first clinical sign of occult cancer. Given that the half-life of cfDNA in both humans and dogs is estimated to be no longer than a few hours [26, 27], 7 days was considered to be a reasonable exclusion period for all subjects with recent trauma. The rationale for exclusion due to pregnancy was likewise due to prior knowledge from human medicine that fetal or placental chromosomal abnormalities and *de novo* mutations are detectable in cfDNA [28–30]. Although the prevalence and distribution of such events during gestation (and their rates of detection in the blood of pregnant females by cfDNA methods) are not well documented in dogs, it was deemed prudent to exclude pregnant subjects given the potential for pregnancy to confound test results.

All cancer-diagnosed subjects had complete staging, performed by the managing veterinarian according to standard-of-care staging guidelines at the enrolling site for that cancer type. Metastasis to a local or distant anatomical site was determined by tissue pathology or, in cases where tissue sampling was not possible due to anatomical location, by imaging diagnosis. “Possible” but unconfirmed nodules or tissue changes on imaging for which the radiologist provided both malignant and benign differentials were not included as part of determining the extent of disease; confirmed nodules or tissue changes determined by the radiologist to most likely represent malignant disease were included as part of determining the extent of disease.

Subjects with cancers amenable to tissue-based diagnosis, or to imaging-based diagnosis (such as those with tumors in anatomical locations that precluded tissue diagnosis, for example heart base masses), were included in the analysis. Subjects that had more than one confirmed primary cancer type were included, and the subject’s diagnosis was recorded as the union of diagnoses. Subjects enrolled into the cancer-diagnosed cohort whose tumors were ultimately determined to be benign by pathology were excluded from analysis.

Cancer size as well as cancer stage are correlated with detection by NGS-based liquid biopsy methods in humans. This study was designed to allow for evaluation of such correlations in dogs. The longest diameter of the largest lesion was measured and recorded for the vast majority of cancer-diagnosed subjects. Furthermore, simplified definitions were developed to allow for classification of extent of disease in cancer-diagnosed subjects, given that the process of cancer staging is less standardized in dogs than it is in humans, and many canine cancer types have distinct staging methodologies [31, 32]. *Localized/regional* was defined as cancer that was limited to the organ of origin or to nearby lymph nodes, tissues, or organs; or lymphomas limited to a single lymph node (Stage I) or multiple lymph nodes on one side of the diaphragm (Stage II). *Disseminated/metastatic* was defined as cancer that had spread to areas of the body distant from the primary tumor; or lymphomas that involved two or more lymph nodes on both sides of the diaphragm and/or one or more extra-nodal sites (Stages III, IV, and V); or any non-lymphoma hematological malignancy. *Undetermined* was used in a small number of cases where it was not possible to accurately determine the extent of disease, despite a complete cancer staging workup. These definitions allowed for all cancer-diagnosed cases (whether solid or hematological) to be classified by extent of disease.

Clinical data for each visit were collected on standardized case report forms (CRFs). Full source-document collection (including but not limited to clinical intake and progress notes, imaging reports, surgery reports, and histopathology reports) and verification were performed for each subject. Queries were issued to clinical sites to clarify or correct data items, as appropriate, before clinical database lock. Final clinical determinations, such as benign versus malignant tumor and cancer type, stage, and size, were performed by board-certified veterinary medical oncologists on the central study team and recorded prior to unblinding of liquid biopsy testing results. This independent clinical adjudication process was informed by all of the written clinical information available, including the diagnosis provided by each subject’s managing veterinarian(s); however, primary data derived from imaging tests (e.g., actual images) and pathology tests (e.g., actual slides or pictures of slides) were not independently reviewed by the central study team.

### Sample collection and laboratory procedures

Whole blood was collected from a peripheral vein (jugular, cephalic, or saphenous) using the Cell-Free DNA Collection Tube (Roche); these specialized blood collection tubes were designed to prevent white blood cell (WBC) lysis and cfDNA degradation for up to seven days in transit at ambient temperature. This allowed for blood samples to be shipped to the central testing laboratory (PetDx, La Jolla, CA) without any processing or special sample handling (such as refrigeration, freezing, or centrifugation) at the collection site. Samples were collected without any restrictions related to the time of day or the time of the dog’s last feeding. Two tubes were collected from each subject, with a minimum of 7 mL of whole blood per tube. In one subject, presented below as a case study, tumor tissue was collected from multiple anatomical locations at necropsy using a 2 mm punch biopsy tool, and each biopsy specimen was stored in a vial containing DNA Shield (Zymo Research) stabilization solution and shipped to the central testing laboratory at ambient temperature.

Subjects who were unable to provide at least two tubes of blood, or whose tubes did not meet minimum fill requirements, were excluded from analysis. Samples that were received or processed more than 7 days from the time of collection were also excluded from analysis.

Upon receipt at the central lab, blood samples were processed with a double-centrifugation protocol to separate plasma from WBCs [21], and were scored on the extent of hemolysis and lipemia. Plasma aliquots, WBC pellets, and tissue samples were stored at -80°C until they were thawed for testing. Samples were tested between February 2021 and September 2021 and were randomized for laboratory processing across batches, operators, and reagent lots, for the avoidance of bias.

Cell-free DNA was extracted from plasma using a proprietary bead-based chemistry optimized to maximize cfDNA yield in canine subjects. Genomic DNA (gDNA) was extracted from WBCs, and from tissue samples, using QIAamp DNA Mini Blood Kit (Qiagen). Amplified DNA libraries were generated for each subject from the matched cfDNA and gDNA extracts. Libraries were prepared by incorporating universal adapters and barcodes into sample DNA via ligation and universal PCR amplification, and were subjected to next-generation sequencing on an Illumina NovaSeq 6000 for somatic variant analysis. All sequencing reads were aligned to the CanFam3.1 reference genome [33] using the BWA-MEM algorithm [34] with default parameters. Somatic variant calling from cfDNA and gDNA sequence data was performed using a custom bioinformatics pipeline leveraging Sentieon TNscope [35], ichorCNA [36], and internally-developed algorithms that are based on the coverage at specific genomic regions or on fragmentomics profiles. CNV profiles were evaluated using 1 Mb bins. Small variant (single nucleotide variants and insertion/deletion variants) annotation was performed using Ensembl Variant Effect Predictor (VEP) Release 103 [37]. Liquid biopsy testing results for each subject underwent dual blinded review, with independent adjudication for discrepant cases prior to final reporting. All analyses were performed on the Google Cloud Platform leveraging the Cloud Life Sciences API.

### Analytical repeatability and reproducibility

Prior to testing prospectively collected samples for clinical validation of the test, analytical repeatability and reproducibility of the test were assessed using contrived samples. Two established canine cell lines, MDCK.1 (ATCC CRL-2935) and Cf2Th (ATCC CRL-1430), were sourced from American Type Culture Collection (ATCC) and were sequenced to confirm baseline variant profiles. DNA was extracted from these cell lines and from the white blood cells (WBC) of a single cancer-free canine subject, respectively. Extracted DNA was fragmented, and cell line-derived DNA was then serially diluted into WBC-derived DNA at defined genomic equivalent ratios. Repeatability at each dilution level was assessed by analyzing agreement among multiple within-run replicates, processed by the same operators under the same conditions. Reproducibility at each dilution level was assessed by analyzing agreement among replicates across multiple runs, operators, days, and reagent lots. The repeatability and reproducibility of the test were each >95% at all mixing ratios tested.

### Training and testing sets for clinical validation

For clinical validation, cancer-diagnosed and presumably cancer-free subjects were randomly assigned to training and testing sets in a 1:4 ratio: that is, approximately 20% of subjects were assigned to training and 80% to testing. Population-level sex, weight, and age matching between the cancer-diagnosed and presumably cancer-free cohorts was enforced in the training set to limit the potential for bias due to demographic variables. This resulted in presumably cancer-free subjects in the training set having a higher median age compared to presumably cancer-free subjects in the testing set, since cancer subjects tended to be older than cancer-free subjects overall. Bioinformatics algorithms were optimized and locked based on analysis of the training set, and the final pipeline was subsequently applied to the separate testing set for independent validation of the test’s clinical performance [38, 39]. Blood samples from all cancer-diagnosed and presumably cancer-free subjects, across the training and testing sets, were processed using the same laboratory workflows.

### Test performance: Sensitivity and specificity

Test sensitivity was defined as the percentage of all cancer-diagnosed dogs in the testing set who received a *Cancer Signal Detected* (positive) result. Test specificity was defined as the percentage of all presumably cancer-free dogs in the testing set who received a *Cancer Signal Not Detected* (negative) result. Binomial confidence intervals were calculated for all performance estimates.

Overall test performance was evaluated using the testing set and included subjects with a single primary cancer as well as subjects with multiple concurrent primary cancers. A subgroup analysis was performed on cancer-diagnosed subjects from the testing set representing a predefined list of three of the most aggressive canine cancers: lymphoma, hemangiosarcoma, and osteosarcoma. Another subgroup analysis focused on cancer-diagnosed subjects from the testing set representing a predefined list of eight common canine cancers [40] that account for most cancer deaths in the species: lymphoma, hemangiosarcoma, osteosarcoma, soft tissue sarcoma, mast cell tumor, mammary gland carcinoma, anal sac adenocarcinoma, and malignant melanoma. Separately, detection rates by cancer type were determined for a large number of distinct cancer types using data from all cancer-diagnosed subjects across the training and testing sets. The detection rate for the subgroup of subjects diagnosed with multiple concurrent primary cancers was also determined in this manner.

Additional analyses were performed to determine the effect of pre-analytical factors such as the time difference between sample collection and processing (time in-transit), and the levels of hemolysis and lipemia, on the test’s performance in the cancer-diagnosed as well as the presumably cancer-free subjects in the testing set. Hemolysis and lipemia were assessed by visual inspection of each sample based on color and extent of turbidity, respectively, using scales of 0–4 and 0–2, respectively.

### Statistical analyses

All metrics were summarized as median and range unless otherwise stated. P-values were calculated using Student’s t-test for continuous variables and the Chi-squared test for categorical variables. A p-value of <0.05 was considered statistically significant. All confidence intervals reported are two-sided 95% binomial intervals. Analyses were performed using R package version 4.0.5.

## Results

### Subject disposition

**Fig 1:**
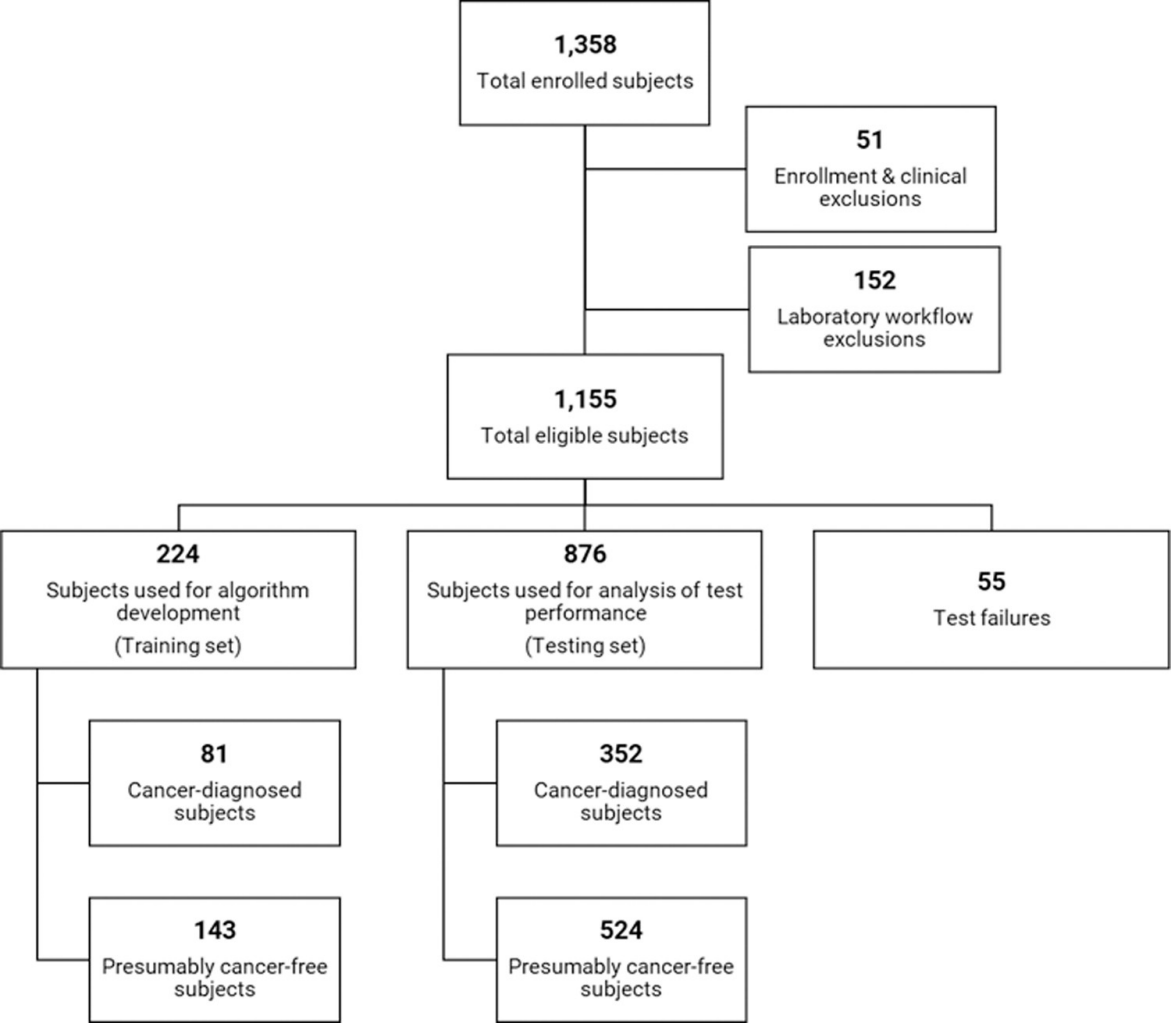
Disposition of subjects in the CANDiD study.

A total of 1,358 dogs were enrolled in the CANDiD study. Of these, 51 subjects were excluded due to enrollment protocol deviations or clinical criteria, such as: age <1 year at time of enrollment; subject initially presumed to have cancer but a definitive diagnosis could not be confirmed, or the mass was determined to be benign by pathology; or subject initially presumed to be cancer-free but received a diagnosis of cancer between the time of blood collection and time of analysis. Additionally, 152 subjects were excluded due to deviations from the standardized laboratory workflow, such as: time from collection to processing >7 days; low blood collection tube fill volume; or only one blood collection tube received. From the remaining 1,155 subjects eligible for clinical validation testing, 55 failed testing for reasons such as: low plasma volume, sample swap detected, DNA library failure, and sequencing-based QC failure. From the remaining 1,100 subjects (the “validation set”), 224 were used for algorithm development (the “training set”), and 876 were used for analysis of test performance (the “testing set”) (Fig 1). Full subject level data are included in S1 Table.

### Subject characteristics

The subject characteristics for the training and testing sets are shown in Table 1. There were no significant differences between the training set and the testing set in the proportions of: male to female subjects; purebred to mixed-breed subjects; cancer-diagnosed to presumably-cancer free subjects; subjects with localized/regional disease; and subjects with a tumor diameter measuring ≤5 cm. Likewise, there was no significant difference between the weights of the subjects in the training set versus the testing set. There was a statistically significant overall difference between the ages of subjects in the two sets, with the training set representing a slightly older cohort (median 7.3 vs 6.6 years, p<0.0001), as expected due to the enforced age matching in the training set, as described above.

**Table 1:** Comparison of subject demographics and cancer characteristics for the training and testing sets.

		Training set (n = 224)	Testing set (n = 876)	p-value
**Age** ^1^	Median (years)	7.3	6.6	<0.0001
	Range (years)	1.0–15.6	1.0–15.8	
**Weight** ^1^	Median (kg)	28.4	29.1	0.2111
	Range (kg)	5.9–81.7	6.0–106.8	
**Sex** ^2^	Male	107 (48%)	463 (53%)	0.1989
	*Intact*	9	74	
	*Neutered*	98	388	
	*Not Provided*	0	1	
	Female	117 (52%)	413 (47%)	
	*Intact*	12	42	
	*Spayed*	105	371	
**Breed** ^2^	Purebred	113 (50%) (40 distinct breeds)	434 (50%) (78 distinct breeds)	0.8679
	Mixed-breed	111 (50%)	442 (50%)	
**Cancer status** ^2^	Cancer-diagnosed	81 (36%)	352 (40%)	0.3064
	Presumably cancer-free	143 (64%)	524 (60%)	
**Extent of disease** ^2^	Localized/regional^3^ (% of cancer-diagnosed subjects)	50/81 (62%)	204/352 (58%)	0.1521
**Tumor size** ^2^	Tumor diameter ≤5 cm^4^ (% of cancer-diagnosed subjects)	47/81 (58%)	185/352 (53%)	0.6727

^1^Significance assessed using t-test.

^2^Significance assessed using Chi-squared test.

^3^Localized/regional was defined as cancer that was limited to the organ of origin or to nearby lymph nodes, tissues, or organs; or lymphomas limited to a single lymph node (Stage I) or multiple lymph nodes on one side of the diaphragm (Stage II).

^4^This measurement was based on the longest diameter of the largest lesion in each subject.

The 224 subjects in the training set included 113 purebred dogs (as provided by the dog owners) representing 40 distinct breeds, and 111 mixed-breed dogs; 107 males and 117 females; median age 7.3 years (range: 1.0–15.6 years); and median weight 28.4 kg (range: 5.9–81.7 kg). The training set included 81 cancer-diagnosed subjects and 143 presumably cancer-free subjects (Table 1).

The 876 subjects in the testing set included 434 purebred dogs (as provided by the dog owners) representing 78 distinct breeds, and 442 mixed-breed dogs; 463 males and 413 females; median age 6.6 years (range: 1.0–15.8 years); and median weight 29.1 kg (range: 6.0–106.8 kg). The testing set included 352 cancer-diagnosed subjects and 524 presumably cancer-free subjects (Table 1).

In the testing set, there were no significant differences between the presumably cancer-free subjects and the cancer-diagnosed subjects in the proportion of male to female subjects and purebred to mixed-breed subjects. Likewise, there was no significant difference between the weights of presumably cancer-free and cancer-diagnosed subjects. There was a statistically significant difference between the ages of subjects in the cancer-diagnosed cohort and the presumably cancer-free cohort, with the cancer-diagnosed subjects representing an older population (median 9.7 vs. 4.2 years, p<0.0001), as expected due to the preponderance of cancer diagnoses in older dogs (Table 2).

**Table 2:** Demographics of presumably cancer-free vs. cancer-diagnosed subjects in the testing set.

		Presumably cancer-free (n = 524)	Cancer-diagnosed (n = 352)	p-value
**Age** ^1^	Median (years)	4.2	9.7	<0.0001
	Range (years)	1.0–15.0	1.9–15.8	
**Weight** ^1^	Median (kg)	28.5	29.7	0.3844
	Range (kg)	6.0–106.8	6.9–67.3	
**Sex** ^2^	Male	273 (52%)	190 (54%)	0.6612
	*Intact*	52	22	
	*Neutered*	221	167	
	*Not Provided*	0	1	
	Female	251 (48%)	162 (46%)	
	*Intact*	31	11	
	*Spayed*	220	151	
**Breed** ^2^	Purebred	260 (50%)	174 (49%)	>0.9999
	Mixed-breed	264 (50%)	178 (51%)	

^1^Significance assessed using t-test.

^2^Significance assessed using Chi-squared test.

The 352 cancer-diagnosed subjects in the testing set comprised 205 (58%) *localized/regional* cases, 136 (39%) *disseminated/metastatic* cases, and 11 (3%) cases where the extent of disease was *undetermined*.

Full lists of the breeds and cancer types represented in the CANDiD study (across the training and testing sets) are provided in Tables 3 and 4, respectively.

**Table 3:** List of 85 dog breeds represented in the training and testing sets of the CANDiD study.

**A**	**D**	**P**
Airedale Terrier	Dachshund	Pembroke Welsh Corgi
Akita	Dalmatian	Pointer
Alaskan Malamute	Doberman Pinscher	Pomeranian
American Staffordshire Terrier	Dogo Argentino	Poodle, Miniature
Anatolian Shepherd	Dogue de Bordeaux	Poodle, Standard
Australian Cattle Dog	**E**	Pug
Australian Shepherd	English Bulldog	Pyrenean Shepherd
Australian Terrier	English Cocker Spaniel	**R**
**B**	English Setter	Rat Terrier
Barbet	English Springer Spaniel	Rhodesian Ridgeback
Basenji	**F**	Rottweiler
Basset Hound	Flat-Coated Retriever	**S**
Beagle	French Bulldog	Samoyed
Belgian Malinois	**G**	Scottish Terrier
Bernese Mountain Dog	German Shepherd	Shetland Sheepdog
Bloodhound	German Shorthaired Pointer	Shih Tzu
Border Collie	German Wirehaired Pointer	Siberian Husky
Boston Terrier	Golden Retriever	Silken Windhound
Bouvier des Flandres	Gordon Setter	Small Munsterlander
Boxer	Great Dane	Soft Coated Wheaten Terrier
Boykin Spaniel	Great Pyrenees	Saint Bernard
Brittany	Greater Swiss Mountain Dog	Stabyhoun
Bull Terrier	Greyhound	Staffordshire Bull Terrier
Bulldog, American	**K**	**V**
Bullmastiff	Kerry Blue Terrier	Vizsla
**C**	**L**	**W**
Cairn Terrier	Labrador Retriever	Weimaraner
Cane Corso	**M**	Welsh Springer Spaniel
Cardigan Welsh Corgi	Mastiff	West Highland White Terrier
Cavalier King Charles Spaniel	McNab	Whippet
Chesapeake Bay Retriever	Miniature Schnauzer	
Chihuahua	**O**	
Chinese Shar-Pei	Old English Sheepdog	
Cocker Spaniel		
Collie		

**Table 4:** List of all cancer types represented in cancer-diagnosed subjects from the training and testing sets of the CANDiD study.

**A**	**M**
Abdominal Cavity	Malignant Melanoma
Adrenal Gland*	Mammary Gland Carcinoma
Anal Sac Adenocarcinoma	Mast Cell Tumor
**B**	Multiple Myeloma*
Bile Duct	**N**
Bone, Fibrosarcoma*	Nasal Cavity and Paranasal Sinuses
Bone, Multilobular Osteochondrosarcoma*	Nasal Planum*
Bone, Osteosarcoma	**O**
Brain	Oral Cavity
**C**	Ovary*
Chondrosarcoma	**P**
**E**	Pancreas, Endocrine*
Ear Canal	Peripheral Nerve Sheath
**H**	Pituitary*
Heart Base	Prostate*
Hemangiosarcoma	**S**
Histiocytic Sarcoma	Salivary Gland
**K**	Skin
Kidney	Soft Tissue Sarcoma
**L**	Spinal Cord*
Large Intestine*	Stomach^§^
Leukemia, Acute Lymphoid (ALL)	**T**
Leukemia, Chronic Lymphoid (CLL)	Thymoma*
Liver	Thyroid
Lung	Transmissible Venereal Tumor
Lymphoma, Indolent	**U**
Lymphoma, Intermediate to Large Cell	Urinary Bladder / Urethra

*Cancer types for which no cancer signal was detected in any cancer-diagnosed subjects in the training and testing sets in the CANDiD study.

^§^Present in one subject with a Cancer Signal Detected result that had one other concurrent primary cancer type.

Cancer-diagnosed subjects in the training and testing sets in the CANDiD study were assigned to one of 42 cancer types (listed above), based primarily on anatomical location. This simplified classification was adapted from Withrow and MacEwen’s Small Animal Clinical Oncology (Sixth Edition) and from the American Joint Committee on Cancer (AJCC) Manual (Eighth Edition).

This simplified list was derived from a more detailed list of 82 cancer subtypes that were additionally defined based on anatomical sub-location and/or histology, as shown in S2 Table.

### Overall performance

In the 876 subjects in the testing set, there were 202 *Cancer Signal Detected* (positive) results and 670 *Cancer Signal Not Detected* (negative) results. In four subjects (one cancer-diagnosed subject and three presumably cancer-free subjects), genomic alterations were detected but their significance was uncertain; these subjects received *Indeterminate* results and were excluded from analysis of test performance.

There were 351 cancer-diagnosed subjects in the testing set that received a positive or a negative result. In these subjects, the test returned a *Cancer Signal Detected* (positive) result for 192 subjects, for an overall sensitivity (detection rate) of 54.7% (192/351; 95% CI: 49.3–60.0%) (Table 5). There was no significant difference in test sensitivity based on age, weight, sex, or breed status (purebred vs mixed-breed) of the cancer-diagnosed subjects (S3 Table).

**Table 5:** Test results and performance in the testing set of the CANDiD study.

Testing Set	Test Results	Test Performance^§^
Cancer-diagnosed subjects (n = 352)	*Cancer Signal Detected* 192/351*	Sensitivity 54.7% (95% CI: 49.3–60.0%)
Presumably cancer-free subjects (n = 524)	*Cancer Signal Not Detected* 511/519**	Specificity 98.5% (95% CI: 97.0–99.3%)

*One of the cancer-diagnosed subjects received an Indeterminate test result and was excluded from analysis of test performance.

^**^Three of the presumably cancer-free subjects received Indeterminate test results and were excluded from analysis of test performance. Additionally, two of the presumably cancer-free subjects received Cancer Signal Detected results and were diagnosed with cancer following confirmatory cancer evaluations; these two subjects were also excluded from analysis of test performance.

^**§**^There was no significant difference in Test Performance between the testing set and the training set. Training set: detection rate 49.4% (p = 0.4583), true negative rate 97.2% (p = 0.5204).

There were 521 presumably cancer-free subjects in the testing set that received a positive or a negative result. In these subjects, the test returned a *Cancer Signal Not Detected* (negative) result for 511 dogs, and a *Cancer Signal Detected* (positive) result for 10 dogs (“putative false positives”, pFP) (S4 Table). Two of these 10 pFP subjects were diagnosed with cancer after undergoing a confirmatory cancer evaluation triggered by a *Cancer Signal Detected* result and were excluded from analysis of test performance, as they could no longer be presumed cancer-free. The remaining 519 presumably cancer-free subjects, including 8 subjects that received a *Cancer Signal Detected* result, were used for the calculation of specificity. The overall specificity of the test at the time of manuscript submission was 98.5% (511/519; 95% CI: 97.0–99.3%), corresponding to a false positive rate (FPR) of 1.5% (8/519; 95% CI: 0.7–3.0%) (Table 5).

One of the two pFP subjects excluded from analysis of test performance (Subject 0374 in S4 Table) was diagnosed with hemangiosarcoma 5 months following collection of the blood sample and is described in the case study section below. In the second pFP subject excluded from analysis (Subject 0148 in S4 Table), a *Cancer Signal Detected* result with “hematological malignancy” signal origin prediction led to fine needle aspiration cytology of a normal-sized popliteal lymph node; a clonal B-cell population was suspected on PARR (PCR for Antigen Receptor Rearrangements) but could not be confirmed due to low cellularity of the sample. The day following the collection of the lymph node sample, the subject died due to unrelated aspiration pneumonia. Necropsy did not reveal evidence of lymphoma; however, PARR performed on spleen and intra-abdominal lymph node samples collected at necropsy revealed a clonal B-cell population consistent with a diagnosis of lymphoma, 19 months after collection of the blood sample.

In addition to the 10 pFP subjects described above, 2 subjects that had originally been enrolled as presumably cancer-free received *Cancer Signal Detected* results, but were independently diagnosed with cancer prior to receiving the liquid biopsy test results: one patient with a monostotic aggressive bone lesion consistent with a malignant primary bone tumor at 1 month, and one with lymphoma at 6 months, respectively, following collection of the blood samples. These 2 subjects were excluded from the validation set because they could no longer be considered as presumably cancer-free.

In summary, among 12 presumably cancer-free subjects who received a *Cancer Signal Detected* result, two received a cancer diagnosis based upon a confirmatory cancer evaluation triggered by the liquid biopsy test result, and 2 had been independently diagnosed with cancer prior to receiving the liquid biopsy test result. Of the remaining 8 pFP subjects, 4 died without a confirmed diagnosis of cancer, and 4 had cancer evaluations but cancer was not found and these patients continue to be monitored for evidence of cancer. Because a diagnosis of cancer could not be confirmed for these 8 pFP subjects as of the time of manuscript submission, they were considered false positives for the purpose of determining test specificity.

### Performance in three of the most aggressive canine cancers

Lymphoma, hemangiosarcoma, and osteosarcoma are highly aggressive canine cancers. In the testing set, there were 137 subjects with a single primary cancer belonging to one of these three cancer types; all received a positive or negative result. The liquid biopsy test returned a positive result in 117 of these 137 subjects, resulting in an overall detection rate of 85.4% (95% CI: 78.4–90.9%) across these three aggressive cancer types (Table 6).

**Table 6:** Test results and performance in the testing set for three of the most aggressive canine cancers.

Three of the Most Aggressive Canine Cancers *(Lymphoma*, *Hemangiosarcoma*, *Osteosarcoma)*		
Testing Set	Test Results	Test Performance
Number of subjects tested (n = 137)	*Cancer Signal Detected* 117/137	Detection rate 85.4% (95% CI: 78.4–90.9%)

### Performance in eight of the most common canine cancers

Certain types of cancer are common in dogs and account for most of the cancer deaths in the species, namely: lymphoma, hemangiosarcoma, osteosarcoma, soft tissue sarcoma, mast cell tumor, mammary gland carcinoma, anal sac adenocarcinoma, and malignant melanoma [40]. In the testing set, there were 237 subjects with a single primary cancer belonging to one of these eight cancer types; of these, 236 received a positive or negative result. The liquid biopsy test returned a positive result in 146 of these 236 subjects, resulting in an overall detection rate of 61.9% (95% CI: 55.3–68.1%) across these eight common cancer types (Table 7).

**Table 7:** Test results and performance in the testing set for eight of the most common canine cancers.

Eight of the Most Common Canine Cancers *(Lymphoma*, *Hemangiosarcoma*, *Osteosarcoma*, *Soft Tissue Sarcoma*, *Mast Cell Tumor*, *Mammary Gland Carcinoma*, *Anal Sac Adenocarcinoma*, *Malignant Melanoma)*		
Testing Set	Test Results	Test Performance
Number of subjects tested (n = 237)	*Cancer Signal Detected* 146/236*	Detection rate 61.9% (95% CI: 55.3–68.1%)

*One of the cancer-diagnosed subjects in the testing set (diagnosed with mast cell tumor) received an Indeterminate test result and was excluded from analysis of test performance.

### Performance by cancer type

**Fig 2:**
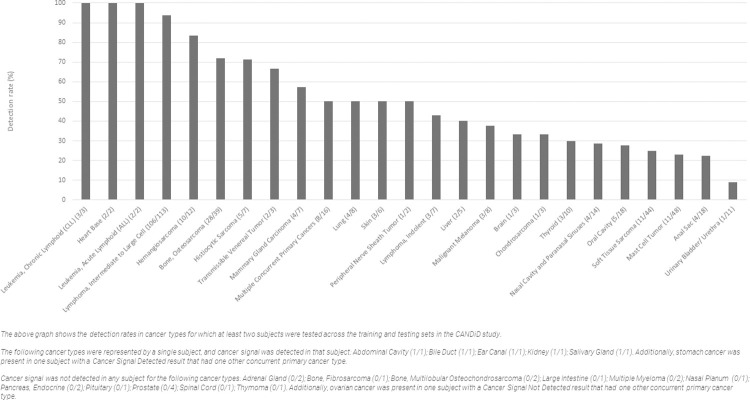
Detection rates by cancer type.

Among the 432 cancer-diagnosed subjects across the training and testing sets that received a positive or a negative result, there were 416 subjects with a confirmed diagnosis of a single primary cancer, representing 40 cancer types (Table 4); and 16 subjects with confirmed diagnoses of multiple concurrent primary cancers representing a total of 17 distinct cancer types, 2 of which were only observed in these 16 subjects (S5 Table). Samples from all cancer-diagnosed subjects in the study were processed using the same standardized workflow and analyzed using the same bioinformatics pipeline. The detection rates of the test by cancer type across all cancer-diagnosed subjects in the training and testing sets are shown in Fig 2.

### Performance by extent of disease and tumor size

**Fig 3:**
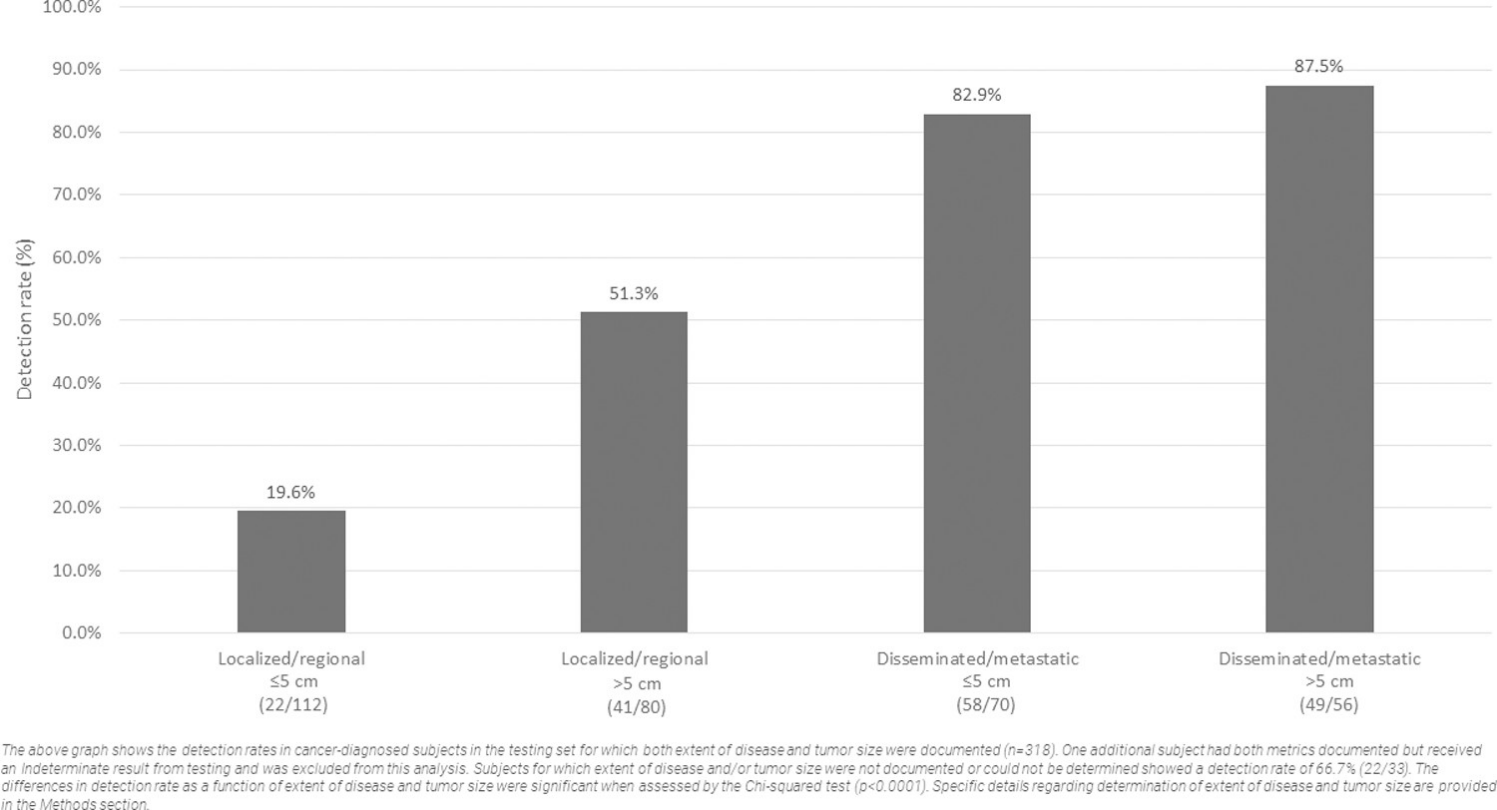
Detection rates by extent of disease and tumor size.

The test’s detection rate was calculated as a function of extent of disease (localized/regional or disseminated/metastatic) and tumor size (≤5 cm or >5 cm, for the longest diameter of the largest lesion). Results are shown in Fig 3. Detection rate increased as a function of stage and tumor size, with localized/regional disease with tumor size ≤5 cm having the lowest performance at 19.6% (22/112; 95% CI: 12-7-28.2%) and disseminated/metastatic disease with tumor size >5 cm having the highest performance at 87.5% (58/70; 95% CI: 72.0–90.8%). Subjects for which extent of disease and/or tumor size was not documented or could not be determined showed a detection rate of 66.7% (22/33; 95% CI: 48.2–82.0).

### Cancer Signal Origin (CSO) prediction

A CSO algorithm for hematological malignancy was developed based on genomic features associated with hematological malignancies, such as copy number gains in chr13 and/or chr31, copy number losses in chr14 [41, 42], and/or penetration of somatic genomic alterations into gDNA. The algorithm was developed using subjects diagnosed with hematological malignancies in the training set; the algorithm was then applied to subjects in the testing set who received a *Cancer Signal Detected* result. A total of 96 subjects with a diagnosis of hematological malignancy in the testing set received a *Cancer Signal Detected* result. Of these 96 subjects, 40% (n = 38) were also assigned a CSO prediction of “hematological malignancy”, while the remaining 58 did not receive a CSO prediction. Three subjects who had been diagnosed with non-hematological malignancies were additionally assigned a CSO prediction of “hematological malignancy”, for an overall CSO prediction accuracy of 92.7% (38/41).

### Test performance as a function of pre-analytical variables

Neither the sensitivity nor the specificity of the test had a significant association with any of the following pre-analytical variables in the testing set: time from collection to processing, extent of hemolysis, and extent of lipemia (S6 Table).

### Case study: Liquid biopsy reveals cancer signal months prior to onset of clinical signs

The following case study presents an adjudicated putative false positive (pFP) result and illustrates the ability of NGS-based liquid biopsy to detect cancer-associated genomic alterations in blood months before the onset of clinical signs.

**Fig 4:**
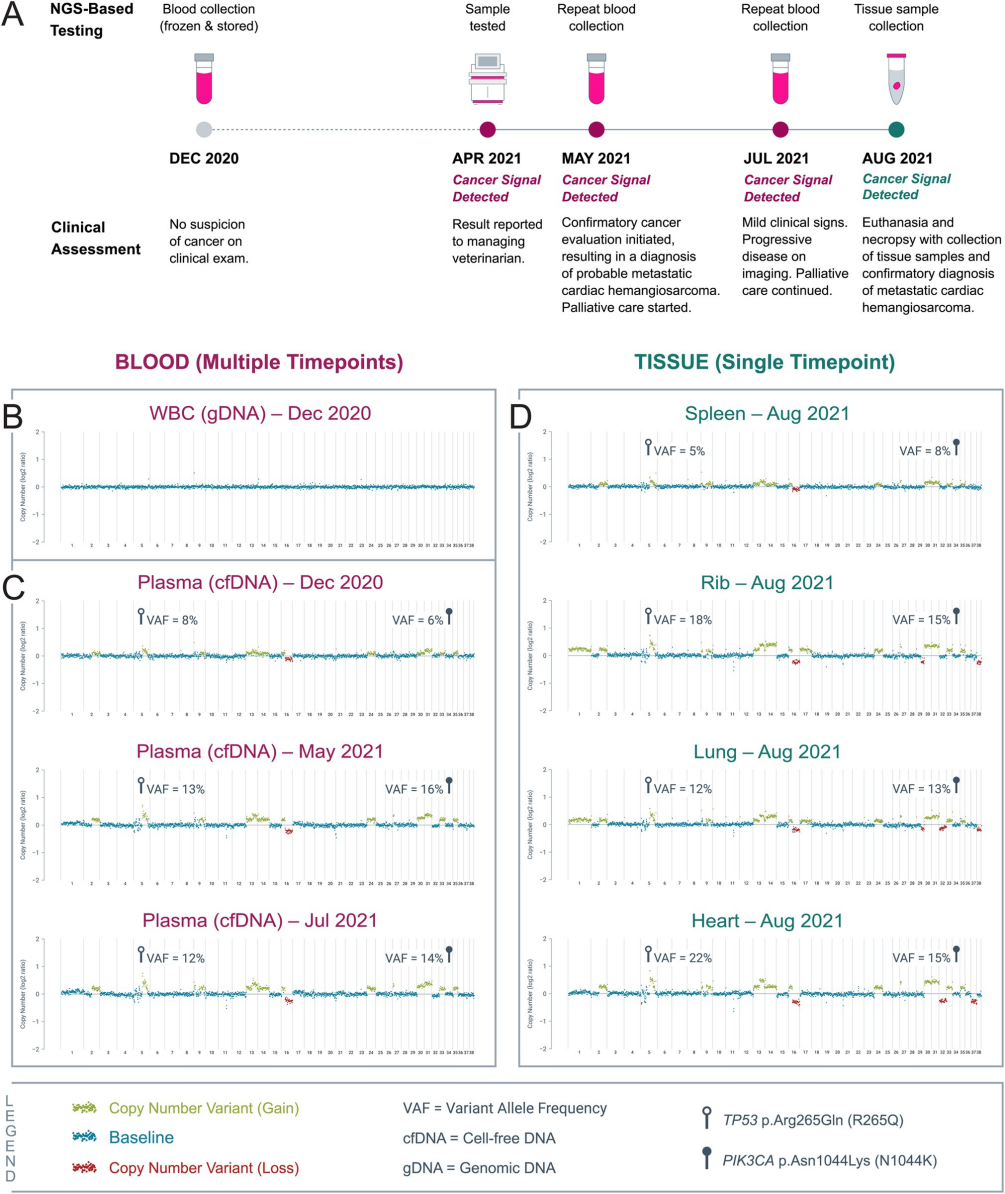
Case study of a presumably cancer-free 7-year-old, 37 kg female spayed mixed-breed dog that received a *Cancer Signal Detected* result and was subsequently diagnosed with hemangiosarcoma. (A) Timeline of NGS-based testing of blood and tissue samples, and the corresponding clinical assessments for the subject. (B) Sequencing data showing no genomic alterations in gDNA derived from white blood cells. (C) Sequencing data showing genomic alterations present in cfDNA from plasma samples obtained at multiple timepoints. (D) Sequencing data showing genomic alterations present in various tissue samples collected during necropsy.

The subject was a 7-year-old, 37 kg female spayed mixed-breed dog initially enrolled in the presumably cancer-free cohort in December 2020. At the time of enrollment, the subject had no suspicion of cancer based on physical exam findings or clinical signs noted by the owner. Blood was collected, and plasma and WBCs were separated upon sample receipt and stored at -80°C until liquid biopsy testing was performed in April 2021; at that time, a *Cancer Signal Detected* result was issued. Genomic alterations detected in cfDNA from the plasma sample included copy number variants (CNVs) across the genome, and single nucleotide variants (SNVs) in two genes that are frequently mutated in human and canine cancers: *TP53* and *PIK3CA* [43]. Both mutations, *TP53* R265Q and *PIK3CA* N1044K, have human orthologues (*TP53* R213Q and *PIK3CA* N1044K, respectively) that have been observed in multiple human cancers as recorded in the COSMIC (Catalogue Of Somatic Mutations In Cancer) database [44]. In particular, *PIK3CA* N1044K is a known oncogenic mutation that is targetable in human breast cancer by an FDA-approved drug [45]. At the time of receipt of the *Cancer Signal Detected* result in April 2021, the subject continued to have a normal physical exam and no clinical signs noted by the owner. Because of the *Cancer Signal Detected* result, the subject underwent a confirmatory cancer evaluation in May 2021, five months after the initial blood sample was collected. Three-view thoracic radiographs identified nodules in the lungs, and metastatic neoplasia was suspected. A thoracic CT was subsequently performed, which confirmed the presence of the nodules in the lungs and revealed a cavitated mass in the left ventricle of the heart. Ultrasound-guided fine needle aspiration cytology of one lung nodule was consistent with probable sarcoma (suspect hemangiosarcoma). The subject was managed with palliative care and remained asymptomatic. Additional blood samples were collected in May 2021 and July 2021 for repeat liquid biopsy testing; both tests confirmed the previously detected genomic alterations in plasma. Mild clinical signs (e.g., intermittent lethargy) were first noted in July 2021, seven months after molecular signs of cancer were measurable in the blood. Clinical monitoring revealed progression of pulmonary nodules, the development of a cavitated mass in the spleen, and mild pericardial effusion. The subject did not experience clinical signs consistent with acute blood loss or tamponade and continued to be managed palliatively. Elective euthanasia was pursued in August 2021 due to clinical signs of progressive lethargy, eight months following the collection of the initial blood sample. Necropsy confirmed a splenic mass, rib mass, diffuse pulmonary nodules, and a multilobular mass infiltrating the muscle of the entire heart and extending through the pericardium; a histopathologic diagnosis of hemangiosarcoma was subsequently confirmed in all tissue sites. Further genomic analysis of the tissue samples confirmed the presence of the genomic alterations previously identified in plasma. To the authors’ knowledge, this is the first known reported case in which cancer-associated genomic alterations were shown to be present and detectable in blood several months prior to the development of clinical signs of cancer in a canine patient; and in which a positive result from an NGS-based liquid biopsy test triggered a cancer workup in a preclinical patient that ultimately resulted in a confirmed diagnosis of cancer (Fig 4).

## Discussion

A novel, NGS-based MCED liquid biopsy test has demonstrated the ability to identify cancer-associated genomic alterations in canine patients representing a wide spectrum of cancer types. To the authors’ knowledge, this is the first NGS-based test in the veterinary space that leverages a robust training and testing study design for algorithm development and performance validation, respectively. A large, representative training set was used to optimize and lock the test’s algorithms and thresholds. This locked pipeline was separately applied to an independent testing set for validation of clinical performance. Use of an independent testing set for validation is critical, as reporting performance from the same dataset used in development may result in inflated estimates of test performance due to overfitting. Validation on a fully independent testing set demonstrates robust generalizability of the test’s clinical performance characteristics [38, 39]. Cumulatively, the training and testing sets comprised over 1,000 canine subjects. To the knowledge of the authors, this is the largest clinical validation study ever published in veterinary cancer diagnostics.

### Clinical sensitivity and specificity

In the testing set, the test was able to detect three of the most aggressive canine cancers (lymphoma, hemangiosarcoma, osteosarcoma) at an overall detection rate of 85.4%. The test also demonstrated an overall detection rate of 61.9% in the testing set for a subset of eight commonly encountered canine cancers: lymphoma, hemangiosarcoma, osteosarcoma, soft tissue sarcoma, mast cell tumor, mammary gland carcinoma, anal sac adenocarcinoma, and malignant melanoma. The overall sensitivity of the test across all cancer-diagnosed subjects in the testing set was 54.7%. The majority (58%) of cancer-diagnosed subjects had *localized/regional* disease.

These detection rates were achieved at a specificity of 98.5% in the testing set, corresponding to a false positive rate of 1.5%. This compares favorably to false positive rates in the range of 3.5% to >10% observed historically in screening programs focused on specific cancer types in human patients [46–49]. High specificity is important because false positives can impose significant financial and psychological costs, and can lead to adverse events resulting from unnecessary diagnostic interventions. For these reasons, two MCED tests recently developed for annual cancer screening in humans have been optimized for specificity (>99.0% in both cases) [50, 51].

Importantly, the specificity of the test was not determined in a cohort of “healthy” dogs. Rather, control subjects were enrolled as “presumably cancer-free” based on having no history of cancer and no suspicion of cancer on clinical evaluation at the time of study enrollment; there were no restrictions on the type or number of medical conditions (other than confirmed or suspected cancer) that could be present in these subjects. The use of “presumably cancer-free" rather than “healthy” subjects was deliberately chosen in order to document the real-world specificity of the test, given that many patients in whom such a test might be used (for screening in older dogs at higher risk of cancer, or for aid-in-diagnosis in dogs suspected of cancer based on clinical presentation) may have other concurrent medical conditions. Achieving a very low false positive rate (1.5%) in a large control group of study subjects with a variety of non-malignant medical conditions was made possible by the fact that NGS-based approaches rely on detection of genomic alterations that are associated with cancer and are not typically encountered in other disease states. The test’s high specificity for cancer also allowed for unambiguous, binary reporting of results (*Cancer Signal Detected* or *Cancer Signal Not Detected*) as opposed to using a continuous risk score or multiple risk categories, which can create interpretation challenges in the clinic.

### Tumor size and extent of disease

The relationship between cancer size and/or spread, and likelihood of detection by liquid biopsy, has been well established in human cancers [52, 53]. A large clinical validation study of an MCED test in humans recently demonstrated the following sensitivity performance (at a specificity of 99.5%): 16.8% for Stage I, 40.4% for Stage II, 77.0% for Stage III, and 90.1% for Stage IV cases [50]. A similar trend was observed in the current study when comparing test sensitivity across extent of disease and tumor size categories in dogs (at a specificity of 98.5%): 19.6% and 51.3% for *localized/regional* cases with ≤5 cm and >5 cm lesions, respectively; 82.9% and 87.5% for *disseminated/metastatic* cases with ≤5 cm and >5 cm lesions, respectively; and 66.7% for cases in which extent of disease and/or tumor size was not documented or could not be determined (Fig 3). The measurement of tumor size relied on the longest diameter of the single largest lesion in each cancer-diagnosed patient; this metric was a reliable indicator of total tumor burden in *localized/regional* cases, but not in *disseminated/ metastatic* cases–where subjects typically had multiple malignant lesions, and the size of the single largest lesion did not provide an accurate reflection of overall tumor burden.

These findings have implications for the clinical utility of this type of testing in both the screening and the aid-in-diagnosis scenarios. For example, a smaller (≤5 cm) lesion that is suspected to represent localized malignancy and is easily accessible by biopsy or fine needle aspiration (FNA) should likely be pursued with conventional tissue sampling rather than with liquid biopsy, given the lower sensitivity of the latter for smaller, localized cancers. However, in a screening situation where there is no prior suspicion of cancer, or in a scenario where a suspicious lesion is identified on imaging in a difficult-to-access anatomical location, a test that can detect smaller localized cancers with high specificity through a simple blood draw could offer significant clinical utility even at lower levels of sensitivity, given the lack of alternatives.

This utility may extend to certain disseminated/metastatic cases. Dogs may not show clinical signs until cancer has advanced to a stage where it is no longer curable at the time of diagnosis. In such situations, preclinical detection of disseminated/metastatic disease may nevertheless provide significant utility to the clinician and the pet owner. For example, it can help to shorten the path to diagnosis, or allow the diagnostic workup to take place without the time constraints that may exist when patients present with acute clinical signs–as, for example, in many hemangiosarcoma cases wherein cancer is detected after development of potentially life-threatening hemorrhage; it may also allow for palliative care to be initiated earlier, for improved quality of life; and it might give the family more time to make important medical management decisions for their pet. The case study presented above (Fig 4) illustrates these multiple elements of clinical and personal utility in a canine patient with metastatic disease who was diagnosed at a preclinical stage following a *Cancer Signal Detected* liquid biopsy test result.

### Positive and negative predictive values

Though sensitivity and specificity are important test performance metrics, the positive predictive value (PPV) associated with an abnormal result is arguably a more relevant number for clinicians, as it describes the probability that a particular patient has cancer, given the positive liquid biopsy test result and his/her unique clinical presentation. Similarly, in the case of a negative result, the negative predictive value (NPV) helps the clinician determine the level of reassurance that can be provided regarding the absence of cancer.

Positive predictive values (PPV) and negative predictive values (NPV) can be calculated based on the estimated prevalence of cancer in the intended use population(s), using the following standard formulas: PPV=(sensitivityxprevalence)/[sensitivityxprevalence+(1−specificity)x(1−prevalence)] and NPV=[specificityx(1−prevalence)]/[(1−sensitivity)xprevalence+specificityx(1−prevalence)]

The test is currently intended for use in dogs who are at higher risk for cancer: as an annual screening test for dogs at higher risk of cancer due to age and/or breed; and as an aid-in-diagnosis for dogs in which cancer is suspected based on clinical signs or other clinical findings. Though the prevalence of cancer in these high-risk populations has not been conclusively established, it is possible to estimate the prior probability of cancer for each population from existing data sources.

It is important to note that the term “screening” is used here in a strict sense. The American Cancer Society (ACS) states that “Screening tests are used to find cancer *before* a person has any symptoms” (emphasis in the original) [54]; and the National Cancer Institute (NCI) states that “Checking for cancer… in people who have no symptoms is called screening” [55]. Veterinary patients do not have symptoms, but they do have clinical signs [56]. In a veterinary context, cancer screening therefore refers to investigations undertaken in patients that are at higher risk of cancer (e.g., due to age or breed) but do not have a current suspicion of cancer based on clinical presentation. Tests performed in patients already suspected of having cancer are not “screening” tests; the term “aid-in-diagnosis" provides a more accurate description for test use in such clinical scenarios.

In the United States, it is estimated that 4.2 to 6 million dogs receive a new cancer diagnosis each year [57, 58] out of a total population of approximately 65 to 77 million dogs [58, 59]. Therefore, the estimated annual incidence of canine cancer is approximately 5.5% to 9.2% across all dogs. Because the liquid biopsy test is intended for annual screening in dogs at higher risk of cancer based on age or breed, a conservative annual incidence of cancer in this screening population may be estimated at 8–10%. Though studies of canine cancer incidence are limited, available data provide support for this estimate in higher risk populations [60].

When used as an aid-in-diagnosis, the test’s PPV is expected to increase due to the higher *a priori* cancer prevalence in the population being tested. A survey conducted in June 2020 collected data from over 300 veterinary general practitioners in the United States and found that approximately 50% of dogs that present with a clinical suspicion of cancer go on to receive a definitive or presumptive diagnosis of cancer (S1 Fig). Therefore, for the purpose of PPV and NPV calculations in this study, a cancer prevalence of 30–50% was used for the aid-in-diagnosis population.

Using the established overall test performance metrics (54.7% sensitivity, 98.5% specificity), and the estimated cancer prevalence in each of the intended use populations (8–10% in the screening population, and 30–50% in the aid-in-diagnosis population), PPVs and NPVs can be calculated for the liquid biopsy test. As shown in Table 8, when a positive result is issued for a screening patient, the PPV is estimated to be 76–80%. PPV rises to 94–97% when a positive result is issued for an aid-in-diagnosis patient, due to the increased prevalence of cancer in this population. In the context of a negative liquid biopsy result for a screening patient, NPV is expected to range from 95–96%, providing a high level of reassurance for the absence of cancer at the time of screening. In the aid-in-diagnosis population, where there is already a clinical suspicion of cancer, the prior probability is higher than in the screening setting, resulting in an NPV in the range of 68–84%. This lower NPV range underscores the importance of further clinical evaluation if the liquid biopsy test is negative as an aid-in-diagnosis, but cancer remains high on the list of differential diagnoses for that patient.

**Table 8:** Positive and negative predictive values based on estimated prior probabilities of cancer in two intended use populations.

			Positive test result	Negative test result
Clinical use case	Prior probability of cancer	Intended use population	PPV	NPV
Screening	8–10%	Higher risk of cancer due to age and/or breed	76–80%	95–96%
Aid-in-diagnosis	30–50%	Cancer suspected based on clinical presentation	94–97%	68–84%

Estimated ranges for positive predictive value (PPV) and negative predictive value (NPV), calculated using a test sensitivity of 54.7% and specificity of 98.5% (range is calculated using the lower and higher ends of prior probability).

### Cancer screening using an MCED test

Traditional cancer screening programs in humans have focused on one cancer type per screening test. For example, a patient would undergo mammography, Pap smear, and colonoscopy to specifically screen for breast, cervical, and colon cancer, respectively [9]; a true positive result for a given test would detect the targeted cancer type but miss any other malignancies that might be present in the patient’s body. In contrast, MCED tests are designed to detect as many cancer types as possible with a single testing method, to maximize the cancer detection rate across the entire at-risk population (number of cases detected as a fraction of all actual cancer cases present in the population). Thus, an overall detection rate of 54.7% across 30 cancer types in a screening population corresponds to a larger absolute number of cancer cases detected than the detection rate of 85.4% in a restricted set of “three of the most aggressive cancers” or 61.9% in a restricted set of “eight of the most common cancers”. Furthermore, in a true screening setting (where the patient has no current suspicion of cancer) there is no firm basis for suspecting a particular cancer type at the outset versus all other possible cancer types that might be present at a preclinical stage in that patient. As with MCED testing in humans, the first goal of screening in dogs is to determine if cancer (of any type or stage) is present in a preclinical patient, before pursuing cancer typing and staging. These screening principles underscore the fact that an MCED test’s PPV is more clinically relevant for any given patient than the test’s sensitivity. As previously noted, the overall sensitivity of this test coupled with its very high specificity for cancer support a PPV estimate above 75% in a canine high-risk screening population; this is significantly higher than the PPVs of 5–21% that have been reported for various clinical symptoms, signs, and non-diagnostic tests in human medicine and are considered highly predictive for cancer in general practice settings [61].

Given these considerations, it is important to note that MCED tests are intended to be used in a manner that fits with a patient’s overall clinical picture. These tests are not a replacement for conventional diagnostic and staging tests, and they should not serve as the sole basis for making important decisions such as treatment selection or euthanasia. Rather, their intent is to assist in the detection of cancer by noninvasive means. The clinical utility of MCED testing is the subject of ongoing research in human medicine, including evaluation of the impact on multiple aspects of patient care and disease management [62–65]. Similar studies will be required to understand the full clinical implications of MCED testing in veterinary medicine.

### Study limitations

A potential limitation of this study is its “case-control” design, wherein all subjects used to determine test performance had already been classified as either “cancer-diagnosed” or “presumably cancer-free” based on prior clinical evaluation. Most of the “cancer-diagnosed” subjects had initially presented for veterinary care due to clinical signs of disease, and subsequently received a definitive diagnosis of malignancy. Many subjects in a real-world screening setting would be expected to have sub-clinical disease; therefore, the sensitivity of the test (at a single point in time) for the screening use case is likely to be somewhat lower than the sensitivity reported here. The currently reported sensitivity is more likely to be reflective of real-world sensitivity in the aid-in-diagnosis use case, where patients receive the test because they are currently suspected of cancer based on observable clinical signs. This consideration regarding the screening use case should be balanced with the possibility that, when screened at regular intervals (e.g., on an annual basis), dogs at higher risk of cancer might benefit from a lifetime “cumulative detection rate” significantly higher than the sensitivity documented in this study at just one point in time; this is because each successive test provides an additional opportunity for detection, if cancer is indeed present [66]. In general, as cancer increases in size and becomes more aggressive, more ctDNA will be shed into circulation, increasing the chances of detection by liquid biopsy. The relationship between cancer size and likelihood of detection by liquid biopsy is well established in human cancers [52, 53] and was also observed in this study when comparing test sensitivity across extent of disease and tumor size categories in dogs.

Of note, each subsequent test also provides an additional opportunity for a false positive to be reported, increasing the probability that a given patient will receive a false positive screening result at some point during a multi-year period of repeat testing [67]. This highlights the value of using a screening test with a high specificity (low false positive rate), as well as the importance of understanding the actual window of opportunity for preclinical detection of various cancer types in dogs. For example, one putative false positive subject in this study (Subject 0148 in S4 Table) was diagnosed with lymphoma 19 months following collection of a blood sample that received a *Cancer Signal Detected* result; given the long duration of this elapsed period, it is unclear if the positive test result was in fact related to the eventual cancer diagnosis. In humans, the latency periods of many cancer types have been extensively researched. A recent analysis estimated a latency range of 2.2 years to 35.7 years for lymphoproliferative and hematopoietic cancers, and 6.6 years to 57 years for solid malignancies; 35 of the 44 cancer types in this analysis were found to progress silently for 10 years or longer prior to detection, representing 89% of the patients in the analysis [68]. This provides a considerable window of opportunity for preclinical identification of cancer in human patients by various screening approaches, including multi-cancer early detection (MCED) testing [15, 63]. The duration of the preclinical phase is not well documented for any canine cancer type, but it is plausible that many dogs may have preclinical disease for months to years before onset of clinical signs. Lifetime screening studies are needed to better understand the duration of the preclinical phase in dogs; the performance of NGS-based liquid biopsy for detecting preclinical cancer in canine patients; the incremental benefits of cumulative sensitivity with interval screening; and the ultimate clinical utility of earlier detection (including impact on clinical management decisions, and actual survival benefit after accounting for lead-time bias) for various cancer types. To explore these important topics, the Cancer Lifetime Assessment Screening Study in Canines (CLASSiC) was launched in December 2021 (PetDx, La Jolla, CA); the study aims to prospectively follow over 1,000 initially cancer-free dogs, with semi-annual liquid biopsy testing and comprehensive documentation of cancer-related clinical outcomes, over many years.

Another limitation is the fact that only one sample (collected around the time of initial cancer diagnosis) was tested for each cancer-diagnosed subject as part of this validation study; as a result, the performance of the test for post-diagnosis longitudinal use cases (such as minimal residual disease detection, recurrence monitoring, and treatment response monitoring) has not been determined. Over 1,000 serial blood samples were obtained from cancer-diagnosed subjects as part of the prospective collection that powered this study, along with clinical data such as the nature and timing of therapeutic interventions, time to recurrence, and extent of disease at recurrence. Future validation studies using these samples are planned to demonstrate the performance of the test in disease-monitoring use cases.

Another potential limitation of this study was that clinical status designations (such as benign vs malignant tumor, and cancer type, stage or size) were collected as provided by the managing veterinarian(s) according to the standard of care at each collection site. This information was independently reviewed and adjudicated by the central study team based on source documents (including but not limited to clinical intake and progress notes, imaging reports, surgery reports, and histopathology reports) prior to database lock. However, the original radiologic images and pathology samples were not independently reviewed by the central study team. Thus, there exists the possibility that the clinical status designations attributed to each subject may not have captured the true clinical status of every subject in the study, given the well-documented variability in interobserver concordance for imaging, pathology, and other clinical assessment areas [69–74].

Finally, many cancer types were represented by only one or a few subjects in the all-comers collection used in this study, making it difficult to determine test performance in those cancer types with a high degree of precision. Further research is planned in order to study the performance of the test in larger numbers of cases for specific cancer types, and the list of detectable cancer types is expected to increase with testing of larger cohorts and with future enhancements to the test. The value of an MCED testing approach lies in its ability to detect cancer-associated genomic alterations across a broad range of cancer types, and to expand the set of detectable cancers over time. As described previously, with each addition to the MCED test’s list of detectable cancer types, both the fraction and the absolute number of cancer cases detected in the tested population will increase (even if the test’s detection rate is low for the individual cancer type being added) [50].

### Challenges and potential confounders

In humans, some cancers are more challenging to detect by liquid biopsy. For instance, it has been shown that ctDNA in blood has a lower detection rate in certain cancer types, such as malignancies of the central nervous system (CNS) or prostate [52, 75]. This observation aligns with the results of this study, where the detection rate in subjects with CNS tumors and prostate cancer, for example, is also low. Further research is needed to determine if NGS-based liquid biopsy tests can be optimized to improve detection of biologically challenging malignancies in blood, or whether other bodily fluids offer better conduits for detection of these cancers [76–78].

The ability to detect a malignant process with very high specificity through a simple blood test can, by itself, provide significant value by helping the clinician narrow the differential diagnosis from among other potential pathological processes in a canine patient. Prediction of a specific *Cancer Signal Origin* for patients with a C*ancer Signal Detected* result would further enhance the utility of the test, as it would help focus the cancer evaluation process and ideally lead to a definitive diagnosis earlier and at lower cost for the pet owner. This study demonstrated the ability of the test to provide CSO prediction for canine hematological malignancies, the most common of which are lymphomas. As more subjects are tested and adjudicated, and larger datasets are accumulated for additional cancer types, bioinformatics algorithms may be developed and optimized to provide accurate CSO predictions for additional cancer types, including those that are less commonly encountered in dogs. Such capabilities have already been documented for MCED tests developed for use in humans, with accurate CSO prediction demonstrated for various cancer types in large numbers of subjects representative of each cancer type [50, 51]. The overall sensitivity and specificity of the test can also be expected to increase over time, with further biochemical and bioinformatics improvements and new learnings gained from testing larger sample sets.

It is important to note that certain biological factors could confound results of NGS-based liquid biopsy tests. Viable chromosomal aneuploidies, constitutional mosaicism, genomic alterations present in one or more fetuses of a pregnant female or in a transplanted organ (including bone marrow), and clonal hematopoiesis of indeterminate potential (CHIP), among others, have the potential to confound results from blood-derived DNA in humans [28–30, 79–83]. The robust design of this test provides for parallel testing of matched gDNA and cfDNA from each sample; the use of an intra-subject gDNA control as part of each test is intended to mitigate the impact of certain confounders noted above. The very high specificity observed in the large all-comers cohort of presumably cancer-free dogs in this validation study suggests that such confounders, if present, would rarely lead to false positive results among canine patients receiving this test in a routine veterinary practice setting.

Masses designated as “benign” by tissue pathology require special consideration as potential confounders and present an opportunity for future research. Tumors exist on a continuum from benign to borderline (premalignant) to malignant, and sometimes these states can coexist (e.g., in a patient where parts of a benign tumor are undergoing malignant transformation) [84–86]. The possibility of tumor heterogeneity across spatially separated sites makes it difficult to ascertain the absence of malignancy in any given subject found to have benign disease based on tissue sampling from a single anatomical site, especially when interobserver variability for benign vs malignant diagnoses (well documented across many tumor types) is additionally considered [22, 87–96]. Cases with benign pathological diagnoses were excluded from analysis in this study for two reasons. First, in the context of the above considerations, the absence of malignancy could not be ascertained in such cases, so they could be used for neither the calculation of sensitivity (which required cancer-diagnosed subjects) nor the calculation of specificity (which required subjects without a diagnosis or a suspicion of cancer). Second, it has been established in human oncology that patients with a history of benign masses have an elevated relative risk of malignancy in multiple organs, such as breast [97, 98], uterus [99], colon [100], and liver [101, 102]. For perspective, two recent large clinical validation studies for MCED tests intended for use in humans also excluded potentially confounding cases (such as those with high-grade dysplasia or with suspected but unconfirmed cancer status at the time of blood collection) from their analysis, and neither study provided an analysis of subjects with benign tumors [50, 51]. Although excluded from the current analysis, canine subjects with benign pathological diagnoses at enrollment in this study will continue to be monitored for long-term clinical outcomes, which will be evaluated alongside genomic analyses of both tissue and blood samples.

This study also evaluated the impact of multiple pre-analytical factors that have the potential to confound the accuracy of test results; these factors have direct relevance for sample collection and handling workflows in the clinic, and for dog owner convenience. The use of specialized blood collection tubes allowed for whole-blood samples to be shipped to the central lab without any processing in the clinic, and without any sample handling requirements for temperature control in the clinic and/or in-transit. Samples were shipped at ambient temperature from many domestic and international sites located in various climates, and were accepted for testing if they arrived at the central lab within 7 days of collection. There was no difference in test performance as a function of days in-transit, within the acceptance period. Hemolysis and lipemia are also common confounders for blood-based tests. Study subjects were enrolled without any restrictions related to the timing of the dog’s last feeding, and lipemia scores (which serve as a proxy for the timing of the most recent meal) [103] had no significant association with test performance. Likewise, hemolysis scores had no significant association with test performance. Finally, there was no significant association between test performance and the following subject-specific characteristics: age, weight, sex (including spay/neuter status), and purebred vs mixed-breed pedigree. Taken together, these findings demonstrate the robustness and versatility of an NGS-based liquid biopsy test for multi-cancer detection in real-world conditions.

Other non-biological factors could also confound test results, such as sample swap (at the clinic or laboratory), sample contamination, laboratory processing artifacts, and bioinformatics limitations, among others.

In light of the multiple biological and non-biological factors that have the potential to confound NGS-based liquid biopsy testing, results that are suspected to be “false positive” or “false negative” should be comprehensively evaluated: in some cases, by repeat liquid biopsy testing, and in all cases by incorporating the clinician’s intuition and firsthand knowledge of each patient’s clinical situation. In particular, positive results from liquid biopsy testing in presumably cancer-free dogs (such as in a screening setting) must be adjudicated by a confirmatory cancer evaluation; as demonstrated by multiple subjects in the control cohort, including the case study presented, liquid biopsy has the ability to detect cancer-associated genomic alterations many months prior to the onset of clinical signs. Furthermore, negative liquid biopsy results in dogs suspected of cancer on clinical grounds should be treated with caution, and a comprehensive cancer evaluation should be completed to confirm the absence of malignancy.

## Conclusion

Unlike the human diagnostics space, the veterinary diagnostics space is unregulated; as a result, peer-reviewed publication of robust clinical validation studies for new personalized-medicine solutions is critical for supporting responsible adoption and informing appropriate clinical decision making [104].

In a large clinical validation study using an independent testing set, a novel NGS-based MCED liquid biopsy test demonstrated the ability to identify cancer-associated genomic alterations in canine patients–in some cases many months prior to the onset of clinical signs–across a large and diverse set of cancer types. The test’s detection rate was particularly high in three of the most aggressive, and in eight of the most common, cancer types in dogs. When added to the veterinarian’s diagnostic toolkit, this type of noninvasive testing may enable earlier cancer detection in screening and aid-in-diagnosis clinical scenarios, and lead to a paradigm shift in the way cancer is diagnosed and managed in dogs.

Though further research is needed to explore the extent to which earlier cancer identification and treatment can improve clinical outcomes in canine patients, established screening programs for various human cancers (breast, colon, prostate, cervix, lung) [9] have historically provided strong support for improved outcomes associated with high-compliance population-level screening programs [105, 106]. Major veterinary organizations have also recognized the value of earlier diagnosis: the American Animal Hospital Association (AAHA) has stated that "early detection is critical for the best outcome” [3], while the American Veterinary Medical Association (AVMA) has advised that “neoplasia is frequently treatable and early diagnosis will aid your veterinarian in delivering the best care possible” [2]. A noninvasive cancer detection method that requires only a routine blood collection may lead to more early diagnoses in dogs simply because of the convenience associated with the testing process, which might encourage adoption as well as compliance with interval screening recommendations.

In addition to its applications in screening and aid-in-diagnosis, NGS-based liquid biopsy testing is expected to expand in use to assist veterinarians in providing highly personalized cancer care through targeted treatment selection, minimal residual disease detection (following curative-intent treatment), recurrence monitoring, and treatment response monitoring [22]. Validation studies specific to each of these post-diagnosis use cases will be required to demonstrate the performance of liquid biopsy methods in these clinical scenarios. Finally, canine and human cancers share many molecular features [57, 107]; therefore, genomic data being generated from liquid biopsy testing in canine cancer subjects may provide benefits to human cancer patients via comparative oncology analyses.

## Supporting information

S1 Fig Questions asked in a survey of over 300 veterinary general practitioners in the United States. (PDF)

S1 Table Full subject level data for subjects enrolled in the CANDiD study. (PDF)

S2 Table Cancer types and subtypes evaluated in the CANDiD study. (PDF)

S3 Table Analysis of test sensitivity based on demographic characteristics of cancer-diagnosed subjects in the testing set. (PDF)

S4 Table Overview of putative false positive subjects from the testing set of the CANDiD study. (PDF)

S5 Table Subjects with confirmed diagnoses of multiple concurrent primary cancers in the training and testing sets (n = 16). (PDF)

S6 Table Analysis of test performance based on pre-analytical variables in the testing set. (PDF)
