# Supplementary material for: Clinical validation of a next-generation sequencing-based multi-cancer early detection “liquid biopsy” blood test in over 1,000 dogs using an independent testing set: The CANcer Detection in Dogs (CANDiD) study
Source: PLoS One. 2022 Apr 26;17(4):e0266623. doi: 10.1371/journal.pone.0266623 (PMC9041869; doi:10.1371/journal.pone.0266623)
Supplement: S1 Fig — (PDF) [file pone.0266623.s001.pdf]

**S1 Fig. Questions asked in a survey of over 300 veterinary general practitioners in the United States. The survey was commissioned by PetDx in 2020 and was conducted by Kynetec, an animal health and agricultural market research firm**

**Q1B_1:** Out of every 100 dogs, how many would typically present with a clinical picture where cancer is one of the top three items in the differential diagnosis? Mean = 13.5 (n=305)

**Q1B_3a:** Of the dogs that go on to receive a diagnostic workup aimed at achieving a presumptive diagnosis of cancer, how many would go on to receive treatment for cancer? Mean = 3.3 (n=305)

**Q1B_3b:** Of the dogs that go on to receive a diagnostic workup aimed at achieving a definitive cancer diagnosis, how many would go on to receive treatment for cancer? Mean = 3.7 (n=305)

## Summary of Survey Findings

13.5% of dogs present to their veterinarian with a clinical picture where cancer is one of the top three items on the differential diagnosis, and 7.0% (3.3% presumptively diagnosed + 3.7% definitively diagnosed) of the total go on to receive treatment for cancer. Thus, 7/13.5 (51.9%) of dogs who present with a clinical picture where cancer is among the top three items on the differential diagnosis go on to receive treatment for cancer.
