# Supplementary material for: Clinical validation of a next-generation sequencing-based multi-cancer early detection “liquid biopsy” blood test in over 1,000 dogs using an independent testing set: The CANcer Detection in Dogs (CANDiD) study
Source: PLoS One. 2022 Apr 26;17(4):e0266623. doi: 10.1371/journal.pone.0266623 (PMC9041869; doi:10.1371/journal.pone.0266623)
Supplement: S1 Table — (PDF) [file pone.0266623.s002.pdf]

S1 Table. Full subject level data for subjects enrolled in the CANDiD study

See legend on last page.

Subject	Excluded	Reason for Exclusion	Training Set	Testing Set	Age	Sex	Spay/ Neuter	Weight (kg)	Purebred or Mixed Breed	Breed (Purebred)	Hemolysis	Lipemia	Cancer Diagnosis at Enrollment	Cancer Diagnosed after Liquid Biopsy	Cancer Type	Immunophenotype (Lymphoid Cancer)	Extent of Disease: Non-Lymphoid Cancer	Extent of Disease: Lymphoid Cancer	Tumor Size	Cancer Type (Cancer #2)	Immunophenotype (Cancer #2 if Lymphoid)	Extent of Disease: Non-Lymphoid Cancer (Cancer #2)	Extent of Disease: Lymphoid Cancer (Cancer #2)	Tumor Size (Cancer #2)	Liquid Biopsy Result	CSO Prediction of Hematological Malignancy
0001	No	NA	No	Yes	3.00	Male	Yes	81.47	Purebred	St. Bernard	3.0	2.0	No	No	NA	NA	NA	NA	NA	NA	NA	NA	NA	NA	Negative	No
0002	No	NA	Yes	No	3.00	Female	Yes	62.53	Purebred	St. Bernard	2.0	2.0	No	No	NA	NA	NA	NA	NA	NA	NA	NA	NA	NA	Negative	No
0003	No	NA	No	Yes	2.42	Male	Yes	25.05	Mixed-breed	NA	0.0	1.0	No	No	NA	NA	NA	NA	NA	NA	NA	NA	NA	NA	Negative	No
0004	Yes	Test failure	No	No	1.75	Female	Yes	20.54	Mixed-breed	NA	0.0	1.0	No	No	NA	NA	NA	NA	NA	NA	NA	NA	NA	NA	Negative	No
0005	Yes	Test failure	No	No	3.00	Male	Yes	42.89	Mixed-breed	NA	0.0	0.0	No	No	NA	NA	NA	NA	NA	NA	NA	NA	NA	NA	Negative	No
0006	No	NA	Yes	No	8.00	Male	Yes	31.87	Mixed-breed	NA	0.5	0.5	No	No	NA	NA	NA	NA	NA	NA	NA	NA	NA	NA	Negative	No
0007	No	NA	No	Yes	7.00	Female	Yes	24.05	Mixed-breed	NA	1.0	1.0	No	No	NA	NA	NA	NA	NA	NA	NA	NA	NA	NA	Negative	No
0008	Yes	Enrollment or Clinical Deviation	No	No	4.00	Male	Yes	38.68	Mixed-breed	NA	0.0	1.0	No	No	NA	NA	NA	NA	NA	NA	NA	NA	NA	NA	Negative	No
0009	No	NA	No	Yes	11.00	Male	Yes	43.18	Mixed-breed	NA	0.0	1.0	No	No	NA	NA	NA	NA	NA	NA	NA	NA	NA	NA	Positive	No
0010	No	NA	No	Yes	1.08	Male	Yes	32.73	Mixed-breed	NA	0.0	0.0	No	No	NA	NA	NA	NA	NA	NA	NA	NA	NA	NA	Negative	No
0011	No	NA	No	Yes	3.00	Male	Yes	11.18	Purebred	Boston Terrier	1.0	1.0	No	No	NA	NA	NA	NA	NA	NA	NA	NA	NA	NA	Negative	No
0012	Yes	Test failure	No	No	1.25	Female	Yes	11.27	Mixed-breed	NA	0.0	0.0	No	No	NA	NA	NA	NA	NA	NA	NA	NA	NA	NA	Negative	No
0013	No	NA	No	Yes	5.58	Male	Yes	34.55	Mixed-breed	NA	0.0	0.0	No	No	NA	NA	NA	NA	NA	NA	NA	NA	NA	NA	Negative	No
0014	No	NA	Yes	No	6.00	Female	Yes	37.00	Purebred	Labrador Retriever	2.0	2.0	No	No	NA	NA	NA	NA	NA	NA	NA	NA	NA	NA	Negative	No
0015	No	NA	Yes	No	6.00	Male	Yes	40.36	Purebred	Labrador Retriever	1.0	1.0	No	No	NA	NA	NA	NA	NA	NA	NA	NA	NA	NA	Negative	No
0016	No	NA	No	Yes	3.67	Female	Yes	24.64	Purebred	Boxer	3.0	NA	No	No	NA	NA	NA	NA	NA	NA	NA	NA	NA	NA	Negative	No
0017	No	NA	No	Yes	1.33	Male	Yes	35.68	Purebred	German Shepherd	0.0	0.0	No	No	NA	NA	NA	NA	NA	NA	NA	NA	NA	NA	Negative	No
0018	No	NA	Yes	No	13.25	Female	Yes	30.45	Purebred	Golden Retriever	0.0	0.0	No	No	NA	NA	NA	NA	NA	NA	NA	NA	NA	NA	Negative	No
0019	No	NA	No	Yes	1.67	Female	Yes	11.59	Mixed-breed	NA	2.0	1.0	No	No	NA	NA	NA	NA	NA	NA	NA	NA	NA	NA	Negative	No
0020	No	NA	No	Yes	2.42	Male	Yes	34.09	Mixed-breed	NA	1.0	1.0	No	No	NA	NA	NA	NA	NA	NA	NA	NA	NA	NA	Negative	No
0021	No	NA	No	Yes	3.67	Female	Yes	29.06	Mixed-breed	NA	1.0	2.0	No	No	NA	NA	NA	NA	NA	NA	NA	NA	NA	NA	Negative	No
0022	No	NA	No	Yes	6.42	Female	Yes	14.43	Mixed-breed	NA	2.5	1.0	No	No	NA	NA	NA	NA	NA	NA	NA	NA	NA	NA	Negative	No
0023	No	NA	No	Yes	12.50	Female	Yes	36.08	Mixed-breed	NA	1.0	1.0	No	No	NA	NA	NA	NA	NA	NA	NA	NA	NA	NA	Negative	No
0024	No	NA	No	Yes	8.17	Female	Yes	30.91	Mixed-breed	NA	3.5	2.0	No	No	NA	NA	NA	NA	NA	NA	NA	NA	NA	NA	Negative	No
0025	No	NA	Yes	No	5.08	Female	Yes	30.00	Mixed-breed	NA	1.5	1.5	No	No	NA	NA	NA	NA	NA	NA	NA	NA	NA	NA	Negative	No
0026	Yes	Test failure	No	No	10.83	Female	Yes	30.91	Purebred	Labrador Retriever	0.0	1.0	No	No	NA	NA	NA	NA	NA	NA	NA	NA	NA	NA	Negative	No
0027	No	NA	No	Yes	3.08	Female	Yes	62.63	Mixed-breed	NA	3.0	1.0	No	No	NA	NA	NA	NA	NA	NA	NA	NA	NA	NA	Negative	No
0028	No	NA	No	Yes	2.00	Female	Yes	34.09	Purebred	German Shepherd	2.0	NA	No	No	NA	NA	NA	NA	NA	NA	NA	NA	NA	NA	Negative	No
0029	No	NA	No	Yes	3.92	Male	Yes	34.09	Purebred	German Shepherd	2.5	1.0	No	No	NA	NA	NA	NA	NA	NA	NA	NA	NA	NA	Negative	No
0030	No	NA	No	Yes	8.83	Male	Yes	25.91	Mixed-breed	NA	1.0	1.0	No	No	NA	NA	NA	NA	NA	NA	NA	NA	NA	NA	Negative	No
0031	No	NA	No	Yes	9.42	Male	Yes	10.45	Purebred	Boston Terrier	3.5	1.0	No	No	NA	NA	NA	NA	NA	NA	NA	NA	NA	NA	Negative	No
0032	No	NA	Yes	No	7.50	Female	Yes	22.73	Mixed-breed	NA	1.5	1.5	No	No	NA	NA	NA	NA	NA	NA	NA	NA	NA	NA	Negative	No
0033	No	NA	Yes	No	12.42	Male	Yes	36.82	Mixed-breed	NA	3.0	1.0	No	No	NA	NA	NA	NA	NA	NA	NA	NA	NA	NA	Negative	No
0034	No	NA	No	Yes	2.00	Male	Yes	34.09	Purebred	German Shepherd	2.0	1.0	No	No	NA	NA	NA	NA	NA	NA	NA	NA	NA	NA	Negative	No
0035	No	NA	Yes	No	6.83	Male	Yes	35.91	Mixed-breed	NA	2.5	1.0	No	No	NA	NA	NA	NA	NA	NA	NA	NA	NA	NA	Negative	No
0036	No	NA	No	Yes	4.42	Male	No	31.09	Purebred	Golden Retriever	4.0	2.0	No	No	NA	NA	NA	NA	NA	NA	NA	NA	NA	NA	Negative	No
0037	No	NA	No	Yes	5.00	Female	Yes	11.23	Purebred	Cocker Spaniel	1.0	1.0	No	No	NA	NA	NA	NA	NA	NA	NA	NA	NA	NA	Negative	No
0038	Yes	Laboratory Workflow Deviation	No	No	11.00	Female	Yes	9.55	Purebred	Pembroke Welsh Corgi	1.0	0.5	No	No	NA	NA	NA	NA	NA	NA	NA	NA	NA	NA	Negative	No
0039	No	NA	No	Yes	2.08	Female	No	38.36	Purebred	Rottweiler	1.0	2.0	No	No	NA	NA	NA	NA	NA	NA	NA	NA	NA	NA	Negative	No
0040	No	NA	No	Yes	5.00	Male	Yes	31.41	Mixed-breed	NA	1.0	1.0	No	No	NA	NA	NA	NA	NA	NA	NA	NA	NA	NA	Negative	No
0041	Yes	Test failure	No	No	4.50	Male	Yes	6.05	Mixed-breed	NA	1.5	1.0	No	No	NA	NA	NA	NA	NA	NA	NA	NA	NA	NA	Negative	No
0042	No	NA	No	Yes	7.17	Male	Yes	11.95	Mixed-breed	NA	0.5	1.0	No	No	NA	NA	NA	NA	NA	NA	NA	NA	NA	NA	Negative	No
0043	No	NA	Yes	No	12.00	Female	Yes	11.68	Mixed-breed	NA	4.0	2.0	No	No	NA	NA	NA	NA	NA	NA	NA	NA	NA	NA	Negative	No
0044	Yes	Enrollment or Clinical Deviation	No	No	10.75	Male	Yes	23.64	Mixed-breed	NA	3.0	1.0	No	No	NA	NA	NA	NA	NA	NA	NA	NA	NA	NA	Negative	No
0045	No	NA	Yes	No	3.33	Female	Yes	34.00	Purebred	Labrador Retriever	2.0	1.0	No	No	NA	NA	NA	NA	NA	NA	NA	NA	NA	NA	Negative	No
0046	No	NA	Yes	No	11.83	Female	Yes	19.55	Purebred	English Springer Spaniel	3.5	2.0	No	No	NA	NA	NA	NA	NA	NA	NA	NA	NA	NA	Negative	No
0047	No	NA	No	Yes	4.83	Female	No	27.73	Purebred	German Shorthaired Pointer	1.0	2.0	No	No	NA	NA	NA	NA	NA	NA	NA	NA	NA	NA	Negative	No
0048	No	NA	No	Yes	1.33	Male	Yes	11.55	Mixed-breed	NA	4.0	2.0	No	No	NA	NA	NA	NA	NA	NA	NA	NA	NA	NA	Negative	No
0049	No	NA	No	Yes	9.42	Female	Yes	12.73	Mixed-breed	NA	4.0	2.0	No	No	NA	NA	NA	NA	NA	NA	NA	NA	NA	NA	Negative	No
0050	No	NA	No	Yes	2.67	Female	Yes	31.32	Mixed-breed	NA	1.0	1.0	No	No	NA	NA	NA	NA	NA	NA	NA	NA	NA	NA	Negative	No
0051	No	NA	No	Yes	4.25	Female	Yes	27.41	Mixed-breed	NA	4.0	2.0	No	No	NA	NA	NA	NA	NA	NA	NA	NA	NA	NA	Negative	No
0052	Yes	Test failure	No	No	4.08	Female	Yes	53.11	Purebred	Cane Corso	NA	1.0	No	No	NA	NA	NA	NA	NA	NA	NA	NA	NA	NA	Negative	No
0053	No	NA	No	Yes	3.67	Male	Yes	30.16	Mixed-breed	NA	3.5	2.0	No	No	NA	NA	NA	NA	NA	NA	NA	NA	NA	NA	Negative	No
0054	No	NA	No	Yes	3.67	Female	Yes	34.07	Mixed-breed	NA	2.5	1.0	No	No	NA	NA	NA	NA	NA	NA	NA	NA	NA	NA	Negative	No
0055	No	NA	No	Yes	2.00	Female	Yes	21.82	Mixed-breed	NA	3.0	2.0	No	No	NA	NA	NA	NA	NA	NA	NA	NA	NA	NA	Negative	No
0056	No	NA	No	Yes	5.50	Male	Yes	15.91	Purebred	Beagle	2.0	1.0	No	No	NA	NA	NA	NA	NA	NA	NA	NA	NA	NA	Negative	No
0057	No	NA	No	Yes	1.92	Male	Yes	44.64	Mixed-breed	NA	1.0	1.0	No	No	NA	NA	NA	NA	NA	NA	NA	NA	NA	NA	Negative	No
0058	No	NA	No	Yes	9.50	Female	Yes	32.64	Purebred	Doberman Pinscher	3.0	1.0	No	No	NA	NA	NA	NA	NA	NA	NA	NA	NA	NA	Negative	No
0059	No	NA	No	Yes	8.25	Female	Yes	22.55	Purebred	Greyhound	4.0	2.0	No	No	NA	NA	NA	NA	NA	NA	NA	NA	NA	NA	Negative	No
0060	No	NA	No	Yes	6.83	Female	Yes	24.65	Mixed-breed	NA	1.0	1.0	No	No	NA	NA	NA	NA	NA	NA	NA	NA	NA	NA	Negative	No
0061	No	NA	No	Yes	5.17	Male	Yes	81.82	Purebred	St. Bernard	2.0	1.0	No	No	NA	NA	NA	NA	NA	NA	NA	NA	NA	NA	Negative	No
0062	Yes	Test failure	No	No	12.00	Male	Yes	26.56	Mixed-breed	NA	3.0	1.0	No	No	NA	NA	NA	NA	NA	NA	NA	NA	NA	NA	Fail	No
0063	No	NA	No	Yes	5.42	Female	Yes	10.72	Purebred	French Bulldog	1.5	2.0	No	No	NA	NA	NA	NA	NA	NA	NA	NA	NA	NA	Negative	No
0064	Yes	Laboratory Workflow Deviation	No	No	3.67	Male	No	33.07	Mixed-breed	NA	4.0	2.0	No	No	NA	NA	NA	NA	NA	NA	NA	NA	NA	NA	Negative	No
0065	No	NA	No	Yes	1.67	Male	Yes	35.45	Purebred	Boxer	3.5	1.0	No	No	NA	NA	NA	NA	NA	NA	NA	NA	NA	NA	Negative	No
0066	No	NA	Yes	No	6.50	Male	Yes	11.12	Mixed-breed	NA	3.5	1.5	No	No	NA	NA	NA	NA	NA	NA	NA	NA	NA	NA	Negative	No
0067	No	NA	Yes	No	1.83	Male	Yes	5.91	Mixed-breed	NA	4.0	2.0	No	No	NA	NA	NA	NA	NA	NA	NA	NA	NA	NA	Negative	No
0068	No	NA	Yes	No	3.67	Male	Yes	29.66	Mixed-breed	NA	2.5	2.0	No	No	NA	NA	NA	NA	NA	NA	NA	NA	NA	NA	Negative	No
0069	No	NA	No	Yes	5.17	Female	Yes	20.04	Purebred	Bulldog, American	4.0	2.0	No	No	NA	NA	NA	NA	NA	NA	NA	NA	NA	NA	Negative	No
0070	No	NA	No	Yes	9.75	Female	Yes	34.09	Mixed-breed	NA	1.5	1.0	No	No	NA	NA	NA	NA	NA	NA	NA	NA	NA	NA	Negative	No
0071	No	NA	No	Yes	4.50	Female	Yes	31.57	Mixed-breed	NA	2.0	2.0	No	No	NA	NA	NA	NA	NA	NA	NA	NA	NA	NA	Negative	No
0072	No	NA	No	Yes	2.00	Male	Yes	30.06	Mixed-breed	NA	3.0	2.0	No	No	NA	NA	NA	NA	NA	NA	NA	NA	NA	NA	Negative	No

S1 Table. Full subject level data for subjects enrolled in the CANDiD study (continued)

See legend on last page.

Subject	Excluded	Reason for Exclusion	Training Set	Testing Set	Age	Sex	Spay/ Neuter	Weight (kg)	Purebred or Mixed Breed	Breed (Purebred)	Hemolysis	Lipemia	Cancer Diagnosis at Enrollment	Cancer Diagnosed after Liquid Biopsy	Cancer Type	Immunophenotype (Lymphoid Cancer)	Extent of Disease: Non-Lymphoid Cancer	Extent of Disease: Lymphoid Cancer	Tumor Size	Cancer Type (Cancer #2)	Immunophenotype (Cancer #2 if Lymphoid)	Extent of Disease: Non-Lymphoid Cancer (Cancer #2)	Extent of Disease: Lymphoid Cancer (Cancer #2)	Tumor Size (Cancer #2)	Liquid Biopsy Result	CSO Prediction of Hematological Malignancy
0073	Yes	Laboratory Workflow Deviation	No	No	NA	NA	NA	NA	NA	NA	4.0	2.0	No	No	NA	NA	NA	NA	NA	NA	NA	NA	NA	NA	Negative	No
0074	No	NA	No	Yes	6.75	Male	Yes	34.50	Purebred	Golden Retriever	0.0	0.0	No	No	NA	NA	NA	NA	NA	NA	NA	NA	NA	NA	Negative	No
0075	No	NA	Yes	No	7.00	Female	Yes	20.59	Purebred	Siberian Husky	3.5	1.0	No	No	NA	NA	NA	NA	NA	NA	NA	NA	NA	NA	Negative	No
0076	Yes	Enrollment or Clinical Deviation	No	No	10.67	Male	Yes	22.82	Mixed-breed	NA	1.0	1.0	No	No	NA	NA	NA	NA	NA	NA	NA	NA	NA	NA	Negative	No
0077	No	NA	No	Yes	3.67	Female	Yes	21.00	Mixed-breed	NA	2.0	2.0	No	No	NA	NA	NA	NA	NA	NA	NA	NA	NA	NA	Negative	No
0078	No	NA	No	Yes	1.92	Male	Yes	50.00	Purebred	Rottweiler	3.0	NA	No	No	NA	NA	NA	NA	NA	NA	NA	NA	NA	NA	Negative	No
0079	No	NA	No	Yes	2.42	Male	Yes	28.45	Mixed-breed	NA	0.0	NA	No	No	NA	NA	NA	NA	NA	NA	NA	NA	NA	NA	Negative	No
0080	No	NA	No	Yes	5.33	Male	Yes	33.64	Purebred	Golden Retriever	2.0	0.0	No	No	NA	NA	NA	NA	NA	NA	NA	NA	NA	NA	Negative	No
0081	No	NA	Yes	No	8.42	Female	Yes	45.09	Purebred	German Shepherd	4.0	2.0	No	No	NA	NA	NA	NA	NA	NA	NA	NA	NA	NA	Negative	No
0082	No	NA	No	Yes	5.00	Female	Yes	17.24	Mixed-breed	NA	3.0	2.0	No	No	NA	NA	NA	NA	NA	NA	NA	NA	NA	NA	Negative	No
0083	No	NA	No	Yes	4.17	Male	Yes	27.27	Purebred	Brittany	1.0	1.0	No	No	NA	NA	NA	NA	NA	NA	NA	NA	NA	NA	Negative	No
0084	No	NA	Yes	No	1.42	Female	Yes	33.18	Purebred	Labrador Retriever	2.5	2.0	No	No	NA	NA	NA	NA	NA	NA	NA	NA	NA	NA	Negative	No
0085	Yes	Test failure	No	No	9.75	Male	Yes	30.26	Mixed-breed	NA	1.0	1.0	No	No	NA	NA	NA	NA	NA	NA	NA	NA	NA	NA	Fail	No
0086	No	NA	No	Yes	3.50	Male	Yes	25.75	Mixed-breed	NA	NA	1.5	No	No	NA	NA	NA	NA	NA	NA	NA	NA	NA	NA	Negative	No
0087	No	NA	No	Yes	2.50	Male	Yes	63.64	Purebred	Great Dane	2.0	1.0	No	No	NA	NA	NA	NA	NA	NA	NA	NA	NA	NA	Negative	No
0088	No	NA	Yes	No	11.50	Female	Yes	16.63	Purebred	Pembroke Welsh Corgi	3.5	2.0	No	No	NA	NA	NA	NA	NA	NA	NA	NA	NA	NA	Negative	No
0089	No	NA	No	Yes	8.00	Male	Yes	14.63	Purebred	Cardigan Welsh Corgi	3.5	2.0	No	No	NA	NA	NA	NA	NA	NA	NA	NA	NA	NA	Negative	No
0090	No	NA	No	Yes	5.00	Female	Yes	33.87	Purebred	Bouviers des Flandres	0.5	1.0	No	No	NA	NA	NA	NA	NA	NA	NA	NA	NA	NA	Negative	No
0091	No	NA	No	Yes	1.67	Female	Yes	38.78	Mixed-breed	NA	2.0	1.0	No	No	NA	NA	NA	NA	NA	NA	NA	NA	NA	NA	Negative	No
0092	No	NA	No	Yes	5.00	Female	Yes	20.84	Mixed-breed	NA	3.0	1.0	No	No	NA	NA	NA	NA	NA	NA	NA	NA	NA	NA	Negative	No
0093	No	NA	Yes	No	6.00	Female	Yes	27.76	Mixed-breed	NA	2.0	1.0	No	No	NA	NA	NA	NA	NA	NA	NA	NA	NA	NA	Negative	No
0094	No	NA	No	Yes	5.42	Male	Yes	22.91	Mixed-breed	NA	4.0	2.0	No	No	NA	NA	NA	NA	NA	NA	NA	NA	NA	NA	Negative	No
0095	No	NA	No	Yes	4.33	Female	No	27.27	Mixed-breed	NA	3.5	2.0	No	No	NA	NA	NA	NA	NA	NA	NA	NA	NA	NA	Negative	No
0096	No	NA	No	Yes	2.08	Female	No	26.82	Mixed-breed	NA	4.0	1.0	No	No	NA	NA	NA	NA	NA	NA	NA	NA	NA	NA	Negative	No
0097	No	NA	No	Yes	2.33	Female	No	38.64	Purebred	Bernese Mountain Dogs	4.0	2.0	No	No	NA	NA	NA	NA	NA	NA	NA	NA	NA	NA	Negative	No
0098	No	NA	No	Yes	3.50	Male	No	32.00	Mixed-breed	NA	3.5	2.0	No	No	NA	NA	NA	NA	NA	NA	NA	NA	NA	NA	Negative	No
0099	No	NA	Yes	No	11.08	Female	Yes	40.09	Purebred	German Shepherd	2.5	1.0	No	No	NA	NA	NA	NA	NA	NA	NA	NA	NA	NA	Negative	No
0100	No	NA	No	Yes	1.33	Male	No	38.55	Purebred	Labrador Retriever	3.5	1.5	No	No	NA	NA	NA	NA	NA	NA	NA	NA	NA	NA	Negative	No
0101	No	NA	No	Yes	2.67	Female	No	27.82	Purebred	Labrador Retriever	3.0	1.0	No	No	NA	NA	NA	NA	NA	NA	NA	NA	NA	NA	Negative	No
0102	No	NA	No	Yes	2.08	Female	No	33.64	Purebred	Golden Retriever	1.0	1.0	No	No	NA	NA	NA	NA	NA	NA	NA	NA	NA	NA	Negative	No
0103	No	NA	No	Yes	4.00	Female	Yes	23.25	Mixed-breed	NA	3.5	1.0	No	No	NA	NA	NA	NA	NA	NA	NA	NA	NA	NA	Negative	No
0104	No	NA	No	Yes	11.00	Male	Yes	24.55	Mixed-breed	NA	2.0	1.0	No	No	NA	NA	NA	NA	NA	NA	NA	NA	NA	NA	Negative	No
0105	No	NA	No	Yes	8.00	Male	Yes	33.67	Mixed-breed	NA	1.0	1.0	No	No	NA	NA	NA	NA	NA	NA	NA	NA	NA	NA	Negative	No
0106	Yes	Test failure	No	No	3.00	Female	No	20.45	Purebred	Siberian Husky	3.5	1.5	No	No	NA	NA	NA	NA	NA	NA	NA	NA	NA	NA	Positive	No
0107	No	NA	No	Yes	7.17	Female	Yes	25.91	Mixed-breed	NA	2.5	2.0	No	No	NA	NA	NA	NA	NA	NA	NA	NA	NA	NA	Negative	No
0108	No	NA	No	Yes	11.00	Female	Yes	10.59	Purebred	Cocker Spaniel	2.5	NA	No	No	NA	NA	NA	NA	NA	NA	NA	NA	NA	NA	Negative	No
0109	No	NA	No	Yes	10.50	Female	Yes	13.86	Purebred	Pembroke Welsh Corgi	4.0	2.0	No	No	NA	NA	NA	NA	NA	NA	NA	NA	NA	NA	Negative	No
0110	No	NA	Yes	No	9.42	Female	Yes	33.64	Purebred	Boxer	3.0	1.0	No	No	NA	NA	NA	NA	NA	NA	NA	NA	NA	NA	Negative	No
0111	No	NA	No	Yes	9.50	Male	Yes	38.45	Purebred	Golden Retriever	3.5	2.0	No	No	NA	NA	NA	NA	NA	NA	NA	NA	NA	NA	Negative	No
0112	No	NA	No	Yes	2.67	Male	No	25.45	Purebred	Poodle, Standard	4.0	2.0	No	No	NA	NA	NA	NA	NA	NA	NA	NA	NA	NA	Negative	No
0113	Yes	Laboratory Workflow Deviation	No	No	2.17	Female	Yes	26.64	Mixed-breed	NA	4.0	2.0	No	No	NA	NA	NA	NA	NA	NA	NA	NA	NA	NA	Negative	No
0114	No	NA	Yes	No	5.00	Female	No	29.55	Mixed-breed	NA	4.0	1.0	No	No	NA	NA	NA	NA	NA	NA	NA	NA	NA	NA	Negative	No
0115	Yes	Test failure	No	No	6.83	Male	Yes	60.45	Purebred	Newfoundland	NA	2.0	No	No	NA	NA	NA	NA	NA	NA	NA	NA	NA	NA	Negative	No
0116	No	NA	No	Yes	8.17	Female	Yes	34.55	Mixed-breed	NA	3.5	2.0	No	No	NA	NA	NA	NA	NA	NA	NA	NA	NA	NA	Negative	No
0117	Yes	Test failure	No	No	3.42	Male	Yes	71.82	Purebred	Mastiff	1.0	1.0	No	No	NA	NA	NA	NA	NA	NA	NA	NA	NA	NA	Fail	No
0118	Yes	Test failure	No	No	10.00	Female	Yes	32.23	Purebred	Labrador Retriever	2.0	2.0	No	No	NA	NA	NA	NA	NA	NA	NA	NA	NA	NA	Fail	No
0119	No	NA	No	Yes	4.17	Female	Yes	19.86	Purebred	Boxer	2.5	2.0	No	No	NA	NA	NA	NA	NA	NA	NA	NA	NA	NA	Negative	No
0120	No	NA	No	Yes	6.08	Female	Yes	23.36	Mixed-breed	NA	1.5	1.5	No	No	NA	NA	NA	NA	NA	NA	NA	NA	NA	NA	Negative	No
0121	No	NA	No	Yes	2.33	Male	Yes	11.36	Purebred	Boston Terrier	4.0	2.0	No	No	NA	NA	NA	NA	NA	NA	NA	NA	NA	NA	Negative	No
0122	Yes	Enrollment or Clinical Deviation	No	No	10.42	Male	Yes	15.91	Purebred	Boston Terrier	4.0	1.5	No	No	NA	NA	NA	NA	NA	NA	NA	NA	NA	NA	Negative	No
0123	No	NA	No	Yes	2.17	Male	Yes	14.41	Purebred	French Bulldog	1.5	2.0	No	No	NA	NA	NA	NA	NA	NA	NA	NA	NA	NA	Negative	No
0124	No	NA	Yes	No	6.00	Male	Yes	38.18	Mixed-breed	NA	2.0	2.0	No	No	NA	NA	NA	NA	NA	NA	NA	NA	NA	NA	Negative	No
0125	No	NA	No	Yes	2.00	Female	Yes	25.59	Mixed-breed	NA	1.0	2.0	No	No	NA	NA	NA	NA	NA	NA	NA	NA	NA	NA	Negative	No
0126	No	NA	No	Yes	3.00	Male	Yes	31.82	Mixed-breed	NA	3.0	2.0	No	No	NA	NA	NA	NA	NA	NA	NA	NA	NA	NA	Negative	No
0127	No	NA	Yes	No	2.00	Female	Yes	24.55	Purebred	English Springer Spaniel	3.0	1.0	No	No	NA	NA	NA	NA	NA	NA	NA	NA	NA	NA	Negative	No
0128	No	NA	No	Yes	3.00	Female	No	28.45	Purebred	Labrador Retriever	3.0	1.0	No	No	NA	NA	NA	NA	NA	NA	NA	NA	NA	NA	Negative	No
0129	No	NA	No	Yes	2.08	Female	No	27.91	Purebred	Golden Retriever	2.0	1.0	No	No	NA	NA	NA	NA	NA	NA	NA	NA	NA	NA	Negative	No
0130	No	NA	No	Yes	2.50	Female	No	36.82	Purebred	Bernese Mountain Dogs	2.0	2.0	No	No	NA	NA	NA	NA	NA	NA	NA	NA	NA	NA	Negative	No
0131	No	NA	No	Yes	4.00	Male	No	27.27	Purebred	Siberian Husky	4.0	2.0	No	No	NA	NA	NA	NA	NA	NA	NA	NA	NA	NA	Negative	No
0132	No	NA	No	Yes	4.17	Female	Yes	33.32	Purebred	German Shepherd	3.0	1.0	No	No	NA	NA	NA	NA	NA	NA	NA	NA	NA	NA	Negative	No
0133	Yes	Enrollment or Clinical Deviation	No	No	10.75	Female	Yes	27.23	Purebred	Greyhound	3.0	1.0	No	No	NA	NA	NA	NA	NA	NA	NA	NA	NA	NA	Positive	No
0134	Yes	Laboratory Workflow Deviation	No	No	10.58	Female	Yes	18.09	Purebred	English Springer Spaniel	4.0	2.0	No	No	NA	NA	NA	NA	NA	NA	NA	NA	NA	NA	Negative	No
0135	Yes	Laboratory Workflow Deviation	No	No	11.08	Male	Yes	10.09	Mixed-breed	NA	2.0	2.0	No	No	NA	NA	NA	NA	NA	NA	NA	NA	NA	NA	Negative	No
0136	No	NA	No	Yes	11.00	Female	Yes	22.64	Mixed-breed	NA	3.0	2.0	No	No	NA	NA	NA	NA	NA	NA	NA	NA	NA	NA	Negative	No
0137	Yes	Test failure	No	No	3.00	Male	Yes	28.64	Mixed-breed	NA	NA	1.5	No	No	NA	NA	NA	NA	NA	NA	NA	NA	NA	NA	Negative	No
0138	No	NA	No	Yes	7.50	Male	Yes	24.09	Purebred	Vizslas	4.0	NA	No	No	NA	NA	NA	NA	NA	NA	NA	NA	NA	NA	Negative	No
0139	No	NA	Yes	No	6.00	Female	Yes	17.64	Purebred	Border Collie	1.0	1.0	No	No	NA	NA	NA	NA	NA	NA	NA	NA	NA	NA	Negative	No
0140	Yes	Test failure	No	No	3.17	Male	Yes	83.18	Purebred	Mastiff	NA	1.0	No	No	NA	NA	NA	NA	NA	NA	NA	NA	NA	NA	Negative	No
0141	No	NA	No	Yes	5.25	Male	Yes	10.36	Purebred	Cavalier King Charles Spaniel	2.0	NA	No	No	NA	NA	NA	NA	NA	NA	NA	NA	NA	NA	Negative	No

S1 Table. Full subject level data for subjects enrolled in the CANDiD study (continued)

See legend on last page.

Subject	Excluded	Reason for Exclusion	Training Set	Testing Set	Age	Sex	Spay/ Neuter	Weight (kg)	Purebred or Mixed Breed	Breed (Purebred)	Hemolysis	Lipemia	Cancer Diagnosis at Enrollment	Cancer Diagnosed after Liquid Biopsy	Cancer Type	Immunophenotype (Lymphoid Cancer)	Extent of Disease: Non-Lymphoid Cancer	Extent of Disease: Lymphoid Cancer	Tumor Size	Cancer Type (Cancer #2)	Immunophenotype (Cancer #2 if Lymphoid)	Extent of Disease: Non-Lymphoid Cancer (Cancer #2)	Extent of Disease: Lymphoid Cancer (Cancer #2)	Tumor Size (Cancer #2)	Liquid Biopsy Result	CSO Prediction of Hematological Malignancy
0142	Yes	Test failure	No	No	4.33	Male	Yes	54.55	Purebred	Newfoundland	NA	2.0	No	No	NA	NA	NA	NA	NA	NA	NA	NA	NA	NA	Negative	No
0143	No	NA	No	Yes	2.33	Male	Yes	40.91	Purebred	Labrador Retriever	1.0	1.0	No	No	NA	NA	NA	NA	NA	NA	NA	NA	NA	NA	Negative	No
0144	No	NA	No	Yes	6.08	Female	Yes	26.59	Mixed-breed	NA	4.0	2.0	No	No	NA	NA	NA	NA	NA	NA	NA	NA	NA	NA	Negative	No
0145	No	NA	No	Yes	6.58	Male	Yes	31.05	Mixed-breed	NA	3.0	1.0	No	No	NA	NA	NA	NA	NA	NA	NA	NA	NA	NA	Negative	No
0146	No	NA	Yes	No	6.08	Female	Yes	26.36	Mixed-breed	NA	3.0	2.0	No	No	NA	NA	NA	NA	NA	NA	NA	NA	NA	NA	Negative	No
0147	No	NA	No	Yes	3.17	Female	Yes	26.82	Purebred	Boxer	2.5	2.0	No	No	NA	NA	NA	NA	NA	NA	NA	NA	NA	NA	Negative	No
0148	No	NA	No	Yes	8.67	Male	Yes	40.36	Purebred	Collie	3.5	2.0	No	Yes	NA	NA	NA	NA	NA	NA	NA	NA	NA	NA	Positive	Yes
0149	No	NA	No	Yes	1.42	Female	Yes	54.55	Purebred	St. Bernard	1.0	1.0	No	No	NA	NA	NA	NA	NA	NA	NA	NA	NA	NA	Negative	No
0150	No	NA	No	Yes	7.58	Male	No	42.73	Purebred	Labrador Retriever	3.5	0.0	No	No	NA	NA	NA	NA	NA	NA	NA	NA	NA	NA	Negative	No
0151	No	NA	Yes	No	5.58	Female	No	35.45	Purebred	Labrador Retriever	3.0	2.0	No	No	NA	NA	NA	NA	NA	NA	NA	NA	NA	NA	Negative	No
0152	No	NA	No	Yes	4.42	Female	Yes	38.18	Mixed-breed	NA	0.5	1.0	No	No	NA	NA	NA	NA	NA	NA	NA	NA	NA	NA	Negative	No
0153	No	NA	No	Yes	3.17	Male	No	27.73	Purebred	German Shorthaired Pointer	1.5	0.5	No	No	NA	NA	NA	NA	NA	NA	NA	NA	NA	NA	Positive	No
0154	No	NA	No	Yes	3.00	Female	Yes	25.00	Mixed-breed	NA	4.0	2.0	No	No	NA	NA	NA	NA	NA	NA	NA	NA	NA	NA	Negative	No
0155	No	NA	Yes	No	10.00	Female	Yes	21.36	Mixed-breed	NA	3.0	2.0	No	No	NA	NA	NA	NA	NA	NA	NA	NA	NA	NA	Negative	No
0156	Yes	Laboratory Workflow Deviation	No	No	6.50	Female	Yes	29.55	Purebred	American Staffordshire Terrier	4.0	2.0	No	No	NA	NA	NA	NA	NA	NA	NA	NA	NA	NA	Negative	No
0157	No	NA	No	Yes	5.67	Male	No	26.14	Purebred	Samoyed	2.5	1.0	No	No	NA	NA	NA	NA	NA	NA	NA	NA	NA	NA	Negative	No
0158	No	NA	No	Yes	6.08	Female	Yes	27.73	Purebred	Boxer	4.0	2.0	No	No	NA	NA	NA	NA	NA	NA	NA	NA	NA	NA	Negative	No
0159	No	NA	No	Yes	2.50	Female	Yes	26.82	Purebred	Siberian Husky	3.5	1.0	No	No	NA	NA	NA	NA	NA	NA	NA	NA	NA	NA	Negative	No
0160	No	NA	No	Yes	9.00	Male	Yes	30.91	Mixed-breed	NA	4.0	2.0	No	No	NA	NA	NA	NA	NA	NA	NA	NA	NA	NA	Negative	No
0161	No	NA	No	Yes	8.50	Male	Yes	77.27	Purebred	Mastiff	2.0	1.0	No	No	NA	NA	NA	NA	NA	NA	NA	NA	NA	NA	Negative	No
0162	No	NA	No	Yes	4.50	Female	Yes	15.23	Mixed-breed	NA	4.0	1.0	No	No	NA	NA	NA	NA	NA	NA	NA	NA	NA	NA	Negative	No
0163	No	NA	No	Yes	9.00	Female	Yes	19.73	Mixed-breed	NA	3.5	1.0	No	No	NA	NA	NA	NA	NA	NA	NA	NA	NA	NA	Negative	No
0164	Yes	Laboratory Workflow Deviation	No	No	3.17	Male	Yes	24.09	Mixed-breed	NA	4.0	2.0	No	No	NA	NA	NA	NA	NA	NA	NA	NA	NA	NA	Negative	No
0165	No	NA	Yes	No	2.42	Female	No	36.82	Purebred	Labrador Retriever	3.5	1.5	No	No	NA	NA	NA	NA	NA	NA	NA	NA	NA	NA	Negative	No
0166	No	NA	No	Yes	3.25	Male	No	32.73	Purebred	Labrador Retriever	1.5	1.0	No	No	NA	NA	NA	NA	NA	NA	NA	NA	NA	NA	Negative	No
0167	No	NA	No	Yes	11.50	Male	Yes	29.27	Purebred	Collie	4.0	1.0	No	No	NA	NA	NA	NA	NA	NA	NA	NA	NA	NA	Negative	No
0168	No	NA	No	Yes	3.67	Male	Yes	44.55	Mixed-breed	NA	1.5	1.0	No	No	NA	NA	NA	NA	NA	NA	NA	NA	NA	NA	Negative	No
0169	No	NA	No	Yes	9.00	Male	No	41.36	Purebred	Labrador Retriever	2.5	1.0	No	No	NA	NA	NA	NA	NA	NA	NA	NA	NA	NA	Negative	No
0170	No	NA	No	Yes	3.83	Female	No	37.27	Purebred	Labrador Retriever	4.0	1.0	No	No	NA	NA	NA	NA	NA	NA	NA	NA	NA	NA	Negative	No
0171	No	NA	No	Yes	4.00	Female	No	35.00	Purebred	Labrador Retriever	4.0	2.0	No	No	NA	NA	NA	NA	NA	NA	NA	NA	NA	NA	Negative	No
0172	Yes	Enrollment or Clinical Deviation	No	No	12.33	Male	Yes	10.68	Purebred	Parson Russell Terrier	3.5	2.0	No	No	NA	NA	NA	NA	NA	NA	NA	NA	NA	NA	Negative	No
0173	No	NA	Yes	No	5.17	Male	Yes	52.73	Purebred	Great Dane	4.0	2.0	No	No	NA	NA	NA	NA	NA	NA	NA	NA	NA	NA	Negative	No
0174	No	NA	No	Yes	8.50	Male	Yes	32.27	Purebred	Bulldog, American	2.0	1.0	No	No	NA	NA	NA	NA	NA	NA	NA	NA	NA	NA	Negative	No
0175	No	NA	No	Yes	2.50	Female	Yes	24.32	Mixed-breed	NA	1.0	1.0	No	No	NA	NA	NA	NA	NA	NA	NA	NA	NA	NA	Negative	No
0176	No	NA	No	Yes	12.00	Female	Yes	40.91	Mixed-breed	NA	2.0	2.0	No	No	NA	NA	NA	NA	NA	NA	NA	NA	NA	NA	Positive	No
0177	No	NA	No	Yes	8.00	Female	Yes	28.18	Mixed-breed	NA	2.0	2.0	No	No	NA	NA	NA	NA	NA	NA	NA	NA	NA	NA	Negative	No
0178	Yes	Test failure	No	No	7.33	Female	Yes	25.91	Mixed-breed	NA	4.0	2.0	No	No	NA	NA	NA	NA	NA	NA	NA	NA	NA	NA	Fail	No
0179	No	NA	No	Yes	8.08	Male	Yes	33.18	Purebred	Golden Retriever	2.5	2.0	No	No	NA	NA	NA	NA	NA	NA	NA	NA	NA	NA	Negative	No
0180	No	NA	No	Yes	3.17	Male	No	45.45	Purebred	Great Pyrenees	3.0	1.0	No	No	NA	NA	NA	NA	NA	NA	NA	NA	NA	NA	Negative	No
0181	No	NA	No	Yes	12.08	Female	Yes	25.82	Mixed-breed	NA	3.0	2.0	No	No	NA	NA	NA	NA	NA	NA	NA	NA	NA	NA	Negative	No
0182	No	NA	No	Yes	7.50	Female	Yes	31.05	Purebred	Golden Retriever	2.0	2.0	No	No	NA	NA	NA	NA	NA	NA	NA	NA	NA	NA	Negative	No
0183	No	NA	No	Yes	12.00	Male	Yes	29.91	Mixed-breed	NA	0.0	1.0	No	No	NA	NA	NA	NA	NA	NA	NA	NA	NA	NA	Negative	No
0184	No	NA	No	Yes	1.42	Male	No	33.64	Mixed-breed	NA	1.0	1.0	No	No	NA	NA	NA	NA	NA	NA	NA	NA	NA	NA	Negative	No
0185	Yes	Test failure	No	No	8.42	Male	No	16.68	Purebred	Soft Coated Wheaten Terrier	2.5	1.0	No	No	NA	NA	NA	NA	NA	NA	NA	NA	NA	NA	Fail	No
0186	No	NA	No	Yes	8.00	Female	Yes	20.91	Purebred	Siberian Husky	3.0	2.0	No	No	NA	NA	NA	NA	NA	NA	NA	NA	NA	NA	Negative	No
0187	No	NA	No	Yes	5.00	Male	Yes	31.55	Mixed-breed	NA	4.0	2.0	No	No	NA	NA	NA	NA	NA	NA	NA	NA	NA	NA	Negative	No
0188	No	NA	No	Yes	7.33	Female	Yes	26.14	Mixed-breed	NA	4.0	1.0	No	No	NA	NA	NA	NA	NA	NA	NA	NA	NA	NA	Negative	No
0189	No	NA	No	Yes	5.00	Male	Yes	26.73	Mixed-breed	NA	3.5	1.0	No	No	NA	NA	NA	NA	NA	NA	NA	NA	NA	NA	Negative	No
0190	No	NA	Yes	No	11.50	Female	Yes	22.05	Mixed-breed	NA	1.5	2.0	No	No	NA	NA	NA	NA	NA	NA	NA	NA	NA	NA	Negative	No
0191	No	NA	No	Yes	3.42	Female	Yes	32.73	Purebred	Golden Retriever	1.0	0.0	No	No	NA	NA	NA	NA	NA	NA	NA	NA	NA	NA	Negative	No
0192	No	NA	No	Yes	1.67	Female	Yes	24.55	Purebred	Labrador Retriever	1.0	1.0	No	No	NA	NA	NA	NA	NA	NA	NA	NA	NA	NA	Negative	No
0193	No	NA	No	Yes	1.50	Male	Yes	11.55	Purebred	Shetland Sheepdog	4.0	2.0	No	No	NA	NA	NA	NA	NA	NA	NA	NA	NA	NA	Negative	No
0194	Yes	Enrollment or Clinical Deviation	No	No	9.00	Female	Yes	25.18	Mixed-breed	NA	4.0	2.0	No	No	NA	NA	NA	NA	NA	NA	NA	NA	NA	NA	Positive	Yes
0195	No	NA	No	Yes	5.00	Female	Yes	11.64	Mixed-breed	NA	4.0	1.0	No	No	NA	NA	NA	NA	NA	NA	NA	NA	NA	NA	Negative	No
0196	Yes	Test failure	No	No	7.00	Female	Yes	18.82	Purebred	Border Collie	2.0	1.0	No	No	NA	NA	NA	NA	NA	NA	NA	NA	NA	NA	Negative	No
0197	No	NA	No	Yes	2.33	Male	Yes	17.91	Purebred	Soft Coated Wheaten Terrier	0.0	NA	No	No	NA	NA	NA	NA	NA	NA	NA	NA	NA	NA	Negative	No
0198	No	NA	Yes	No	2.92	Male	Yes	60.00	Purebred	Rottweiler	0.5	1.0	No	No	NA	NA	NA	NA	NA	NA	NA	NA	NA	NA	Negative	No
0199	No	NA	No	Yes	8.33	Female	Yes	21.73	Mixed-breed	NA	4.0	NA	No	No	NA	NA	NA	NA	NA	NA	NA	NA	NA	NA	Negative	No
0200	No	NA	No	Yes	8.17	Male	Yes	30.91	Mixed-breed	NA	3.5	2.0	No	No	NA	NA	NA	NA	NA	NA	NA	NA	NA	NA	Negative	No
0201	No	NA	Yes	No	11.00	Female	Yes	29.55	Mixed-breed	NA	3.0	2.0	No	No	NA	NA	NA	NA	NA	NA	NA	NA	NA	NA	Negative	No
0202	No	NA	No	Yes	3.67	Male	Yes	33.32	Mixed-breed	NA	0.0	1.0	No	No	NA	NA	NA	NA	NA	NA	NA	NA	NA	NA	Negative	No
0204	No	NA	No	Yes	11.33	Male	Yes	13.55	Mixed-breed	NA	3.0	1.0	No	No	NA	NA	NA	NA	NA	NA	NA	NA	NA	NA	Negative	No
0205	No	NA	Yes	No	12.17	Male	Yes	17.27	Mixed-breed	NA	1.0	2.0	No	No	NA	NA	NA	NA	NA	NA	NA	NA	NA	NA	Negative	No
0206	Yes	Enrollment or Clinical Deviation	No	No	3.83	Male	Yes	14.09	Mixed-breed	NA	2.0	2.0	No	No	NA	NA	NA	NA	NA	NA	NA	NA	NA	NA	Negative	No
0207	Yes	Laboratory Workflow Deviation	No	No	11.08	Female	Yes	32.18	Mixed-breed	NA	2.0	2.0	No	No	NA	NA	NA	NA	NA	NA	NA	NA	NA	NA	Negative	No
0208	Yes	Laboratory Workflow Deviation	No	No	7.50	Male	Yes	36.82	Mixed-breed	NA	2.0	1.0	No	No	NA	NA	NA	NA	NA	NA	NA	NA	NA	NA	Fail	No
0209	No	NA	No	Yes	10.92	Male	Yes	38.45	Purebred	Labrador Retriever	2.0	1.0	No	No	NA	NA	NA	NA	NA	NA	NA	NA	NA	NA	Negative	No

S1 Table. Full subject level data for subjects enrolled in the CANDiD study (continued)

See legend on last page.

Subject	Excluded	Reason for Exclusion	Training Set	Testing Set	Age	Sex	Spay/ Neuter	Weight (kg)	Purebred or Mixed Breed	Breed (Purebred)	Hemolysis	Lipemia	Cancer Diagnosis at Enrollment	Cancer Diagnosed after Liquid Biopsy	Cancer Type	Immunophenotype (Lymphoid Cancer)	Extent of Disease: Non-Lymphoid Cancer	Extent of Disease: Lymphoid Cancer	Tumor Size	Cancer Type (Cancer #2)	Immunophenotype (Cancer #2 if Lymphoid)	Extent of Disease: Non-Lymphoid Cancer (Cancer #2)	Extent of Disease: Lymphoid Cancer (Cancer #2)	Tumor Size (Cancer #2)	Liquid Biopsy Result	CSO Prediction of Hematological Malignancy
0210	No	NA	Yes	No	6.33	Female	Yes	21.91	Mixed-breed	NA	2.0	1.0	No	No	NA	NA	NA	NA	NA	NA	NA	NA	NA	NA	Negative	No
0211	No	NA	No	Yes	5.00	Female	No	21.82	Purebred	Siberian Husky	2.5	2.0	No	No	NA	NA	NA	NA	NA	NA	NA	NA	NA	NA	Negative	No
0212	No	NA	Yes	No	10.33	Female	Yes	19.09	Purebred	Dalmation	1.0	2.0	No	No	NA	NA	NA	NA	NA	NA	NA	NA	NA	NA	Negative	No
0213	Yes	Test failure	No	No	1.33	Male	Yes	29.77	Mixed-breed	NA	2.0	2.0	No	No	NA	NA	NA	NA	NA	NA	NA	NA	NA	NA	Fail	No
0214	No	NA	No	Yes	10.00	Female	Yes	35.18	Mixed-breed	NA	3.0	2.0	No	No	NA	NA	NA	NA	NA	NA	NA	NA	NA	NA	Negative	No
0215	No	NA	Yes	No	7.00	Male	Yes	45.00	Mixed-breed	NA	1.5	1.5	No	No	NA	NA	NA	NA	NA	NA	NA	NA	NA	NA	Negative	No
0216	Yes	Test failure	No	No	6.33	Male	Yes	21.18	Mixed-breed	NA	3.0	1.5	No	No	NA	NA	NA	NA	NA	NA	NA	NA	NA	NA	Negative	No
0217	No	NA	No	Yes	12.42	Female	Yes	29.77	Mixed-breed	NA	3.0	1.0	No	No	NA	NA	NA	NA	NA	NA	NA	NA	NA	NA	Negative	No
0218	No	NA	Yes	No	8.33	Male	Yes	12.86	Purebred	French Bulldog	1.0	1.0	No	No	NA	NA	NA	NA	NA	NA	NA	NA	NA	NA	Negative	No
0219	No	NA	No	Yes	10.92	Female	Yes	25.95	Purebred	Labrador Retriever	2.5	1.0	No	No	NA	NA	NA	NA	NA	NA	NA	NA	NA	NA	Negative	No
0220	No	NA	No	Yes	1.67	Female	Yes	28.77	Purebred	Labrador Retriever	0.0	1.0	No	No	NA	NA	NA	NA	NA	NA	NA	NA	NA	NA	Negative	No
0221	No	NA	Yes	No	9.00	Female	Yes	23.73	Mixed-breed	NA	1.0	1.0	No	No	NA	NA	NA	NA	NA	NA	NA	NA	NA	NA	Negative	No
0222	No	NA	No	Yes	8.00	Female	Yes	27.14	Mixed-breed	NA	3.0	1.0	No	No	NA	NA	NA	NA	NA	NA	NA	NA	NA	NA	Negative	No
0223	No	NA	No	Yes	11.00	Male	Yes	10.27	Purebred	Cairn Terrier	3.0	2.0	No	No	NA	NA	NA	NA	NA	NA	NA	NA	NA	NA	Negative	No
0224	No	NA	No	Yes	7.17	Female	Yes	28.18	Purebred	Golden Retriever	2.0	1.0	No	No	NA	NA	NA	NA	NA	NA	NA	NA	NA	NA	Negative	No
0225	No	NA	No	Yes	8.75	Female	Yes	20.55	Purebred	Soft Coated Wheaten Terrier	2.5	1.0	No	No	NA	NA	NA	NA	NA	NA	NA	NA	NA	NA	Negative	No
0226	No	NA	No	Yes	5.33	Female	Yes	10.95	Mixed-breed	NA	2.5	1.0	No	No	NA	NA	NA	NA	NA	NA	NA	NA	NA	NA	Negative	No
0227	No	NA	No	Yes	3.50	Female	Yes	12.95	Mixed-breed	NA	4.0	1.0	No	No	NA	NA	NA	NA	NA	NA	NA	NA	NA	NA	Negative	No
0228	Yes	Test failure	No	No	3.42	Male	Yes	38.41	Mixed-breed	NA	4.0	2.0	No	No	NA	NA	NA	NA	NA	NA	NA	NA	NA	NA	Negative	No
0229	Yes	Test failure	No	No	6.25	Male	Yes	42.41	Mixed-breed	NA	4.0	1.0	No	No	NA	NA	NA	NA	NA	NA	NA	NA	NA	NA	Negative	No
0230	No	NA	No	Yes	8.50	Female	Yes	19.59	Mixed-breed	NA	1.0	2.0	No	No	NA	NA	NA	NA	NA	NA	NA	NA	NA	NA	Negative	No
0231	No	NA	No	Yes	8.00	Female	Yes	43.18	Purebred	Golden Retriever	2.0	2.0	No	No	NA	NA	NA	NA	NA	NA	NA	NA	NA	NA	Negative	No
0232	No	NA	No	Yes	2.33	Female	Yes	25.91	Mixed-breed	NA	2.5	1.0	No	No	NA	NA	NA	NA	NA	NA	NA	NA	NA	NA	Negative	No
0233	No	NA	No	Yes	10.00	Male	No	53.18	Purebred	Rottweiler	2.5	1.0	No	No	NA	NA	NA	NA	NA	NA	NA	NA	NA	NA	Negative	No
0234	Yes	Enrollment or Clinical Deviation	No	No	4.33	Male	Yes	22.73	Mixed-breed	NA	2.0	1.0	No	No	NA	NA	NA	NA	NA	NA	NA	NA	NA	NA	Negative	No
0235	No	NA	No	Yes	4.42	Female	Yes	35.45	Mixed-breed	NA	1.5	1.5	No	No	NA	NA	NA	NA	NA	NA	NA	NA	NA	NA	Negative	No
0236	No	NA	Yes	No	11.08	Male	Yes	11.82	Mixed-breed	NA	1.0	1.0	No	No	NA	NA	NA	NA	NA	NA	NA	NA	NA	NA	Negative	No
0237	No	NA	No	Yes	10.00	Female	Yes	29.55	Mixed-breed	NA	0.0	1.0	No	No	NA	NA	NA	NA	NA	NA	NA	NA	NA	NA	Negative	No
0238	No	NA	No	Yes	12.50	Male	Yes	29.55	Mixed-breed	NA	3.0	2.0	No	No	NA	NA	NA	NA	NA	NA	NA	NA	NA	NA	Negative	No
0239	No	NA	No	Yes	2.00	Male	Yes	16.82	Mixed-breed	NA	1.5	1.0	No	No	NA	NA	NA	NA	NA	NA	NA	NA	NA	NA	Negative	No
0240	Yes	Laboratory Workflow Deviation	No	No	3.00	Male	Yes	7.50	Mixed-breed	NA	1.0	1.0	No	No	NA	NA	NA	NA	NA	NA	NA	NA	NA	NA	Negative	No
0241	No	NA	Yes	No	6.33	Male	Yes	47.73	Purebred	Bernese Mountain Dogs	3.0	1.0	No	No	NA	NA	NA	NA	NA	NA	NA	NA	NA	NA	Positive	No
0242	Yes	Test failure	No	No	11.33	Female	Yes	36.36	Purebred	Golden Retriever	1.0	1.0	No	No	NA	NA	NA	NA	NA	NA	NA	NA	NA	NA	Fail	No
0243	No	NA	No	Yes	3.50	Female	Yes	29.55	Mixed-breed	NA	1.0	1.0	No	No	NA	NA	NA	NA	NA	NA	NA	NA	NA	NA	Negative	No
0244	Yes	Laboratory Workflow Deviation	No	No	14.00	Male	Yes	6.82	Mixed-breed	NA	2.0	1.0	No	No	NA	NA	NA	NA	NA	NA	NA	NA	NA	NA	Negative	No
0245	No	NA	No	Yes	10.75	Female	Yes	25.00	Mixed-breed	NA	1.0	2.0	No	No	NA	NA	NA	NA	NA	NA	NA	NA	NA	NA	Negative	No
0246	No	NA	No	Yes	1.08	Female	No	38.73	Purebred	Rottweiler	4.0	2.0	No	No	NA	NA	NA	NA	NA	NA	NA	NA	NA	NA	Negative	No
0247	No	NA	No	Yes	9.00	Female	Yes	22.73	Mixed-breed	NA	4.0	1.0	No	No	NA	NA	NA	NA	NA	NA	NA	NA	NA	NA	Negative	No
0248	No	NA	No	Yes	7.42	Male	Yes	27.27	Mixed-breed	NA	1.0	1.0	No	No	NA	NA	NA	NA	NA	NA	NA	NA	NA	NA	Negative	No
0249	No	NA	No	Yes	3.25	Female	Yes	16.36	Mixed-breed	NA	4.0	1.0	No	No	NA	NA	NA	NA	NA	NA	NA	NA	NA	NA	Negative	No
0250	No	NA	No	Yes	7.25	Male	Yes	25.00	Purebred	Poodle, Standard	3.0	1.0	No	No	NA	NA	NA	NA	NA	NA	NA	NA	NA	NA	Negative	No
0251	Yes	Test failure	No	No	5.42	Female	Yes	16.09	Mixed-breed	NA	4.0	2.0	No	No	NA	NA	NA	NA	NA	NA	NA	NA	NA	NA	Negative	No
0252	No	NA	No	Yes	1.75	Male	Yes	10.18	Purebred	Boston Terrier	4.0	2.0	No	No	NA	NA	NA	NA	NA	NA	NA	NA	NA	NA	Negative	No
0253	No	NA	No	Yes	5.67	Female	No	28.18	Purebred	Labrador Retriever	3.5	2.0	No	No	NA	NA	NA	NA	NA	NA	NA	NA	NA	NA	Negative	No
0254	No	NA	No	Yes	1.25	Female	Yes	11.09	Mixed-breed	NA	2.0	1.0	No	No	NA	NA	NA	NA	NA	NA	NA	NA	NA	NA	Negative	No
0255	No	NA	Yes	No	10.00	Male	Yes	15.27	Purebred	English Cocker Spaniel	4.0	2.0	No	No	NA	NA	NA	NA	NA	NA	NA	NA	NA	NA	Negative	No
0256	No	NA	No	Yes	2.50	Male	Yes	28.55	Mixed-breed	NA	4.0	2.0	No	No	NA	NA	NA	NA	NA	NA	NA	NA	NA	NA	Negative	No
0257	No	NA	No	Yes	1.33	Female	Yes	20.64	Mixed-breed	NA	3.5	2.0	No	No	NA	NA	NA	NA	NA	NA	NA	NA	NA	NA	Negative	No
0258	No	NA	No	Yes	3.00	Male	Yes	51.41	Purebred	Great Dane	2.5	1.0	No	No	NA	NA	NA	NA	NA	NA	NA	NA	NA	NA	Negative	No
0259	No	NA	No	Yes	4.92	Female	Yes	40.99	Mixed-breed	NA	2.5	2.0	No	No	NA	NA	NA	NA	NA	NA	NA	NA	NA	NA	Negative	No
0260	No	NA	No	Yes	4.42	Male	Yes	25.15	Purebred	Border Collie	4.0	2.0	No	No	NA	NA	NA	NA	NA	NA	NA	NA	NA	NA	Negative	No
0261	No	NA	Yes	No	2.08	Male	No	19.32	Purebred	Chesapeake Bay Retriever	2.0	1.0	No	No	NA	NA	NA	NA	NA	NA	NA	NA	NA	NA	Negative	No
0262	No	NA	No	Yes	2.33	Male	Yes	38.68	Purebred	German Shepherd	4.0	1.0	No	No	NA	NA	NA	NA	NA	NA	NA	NA	NA	NA	Negative	No
0263	Yes	Test failure	No	No	3.92	Male	Yes	58.62	Purebred	St. Bernard	2.0	2.0	No	No	NA	NA	NA	NA	NA	NA	NA	NA	NA	NA	Fail	No
0264	No	NA	No	Yes	9.50	Male	Yes	20.95	Mixed-breed	NA	3.5	1.0	No	No	NA	NA	NA	NA	NA	NA	NA	NA	NA	NA	Negative	No
0265	No	NA	No	Yes	5.33	Female	Yes	18.18	Mixed-breed	NA	2.0	2.0	No	No	NA	NA	NA	NA	NA	NA	NA	NA	NA	NA	Negative	No
0266	No	NA	No	Yes	2.00	Female	Yes	22.73	Mixed-breed	NA	1.0	2.0	No	No	NA	NA	NA	NA	NA	NA	NA	NA	NA	NA	Negative	No
0267	No	NA	No	Yes	5.00	Female	Yes	25.91	Purebred	Boxer	4.0	2.0	No	No	NA	NA	NA	NA	NA	NA	NA	NA	NA	NA	Negative	No
0268	No	NA	No	Yes	2.17	Female	Yes	6.51	Mixed-breed	NA	3.5	2.0	No	No	NA	NA	NA	NA	NA	NA	NA	NA	NA	NA	Negative	No
0269	No	NA	Yes	No	11.00	Male	Yes	24.09	Mixed-breed	NA	4.0	2.0	No	No	NA	NA	NA	NA	NA	NA	NA	NA	NA	NA	Negative	No
0270	No	NA	No	Yes	3.00	Male	No	49.00	Purebred	Mastiff	3.0	1.5	No	No	NA	NA	NA	NA	NA	NA	NA	NA	NA	NA	Negative	No
0271	No	NA	Yes	No	8.67	Female	Yes	28.64	Mixed-breed	NA	3.0	1.0	No	No	NA	NA	NA	NA	NA	NA	NA	NA	NA	NA	Negative	No
0272	No	NA	No	Yes	9.67	Female	Yes	32.73	Mixed-breed	NA	3.5	1.5	No	No	NA	NA	NA	NA	NA	NA	NA	NA	NA	NA	Negative	No
0273	No	NA	Yes	No	8.08	Female	Yes	26.82	Mixed-breed	NA	4.0	2.0	No	No	NA	NA	NA	NA	NA	NA	NA	NA	NA	NA	Negative	No
0274	No	NA	No	Yes	3.00	Male	Yes	20.00	Purebred	English Setter	3.0	1.0	No	No	NA	NA	NA	NA	NA	NA	NA	NA	NA	NA	Negative	No
0275	Yes	Enrollment or Clinical Deviation	No	No	12.67	Female	Yes	22.73	Purebred	Basset Hound	4.0	2.0	No	No	NA	NA	NA	NA	NA	NA	NA	NA	NA	NA	Negative	No
0276	No	NA	No	Yes	10.00	Male	Yes	34.09	Mixed-breed	NA	2.5	2.0	No	No	NA	NA	NA	NA	NA	NA	NA	NA	NA	NA	Negative	No
0277	No	NA	No	Yes	10.00	Male	Yes	25.91	Purebred	English Setter	2.0	1.0	No	No	NA	NA	NA	NA	NA	NA	NA	NA	NA	NA	Negative	No
0278	No	NA	No	Yes	7.25	Female	Yes	48.86	Mixed-breed	NA	1.5	1.0	No	No	NA	NA	NA	NA	NA	NA	NA	NA	NA	NA	Negative	No
0279	No	NA	Yes	No	6.00	Female	Yes	14.23	Mixed-breed	NA	4.0	2.0	No	No	NA	NA	NA	NA	NA	NA	NA	NA	NA	NA	Negative	No

S1 Table. Full subject level data for subjects enrolled in the CANDiD study (continued)

See legend on last page.

Subject	Excluded	Reason for Exclusion	Training Set	Testing Set	Age	Sex	Spay/ Neuter	Weight (kg)	Purebred or Mixed Breed	Breed (Purebred)	Hemolysis	Lipemia	Cancer Diagnosis at Enrollment	Cancer Diagnosed after Liquid Biopsy	Cancer Type	Immunophenotype (Lymphoid Cancer)	Extent of Disease: Non-Lymphoid Cancer	Extent of Disease: Lymphoid Cancer	Tumor Size	Cancer Type (Cancer #2)	Immunophenotype (Cancer #2 if Lymphoid)	Extent of Disease: Non-Lymphoid Cancer (Cancer #2)	Extent of Disease: Lymphoid Cancer (Cancer #2)	Tumor Size (Cancer #2)	Liquid Biopsy Result	CSO Prediction of Hematological Malignancy
0280	No	NA	Yes	No	7.25	Female	Yes	33.32	Mixed-breed	NA	3.0	1.0	No	No	NA	NA	NA	NA	NA	NA	NA	NA	NA	NA	Negative	No
0281	No	NA	Yes	No	10.00	Female	Yes	30.91	Purebred	Labrador Retriever	2.0	2.0	No	No	NA	NA	NA	NA	NA	NA	NA	NA	NA	NA	Negative	No
0282	No	NA	Yes	No	1.00	Male	Yes	34.09	Mixed-breed	NA	3.0	1.5	No	No	NA	NA	NA	NA	NA	NA	NA	NA	NA	NA	Negative	No
0283	No	NA	No	Yes	2.50	Male	Yes	23.00	Purebred	English Bulldog	3.0	1.0	No	No	NA	NA	NA	NA	NA	NA	NA	NA	NA	NA	Negative	No
0284	No	NA	No	Yes	6.50	Male	Yes	35.45	Purebred	German Shepherd	4.0	2.0	No	No	NA	NA	NA	NA	NA	NA	NA	NA	NA	NA	Negative	No
0286	No	NA	Yes	No	6.67	Female	Yes	30.91	Purebred	Doberman Pinscher	3.0	1.0	No	No	NA	NA	NA	NA	NA	NA	NA	NA	NA	NA	Negative	No
0287	No	NA	Yes	No	6.83	Female	Yes	12.36	Purebred	French Bulldog	4.0	2.0	No	No	NA	NA	NA	NA	NA	NA	NA	NA	NA	NA	Negative	No
0288	No	NA	No	Yes	4.00	Female	Yes	55.14	Purebred	Great Dane	2.5	2.0	No	No	NA	NA	NA	NA	NA	NA	NA	NA	NA	NA	Negative	No
0289	No	NA	No	Yes	11.00	Male	Yes	18.91	Mixed-breed	NA	2.0	0.0	No	No	NA	NA	NA	NA	NA	NA	NA	NA	NA	NA	Negative	No
0290	No	NA	No	Yes	6.33	Male	Yes	56.64	Mixed-breed	NA	2.5	2.0	No	No	NA	NA	NA	NA	NA	NA	NA	NA	NA	NA	Negative	No
0291	No	NA	No	Yes	5.33	Female	Yes	38.45	Purebred	Golden Retriever	3.0	2.0	No	No	NA	NA	NA	NA	NA	NA	NA	NA	NA	NA	Negative	No
0292	No	NA	No	Yes	3.00	Female	Yes	47.73	Purebred	Great Dane	3.0	1.0	No	No	NA	NA	NA	NA	NA	NA	NA	NA	NA	NA	Negative	No
0293	No	NA	No	Yes	9.17	Female	Yes	16.64	Purebred	Australian Shepherd	2.0	1.0	No	No	NA	NA	NA	NA	NA	NA	NA	NA	NA	NA	Negative	No
0294	No	NA	No	Yes	6.00	Female	Yes	30.95	Mixed-breed	NA	2.5	1.0	No	No	NA	NA	NA	NA	NA	NA	NA	NA	NA	NA	Negative	No
0295	No	NA	No	Yes	5.33	Male	No	9.18	Purebred	Scottish Terrier	0.0	1.0	No	No	NA	NA	NA	NA	NA	NA	NA	NA	NA	NA	Negative	No
0296	No	NA	No	Yes	7.17	Female	Yes	12.00	Purebred	Scottish Terrier	2.0	0.0	No	No	NA	NA	NA	NA	NA	NA	NA	NA	NA	NA	Negative	No
0297	No	NA	No	Yes	1.58	Female	Yes	25.45	Mixed-breed	NA	4.0	2.0	No	No	NA	NA	NA	NA	NA	NA	NA	NA	NA	NA	Negative	No
0298	No	NA	No	Yes	4.58	Female	Yes	12.27	Mixed-breed	NA	2.5	1.0	No	No	NA	NA	NA	NA	NA	NA	NA	NA	NA	NA	Negative	No
0299	No	NA	Yes	No	8.00	Female	Yes	34.47	Mixed-breed	NA	2.0	1.0	No	No	NA	NA	NA	NA	NA	NA	NA	NA	NA	NA	Negative	No
0300	No	NA	No	Yes	2.75	Female	Yes	23.85	Mixed-breed	NA	3.0	2.0	No	No	NA	NA	NA	NA	NA	NA	NA	NA	NA	NA	Negative	No
0301	No	NA	Yes	No	1.17	Female	Yes	36.27	Purebred	German Shepherd	4.0	2.0	No	No	NA	NA	NA	NA	NA	NA	NA	NA	NA	NA	Negative	No
0302	No	NA	No	Yes	6.33	Male	Yes	27.06	Purebred	Labrador Retriever	4.0	2.0	No	No	NA	NA	NA	NA	NA	NA	NA	NA	NA	NA	Negative	No
0303	No	NA	No	Yes	5.25	Male	Yes	28.16	Mixed-breed	NA	3.5	2.0	No	No	NA	NA	NA	NA	NA	NA	NA	NA	NA	NA	Negative	No
0304	No	NA	No	Yes	13.08	Male	Yes	19.34	Mixed-breed	NA	3.0	2.0	No	No	NA	NA	NA	NA	NA	NA	NA	NA	NA	NA	Negative	No
0305	No	NA	No	Yes	3.50	Female	Yes	14.03	Mixed-breed	NA	2.0	2.0	No	No	NA	NA	NA	NA	NA	NA	NA	NA	NA	NA	Negative	No
0306	No	NA	Yes	No	10.00	Male	Yes	18.41	Purebred	Cardigan Welsh Corgi	3.0	2.0	No	No	NA	NA	NA	NA	NA	NA	NA	NA	NA	NA	Negative	No
0307	No	NA	No	Yes	9.00	Female	Yes	31.36	Purebred	Labrador Retriever	2.5	1.5	No	No	NA	NA	NA	NA	NA	NA	NA	NA	NA	NA	Negative	No
0308	No	NA	Yes	No	7.67	Male	Yes	28.73	Mixed-breed	NA	3.5	1.0	No	No	NA	NA	NA	NA	NA	NA	NA	NA	NA	NA	Negative	No
0309	No	NA	No	Yes	2.33	Male	Yes	33.86	Purebred	Labrador Retriever	3.0	1.0	No	No	NA	NA	NA	NA	NA	NA	NA	NA	NA	NA	Negative	No
0310	No	NA	No	Yes	7.92	Female	Yes	30.32	Purebred	Labrador Retriever	2.0	1.0	No	No	NA	NA	NA	NA	NA	NA	NA	NA	NA	NA	Negative	No
0311	No	NA	No	Yes	1.58	Male	Yes	29.86	Mixed-breed	NA	4.0	1.0	No	No	NA	NA	NA	NA	NA	NA	NA	NA	NA	NA	Negative	No
0312	No	NA	Yes	No	7.75	Female	Yes	20.45	Mixed-breed	NA	2.5	1.0	No	No	NA	NA	NA	NA	NA	NA	NA	NA	NA	NA	Negative	No
0313	No	NA	Yes	No	12.92	Male	Yes	14.23	Mixed-breed	NA	4.0	1.0	No	No	NA	NA	NA	NA	NA	NA	NA	NA	NA	NA	Negative	No
0314	No	NA	No	Yes	7.17	Male	Yes	42.59	Purebred	Labrador Retriever	2.5	1.0	No	No	NA	NA	NA	NA	NA	NA	NA	NA	NA	NA	Negative	No
0315	No	NA	No	Yes	2.42	Male	Yes	14.73	Purebred	Australian Shepherd	3.0	1.0	No	No	NA	NA	NA	NA	NA	NA	NA	NA	NA	NA	Negative	No
0316	No	NA	No	Yes	1.00	Female	Yes	23.64	Mixed-breed	NA	1.0	1.0	No	No	NA	NA	NA	NA	NA	NA	NA	NA	NA	NA	Negative	No
0317	No	NA	No	Yes	3.42	Female	Yes	31.18	Purebred	Labrador Retriever	4.0	2.0	No	No	NA	NA	NA	NA	NA	NA	NA	NA	NA	NA	Negative	No
0318	No	NA	No	Yes	1.67	Male	Yes	45.45	Purebred	Labrador Retriever	4.0	2.0	No	No	NA	NA	NA	NA	NA	NA	NA	NA	NA	NA	Negative	No
0319	Yes	Laboratory Workflow Deviation	No	No	1.58	Male	Yes	25.82	Purebred	American Staffordshire Terrier	3.5	1.5	No	No	NA	NA	NA	NA	NA	NA	NA	NA	NA	NA	Negative	No
0320	No	NA	No	Yes	7.00	Male	Yes	33.00	Purebred	Labrador Retriever	3.0	2.0	No	No	NA	NA	NA	NA	NA	NA	NA	NA	NA	NA	Negative	No
0321	No	NA	No	Yes	6.08	Male	Yes	106.82	Purebred	Mastiff	3.5	1.0	No	No	NA	NA	NA	NA	NA	NA	NA	NA	NA	NA	Negative	No
0322	Yes	Laboratory Workflow Deviation	No	No	2.58	Female	Yes	23.09	Purebred	Wirehairded Pointing Griffon	4.0	2.0	No	No	NA	NA	NA	NA	NA	NA	NA	NA	NA	NA	Negative	No
0323	No	NA	No	Yes	4.58	Male	Yes	37.27	Purebred	Golden Retriever	1.0	1.0	No	No	NA	NA	NA	NA	NA	NA	NA	NA	NA	NA	Negative	No
0324	No	NA	No	Yes	1.33	Male	Yes	31.91	Mixed-breed	NA	3.0	2.0	No	No	NA	NA	NA	NA	NA	NA	NA	NA	NA	NA	Negative	No
0325	No	NA	No	Yes	7.00	Female	Yes	37.27	Purebred	Golden Retriever	2.0	1.5	No	No	NA	NA	NA	NA	NA	NA	NA	NA	NA	NA	Negative	No
0326	No	NA	No	Yes	1.08	Male	Yes	32.23	Mixed-breed	NA	1.0	1.0	No	No	NA	NA	NA	NA	NA	NA	NA	NA	NA	NA	Positive	No
0327	No	NA	No	Yes	7.42	Female	Yes	35.23	Purebred	Golden Retriever	3.0	1.0	No	No	NA	NA	NA	NA	NA	NA	NA	NA	NA	NA	Negative	No
0328	No	NA	Yes	No	12.33	Male	Yes	17.05	Purebred	Border Collie	2.0	1.0	No	No	NA	NA	NA	NA	NA	NA	NA	NA	NA	NA	Negative	No
0329	No	NA	No	Yes	2.25	Female	Yes	25.23	Purebred	Labrador Retriever	2.0	1.0	No	No	NA	NA	NA	NA	NA	NA	NA	NA	NA	NA	Negative	No
0330	No	NA	No	Yes	2.50	Male	Yes	22.82	Purebred	Welsh Springer Spaniel	2.0	1.0	No	No	NA	NA	NA	NA	NA	NA	NA	NA	NA	NA	Negative	No
0331	No	NA	No	Yes	4.00	Female	Yes	22.73	Purebred	Australian Cattle Dog	3.5	2.0	No	No	NA	NA	NA	NA	NA	NA	NA	NA	NA	NA	Negative	No
0332	No	NA	No	Yes	3.00	Male	Yes	37.73	Mixed-breed	NA	4.0	2.0	No	No	NA	NA	NA	NA	NA	NA	NA	NA	NA	NA	Negative	No
0333	No	NA	Yes	No	12.50	Male	Yes	13.09	Mixed-breed	NA	2.0	2.0	No	No	NA	NA	NA	NA	NA	NA	NA	NA	NA	NA	Negative	No
0334	No	NA	No	Yes	8.00	Female	Yes	26.95	Mixed-breed	NA	1.0	0.0	No	No	NA	NA	NA	NA	NA	NA	NA	NA	NA	NA	Positive	No
0335	No	NA	No	Yes	8.00	Male	Yes	38.59	Purebred	Golden Retriever	2.5	1.0	No	No	NA	NA	NA	NA	NA	NA	NA	NA	NA	NA	Negative	No
0336	Yes	Test failure	No	No	7.08	Male	Yes	40.18	Mixed-breed	NA	2.0	2.0	No	No	NA	NA	NA	NA	NA	NA	NA	NA	NA	NA	Fail	No
0337	No	NA	No	Yes	3.00	Male	Yes	11.59	Mixed-breed	NA	3.0	2.0	No	No	NA	NA	NA	NA	NA	NA	NA	NA	NA	NA	Negative	No
0338	No	NA	Yes	No	12.00	Male	Yes	16.14	Mixed-breed	NA	2.0	1.0	No	No	NA	NA	NA	NA	NA	NA	NA	NA	NA	NA	Negative	No
0339	No	NA	No	Yes	2.33	Male	Yes	11.45	Mixed-breed	NA	3.5	1.5	No	No	NA	NA	NA	NA	NA	NA	NA	NA	NA	NA	Negative	No
0340	No	NA	Yes	No	11.08	Male	Yes	27.23	Mixed-breed	NA	2.0	2.0	No	No	NA	NA	NA	NA	NA	NA	NA	NA	NA	NA	Negative	No
0341	Yes	Laboratory Workflow Deviation	No	No	10.50	Male	Yes	12.27	Purebred	Australian Shepherd	4.0	2.0	No	No	NA	NA	NA	NA	NA	NA	NA	NA	NA	NA	Negative	No
0342	No	NA	No	Yes	7.00	Male	Yes	36.45	Mixed-breed	NA	4.0	2.0	No	No	NA	NA	NA	NA	NA	NA	NA	NA	NA	NA	Negative	No
0343	No	NA	Yes	No	5.50	Female	Yes	27.45	Mixed-breed	NA	3.0	2.0	No	No	NA	NA	NA	NA	NA	NA	NA	NA	NA	NA	Negative	No
0344	No	NA	No	Yes	2.58	Male	Yes	34.09	Purebred	Golden Retriever	2.5	1.0	No	No	NA	NA	NA	NA	NA	NA	NA	NA	NA	NA	Negative	No
0345	No	NA	No	Yes	2.42	Male	No	29.77	Purebred	Labrador Retriever	4.0	2.0	No	No	NA	NA	NA	NA	NA	NA	NA	NA	NA	NA	Negative	No
0351	No	NA	No	Yes	10.42	Female	Yes	29.09	Purebred	Labrador Retriever	4.0	2.0	No	No	NA	NA	NA	NA	NA	NA	NA	NA	NA	NA	Negative	No
0352	Yes	Laboratory Workflow Deviation	No	No	1.25	Female	No	15.00	Purebred	French Bulldog	4.0	2.0	No	No	NA	NA	NA	NA	NA	NA	NA	NA	NA	NA	Negative	No
0353	No	NA	No	Yes	9.00	Male	No	30.36	Purebred	English Springer Spaniel	3.0	1.0	No	No	NA	NA	NA	NA	NA	NA	NA	NA	NA	NA	Negative	No
0354	No	NA	Yes	No	5.25	Male	Yes	25.09	Mixed-breed	NA	2.0	1.0	No	No	NA	NA	NA	NA	NA	NA	NA	NA	NA	NA	Negative	No
0355	No	NA	Yes	No	11.67	Female	Yes	20.50	Purebred	McNab Shepherd/McNab Sheepdog	4.0	2.0	No	No	NA	NA	NA	NA	NA	NA	NA	NA	NA	NA	Negative	No

S1 Table. Full subject level data for subjects enrolled in the CANDiD study (continued)

See legend on last page.

Subject	Excluded	Reason for Exclusion	Training Set	Testing Set	Age	Sex	Spay/ Neuter	Weight (kg)	Purebred or Mixed Breed	Breed (Purebred)	Hemolysis	Lipemia	Cancer Diagnosis at Enrollment	Cancer Diagnosed after Liquid Biopsy	Cancer Type	Immunophenotype (Lymphoid Cancer)	Extent of Disease: Non-Lymphoid Cancer	Extent of Disease: Lymphoid Cancer	Tumor Size	Cancer Type (Cancer #2)	Immunophenotype (Cancer #2 if Lymphoid)	Extent of Disease: Non-Lymphoid Cancer (Cancer #2)	Extent of Disease: Lymphoid Cancer (Cancer #2)	Tumor Size (Cancer #2)	Liquid Biopsy Result	CSO Prediction of Hematological Malignancy
0356	No	NA	No	Yes	6.58	Male	Yes	36.45	Purebred	Labrador Retriever	3.0	1.0	No	No	NA	NA	NA	NA	NA	NA	NA	NA	NA	NA	Negative	No
0357	No	NA	No	Yes	7.58	Female	Yes	8.82	Purebred	Shih Tzu	3.0	1.5	No	No	NA	NA	NA	NA	NA	NA	NA	NA	NA	NA	Negative	No
0358	No	NA	No	Yes	6.92	Male	Yes	39.55	Purebred	Greyhound	4.0	1.0	No	No	NA	NA	NA	NA	NA	NA	NA	NA	NA	NA	Negative	No
0359	No	NA	No	Yes	2.50	Male	Yes	36.36	Mixed-breed	NA	2.0	1.0	No	No	NA	NA	NA	NA	NA	NA	NA	NA	NA	NA	Negative	No
0360	No	NA	Yes	No	3.50	Female	No	30.56	Purebred	Golden Retriever	1.0	0.0	No	No	NA	NA	NA	NA	NA	NA	NA	NA	NA	NA	Negative	No
0361	No	NA	No	Yes	5.92	Male	Yes	54.09	Purebred	Bloodhound	0.5	1.0	No	No	NA	NA	NA	NA	NA	NA	NA	NA	NA	NA	Negative	No
0362	No	NA	No	Yes	11.00	Male	Yes	36.23	Purebred	Pointer	2.0	2.0	No	No	NA	NA	NA	NA	NA	NA	NA	NA	NA	NA	Negative	No
0363	No	NA	No	Yes	5.75	Female	Yes	41.41	Purebred	Bloodhound	2.5	2.0	No	No	NA	NA	NA	NA	NA	NA	NA	NA	NA	NA	Negative	No
0364	No	NA	No	Yes	1.58	Female	Yes	17.77	Mixed-breed	NA	1.0	2.0	No	No	NA	NA	NA	NA	NA	NA	NA	NA	NA	NA	Negative	No
0365	No	NA	Yes	No	14.17	Female	Yes	37.91	Mixed-breed	NA	1.0	1.0	No	No	NA	NA	NA	NA	NA	NA	NA	NA	NA	NA	Negative	No
0366	No	NA	Yes	No	7.00	Female	Yes	17.77	Mixed-breed	NA	2.0	2.0	No	No	NA	NA	NA	NA	NA	NA	NA	NA	NA	NA	Negative	No
0367	No	NA	No	Yes	7.50	Female	Yes	50.68	Purebred	Akita	2.0	1.0	No	No	NA	NA	NA	NA	NA	NA	NA	NA	NA	NA	Negative	No
0368	Yes	Laboratory Workflow Deviation	No	No	4.25	Male	Yes	29.77	Mixed-breed	NA	4.0	2.0	No	No	NA	NA	NA	NA	NA	NA	NA	NA	NA	NA	Negative	No
0369	No	NA	No	Yes	3.83	Female	Yes	15.82	Mixed-breed	NA	3.5	2.0	No	No	NA	NA	NA	NA	NA	NA	NA	NA	NA	NA	Negative	No
0370	No	NA	Yes	No	5.92	Female	Yes	42.50	Purebred	Rottweiler	1.0	1.0	No	No	NA	NA	NA	NA	NA	NA	NA	NA	NA	NA	Negative	No
0371	No	NA	No	Yes	11.92	Female	Yes	17.54	Purebred	Silken Windhound	3.5	1.0	No	No	NA	NA	NA	NA	NA	NA	NA	NA	NA	NA	Negative	No
0372	No	NA	Yes	No	5.83	Female	Yes	26.45	Purebred	Golden Retriever	3.0	2.0	No	No	NA	NA	NA	NA	NA	NA	NA	NA	NA	NA	Negative	No
0373	No	NA	Yes	No	6.75	Female	Yes	32.45	Purebred	Golden Retriever	2.0	1.0	No	No	NA	NA	NA	NA	NA	NA	NA	NA	NA	NA	Negative	No
0374	No	NA	No	Yes	7.75	Female	Yes	37.18	Mixed-breed	NA	2.0	0.0	No	Yes	NA	NA	NA	NA	NA	NA	NA	NA	NA	NA	Positive	No
0375	No	NA	Yes	No	6.00	Male	Yes	44.18	Mixed-breed	NA	3.5	2.0	No	No	NA	NA	NA	NA	NA	NA	NA	NA	NA	NA	Negative	No
0376	No	NA	Yes	No	1.00	Female	Yes	27.05	Mixed-breed	NA	2.5	2.0	No	No	NA	NA	NA	NA	NA	NA	NA	NA	NA	NA	Negative	No
0377	No	NA	No	Yes	5.00	Male	No	68.32	Purebred	Mastiff	1.5	1.0	No	No	NA	NA	NA	NA	NA	NA	NA	NA	NA	NA	Negative	No
0378	No	NA	No	Yes	9.42	Male	Yes	43.18	Purebred	Labrador Retriever	4.0	2.0	No	No	NA	NA	NA	NA	NA	NA	NA	NA	NA	NA	Negative	No
0379	No	NA	No	Yes	1.92	Male	Yes	30.18	Purebred	Labrador Retriever	3.0	1.0	No	No	NA	NA	NA	NA	NA	NA	NA	NA	NA	NA	Negative	No
0380	No	NA	No	Yes	6.00	Female	Yes	14.23	Purebred	Pembroke Welsh Corgi	3.5	2.0	No	No	NA	NA	NA	NA	NA	NA	NA	NA	NA	NA	Negative	No
0381	No	NA	Yes	No	4.42	Female	Yes	29.45	Purebred	German Shepherd	3.0	1.0	No	No	NA	NA	NA	NA	NA	NA	NA	NA	NA	NA	Negative	No
0382	No	NA	No	Yes	12.33	Female	Yes	21.36	Purebred	Golden Retriever	1.5	1.0	No	No	NA	NA	NA	NA	NA	NA	NA	NA	NA	NA	Negative	No
0383	No	NA	No	Yes	5.17	Male	Yes	33.82	Purebred	Boxer	3.0	1.0	No	No	NA	NA	NA	NA	NA	NA	NA	NA	NA	NA	Positive	No
0384	No	NA	No	Yes	5.25	Female	Yes	12.05	Purebred	Australian Cattle Dog	3.0	1.0	No	No	NA	NA	NA	NA	NA	NA	NA	NA	NA	NA	Negative	No
0385	No	NA	No	Yes	3.00	Female	Yes	21.77	Purebred	Anatolian Shepherd	1.0	1.0	No	No	NA	NA	NA	NA	NA	NA	NA	NA	NA	NA	Negative	No
0386	Yes	Enrollment or Clinical Deviation	No	No	6.00	Male	Yes	24.05	Purebred	Poodle, Standard	1.0	1.0	No	No	NA	NA	NA	NA	NA	NA	NA	NA	NA	NA	Negative	No
0387	No	NA	No	Yes	5.50	Male	No	34.07	Purebred	Golden Retriever	2.5	2.0	No	No	NA	NA	NA	NA	NA	NA	NA	NA	NA	NA	Negative	No
0388	No	NA	No	Yes	3.50	Male	No	33.27	Purebred	Golden Retriever	1.0	2.0	No	No	NA	NA	NA	NA	NA	NA	NA	NA	NA	NA	Negative	No
0389	No	NA	No	Yes	3.08	Male	Yes	30.36	Mixed-breed	NA	4.0	2.0	No	No	NA	NA	NA	NA	NA	NA	NA	NA	NA	NA	Negative	No
0390	No	NA	No	Yes	5.08	Female	Yes	21.45	Purebred	Belgian Malinois	3.5	2.0	No	No	NA	NA	NA	NA	NA	NA	NA	NA	NA	NA	Negative	No
0391	No	NA	No	Yes	7.25	Female	Yes	18.00	Mixed-breed	NA	4.0	2.0	No	No	NA	NA	NA	NA	NA	NA	NA	NA	NA	NA	Negative	No
0392	No	NA	No	Yes	2.33	Female	Yes	11.05	Mixed-breed	NA	4.0	2.0	No	No	NA	NA	NA	NA	NA	NA	NA	NA	NA	NA	Negative	No
0393	No	NA	No	Yes	2.17	Male	Yes	22.41	Purebred	Basset Hound	3.0	2.0	No	No	NA	NA	NA	NA	NA	NA	NA	NA	NA	NA	Negative	No
0394	Yes	Laboratory Workflow Deviation	No	No	7.00	Female	Yes	66.64	Purebred	Irish Wolfhound	4.0	1.0	No	No	NA	NA	NA	NA	NA	NA	NA	NA	NA	NA	Negative	No
0395	No	NA	No	Yes	1.50	Male	No	19.84	Purebred	Stabyhoun	4.0	2.0	No	No	NA	NA	NA	NA	NA	NA	NA	NA	NA	NA	Negative	No
0396	No	NA	No	Yes	4.50	Male	Yes	32.18	Purebred	Gordon Setter	3.0	1.0	No	No	NA	NA	NA	NA	NA	NA	NA	NA	NA	NA	Negative	No
0397	Yes	Laboratory Workflow Deviation	No	No	8.25	Male	Yes	12.27	Mixed-breed	NA	4.0	2.0	No	No	NA	NA	NA	NA	NA	NA	NA	NA	NA	NA	Negative	No
0398	No	NA	Yes	No	3.75	Male	Yes	46.45	Purebred	Chesapeake Bay Retriever	3.0	2.0	No	No	NA	NA	NA	NA	NA	NA	NA	NA	NA	NA	Negative	No
0399	No	NA	Yes	No	7.00	Female	Yes	75.16	Purebred	German Shepherd	3.0	2.0	No	No	NA	NA	NA	NA	NA	NA	NA	NA	NA	NA	Negative	No
0400	Yes	Test failure	No	No	15.08	Male	Yes	13.68	Purebred	Dachshund	4.0	2.0	No	No	NA	NA	NA	NA	NA	NA	NA	NA	NA	NA	Negative	No
0401	No	NA	No	Yes	2.50	Male	Yes	29.64	Purebred	Poodle, Standard	2.0	2.0	No	No	NA	NA	NA	NA	NA	NA	NA	NA	NA	NA	Negative	No
0402	No	NA	No	Yes	4.17	Female	Yes	31.77	Purebred	Labrador Retriever	3.0	0.5	No	No	NA	NA	NA	NA	NA	NA	NA	NA	NA	NA	Negative	No
0403	No	NA	No	Yes	4.25	Female	Yes	38.18	Mixed-breed	NA	4.0	2.0	No	No	NA	NA	NA	NA	NA	NA	NA	NA	NA	NA	Negative	No
0404	No	NA	No	Yes	1.17	Male	Yes	37.09	Mixed-breed	NA	1.5	1.0	No	No	NA	NA	NA	NA	NA	NA	NA	NA	NA	NA	Negative	No
0405	No	NA	No	Yes	5.08	Male	Yes	30.82	Purebred	Golden Retriever	4.0	2.0	No	No	NA	NA	NA	NA	NA	NA	NA	NA	NA	NA	Negative	No
0406	No	NA	No	Yes	8.92	Male	Yes	11.09	Mixed-breed	NA	2.5	1.0	No	No	NA	NA	NA	NA	NA	NA	NA	NA	NA	NA	Negative	No
0407	No	NA	No	Yes	1.00	Male	Yes	36.68	Purebred	Labrador Retriever	1.0	1.0	No	No	NA	NA	NA	NA	NA	NA	NA	NA	NA	NA	Negative	No
0408	No	NA	No	Yes	1.83	Female	Yes	24.36	Mixed-breed	NA	2.5	1.0	No	No	NA	NA	NA	NA	NA	NA	NA	NA	NA	NA	Negative	No
0409	No	NA	Yes	No	7.67	Female	Yes	12.14	Mixed-breed	NA	4.0	1.0	No	No	NA	NA	NA	NA	NA	NA	NA	NA	NA	NA	Negative	No
0410	Yes	Laboratory Workflow Deviation	No	No	3.92	Female	Yes	11.73	Mixed-breed	NA	3.5	2.0	No	No	NA	NA	NA	NA	NA	NA	NA	NA	NA	NA	Negative	No
0411	No	NA	No	Yes	4.33	Male	Yes	39.77	Purebred	American Staffordshire Terrier	2.0	2.0	No	No	NA	NA	NA	NA	NA	NA	NA	NA	NA	NA	Negative	No
0412	No	NA	No	Yes	4.58	Male	Yes	31.55	Purebred	American Staffordshire Terrier	3.5	2.0	No	No	NA	NA	NA	NA	NA	NA	NA	NA	NA	NA	Negative	No
0413	No	NA	No	Yes	2.67	Female	Yes	29.64	Purebred	Chesapeake Bay Retriever	1.0	1.0	No	No	NA	NA	NA	NA	NA	NA	NA	NA	NA	NA	Negative	No
0414	No	NA	Yes	No	8.67	Female	Yes	30.27	Mixed-breed	NA	3.0	1.0	No	No	NA	NA	NA	NA	NA	NA	NA	NA	NA	NA	Negative	No
0415	No	NA	No	Yes	4.08	Male	Yes	24.18	Purebred	Australian Shepherd	1.5	2.0	No	No	NA	NA	NA	NA	NA	NA	NA	NA	NA	NA	Negative	No
0416	Yes	Laboratory Workflow Deviation	No	No	11.75	Female	Yes	24.09	Mixed-breed	NA	4.0	1.0	No	No	NA	NA	NA	NA	NA	NA	NA	NA	NA	NA	Negative	No
0417	No	NA	No	Yes	7.67	Female	Yes	17.50	Mixed-breed	NA	1.0	1.0	No	No	NA	NA	NA	NA	NA	NA	NA	NA	NA	NA	Negative	No
0418	No	NA	No	Yes	1.33	Male	Yes	45.45	Mixed-breed	NA	1.5	1.5	No	No	NA	NA	NA	NA	NA	NA	NA	NA	NA	NA	Negative	No
0419	No	NA	No	Yes	3.17	Female	Yes	21.14	Purebred	Poodle, Standard	2.0	1.0	No	No	NA	NA	NA	NA	NA	NA	NA	NA	NA	NA	Negative	No
0420	Yes	Test failure	No	No	6.83	Female	Yes	28.76	Purebred	English Bulldog	4.0	2.0	No	No	NA	NA	NA	NA	NA	NA	NA	NA	NA	NA	Fail	No
0421	No	NA	No	Yes	5.42	Female	Yes	31.18	Purebred	Golden Retriever	3.0	2.0	No	No	NA	NA	NA	NA	NA	NA	NA	NA	NA	NA	Negative	No
0422	Yes	Laboratory Workflow Deviation	No	No	4.00	Male	Yes	16.14	Mixed-breed	NA	3.0	2.0	No	No	NA	NA	NA	NA	NA	NA	NA	NA	NA	NA	Negative	No

See legend on last page.

Subject	Excluded	Reason for Exclusion	Training Set	Testing Set	Age	Sex	Spay/ Neuter	Weight (kg)	Purebred or Mixed Breed	Breed (Purebred)	Hemolysis	Lipemia	Cancer Diagnosis at Enrollment	Cancer Diagnosed after Liquid Biopsy	Cancer Type	Immunophenotype (Lymphoid Cancer)	Extent of Disease: Non-Lymphoid Cancer	Extent of Disease: Lymphoid Cancer	Tumor Size	Cancer Type (Cancer #2)	Immunophenotype (Cancer #2 if Lymphoid)	Extent of Disease: Non-Lymphoid Cancer (Cancer #2)	Extent of Disease: Lymphoid Cancer (Cancer #2)	Tumor Size (Cancer #2)	Liquid Biopsy Result	CSO Prediction of Hematological Malignancy
0423	No	NA	No	Yes	4.67	Male	Yes	40.18	Mixed-breed	NA	2.0	1.0	No	No	NA	NA	NA	NA	NA	NA	NA	NA	NA	NA	Negative	No
0424	No	NA	No	Yes	2.00	Male	Yes	28.73	Mixed-breed	NA	3.5	1.0	No	No	NA	NA	NA	NA	NA	NA	NA	NA	NA	NA	Negative	No
0425	No	NA	No	Yes	2.50	Male	Yes	52.73	Purebred	Great Dane	1.5	1.0	No	No	NA	NA	NA	NA	NA	NA	NA	NA	NA	NA	Negative	No
0426	No	NA	No	Yes	2.00	Female	Yes	16.32	Purebred	Border Collie	3.0	1.0	No	No	NA	NA	NA	NA	NA	NA	NA	NA	NA	NA	Negative	No
0427	Yes	Laboratory Workflow Deviation	No	No	4.58	Female	Yes	28.27	Purebred	Boxer	3.5	2.0	No	No	NA	NA	NA	NA	NA	NA	NA	NA	NA	NA	Negative	No
0428	No	NA	No	Yes	3.08	Male	Yes	36.95	Purebred	Labrador Retriever	1.0	1.0	No	No	NA	NA	NA	NA	NA	NA	NA	NA	NA	NA	Negative	No
0429	No	NA	No	Yes	1.58	Male	Yes	40.14	Purebred	Golden Retriever	1.5	1.0	No	No	NA	NA	NA	NA	NA	NA	NA	NA	NA	NA	Negative	No
0430	No	NA	No	Yes	6.08	Male	Yes	27.86	Mixed-breed	NA	2.0	1.0	No	No	NA	NA	NA	NA	NA	NA	NA	NA	NA	NA	Negative	No
0431	No	NA	No	Yes	6.00	Male	Yes	12.27	Mixed-breed	NA	2.5	1.5	No	No	NA	NA	NA	NA	NA	NA	NA	NA	NA	NA	Negative	No
0432	Yes	Enrollment or Clinical Deviation	No	No	10.00	Female	Yes	21.14	Purebred	Australian Shepherd	2.0	0.0	No	No	NA	NA	NA	NA	NA	NA	NA	NA	NA	NA	Negative	No
0433	No	NA	No	Yes	5.00	Male	Yes	40.45	Mixed-breed	NA	2.5	1.0	No	No	NA	NA	NA	NA	NA	NA	NA	NA	NA	NA	Negative	No
0434	No	NA	Yes	No	8.33	Male	Yes	39.64	Purebred	Labrador Retriever	3.0	2.0	No	No	NA	NA	NA	NA	NA	NA	NA	NA	NA	NA	Negative	No
0435	No	NA	No	Yes	4.33	Female	Yes	6.73	Purebred	Australian Terrier	2.0	1.0	No	No	NA	NA	NA	NA	NA	NA	NA	NA	NA	NA	Negative	No
0436	No	NA	No	Yes	4.92	Female	Yes	22.27	Mixed-breed	NA	2.0	1.0	No	No	NA	NA	NA	NA	NA	NA	NA	NA	NA	NA	Negative	No
0437	No	NA	No	Yes	3.75	Female	Yes	29.64	Purebred	Bull Terrier	1.0	1.0	No	No	NA	NA	NA	NA	NA	NA	NA	NA	NA	NA	Negative	No
0438	No	NA	No	Yes	4.75	Male	Yes	13.55	Purebred	Australian Cattle Dog	3.0	1.0	No	No	NA	NA	NA	NA	NA	NA	NA	NA	NA	NA	Negative	No
0439	No	NA	No	Yes	1.33	Female	No	11.00	Purebred	Whippet	3.0	2.0	No	No	NA	NA	NA	NA	NA	NA	NA	NA	NA	NA	Negative	No
0440	No	NA	No	Yes	3.42	Female	Yes	10.55	Mixed-breed	NA	3.5	2.0	No	No	NA	NA	NA	NA	NA	NA	NA	NA	NA	NA	Negative	No
0441	No	NA	No	Yes	3.75	Male	Yes	25.77	Purebred	Australian Cattle Dog	4.0	2.0	No	No	NA	NA	NA	NA	NA	NA	NA	NA	NA	NA	Negative	No
0442	No	NA	No	Yes	14.00	Female	Yes	27.41	Mixed-breed	NA	1.5	1.0	No	No	NA	NA	NA	NA	NA	NA	NA	NA	NA	NA	Positive	No
0443	No	NA	No	Yes	2.00	Female	Yes	25.68	Mixed-breed	NA	1.0	1.0	No	No	NA	NA	NA	NA	NA	NA	NA	NA	NA	NA	Negative	No
0444	No	NA	Yes	No	7.00	Male	Yes	12.05	Mixed-breed	NA	2.5	1.5	No	No	NA	NA	NA	NA	NA	NA	NA	NA	NA	NA	Negative	No
0445	No	NA	Yes	No	1.50	Female	Yes	35.41	Purebred	Bloodhound	1.0	1.0	No	No	NA	NA	NA	NA	NA	NA	NA	NA	NA	NA	Negative	No
0446	No	NA	Yes	No	10.00	Female	Yes	30.27	Mixed-breed	NA	2.5	1.0	No	No	NA	NA	NA	NA	NA	NA	NA	NA	NA	NA	Negative	No
0447	No	NA	No	Yes	3.50	Female	Yes	28.27	Mixed-breed	NA	4.0	2.0	No	No	NA	NA	NA	NA	NA	NA	NA	NA	NA	NA	Negative	No
0448	No	NA	No	Yes	7.67	Female	Yes	27.91	Purebred	German Shepherd	3.0	1.0	No	No	NA	NA	NA	NA	NA	NA	NA	NA	NA	NA	Negative	No
0449	Yes	Laboratory Workflow Deviation	No	No	1.25	Male	Yes	29.73	Mixed-breed	NA	4.0	2.0	No	No	NA	NA	NA	NA	NA	NA	NA	NA	NA	NA	Negative	No
0450	No	NA	No	Yes	1.00	Female	No	7.73	Mixed-breed	NA	1.0	1.0	No	No	NA	NA	NA	NA	NA	NA	NA	NA	NA	NA	Negative	No
0451	No	NA	No	Yes	9.75	Male	No	34.18	Purebred	Labrador Retriever	0.5	1.0	No	No	NA	NA	NA	NA	NA	NA	NA	NA	NA	NA	Negative	No
0452	No	NA	No	Yes	11.25	Female	Yes	6.91	Mixed-breed	NA	2.0	2.0	No	No	NA	NA	NA	NA	NA	NA	NA	NA	NA	NA	Negative	No
0453	No	NA	Yes	No	12.67	Male	Yes	6.45	Purebred	Shih Tzu	1.0	1.0	No	No	NA	NA	NA	NA	NA	NA	NA	NA	NA	NA	Negative	No
0454	No	NA	No	Yes	2.50	Male	Yes	38.18	Mixed-breed	NA	1.0	1.0	No	No	NA	NA	NA	NA	NA	NA	NA	NA	NA	NA	Negative	No
0455	Yes	Laboratory Workflow Deviation	No	No	3.58	Female	Yes	28.09	Mixed-breed	NA	3.5	2.0	No	No	NA	NA	NA	NA	NA	NA	NA	NA	NA	NA	Negative	No
0456	No	NA	Yes	No	9.58	Male	Yes	29.82	Mixed-breed	NA	3.5	2.0	No	No	NA	NA	NA	NA	NA	NA	NA	NA	NA	NA	Negative	No
0457	Yes	Laboratory Workflow Deviation	No	No	11.83	Female	Yes	28.55	Purebred	Labrador Retriever	3.5	2.0	No	No	NA	NA	NA	NA	NA	NA	NA	NA	NA	NA	Negative	No
0458	No	NA	No	Yes	1.50	Male	Yes	34.32	Purebred	Labrador Retriever	2.5	2.0	No	No	NA	NA	NA	NA	NA	NA	NA	NA	NA	NA	Negative	No
0459	No	NA	No	Yes	3.92	Female	Yes	43.36	Purebred	German Shepherd	3.0	1.0	No	No	NA	NA	NA	NA	NA	NA	NA	NA	NA	NA	Negative	No
0460	No	NA	Yes	No	1.92	Female	No	34.50	Mixed-breed	NA	3.0	2.0	No	No	NA	NA	NA	NA	NA	NA	NA	NA	NA	NA	Negative	No
0461	No	NA	Yes	No	10.25	Female	Yes	34.27	Purebred	Golden Retriever	2.0	1.0	No	No	NA	NA	NA	NA	NA	NA	NA	NA	NA	NA	Positive	No
0462	No	NA	No	Yes	NA	Male	Yes	28.27	Mixed-breed	NA	2.0	1.0	No	No	NA	NA	NA	NA	NA	NA	NA	NA	NA	NA	Negative	No
0463	No	NA	No	Yes	4.67	Male	Yes	26.15	Mixed-breed	NA	1.0	1.0	No	No	NA	NA	NA	NA	NA	NA	NA	NA	NA	NA	Negative	No
0464	No	NA	No	Yes	6.00	Female	Yes	21.64	Mixed-breed	NA	1.5	1.0	No	No	NA	NA	NA	NA	NA	NA	NA	NA	NA	NA	Negative	No
0465	No	NA	No	Yes	3.00	Female	Yes	19.64	Mixed-breed	NA	1.5	2.0	No	No	NA	NA	NA	NA	NA	NA	NA	NA	NA	NA	Negative	No
0466	No	NA	No	Yes	4.50	Male	Yes	18.86	Purebred	Australian Shepherd	2.5	2.0	No	No	NA	NA	NA	NA	NA	NA	NA	NA	NA	NA	Negative	No
0467	No	NA	No	Yes	6.25	Male	Yes	24.36	Mixed-breed	NA	2.0	1.0	No	No	NA	NA	NA	NA	NA	NA	NA	NA	NA	NA	Negative	No
0468	No	NA	No	Yes	1.50	Male	Yes	40.64	Mixed-breed	NA	3.0	1.0	No	No	NA	NA	NA	NA	NA	NA	NA	NA	NA	NA	Negative	No
0469	No	NA	No	Yes	4.00	Male	Yes	32.73	Mixed-breed	NA	2.0	1.0	No	No	NA	NA	NA	NA	NA	NA	NA	NA	NA	NA	Negative	No
0470	No	NA	No	Yes	4.08	Male	No	22.23	Mixed-breed	NA	2.0	1.0	No	No	NA	NA	NA	NA	NA	NA	NA	NA	NA	NA	Negative	No
0471	No	NA	No	Yes	3.67	Female	Yes	30.27	Mixed-breed	NA	2.0	1.0	No	No	NA	NA	NA	NA	NA	NA	NA	NA	NA	NA	Negative	No
0472	No	NA	No	Yes	3.33	Male	Yes	44.36	Purebred	German Shepherd	3.5	2.0	No	No	NA	NA	NA	NA	NA	NA	NA	NA	NA	NA	Negative	No
0473	No	NA	No	Yes	5.08	Male	Yes	48.36	Purebred	Labrador Retriever	2.0	2.0	No	No	NA	NA	NA	NA	NA	NA	NA	NA	NA	NA	Negative	No
0474	No	NA	No	Yes	10.00	Female	Yes	31.91	Purebred	American Staffordshire Terrier	3.0	1.0	No	No	NA	NA	NA	NA	NA	NA	NA	NA	NA	NA	Negative	No
0475	No	NA	Yes	No	2.50	Female	Yes	42.55	Purebred	Golden Retriever	3.0	2.0	No	No	NA	NA	NA	NA	NA	NA	NA	NA	NA	NA	Negative	No
0476	No	NA	Yes	No	7.92	Male	Yes	32.47	Mixed-breed	NA	3.5	2.0	No	No	NA	NA	NA	NA	NA	NA	NA	NA	NA	NA	Negative	No
0477	No	NA	No	Yes	4.00	Female	Yes	30.73	Purebred	Golden Retriever	2.0	2.0	No	No	NA	NA	NA	NA	NA	NA	NA	NA	NA	NA	Negative	No
0478	No	NA	No	Yes	3.42	Male	Yes	32.97	Mixed-breed	NA	2.0	1.0	No	No	NA	NA	NA	NA	NA	NA	NA	NA	NA	NA	Negative	No
0479	No	NA	No	Yes	4.00	Male	Yes	29.00	Purebred	Golden Retriever	1.5	1.0	No	No	NA	NA	NA	NA	NA	NA	NA	NA	NA	NA	Negative	No
0480	No	NA	Yes	No	6.00	Female	Yes	20.23	Mixed-breed	NA	4.0	2.0	No	No	NA	NA	NA	NA	NA	NA	NA	NA	NA	NA	Negative	No
0481	No	NA	No	Yes	1.08	Male	Yes	21.54	Mixed-breed	NA	2.0	1.0	No	No	NA	NA	NA	NA	NA	NA	NA	NA	NA	NA	Negative	No
0482	No	NA	No	Yes	9.00	Female	Yes	7.72	Mixed-breed	NA	3.5	1.5	No	No	NA	NA	NA	NA	NA	NA	NA	NA	NA	NA	Negative	No
0483	No	NA	Yes	No	6.50	Male	Yes	13.36	Purebred	Beagle	1.0	0.5	No	No	NA	NA	NA	NA	NA	NA	NA	NA	NA	NA	Negative	No
0484	No	NA	No	Yes	5.75	Male	Yes	21.36	Mixed-breed	NA	2.0	1.5	No	No	NA	NA	NA	NA	NA	NA	NA	NA	NA	NA	Negative	No
0485	No	NA	No	Yes	2.75	Female	Yes	8.55	Mixed-breed	NA	1.5	0.5	No	No	NA	NA	NA	NA	NA	NA	NA	NA	NA	NA	Negative	No
0486	No	NA	No	Yes	2.17	Male	Yes	29.55	Mixed-breed	NA	1.0	0.0	No	No	NA	NA	NA	NA	NA	NA	NA	NA	NA	NA	Negative	No
0487	No	NA	No	Yes	1.25	Male	Yes	31.82	Mixed-breed	NA	2.0	1.0	No	No	NA	NA	NA	NA	NA	NA	NA	NA	NA	NA	Negative	No
0488	No	NA	Yes	No	7.25	Male	Yes	23.23	Purebred	Poodle, Standard	4.0	2.0	No	No	NA	NA	NA	NA	NA	NA	NA	NA	NA	NA	Negative	No
0489	No	NA	No	Yes	5.25	Male	Yes	45.91	Purebred	Labrador Retriever	2.0	1.0	No	No	NA	NA	NA	NA	NA	NA	NA	NA	NA	NA	Negative	No
0490	No	NA	No	Yes	4.00	Female	Yes	21.75	Purebred	Poodle, Standard	3.0	2.0	No	No	NA	NA	NA	NA	NA	NA	NA	NA	NA	NA	Negative	No
0491	No	NA	No	Yes	4.25	Female	Yes	22.23	Purebred	Poodle, Standard	1.0	1.0	No	No	NA	NA	NA	NA	NA	NA	NA	NA	NA	NA	Negative	No

S1 Table. Full subject level data for subjects enrolled in the CANDiD study (continued)

See legend on last page.

Subject	Excluded	Reason for Exclusion	Training Set	Testing Set	Age	Sex	Spay/ Neuter	Weight (kg)	Purebred or Mixed Breed	Breed (Purebred)	Hemolysis	Lipemia	Cancer Diagnosis at Enrollment	Cancer Diagnosed after Liquid Biopsy	Cancer Type	Immunophenotype (Lymphoid Cancer)	Extent of Disease: Non-Lymphoid Cancer	Extent of Disease: Lymphoid Cancer	Tumor Size	Cancer Type (Cancer #2)	Immunophenotype (Cancer #2 if Lymphoid)	Extent of Disease: Non-Lymphoid Cancer (Cancer #2)	Extent of Disease: Lymphoid Cancer (Cancer #2)	Tumor Size (Cancer #2)	Liquid Biopsy Result	CSO Prediction of Hematological Malignancy
0492	No	NA	No	Yes	1.33	Male	Yes	25.14	Purebred	Labrador Retriever	3.0	2.0	No	No	NA	NA	NA	NA	NA	NA	NA	NA	NA	NA	Negative	No
0493	No	NA	No	Yes	1.17	Male	Yes	47.73	Purebred	Pyrenean Shepherd	1.0	1.0	No	No	NA	NA	NA	NA	NA	NA	NA	NA	NA	NA	Negative	No
0494	No	NA	No	Yes	9.58	Female	Yes	14.23	Mixed-breed	NA	2.5	2.0	No	No	NA	NA	NA	NA	NA	NA	NA	NA	NA	NA	Negative	No
0495	No	NA	No	Yes	3.25	Female	Yes	18.82	Mixed-breed	NA	1.0	1.0	No	No	NA	NA	NA	NA	NA	NA	NA	NA	NA	NA	Negative	No
0496	No	NA	No	Yes	1.00	Male	Yes	13.73	Mixed-breed	NA	1.0	1.0	No	No	NA	NA	NA	NA	NA	NA	NA	NA	NA	NA	Negative	No
0497	No	NA	No	Yes	3.08	Male	No	41.59	Purebred	Labrador Retriever	2.0	1.0	No	No	NA	NA	NA	NA	NA	NA	NA	NA	NA	NA	Negative	No
0498	No	NA	No	Yes	3.08	Female	Yes	17.27	Purebred	Australian Cattle Dog	2.5	1.0	No	No	NA	NA	NA	NA	NA	NA	NA	NA	NA	NA	Negative	No
0499	No	NA	Yes	No	7.33	Male	Yes	28.64	Mixed-breed	NA	3.5	2.0	No	No	NA	NA	NA	NA	NA	NA	NA	NA	NA	NA	Positive	No
0500	No	NA	No	Yes	3.58	Female	Yes	29.36	Purebred	Golden Retriever	3.0	2.0	No	No	NA	NA	NA	NA	NA	NA	NA	NA	NA	NA	Negative	No
0501	No	NA	Yes	No	2.67	Female	Yes	34.73	Purebred	Golden Retriever	3.0	1.0	No	No	NA	NA	NA	NA	NA	NA	NA	NA	NA	NA	Negative	No
0502	No	NA	No	Yes	2.75	Male	Yes	38.91	Purebred	Golden Retriever	3.0	1.0	No	No	NA	NA	NA	NA	NA	NA	NA	NA	NA	NA	Negative	No
0503	No	NA	Yes	No	4.50	Female	No	34.00	Purebred	Golden Retriever	4.0	1.0	No	No	NA	NA	NA	NA	NA	NA	NA	NA	NA	NA	Negative	No
0504	No	NA	Yes	No	4.75	Female	Yes	31.59	Mixed-breed	NA	3.0	1.0	No	No	NA	NA	NA	NA	NA	NA	NA	NA	NA	NA	Negative	No
0505	No	NA	No	Yes	10.75	Male	Yes	39.45	Purebred	Golden Retriever	3.0	2.0	No	No	NA	NA	NA	NA	NA	NA	NA	NA	NA	NA	Negative	No
0506	No	NA	Yes	No	3.42	Female	Yes	24.05	Mixed-breed	NA	3.5	1.5	No	No	NA	NA	NA	NA	NA	NA	NA	NA	NA	NA	Negative	No
0507	No	NA	No	Yes	2.83	Female	No	24.55	Purebred	Golden Retriever	3.0	1.0	No	No	NA	NA	NA	NA	NA	NA	NA	NA	NA	NA	Negative	No
0508	No	NA	No	Yes	7.25	Female	Yes	31.00	Purebred	Golden Retriever	4.0	1.0	No	No	NA	NA	NA	NA	NA	NA	NA	NA	NA	NA	Negative	No
0509	No	NA	Yes	No	6.17	Female	Yes	20.36	Purebred	Golden Retriever	3.0	1.0	No	No	NA	NA	NA	NA	NA	NA	NA	NA	NA	NA	Negative	No
0510	No	NA	No	Yes	8.00	Male	Yes	39.86	Mixed-breed	NA	1.5	1.0	No	No	NA	NA	NA	NA	NA	NA	NA	NA	NA	NA	Negative	No
0511	No	NA	No	Yes	2.92	Female	No	42.00	Purebred	Rottweiler	1.5	NA	No	No	NA	NA	NA	NA	NA	NA	NA	NA	NA	NA	Negative	No
0512	No	NA	Yes	No	6.00	Male	No	31.82	Purebred	German Shepherd	2.0	1.0	No	No	NA	NA	NA	NA	NA	NA	NA	NA	NA	NA	Negative	No
0513	No	NA	No	Yes	6.42	Female	No	27.86	Purebred	German Shepherd	1.5	1.0	No	No	NA	NA	NA	NA	NA	NA	NA	NA	NA	NA	Negative	No
0514	No	NA	No	Yes	5.00	Male	Yes	30.32	Mixed-breed	NA	3.0	2.0	No	No	NA	NA	NA	NA	NA	NA	NA	NA	NA	NA	Negative	No
0515	No	NA	Yes	No	8.00	Male	Yes	61.36	Mixed-breed	NA	1.0	1.0	No	No	NA	NA	NA	NA	NA	NA	NA	NA	NA	NA	Negative	No
0516	No	NA	No	Yes	2.00	Male	No	24.09	Mixed-breed	NA	1.0	1.0	No	No	NA	NA	NA	NA	NA	NA	NA	NA	NA	NA	Negative	No
0517	No	NA	No	Yes	3.83	Male	Yes	22.41	Mixed-breed	NA	3.0	2.0	No	No	NA	NA	NA	NA	NA	NA	NA	NA	NA	NA	Negative	No
0518	No	NA	No	Yes	1.00	Male	Yes	17.73	Mixed-breed	NA	1.0	1.0	No	No	NA	NA	NA	NA	NA	NA	NA	NA	NA	NA	Negative	No
0519	No	NA	Yes	No	2.08	Female	Yes	29.41	Purebred	Labrador Retriever	3.0	1.5	No	No	NA	NA	NA	NA	NA	NA	NA	NA	NA	NA	Negative	No
0520	No	NA	No	Yes	2.58	Male	No	21.82	Purebred	Australian Cattle Dog	3.5	2.0	No	No	NA	NA	NA	NA	NA	NA	NA	NA	NA	NA	Negative	No
0521	No	NA	Yes	No	10.00	Male	Yes	7.45	Purebred	Poodle, Miniature	4.0	2.0	No	No	NA	NA	NA	NA	NA	NA	NA	NA	NA	NA	Negative	No
0522	No	NA	No	Yes	4.17	Male	Yes	25.91	Mixed-breed	NA	2.0	2.0	No	No	NA	NA	NA	NA	NA	NA	NA	NA	NA	NA	Negative	No
0523	No	NA	Yes	No	3.00	Male	No	40.82	Purebred	American Staffordshire Terrier	3.0	2.0	No	No	NA	NA	NA	NA	NA	NA	NA	NA	NA	NA	Negative	No
0524	No	NA	Yes	No	2.00	Male	Yes	35.45	Mixed-breed	NA	3.0	1.0	No	No	NA	NA	NA	NA	NA	NA	NA	NA	NA	NA	Negative	No
0525	No	NA	No	Yes	2.50	Male	Yes	27.05	Mixed-breed	NA	4.0	1.0	No	No	NA	NA	NA	NA	NA	NA	NA	NA	NA	NA	Negative	No
0526	No	NA	No	Yes	6.08	Female	Yes	49.55	Purebred	German Shepherd	3.0	2.0	No	No	NA	NA	NA	NA	NA	NA	NA	NA	NA	NA	Negative	No
0527	No	NA	Yes	No	10.00	Male	Yes	29.86	Mixed-breed	NA	3.0	1.0	No	No	NA	NA	NA	NA	NA	NA	NA	NA	NA	NA	Negative	No
0528	Yes	Laboratory Workflow Deviation	No	No	5.42	Male	Yes	34.55	Mixed-breed	NA	4.0	1.0	No	No	NA	NA	NA	NA	NA	NA	NA	NA	NA	NA	Negative	No
0529	No	NA	No	Yes	6.00	Female	Yes	39.82	Mixed-breed	NA	4.0	2.0	No	No	NA	NA	NA	NA	NA	NA	NA	NA	NA	NA	Negative	No
0530	No	NA	No	Yes	2.33	Female	Yes	64.55	Mixed-breed	NA	3.0	1.0	No	No	NA	NA	NA	NA	NA	NA	NA	NA	NA	NA	Negative	No
0531	No	NA	No	Yes	4.00	Male	Yes	38.27	Mixed-breed	NA	4.0	2.0	No	No	NA	NA	NA	NA	NA	NA	NA	NA	NA	NA	Negative	No
0532	No	NA	Yes	No	2.25	Male	No	42.27	Purebred	German Shepherd	3.0	1.0	No	No	NA	NA	NA	NA	NA	NA	NA	NA	NA	NA	Negative	No
0533	Yes	Test failure	No	No	6.00	Female	Yes	29.55	Purebred	Golden Retriever	3.0	1.0	No	No	NA	NA	NA	NA	NA	NA	NA	NA	NA	NA	Negative	No
0534	No	NA	No	Yes	8.08	Male	Yes	11.64	Mixed-breed	NA	3.0	1.0	No	No	NA	NA	NA	NA	NA	NA	NA	NA	NA	NA	Negative	No
0535	No	NA	No	Yes	3.25	Male	Yes	35.45	Mixed-breed	NA	4.0	2.0	No	No	NA	NA	NA	NA	NA	NA	NA	NA	NA	NA	Negative	No
0536	No	NA	No	Yes	2.83	Female	No	13.68	Mixed-breed	NA	3.5	2.0	No	No	NA	NA	NA	NA	NA	NA	NA	NA	NA	NA	Negative	No
0537	No	NA	No	Yes	2.33	Female	Yes	31.59	Purebred	Golden Retriever	3.5	2.0	No	No	NA	NA	NA	NA	NA	NA	NA	NA	NA	NA	Negative	No
0538	No	NA	No	Yes	6.50	Female	Yes	34.09	Purebred	Boxer	3.0	1.0	No	No	NA	NA	NA	NA	NA	NA	NA	NA	NA	NA	Negative	No
0539	No	NA	No	Yes	4.50	Male	Yes	43.89	Mixed-breed	NA	2.0	1.0	No	No	NA	NA	NA	NA	NA	NA	NA	NA	NA	NA	Negative	No
0540	No	NA	No	Yes	5.83	Male	Yes	37.68	Purebred	Golden Retriever	3.0	1.0	No	No	NA	NA	NA	NA	NA	NA	NA	NA	NA	NA	Negative	No
0541	No	NA	No	Yes	5.75	Female	Yes	10.22	Purebred	Chihuahua	3.5	2.0	No	No	NA	NA	NA	NA	NA	NA	NA	NA	NA	NA	Negative	No
0542	No	NA	No	Yes	1.25	Female	Yes	19.32	Mixed-breed	NA	4.0	2.0	No	No	NA	NA	NA	NA	NA	NA	NA	NA	NA	NA	Negative	No
0543	No	NA	Yes	No	7.17	Female	Yes	17.84	Purebred	English Bulldog	4.0	2.0	No	No	NA	NA	NA	NA	NA	NA	NA	NA	NA	NA	Negative	No
0544	No	NA	Yes	No	4.25	Female	Yes	33.86	Mixed-breed	NA	4.0	2.0	No	No	NA	NA	NA	NA	NA	NA	NA	NA	NA	NA	Negative	No
0545	No	NA	No	Yes	1.08	Male	Yes	22.05	Mixed-breed	NA	2.0	1.0	No	No	NA	NA	NA	NA	NA	NA	NA	NA	NA	NA	Negative	No
0546	No	NA	No	Yes	5.00	Female	Yes	21.18	Mixed-breed	NA	4.0	2.0	No	No	NA	NA	NA	NA	NA	NA	NA	NA	NA	NA	Negative	No
0547	No	NA	No	Yes	8.17	Female	Yes	36.45	Purebred	Weimaraner	1.0	1.0	No	No	NA	NA	NA	NA	NA	NA	NA	NA	NA	NA	Negative	No
0548	No	NA	No	Yes	8.92	Female	Yes	31.95	Purebred	Labrador Retriever	4.0	1.0	No	No	NA	NA	NA	NA	NA	NA	NA	NA	NA	NA	Negative	No
0549	No	NA	No	Yes	14.75	Male	Yes	16.05	Mixed-breed	NA	0.0	0.0	No	No	NA	NA	NA	NA	NA	NA	NA	NA	NA	NA	Negative	No
0550	No	NA	No	Yes	4.58	Male	No	41.18	Purebred	Rhodesian Ridgeback	3.5	2.0	No	No	NA	NA	NA	NA	NA	NA	NA	NA	NA	NA	Negative	No
0551	No	NA	No	Yes	5.00	Female	Yes	27.50	Purebred	Border Collie	3.0	2.0	No	No	NA	NA	NA	NA	NA	NA	NA	NA	NA	NA	Negative	No
0552	No	NA	No	Yes	2.92	Male	Yes	47.18	Purebred	Rottweiler	1.5	1.0	No	No	NA	NA	NA	NA	NA	NA	NA	NA	NA	NA	Negative	No
0553	No	NA	No	Yes	2.50	Female	No	37.64	Purebred	Labrador Retriever	0.5	1.0	No	No	NA	NA	NA	NA	NA	NA	NA	NA	NA	NA	Negative	No
0554	Yes	Laboratory Workflow Deviation	No	No	3.67	Female	Yes	21.05	Mixed-breed	NA	4.0	1.0	No	No	NA	NA	NA	NA	NA	NA	NA	NA	NA	NA	Negative	No
0555	No	NA	No	Yes	2.25	Female	Yes	16.82	Purebred	English Springer Spaniel	3.0	1.0	No	No	NA	NA	NA	NA	NA	NA	NA	NA	NA	NA	Negative	No
0556	No	NA	No	Yes	2.33	Male	Yes	20.45	Purebred	Australian Shepherd	1.5	2.0	No	No	NA	NA	NA	NA	NA	NA	NA	NA	NA	NA	Negative	No
0557	No	NA	No	Yes	3.67	Male	Yes	30.64	Mixed-breed	NA	4.0	2.0	No	No	NA	NA	NA	NA	NA	NA	NA	NA	NA	NA	Negative	No
0558	No	NA	Yes	No	6.83	Male	Yes	18.41	Mixed-breed	NA	1.0	1.0	No	No	NA	NA	NA	NA	NA	NA	NA	NA	NA	NA	Negative	No
0559	No	NA	No	Yes	4.67	Female	Yes	38.32	Purebred	German Wirehaired Pointer	3.0	1.0	No	No	NA	NA	NA	NA	NA	NA	NA	NA	NA	NA	Negative	No
0560	Yes	Laboratory Workflow Deviation	No	No	7.17	Female	Yes	26.55	Mixed-breed	NA	4.0	2.0	No	No	NA	NA	NA	NA	NA	NA	NA	NA	NA	NA	Negative	No
0561	No	NA	Yes	No	7.83	Male	Yes	25.73	Purebred	English Bulldog	4.0	NA	No	No	NA	NA	NA	NA	NA	NA	NA	NA	NA	NA	Negative	No

Subject	Excluded	Reason for Exclusion	Training Set	Testing Set	Age	Sex	Spay/ Neuter	Weight (kg)	Purebred or Mixed Breed	Breed (Purebred)	Hemolysis	Lipemia	Cancer Diagnosis at Enrollment	Cancer Diagnosed after Liquid Biopsy	Cancer Type	Immunophenotype (Lymphoid Cancer)	Extent of Disease: Non-Lymphoid Cancer	Extent of Disease: Lymphoid Cancer	Tumor Size	Cancer Type (Cancer #2)	Immunophenotype (Cancer #2 if Lymphoid)	Extent of Disease: Non-Lymphoid Cancer (Cancer #2)	Extent of Disease: Lymphoid Cancer (Cancer #2)	Tumor Size (Cancer #2)	Liquid Biopsy Result	CSO Prediction of Hematological Malignancy
0562	No	NA	No	Yes	1.67	Male	Yes	32.59	Mixed-breed	NA	3.0	0.0	No	No	NA	NA	NA	NA	NA	NA	NA	NA	NA	NA	Negative	No
0563	No	NA	Yes	No	3.58	Female	Yes	23.91	Mixed-breed	NA	4.0	2.0	No	No	NA	NA	NA	NA	NA	NA	NA	NA	NA	NA	Negative	No
0564	Yes	Laboratory Workflow Deviation	No	No	12.83	Female	Yes	26.32	Mixed-breed	NA	4.0	1.0	No	No	NA	NA	NA	NA	NA	NA	NA	NA	NA	NA	Negative	No
0565	No	NA	No	Yes	2.50	Male	Yes	25.68	Purebred	Siberian Husky	4.0	1.0	No	No	NA	NA	NA	NA	NA	NA	NA	NA	NA	NA	Negative	No
0566	No	NA	Yes	No	4.08	Female	Yes	20.55	Mixed-breed	NA	3.0	0.5	No	No	NA	NA	NA	NA	NA	NA	NA	NA	NA	NA	Negative	No
0567	No	NA	No	Yes	4.17	Female	Yes	36.14	Mixed-breed	NA	3.0	0.0	No	No	NA	NA	NA	NA	NA	NA	NA	NA	NA	NA	Negative	No
0568	No	NA	No	Yes	1.17	Male	No	12.53	Purebred	French Bulldog	4.0	2.0	No	No	NA	NA	NA	NA	NA	NA	NA	NA	NA	NA	Negative	No
0569	No	NA	No	Yes	1.83	Male	Yes	27.96	Purebred	Labrador Retriever	4.0	2.0	No	No	NA	NA	NA	NA	NA	NA	NA	NA	NA	NA	Negative	No
0570	Yes	Test failure	No	No	1.50	Male	No	34.97	Mixed-breed	NA	4.0	2.0	No	No	NA	NA	NA	NA	NA	NA	NA	NA	NA	NA	Positive	No
0571	Yes	Laboratory Workflow Deviation	No	No	5.17	Female	Yes	45.91	Purebred	Doberman Pinscher	4.0	2.0	No	No	NA	NA	NA	NA	NA	NA	NA	NA	NA	NA	Negative	No
0572	No	NA	No	Yes	1.17	Male	Yes	21.18	Mixed-breed	NA	3.5	1.5	No	No	NA	NA	NA	NA	NA	NA	NA	NA	NA	NA	Negative	No
0573	Yes	Test failure	No	No	6.00	Female	Yes	33.18	Mixed-breed	NA	3.0	2.0	No	No	NA	NA	NA	NA	NA	NA	NA	NA	NA	NA	Negative	No
0574	Yes	Laboratory Workflow Deviation	No	No	2.00	Male	Yes	29.27	Mixed-breed	NA	3.5	2.0	No	No	NA	NA	NA	NA	NA	NA	NA	NA	NA	NA	Negative	No
0575	No	NA	Yes	No	6.33	Male	Yes	18.64	Mixed-breed	NA	4.0	2.0	No	No	NA	NA	NA	NA	NA	NA	NA	NA	NA	NA	Negative	No
0576	No	NA	No	Yes	3.25	Male	Yes	24.86	Purebred	Poodle, Standard	2.5	2.0	No	No	NA	NA	NA	NA	NA	NA	NA	NA	NA	NA	Negative	No
0577	No	NA	Yes	No	10.67	Male	Yes	16.00	Mixed-breed	NA	3.0	2.0	No	No	NA	NA	NA	NA	NA	NA	NA	NA	NA	NA	Negative	No
0578	No	NA	No	Yes	4.00	Male	Yes	32.73	Purebred	Boxer	4.0	2.0	No	No	NA	NA	NA	NA	NA	NA	NA	NA	NA	NA	Negative	No
0579	No	NA	No	Yes	5.08	Female	Yes	47.27	Mixed-breed	NA	4.0	2.0	No	No	NA	NA	NA	NA	NA	NA	NA	NA	NA	NA	Negative	No
0580	No	NA	Yes	No	6.67	Female	Yes	27.45	Mixed-breed	NA	3.5	2.0	No	No	NA	NA	NA	NA	NA	NA	NA	NA	NA	NA	Negative	No
0581	No	NA	No	Yes	2.50	Male	Yes	46.18	Purebred	Rottweiler	2.0	0.0	No	No	NA	NA	NA	NA	NA	NA	NA	NA	NA	NA	Negative	No
0582	Yes	Laboratory Workflow Deviation	No	No	6.67	Female	Yes	25.64	Mixed-breed	NA	4.0	NA	No	No	NA	NA	NA	NA	NA	NA	NA	NA	NA	NA	Negative	No
0583	No	NA	No	Yes	8.50	Male	Yes	33.18	Mixed-breed	NA	3.0	1.0	No	No	NA	NA	NA	NA	NA	NA	NA	NA	NA	NA	Negative	No
0584	No	NA	No	Yes	15.00	Female	Yes	7.64	Purebred	Rat Terrier	3.0	2.0	No	No	NA	NA	NA	NA	NA	NA	NA	NA	NA	NA	Negative	No
0585	No	NA	No	Yes	11.50	Female	Yes	39.64	Mixed-breed	NA	4.0	1.0	No	No	NA	NA	NA	NA	NA	NA	NA	NA	NA	NA	Negative	No
0586	No	NA	No	Yes	5.50	Female	Yes	52.27	Purebred	Great Dane	4.0	1.0	No	No	NA	NA	NA	NA	NA	NA	NA	NA	NA	NA	Negative	No
0587	No	NA	No	Yes	1.00	Male	No	28.00	Mixed-breed	NA	0.0	1.0	No	No	NA	NA	NA	NA	NA	NA	NA	NA	NA	NA	Negative	No
0588	No	NA	No	Yes	5.08	Male	No	62.73	Mixed-breed	NA	1.0	1.0	No	No	NA	NA	NA	NA	NA	NA	NA	NA	NA	NA	Negative	No
0589	Yes	Test failure	No	No	9.00	Female	Yes	19.73	Mixed-breed	NA	3.5	0.0	No	No	NA	NA	NA	NA	NA	NA	NA	NA	NA	NA	Fail	No
0590	No	NA	No	Yes	1.50	Female	No	21.82	Mixed-breed	NA	0.5	1.0	No	No	NA	NA	NA	NA	NA	NA	NA	NA	NA	NA	Negative	No
0591	No	NA	No	Yes	11.75	Female	Yes	32.95	Purebred	Labrador Retriever	2.5	0.5	No	No	NA	NA	NA	NA	NA	NA	NA	NA	NA	NA	Negative	No
0592	No	NA	No	Yes	4.50	Female	Yes	23.09	Mixed-breed	NA	4.0	2.0	No	No	NA	NA	NA	NA	NA	NA	NA	NA	NA	NA	Negative	No
0593	No	NA	Yes	No	4.67	Female	No	26.73	Mixed-breed	NA	2.0	0.0	No	No	NA	NA	NA	NA	NA	NA	NA	NA	NA	NA	Negative	No
0594	No	NA	No	Yes	4.33	Male	Yes	31.73	Purebred	Golden Retriever	2.5	1.0	No	No	NA	NA	NA	NA	NA	NA	NA	NA	NA	NA	Negative	No
0595	No	NA	No	Yes	2.00	Male	No	25.18	Mixed-breed	NA	4.0	NA	No	No	NA	NA	NA	NA	NA	NA	NA	NA	NA	NA	Negative	No
0596	Yes	Laboratory Workflow Deviation	No	No	4.67	Male	Yes	41.14	Mixed-breed	NA	4.0	1.0	No	No	NA	NA	NA	NA	NA	NA	NA	NA	NA	NA	Negative	No
0597	No	NA	No	Yes	4.00	Male	No	25.45	Purebred	Siberian Husky	3.0	2.0	No	No	NA	NA	NA	NA	NA	NA	NA	NA	NA	NA	Negative	No
0598	No	NA	Yes	No	1.50	Male	Yes	27.55	Purebred	German Shorthaired Pointer	3.0	2.0	No	No	NA	NA	NA	NA	NA	NA	NA	NA	NA	NA	Negative	No
0599	No	NA	No	Yes	10.25	Male	No	53.82	Mixed-breed	NA	4.0	2.0	No	No	NA	NA	NA	NA	NA	NA	NA	NA	NA	NA	Negative	No
0600	No	NA	No	Yes	8.50	Male	Yes	12.41	Purebred	Australian Shepherd	3.0	2.0	No	No	NA	NA	NA	NA	NA	NA	NA	NA	NA	NA	Negative	No
0601	No	NA	No	Yes	6.67	Female	Yes	38.18	Purebred	Staffordshire Bull Terrier	4.0	1.0	No	No	NA	NA	NA	NA	NA	NA	NA	NA	NA	NA	Negative	No
0602	No	NA	No	Yes	3.92	Female	Yes	33.47	Mixed-breed	NA	3.0	2.0	No	No	NA	NA	NA	NA	NA	NA	NA	NA	NA	NA	Negative	No
0603	No	NA	No	Yes	7.67	Female	Yes	26.56	Mixed-breed	NA	3.0	NA	No	No	NA	NA	NA	NA	NA	NA	NA	NA	NA	NA	Negative	No
0604	Yes	Enrollment or Clinical Deviation	No	No	5.08	Female	Yes	10.59	Purebred	Boxer	4.0	2.0	No	No	NA	NA	NA	NA	NA	NA	NA	NA	NA	NA	Negative	No
0605	No	NA	No	Yes	9.00	Female	Yes	28.32	Mixed-breed	NA	4.0	2.0	No	No	NA	NA	NA	NA	NA	NA	NA	NA	NA	NA	Negative	No
0606	No	NA	No	Yes	5.08	Male	Yes	46.82	Purebred	German Shepherd	3.5	2.0	No	No	NA	NA	NA	NA	NA	NA	NA	NA	NA	NA	Negative	No
0607	No	NA	No	Yes	5.58	Female	Yes	17.09	Mixed-breed	NA	2.0	1.0	No	No	NA	NA	NA	NA	NA	NA	NA	NA	NA	NA	Negative	No
0608	No	NA	Yes	No	10.00	Female	Yes	8.45	Mixed-breed	NA	4.0	2.0	No	No	NA	NA	NA	NA	NA	NA	NA	NA	NA	NA	Negative	No
0609	No	NA	No	Yes	1.33	Female	No	21.82	Mixed-breed	NA	1.0	1.0	No	No	NA	NA	NA	NA	NA	NA	NA	NA	NA	NA	Negative	No
0610	Yes	Laboratory Workflow Deviation	No	No	4.67	Female	Yes	29.73	Purebred	German Wirehaired Pointer	3.0	1.0	No	No	NA	NA	NA	NA	NA	NA	NA	NA	NA	NA	Negative	No
0611	No	NA	No	Yes	1.75	Male	No	25.41	Purebred	Golden Retriever	4.0	2.0	No	No	NA	NA	NA	NA	NA	NA	NA	NA	NA	NA	Negative	No
0612	No	NA	No	Yes	3.17	Female	Yes	31.05	Mixed-breed	NA	4.0	2.0	No	No	NA	NA	NA	NA	NA	NA	NA	NA	NA	NA	Negative	No
0613	No	NA	No	Yes	6.92	Female	Yes	35.00	Mixed-breed	NA	4.0	2.0	No	No	NA	NA	NA	NA	NA	NA	NA	NA	NA	NA	Negative	No
0614	Yes	Laboratory Workflow Deviation	No	No	10.50	Female	Yes	15.41	Mixed-breed	NA	2.5	2.0	No	No	NA	NA	NA	NA	NA	NA	NA	NA	NA	NA	Negative	No
0615	No	NA	No	Yes	3.50	Female	Yes	28.91	Purebred	German Shepherd	2.0	1.0	No	No	NA	NA	NA	NA	NA	NA	NA	NA	NA	NA	Negative	No
0616	Yes	Test failure	No	No	7.17	Male	Yes	49.73	Purebred	Pyrenean Shepherd	4.0	2.0	No	No	NA	NA	NA	NA	NA	NA	NA	NA	NA	NA	Negative	No
0617	No	NA	Yes	No	7.33	Male	Yes	35.64	Mixed-breed	NA	4.0	NA	No	No	NA	NA	NA	NA	NA	NA	NA	NA	NA	NA	Negative	No
0618	Yes	Test failure	No	No	8.75	Female	Yes	35.55	Purebred	Labrador Retriever	4.0	NA	No	No	NA	NA	NA	NA	NA	NA	NA	NA	NA	NA	Negative	No
0619	Yes	Test failure	No	No	1.00	Female	Yes	21.64	Purebred	Golden Retriever	4.0	NA	No	No	NA	NA	NA	NA	NA	NA	NA	NA	NA	NA	Negative	No
0620	No	NA	No	Yes	6.50	Male	Yes	33.82	Purebred	Golden Retriever	2.0	1.0	No	No	NA	NA	NA	NA	NA	NA	NA	NA	NA	NA	Negative	No
0621	Yes	Test failure	No	No	2.08	Female	Yes	19.14	Purebred	Vizslas	4.0	NA	No	No	NA	NA	NA	NA	NA	NA	NA	NA	NA	NA	Negative	No
0622	Yes	Test failure	No	No	4.42	Male	Yes	33.82	Purebred	Labrador Retriever	4.0	2.0	No	No	NA	NA	NA	NA	NA	NA	NA	NA	NA	NA	Negative	No
0623	No	NA	Yes	No	6.33	Male	Yes	43.45	Purebred	Labrador Retriever	4.0	NA	No	No	NA	NA	NA	NA	NA	NA	NA	NA	NA	NA	Positive	No
0624	No	NA	Yes	No	7.33	Male	Yes	42.91	Purebred	Labrador Retriever	4.0	1.5	No	No	NA	NA	NA	NA	NA	NA	NA	NA	NA	NA	Negative	No
0625	Yes	Test failure	No	No	4.50	Female	Yes	36.36	Purebred	Bernese Mountain Dogs	4.0	NA	No	No	NA	NA	NA	NA	NA	NA	NA	NA	NA	NA	Negative	No
0626	No	NA	No	Yes	3.67	Male	Yes	46.82	Mixed-breed	NA	4.0	2.0	No	No	NA	NA	NA	NA	NA	NA	NA	NA	NA	NA	Negative	No
0627	No	NA	Yes	No	3.50	Female	Yes	21.73	Mixed-breed	NA	2.5	2.0	No	No	NA	NA	NA	NA	NA	NA	NA	NA	NA	NA	Negative	No
0628	No	NA	No	Yes	1.25	Female	No	17.95	Mixed-breed	NA	4.0	2.0	No	No	NA	NA	NA	NA	NA	NA	NA	NA	NA	NA	Negative	No

Subject	Excluded	Reason for Exclusion	Training Set	Testing Set	Age	Sex	Spay/ Neuter	Weight (kg)	Purebred or Mixed Breed	Breed (Purebred)	Hemolysis	Lipemia	Cancer Diagnosis at Enrollment	Cancer Diagnosed after Liquid Biopsy	Cancer Type	Immunophenotype (Lymphoid Cancer)	Extent of Disease: Non-Lymphoid Cancer	Extent of Disease: Lymphoid Cancer	Tumor Size	Cancer Type (Cancer #2)	Immunophenotype (Cancer #2 if Lymphoid)	Extent of Disease: Non-Lymphoid Cancer (Cancer #2)	Extent of Disease: Lymphoid Cancer (Cancer #2)	Tumor Size (Cancer #2)	Liquid Biopsy Result	CSO Prediction of Hematological Malignancy
0629	No	NA	No	Yes	10.00	Male	Yes	19.55	Mixed-breed	NA	1.0	0.0	No	No	NA	NA	NA	NA	NA	NA	NA	NA	NA	NA	Negative	No
0630	No	NA	No	Yes	5.25	Male	Yes	19.50	Mixed-breed	NA	3.0	0.0	No	No	NA	NA	NA	NA	NA	NA	NA	NA	NA	NA	Negative	No
0631	No	NA	Yes	No	6.42	Male	No	16.00	Purebred	Brittany	4.0	2.0	No	No	NA	NA	NA	NA	NA	NA	NA	NA	NA	NA	Negative	No
0632	Yes	Laboratory Workflow Deviation	No	No	2.67	Female	Yes	51.55	Purebred	Mastiff	4.0	1.0	No	No	NA	NA	NA	NA	NA	NA	NA	NA	NA	NA	Negative	No
0633	No	NA	Yes	No	10.33	Male	Yes	12.36	Mixed-breed	NA	3.0	0.0	No	No	NA	NA	NA	NA	NA	NA	NA	NA	NA	NA	Negative	No
0634	No	NA	No	Yes	4.58	Female	Yes	49.09	Purebred	German Shepherd	3.0	0.0	No	No	NA	NA	NA	NA	NA	NA	NA	NA	NA	NA	Negative	No
0635	No	NA	No	Yes	3.58	Female	Yes	20.68	Mixed-breed	NA	3.5	1.0	No	No	NA	NA	NA	NA	NA	NA	NA	NA	NA	NA	Negative	No
0636	No	NA	Yes	No	9.50	Male	Yes	35.77	Purebred	Labrador Retriever	3.0	2.0	No	No	NA	NA	NA	NA	NA	NA	NA	NA	NA	NA	Negative	No
0637	No	NA	No	Yes	10.00	Male	Yes	34.09	Mixed-breed	NA	4.0	1.0	No	No	NA	NA	NA	NA	NA	NA	NA	NA	NA	NA	Negative	No
0638	Yes	Laboratory Workflow Deviation	No	No	6.67	Female	Yes	20.34	Mixed-breed	NA	3.5	1.5	No	No	NA	NA	NA	NA	NA	NA	NA	NA	NA	NA	Negative	No
0639	No	NA	No	Yes	13.50	Female	Yes	16.73	Purebred	Basenjis	3.0	0.0	No	No	NA	NA	NA	NA	NA	NA	NA	NA	NA	NA	Negative	No
0640	No	NA	No	Yes	3.83	Female	Yes	29.26	Mixed-breed	NA	2.0	0.0	No	No	NA	NA	NA	NA	NA	NA	NA	NA	NA	NA	Negative	No
0641	No	NA	No	Yes	1.50	Male	Yes	22.25	Mixed-breed	NA	3.0	0.0	No	No	NA	NA	NA	NA	NA	NA	NA	NA	NA	NA	Negative	No
0642	No	NA	Yes	No	1.00	Female	Yes	19.14	Mixed-breed	NA	2.5	2.0	No	No	NA	NA	NA	NA	NA	NA	NA	NA	NA	NA	Negative	No
0643	No	NA	Yes	No	10.67	Male	Yes	19.44	Mixed-breed	NA	3.0	2.0	No	No	NA	NA	NA	NA	NA	NA	NA	NA	NA	NA	Negative	No
0644	No	NA	No	Yes	10.25	Female	Yes	38.64	Mixed-breed	NA	2.0	2.0	No	No	NA	NA	NA	NA	NA	NA	NA	NA	NA	NA	Negative	No
0645	No	NA	No	Yes	9.58	Female	Yes	28.46	Purebred	Shetland Sheepdog	4.0	1.0	No	No	NA	NA	NA	NA	NA	NA	NA	NA	NA	NA	Negative	No
0646	No	NA	No	Yes	2.17	Male	No	25.55	Mixed-breed	NA	2.0	1.0	No	No	NA	NA	NA	NA	NA	NA	NA	NA	NA	NA	Negative	No
0647	No	NA	No	Yes	5.83	Female	Yes	39.28	Mixed-breed	NA	4.0	2.0	No	No	NA	NA	NA	NA	NA	NA	NA	NA	NA	NA	Negative	No
0648	No	NA	Yes	No	4.25	Male	No	27.27	Mixed-breed	NA	4.0	1.0	No	No	NA	NA	NA	NA	NA	NA	NA	NA	NA	NA	Negative	No
0649	No	NA	No	Yes	2.75	Female	Yes	21.65	Mixed-breed	NA	1.0	1.0	No	No	NA	NA	NA	NA	NA	NA	NA	NA	NA	NA	Negative	No
0650	No	NA	No	Yes	5.00	Male	Yes	26.25	Mixed-breed	NA	3.5	2.0	No	No	NA	NA	NA	NA	NA	NA	NA	NA	NA	NA	Negative	No
0651	No	NA	Yes	No	6.50	Female	Yes	35.57	Purebred	Akita	3.0	2.0	No	No	NA	NA	NA	NA	NA	NA	NA	NA	NA	NA	Negative	No
0652	No	NA	No	Yes	1.42	Male	Yes	32.07	Purebred	Australian Shepherd	3.5	2.0	No	No	NA	NA	NA	NA	NA	NA	NA	NA	NA	NA	Negative	No
0653	No	NA	Yes	No	5.00	Male	Yes	11.32	Mixed-breed	NA	4.0	1.0	No	No	NA	NA	NA	NA	NA	NA	NA	NA	NA	NA	Negative	No
0654	No	NA	Yes	No	4.50	Male	Yes	30.45	Mixed-breed	NA	1.5	0.0	No	No	NA	NA	NA	NA	NA	NA	NA	NA	NA	NA	Negative	No
0655	No	NA	Yes	No	3.33	Female	No	28.96	Mixed-breed	NA	3.5	2.0	No	No	NA	NA	NA	NA	NA	NA	NA	NA	NA	NA	Negative	No
0656	Yes	Test failure	No	No	2.75	Female	Yes	22.25	Mixed-breed	NA	4.0	2.0	No	No	NA	NA	NA	NA	NA	NA	NA	NA	NA	NA	Negative	No
0657	No	NA	No	Yes	2.25	Male	Yes	42.59	Purebred	Golden Retriever	2.0	1.0	No	No	NA	NA	NA	NA	NA	NA	NA	NA	NA	NA	Negative	No
0658	No	NA	No	Yes	6.67	Male	Yes	11.32	Purebred	Dachshund	4.0	2.0	No	No	NA	NA	NA	NA	NA	NA	NA	NA	NA	NA	Negative	No
0659	No	NA	No	Yes	1.00	Male	No	12.83	Mixed-breed	NA	3.0	0.0	No	No	NA	NA	NA	NA	NA	NA	NA	NA	NA	NA	Negative	No
0660	No	NA	No	Yes	4.17	Male	No	32.47	Purebred	Dogues de Bordeaux	3.0	0.0	No	No	NA	NA	NA	NA	NA	NA	NA	NA	NA	NA	Negative	No
0661	No	NA	No	Yes	4.00	Male	Yes	47.30	Purebred	German Shepherd	4.0	2.0	No	No	NA	NA	NA	NA	NA	NA	NA	NA	NA	NA	Negative	No
0662	No	NA	No	Yes	1.58	Male	Yes	36.28	Purebred	Boxer	3.0	1.0	No	No	NA	NA	NA	NA	NA	NA	NA	NA	NA	NA	Negative	No
0663	No	NA	No	Yes	3.42	Male	No	29.06	Mixed-breed	NA	4.0	2.0	No	No	NA	NA	NA	NA	NA	NA	NA	NA	NA	NA	Negative	No
0664	No	NA	No	Yes	4.00	Male	Yes	55.12	Purebred	Great Dane	4.0	2.0	No	No	NA	NA	NA	NA	NA	NA	NA	NA	NA	NA	Negative	No
0665	No	NA	No	Yes	8.00	Male	Yes	11.22	Mixed-breed	NA	2.0	1.0	No	No	NA	NA	NA	NA	NA	NA	NA	NA	NA	NA	Negative	No
0666	No	NA	No	Yes	3.33	Male	Yes	39.48	Mixed-breed	NA	3.0	2.0	No	No	NA	NA	NA	NA	NA	NA	NA	NA	NA	NA	Negative	No
0667	No	NA	No	Yes	3.17	Female	Yes	30.56	Mixed-breed	NA	3.0	2.0	No	No	NA	NA	NA	NA	NA	NA	NA	NA	NA	NA	Negative	No
0668	No	NA	No	Yes	1.17	Male	No	25.95	Mixed-breed	NA	4.0	2.0	No	No	NA	NA	NA	NA	NA	NA	NA	NA	NA	NA	Negative	No
0669	No	NA	Yes	No	7.00	Female	Yes	21.45	Mixed-breed	NA	4.0	1.5	No	No	NA	NA	NA	NA	NA	NA	NA	NA	NA	NA	Negative	No
0670	No	NA	No	Yes	1.17	Female	Yes	39.98	Purebred	Bernese Mountain Dogs	3.0	1.0	No	No	NA	NA	NA	NA	NA	NA	NA	NA	NA	NA	Negative	No
0671	No	NA	No	Yes	4.00	Female	Yes	25.45	Purebred	German Shepherd	3.0	1.5	No	No	NA	NA	NA	NA	NA	NA	NA	NA	NA	NA	Negative	No
0672	No	NA	No	Yes	2.00	Male	Yes	28.64	Mixed-breed	NA	2.0	0.0	No	No	NA	NA	NA	NA	NA	NA	NA	NA	NA	NA	Negative	No
0673	No	NA	No	Yes	3.17	Female	Yes	31.06	Purebred	German Shepherd	3.0	2.0	No	No	NA	NA	NA	NA	NA	NA	NA	NA	NA	NA	Negative	No
0674	Yes	Laboratory Workflow Deviation	No	No	1.17	Male	No	29.76	Purebred	Doberman Pinscher	4.0	2.0	No	No	NA	NA	NA	NA	NA	NA	NA	NA	NA	NA	Negative	No
0675	No	NA	No	Yes	1.50	Male	No	25.05	Mixed-breed	NA	4.0	1.0	No	No	NA	NA	NA	NA	NA	NA	NA	NA	NA	NA	Negative	No
0676	Yes	Laboratory Workflow Deviation	No	No	6.17	Female	Yes	27.96	Mixed-breed	NA	4.0	2.0	No	No	NA	NA	NA	NA	NA	NA	NA	NA	NA	NA	Negative	No
0677	No	NA	Yes	No	3.58	Male	Yes	30.06	Mixed-breed	NA	4.0	2.0	No	No	NA	NA	NA	NA	NA	NA	NA	NA	NA	NA	Negative	No
0678	No	NA	Yes	No	2.08	Female	No	32.37	Purebred	German Shepherd	4.0	2.0	No	No	NA	NA	NA	NA	NA	NA	NA	NA	NA	NA	Negative	No
0679	Yes	Enrollment or Clinical Deviation	No	No	4.67	Female	Yes	39.18	Purebred	Bernese Mountain Dogs	4.0	2.0	No	No	NA	NA	NA	NA	NA	NA	NA	NA	NA	NA	Negative	No
0680	No	NA	Yes	No	10.42	Male	Yes	66.84	Purebred	Akita	4.0	2.0	No	No	NA	NA	NA	NA	NA	NA	NA	NA	NA	NA	Negative	No
0681	No	NA	No	Yes	3.17	Female	Yes	26.56	Mixed-breed	NA	2.0	1.0	No	No	NA	NA	NA	NA	NA	NA	NA	NA	NA	NA	Negative	No
0682	No	NA	No	Yes	1.00	Male	Yes	38.28	Purebred	Golden Retriever	4.0	2.0	No	No	NA	NA	NA	NA	NA	NA	NA	NA	NA	NA	Negative	No
0683	No	NA	Yes	No	1.25	Male	Yes	30.96	Purebred	Labrador Retriever	3.0	1.0	No	No	NA	NA	NA	NA	NA	NA	NA	NA	NA	NA	Negative	No
0684	No	NA	Yes	No	4.17	Male	Yes	36.48	Purebred	Samoyed	4.0	NA	No	No	NA	NA	NA	NA	NA	NA	NA	NA	NA	NA	Negative	No
0685	No	NA	No	Yes	2.00	Male	Yes	33.57	Mixed-breed	NA	3.5	1.0	No	No	NA	NA	NA	NA	NA	NA	NA	NA	NA	NA	Negative	No
0686	Yes	Laboratory Workflow Deviation	No	No	3.00	Male	Yes	29.16	Mixed-breed	NA	4.0	2.0	No	No	NA	NA	NA	NA	NA	NA	NA	NA	NA	NA	Negative	No
0687	Yes	Laboratory Workflow Deviation	No	No	1.33	Female	No	26.05	Purebred	Labrador Retriever	4.0	2.0	No	No	NA	NA	NA	NA	NA	NA	NA	NA	NA	NA	Positive	No
0688	No	NA	No	Yes	2.25	Female	Yes	34.67	Mixed-breed	NA	3.0	1.0	No	No	NA	NA	NA	NA	NA	NA	NA	NA	NA	NA	Negative	No
0689	No	NA	No	Yes	2.33	Female	Yes	35.67	Mixed-breed	NA	4.0	2.0	No	No	NA	NA	NA	NA	NA	NA	NA	NA	NA	NA	Negative	No
0690	No	NA	No	Yes	2.00	Female	Yes	16.73	Mixed-breed	NA	3.0	2.0	No	No	NA	NA	NA	NA	NA	NA	NA	NA	NA	NA	Negative	No
0691	No	NA	No	Yes	12.00	Male	Yes	29.32	Mixed-breed	NA	2.0	1.0	No	No	NA	NA	NA	NA	NA	NA	NA	NA	NA	NA	Negative	No
0692	No	NA	Yes	No	5.33	Female	Yes	28.09	Purebred	Golden Retriever	4.0	2.0	No	No	NA	NA	NA	NA	NA	NA	NA	NA	NA	NA	Negative	No
0693	Yes	Laboratory Workflow Deviation	No	No	NA	NA	NA	NA	NA	NA	4.0	2.0	No	No	NA	NA	NA	NA	NA	NA	NA	NA	NA	NA	Negative	No
0694	No	NA	No	Yes	6.00	Male	Yes	22.05	Purebred	Siberian Husky	3.5	2.0	No	No	NA	NA	NA	NA	NA	NA	NA	NA	NA	NA	Negative	No
0695	No	NA	No	Yes	7.00	Female	Yes	35.07	Purebred	Alaskan Malamute	3.0	2.0	No	No	NA	NA	NA	NA	NA	NA	NA	NA	NA	NA	Negative	No
0696	No	NA	No	Yes	1.00	Male	Yes	27.06	Purebred	Golden Retriever	3.0	2.0	No	No	NA	NA	NA	NA	NA	NA	NA	NA	NA	NA	Negative	No

S1 Table. Full subject level data for subjects enrolled in the CANDiD study (continued)

See legend on last page.

Subject	Excluded	Reason for Exclusion	Training Set	Testing Set	Age	Sex	Spay/ Neuter	Weight (kg)	Purebred or Mixed Breed	Breed (Purebred)	Hemolysis	Lipemia	Cancer Diagnosis at Enrollment	Cancer Diagnosed after Liquid Biopsy	Cancer Type	Immunophenotype (Lymphoid Cancer)	Extent of Disease: Non-Lymphoid Cancer	Extent of Disease: Lymphoid Cancer	Tumor Size	Cancer Type (Cancer #2)	Immunophenotype (Cancer #2 if Lymphoid)	Extent of Disease: Non-Lymphoid Cancer (Cancer #2)	Extent of Disease: Lymphoid Cancer (Cancer #2)	Tumor Size (Cancer #2)	Liquid Biopsy Result	CSO Prediction of Hematological Malignancy
0697	Yes	Laboratory Workflow Deviation	No	No	14.00	Female	Yes	5.71	Mixed-breed	NA	3.0	NA	No	No	NA	NA	NA	NA	NA	NA	NA	NA	NA	NA	Negative	No
0698	Yes	Test failure	No	No	8.00	Female	No	23.55	Mixed-breed	NA	3.0	2.0	No	No	NA	NA	NA	NA	NA	NA	NA	NA	NA	NA	Negative	No
0699	Yes	Laboratory Workflow Deviation	No	No	11.00	Male	Yes	9.72	Mixed-breed	NA	4.0	1.0	No	No	NA	NA	NA	NA	NA	NA	NA	NA	NA	NA	Negative	No
0700	Yes	Laboratory Workflow Deviation	No	No	7.92	Male	No	25.05	Purebred	Belgian Malinois	4.0	2.0	No	No	NA	NA	NA	NA	NA	NA	NA	NA	NA	NA	Negative	No
0701	Yes	Test failure	No	No	9.50	Male	Yes	14.23	Mixed-breed	NA	4.0	1.0	No	No	NA	NA	NA	NA	NA	NA	NA	NA	NA	NA	Negative	No
0702	No	NA	No	Yes	4.83	Male	Yes	40.23	Mixed-breed	NA	3.5	2.0	No	No	NA	NA	NA	NA	NA	NA	NA	NA	NA	NA	Negative	No
0703	No	NA	No	Yes	10.67	Male	Yes	13.64	Purebred	Cocker Spaniel	4.0	2.0	No	No	NA	NA	NA	NA	NA	NA	NA	NA	NA	NA	Negative	No
0704	Yes	Laboratory Workflow Deviation	No	No	8.33	Male	Yes	10.68	Mixed-breed	NA	4.0	2.0	No	No	NA	NA	NA	NA	NA	NA	NA	NA	NA	NA	Negative	No
0705	No	NA	No	Yes	8.83	Male	Yes	22.09	Mixed-breed	NA	3.5	1.0	No	No	NA	NA	NA	NA	NA	NA	NA	NA	NA	NA	Negative	No
0706	No	NA	No	Yes	2.08	Female	Yes	27.09	Mixed-breed	NA	3.5	1.0	No	No	NA	NA	NA	NA	NA	NA	NA	NA	NA	NA	Negative	No
0707	No	NA	No	Yes	5.25	Female	Yes	25.09	Mixed-breed	NA	3.5	2.0	No	No	NA	NA	NA	NA	NA	NA	NA	NA	NA	NA	Negative	No
0708	Yes	Test failure	No	No	5.00	Female	Yes	29.45	Mixed-breed	NA	4.0	2.0	No	No	NA	NA	NA	NA	NA	NA	NA	NA	NA	NA	Negative	No
0709	No	NA	No	Yes	6.42	Male	Yes	25.32	Mixed-breed	NA	4.0	2.0	No	No	NA	NA	NA	NA	NA	NA	NA	NA	NA	NA	Negative	No
0710	No	NA	No	Yes	1.17	Male	No	29.82	Purebred	Labrador Retriever	2.0	2.0	No	No	NA	NA	NA	NA	NA	NA	NA	NA	NA	NA	Negative	No
0711	No	NA	No	Yes	7.17	Male	Yes	29.32	Mixed-breed	NA	1.0	0.5	No	No	NA	NA	NA	NA	NA	NA	NA	NA	NA	NA	Negative	No
0712	No	NA	No	Yes	1.58	Female	Yes	18.64	Purebred	Basset Hound	2.0	1.0	No	No	NA	NA	NA	NA	NA	NA	NA	NA	NA	NA	Negative	No
0713	No	NA	No	Yes	13.00	Female	Yes	17.55	Mixed-breed	NA	3.5	2.0	No	No	NA	NA	NA	NA	NA	NA	NA	NA	NA	NA	Negative	No
0714	No	NA	No	Yes	8.17	Male	Yes	33.91	Purebred	Golden Retriever	3.5	2.0	No	No	NA	NA	NA	NA	NA	NA	NA	NA	NA	NA	Negative	No
0715	No	NA	Yes	No	5.92	Male	Yes	32.95	Mixed-breed	NA	4.0	1.5	No	No	NA	NA	NA	NA	NA	NA	NA	NA	NA	NA	Negative	No
0716	Yes	Laboratory Workflow Deviation	No	No	1.58	Male	Yes	33.27	Mixed-breed	NA	4.0	2.0	No	No	NA	NA	NA	NA	NA	NA	NA	NA	NA	NA	Negative	No
0717	No	NA	No	Yes	2.25	Male	Yes	26.41	Mixed-breed	NA	1.0	1.0	No	No	NA	NA	NA	NA	NA	NA	NA	NA	NA	NA	Negative	No
0718	No	NA	No	Yes	2.00	Female	No	33.00	Purebred	Labrador Retriever	2.0	1.0	No	No	NA	NA	NA	NA	NA	NA	NA	NA	NA	NA	Negative	No
0719	No	NA	No	Yes	10.00	Female	Yes	12.91	Mixed-breed	NA	4.0	2.0	No	No	NA	NA	NA	NA	NA	NA	NA	NA	NA	NA	Negative	No
0720	No	NA	No	Yes	2.50	Female	Yes	21.04	Mixed-breed	NA	2.5	0.0	No	No	NA	NA	NA	NA	NA	NA	NA	NA	NA	NA	Negative	No
0721	No	NA	No	Yes	5.50	Female	No	33.57	Purebred	Golden Retriever	4.0	2.0	No	No	NA	NA	NA	NA	NA	NA	NA	NA	NA	NA	Indetermi-nate	No
0722	Yes	Laboratory Workflow Deviation	No	No	6.25	Male	Yes	10.62	Mixed-breed	NA	4.0	1.5	No	No	NA	NA	NA	NA	NA	NA	NA	NA	NA	NA	Negative	No
0723	No	NA	No	Yes	7.33	Male	Yes	35.67	Mixed-breed	NA	2.5	2.0	No	No	NA	NA	NA	NA	NA	NA	NA	NA	NA	NA	Negative	No
0724	No	NA	No	Yes	6.00	Male	Yes	29.26	Mixed-breed	NA	3.0	1.0	No	No	NA	NA	NA	NA	NA	NA	NA	NA	NA	NA	Negative	No
0725	No	NA	No	Yes	8.25	Male	Yes	20.04	Mixed-breed	NA	4.0	2.0	No	No	NA	NA	NA	NA	NA	NA	NA	NA	NA	NA	Negative	No
0726	No	NA	No	Yes	2.92	Female	No	36.08	Purebred	Rottweiler	2.0	1.0	No	No	NA	NA	NA	NA	NA	NA	NA	NA	NA	NA	Negative	No
0727	No	NA	No	Yes	4.08	Male	No	17.54	Purebred	Pembroke Welsh Corgi	3.5	NA	No	No	NA	NA	NA	NA	NA	NA	NA	NA	NA	NA	Negative	No
0728	No	NA	No	Yes	7.08	Male	Yes	26.35	Mixed-breed	NA	4.0	2.0	No	No	NA	NA	NA	NA	NA	NA	NA	NA	NA	NA	Negative	No
0729	No	NA	No	Yes	1.00	Male	Yes	32.87	Purebred	German Shepherd	3.0	2.0	No	No	NA	NA	NA	NA	NA	NA	NA	NA	NA	NA	Negative	No
0730	No	NA	No	Yes	8.00	Male	Yes	20.44	Mixed-breed	NA	3.0	2.0	No	No	NA	NA	NA	NA	NA	NA	NA	NA	NA	NA	Negative	No
0731	No	NA	No	Yes	1.42	Male	Yes	6.01	Mixed-breed	NA	2.0	2.0	No	No	NA	NA	NA	NA	NA	NA	NA	NA	NA	NA	Negative	No
0732	No	NA	No	Yes	6.67	Male	No	23.45	Mixed-breed	NA	2.5	1.5	No	No	NA	NA	NA	NA	NA	NA	NA	NA	NA	NA	Negative	No
0733	No	NA	No	Yes	5.92	Male	Yes	21.75	Mixed-breed	NA	2.0	1.0	No	No	NA	NA	NA	NA	NA	NA	NA	NA	NA	NA	Negative	No
0734	No	NA	No	Yes	6.50	Female	Yes	18.74	Purebred	Siberian Husky	3.5	2.0	No	No	NA	NA	NA	NA	NA	NA	NA	NA	NA	NA	Negative	No
0735	No	NA	No	Yes	2.25	Female	No	25.85	Purebred	Labrador Retriever	1.5	2.0	No	No	NA	NA	NA	NA	NA	NA	NA	NA	NA	NA	Positive	Yes
0736	No	NA	No	Yes	6.58	Male	Yes	25.05	Mixed-breed	NA	3.0	1.0	No	No	NA	NA	NA	NA	NA	NA	NA	NA	NA	NA	Negative	No
0737	No	NA	No	Yes	1.17	Male	Yes	35.57	Purebred	German Shepherd	2.0	0.0	No	No	NA	NA	NA	NA	NA	NA	NA	NA	NA	NA	Negative	No
0738	No	NA	No	Yes	1.25	Male	No	22.65	Mixed-breed	NA	2.5	2.0	No	No	NA	NA	NA	NA	NA	NA	NA	NA	NA	NA	Negative	No
0739	No	NA	No	Yes	2.25	Female	Yes	33.27	Mixed-breed	NA	3.0	2.0	No	No	NA	NA	NA	NA	NA	NA	NA	NA	NA	NA	Negative	No
0740	No	NA	No	Yes	5.25	Female	Yes	33.87	Mixed-breed	NA	2.0	1.0	No	No	NA	NA	NA	NA	NA	NA	NA	NA	NA	NA	Negative	No
0741	No	NA	No	Yes	2.08	Male	No	25.15	Purebred	German Shorthaired Pointer	4.0	2.0	No	No	NA	NA	NA	NA	NA	NA	NA	NA	NA	NA	Indetermi-nate	No
0742	No	NA	No	Yes	4.00	Female	Yes	20.94	Mixed-breed	NA	3.5	2.0	No	No	NA	NA	NA	NA	NA	NA	NA	NA	NA	NA	Negative	No
0743	No	NA	No	Yes	12.50	Female	Yes	17.84	Mixed-breed	NA	3.0	2.0	No	No	NA	NA	NA	NA	NA	NA	NA	NA	NA	NA	Negative	No
0744	No	NA	No	Yes	5.92	Male	No	35.47	Purebred	German Shorthaired Pointer	3.0	2.0	No	No	NA	NA	NA	NA	NA	NA	NA	NA	NA	NA	Negative	No
0745	Yes	Laboratory Workflow Deviation	No	No	8.33	Female	Yes	39.68	Mixed-breed	NA	4.0	2.0	No	No	NA	NA	NA	NA	NA	NA	NA	NA	NA	NA	Negative	No
0746	No	NA	No	Yes	3.00	Male	Yes	34.87	Mixed-breed	NA	3.0	2.0	No	No	NA	NA	NA	NA	NA	NA	NA	NA	NA	NA	Negative	No
0747	No	NA	No	Yes	2.83	Female	No	22.95	Purebred	Siberian Husky	3.5	1.0	No	No	NA	NA	NA	NA	NA	NA	NA	NA	NA	NA	Negative	No
0748	No	NA	No	Yes	2.83	Female	Yes	37.28	Mixed-breed	NA	3.5	2.0	No	No	NA	NA	NA	NA	NA	NA	NA	NA	NA	NA	Negative	No
0749	No	NA	No	Yes	4.67	Male	No	52.11	Purebred	Great Dane	1.5	1.0	No	No	NA	NA	NA	NA	NA	NA	NA	NA	NA	NA	Negative	No
0750	No	NA	No	Yes	5.25	Male	Yes	70.55	Purebred	Great Dane	4.0	2.0	No	No	NA	NA	NA	NA	NA	NA	NA	NA	NA	NA	Negative	No
0751	No	NA	No	Yes	3.00	Male	Yes	20.04	Mixed-breed	NA	2.5	2.0	No	No	NA	NA	NA	NA	NA	NA	NA	NA	NA	NA	Negative	No
0752	Yes	Laboratory Workflow Deviation	No	No	4.00	Female	Yes	18.54	Purebred	Pointer	4.0	2.0	No	No	NA	NA	NA	NA	NA	NA	NA	NA	NA	NA	Negative	No
0753	No	NA	No	Yes	2.17	Male	No	25.65	Purebred	Anatolian Shepherd	4.0	2.0	No	No	NA	NA	NA	NA	NA	NA	NA	NA	NA	NA	Negative	No
0754	No	NA	No	Yes	2.17	Female	Yes	19.04	Mixed-breed	NA	3.0	2.0	No	No	NA	NA	NA	NA	NA	NA	NA	NA	NA	NA	Negative	No
0755	No	NA	No	Yes	3.58	Female	Yes	16.23	Purebred	Beagle	3.5	1.5	No	No	NA	NA	NA	NA	NA	NA	NA	NA	NA	NA	Negative	No
0756	No	NA	No	Yes	5.33	Female	Yes	36.68	Mixed-breed	NA	2.0	2.0	No	No	NA	NA	NA	NA	NA	NA	NA	NA	NA	NA	Negative	No
0757	Yes	Laboratory Workflow Deviation	No	No	NA	NA	NA	NA	NA	NA	3.0	1.0	No	No	NA	NA	NA	NA	NA	NA	NA	NA	NA	NA	Negative	No
0758	Yes	Laboratory Workflow Deviation	No	No	NA	NA	NA	NA	NA	NA	4.0	2.0	No	No	NA	NA	NA	NA	NA	NA	NA	NA	NA	NA	Negative	No
0759	Yes	Laboratory Workflow Deviation	No	No	NA	NA	NA	NA	NA	NA	4.0	1.0	No	No	NA	NA	NA	NA	NA	NA	NA	NA	NA	NA	Negative	No
0760	Yes	Laboratory Workflow Deviation	No	No	NA	NA	NA	NA	NA	NA	4.0	2.0	No	No	NA	NA	NA	NA	NA	NA	NA	NA	NA	NA	Negative	No

S1 Table. Full subject level data for subjects enrolled in the CANDiD study (continued)

See legend on last page.

Subject	Excluded	Reason for Exclusion	Training Set	Testing Set	Age	Sex	Spay/ Neuter	Weight (kg)	Purebred or Mixed Breed	Breed (Purebred)	Hemolysis	Lipemia	Cancer Diagnosis at Enrollment	Cancer Diagnosed after Liquid Biopsy	Cancer Type	Immunophenotype (Lymphoid Cancer)	Extent of Disease: Non-Lymphoid Cancer	Extent of Disease: Lymphoid Cancer	Tumor Size	Cancer Type (Cancer #2)	Immunophenotype (Cancer #2 if Lymphoid)	Extent of Disease: Non-Lymphoid Cancer (Cancer #2)	Extent of Disease: Lymphoid Cancer (Cancer #2)	Tumor Size (Cancer #2)	Liquid Biopsy Result	CSO Prediction of Hematological Malignancy
0761	Yes	Laboratory Workflow Deviation	No	No	NA	NA	NA	NA	NA	NA	2.0	1.0	No	No	NA	NA	NA	NA	NA	NA	NA	NA	NA	NA	Negative	No
0762	No	NA	No	Yes	2.83	Male	No	44.86	Purebred	Labrador Retriever	3.5	1.0	No	No	NA	NA	NA	NA	NA	NA	NA	NA	NA	NA	Negative	No
0763	No	NA	No	Yes	6.92	Male	Yes	30.86	Mixed-breed	NA	2.5	1.0	No	No	NA	NA	NA	NA	NA	NA	NA	NA	NA	NA	Negative	No
0764	No	NA	No	Yes	1.33	Male	No	35.67	Purebred	Golden Retriever	3.0	2.0	No	No	NA	NA	NA	NA	NA	NA	NA	NA	NA	NA	Negative	No
0765	No	NA	No	Yes	1.75	Female	Yes	27.66	Mixed-breed	NA	4.0	1.0	No	No	NA	NA	NA	NA	NA	NA	NA	NA	NA	NA	Negative	No
0766	Yes	Laboratory Workflow Deviation	No	No	NA	NA	NA	NA	NA	NA	3.0	2.0	No	No	NA	NA	NA	NA	NA	NA	NA	NA	NA	NA	Negative	No
0767	No	NA	No	Yes	1.67	Female	Yes	11.36	Purebred	French Bulldog	4.0	1.0	No	No	NA	NA	NA	NA	NA	NA	NA	NA	NA	NA	Negative	No
0768	No	NA	No	Yes	4.33	Male	No	13.13	Mixed-breed	NA	2.5	1.0	No	No	NA	NA	NA	NA	NA	NA	NA	NA	NA	NA	Negative	No
0769	No	NA	No	Yes	1.25	Male	Yes	30.46	Purebred	German Shepherd	2.0	0.0	No	No	NA	NA	NA	NA	NA	NA	NA	NA	NA	NA	Negative	No
0770	No	NA	No	Yes	3.00	Male	Yes	44.99	Purebred	German Shepherd	3.5	1.0	No	No	NA	NA	NA	NA	NA	NA	NA	NA	NA	NA	Negative	No
0771	No	NA	No	Yes	2.50	Female	Yes	44.19	Purebred	Australian Shepherd	4.0	NA	No	No	NA	NA	NA	NA	NA	NA	NA	NA	NA	NA	Negative	No
0772	No	NA	No	Yes	2.17	Female	Yes	23.45	Purebred	Australian Shepherd	2.0	1.0	No	No	NA	NA	NA	NA	NA	NA	NA	NA	NA	NA	Negative	No
0773	No	NA	No	Yes	3.08	Male	Yes	15.23	Mixed-breed	NA	2.0	1.0	No	No	NA	NA	NA	NA	NA	NA	NA	NA	NA	NA	Negative	No
0774	No	NA	No	Yes	2.92	Female	Yes	41.89	Mixed-breed	NA	3.0	1.0	No	No	NA	NA	NA	NA	NA	NA	NA	NA	NA	NA	Negative	No
0775	No	NA	No	Yes	2.67	Male	Yes	24.55	Mixed-breed	NA	4.0	1.0	No	No	NA	NA	NA	NA	NA	NA	NA	NA	NA	NA	Negative	No
0776	Yes	Laboratory Workflow Deviation	No	No	NA	NA	NA	NA	NA	NA	2.5	1.0	No	No	NA	NA	NA	NA	NA	NA	NA	NA	NA	NA	Negative	No
0777	No	NA	No	Yes	2.92	Male	No	39.14	Purebred	Labrador Retriever	4.0	2.0	No	No	NA	NA	NA	NA	NA	NA	NA	NA	NA	NA	Negative	No
0778	Yes	Laboratory Workflow Deviation	No	No	7.58	Male	Yes	20.45	Mixed-breed	NA	4.0	2.0	No	No	NA	NA	NA	NA	NA	NA	NA	NA	NA	NA	Negative	No
0779	No	NA	No	Yes	5.42	Female	Yes	31.67	Mixed-breed	NA	4.0	2.0	No	No	NA	NA	NA	NA	NA	NA	NA	NA	NA	NA	Indetermi-nate	No
0780	No	NA	No	Yes	2.50	Female	No	25.05	Purebred	Labrador Retriever	4.0	1.0	No	No	NA	NA	NA	NA	NA	NA	NA	NA	NA	NA	Negative	No
0781	Yes	Laboratory Workflow Deviation	No	No	NA	NA	NA	NA	NA	NA	2.0	1.0	No	No	NA	NA	NA	NA	NA	NA	NA	NA	NA	NA	Negative	No
0782	No	NA	No	Yes	3.92	Female	Yes	30.26	Mixed-breed	NA	3.5	2.0	No	No	NA	NA	NA	NA	NA	NA	NA	NA	NA	NA	Negative	No
0783	No	NA	No	Yes	2.25	Male	Yes	13.63	Purebred	Boston Terrier	3.0	2.0	No	No	NA	NA	NA	NA	NA	NA	NA	NA	NA	NA	Negative	No
0784	No	NA	No	Yes	8.75	Female	Yes	40.99	Purebred	Doberman Pinscher	2.5	NA	Yes	No	Bone, Osteosarcoma	NA	Localized/Regional	NA	UNK	NA	NA	NA	NA	NA	Negative	No
0785	No	NA	No	Yes	11.75	Female	Yes	29.16	Mixed-breed	NA	2.0	NA	Yes	No	Abdominal Cavity	NA	Localized/Regional	NA	<=5cm	NA	NA	NA	NA	NA	Positive	No
0786	No	NA	No	Yes	9.25	Male	Yes	53.81	Mixed-breed	NA	3.0	NA	Yes	No	Oral Cavity	NA	Localized/Regional	NA	<=5cm	NA	NA	NA	NA	NA	Positive	No
0787	Yes	Enrollment or Clinical Deviation	No	No	3.42	Female	Yes	26.66	Mixed-breed	NA	2.0	NA	Yes	No	NA	NA	NA	NA	NA	NA	NA	NA	NA	NA	Positive	No
0788	Yes	Enrollment or Clinical Deviation	No	No	7.58	Female	Yes	48.10	Purebred	Doberman Pinscher	1.0	NA	Yes	No	NA	NA	NA	NA	NA	NA	NA	NA	NA	NA	Negative	No
0789	No	NA	No	Yes	9.75	Male	Yes	27.76	Purebred	Golden Retriever	2.0	NA	Yes	No	Hemangiosarcoma	NA	Disseminated/Metastatic	NA	<=5cm	NA	NA	NA	NA	NA	Positive	No
0790	No	NA	No	Yes	13.33	Male	Yes	38.68	Mixed-breed	NA	1.0	NA	Yes	No	Soft Tissue Sarcoma	NA	Localized/Regional	NA	<=5cm	NA	NA	NA	NA	NA	Negative	No
0791	No	NA	Yes	No	5.83	Male	Yes	80.37	Purebred	St. Bernard	0.0	NA	Yes	No	Bone, Osteosarcoma	NA	Localized/Regional	NA	>5cm	NA	NA	NA	NA	NA	Positive	No
0792	No	NA	No	Yes	6.42	Female	Yes	45.09	Mixed-breed	NA	1.0	2.0	Yes	No	Lymphoma, Intermediate to Large Cell	B-cell	NA	Disseminated/ Metastatic	<=5cm	NA	NA	NA	NA	NA	Positive	Yes
0793	No	NA	No	Yes	10.08	Male	No	33.07	Purebred	Golden Retriever	1.0	2.0	Yes	No	Lymphoma, Intermediate to Large Cell	B-cell	NA	Disseminated/ Metastatic	>5cm	NA	NA	NA	NA	NA	Positive	No
0794	No	NA	No	Yes	12.17	Female	No	30.06	Mixed-breed	NA	2.0	0.0	Yes	No	Urinary Bladder/Urethra	NA	Localized/Regional	NA	<=5cm	NA	NA	NA	NA	NA	Negative	No
0795	No	NA	No	Yes	8.08	Female	Yes	21.95	Mixed-breed	NA	2.0	NA	Yes	No	Lymphoma, Intermediate to Large Cell	T-Cell	NA	Disseminated/ Metastatic	<=5cm	NA	NA	NA	NA	NA	Positive	No
0796	Yes	Test failure	No	No	12.17	Female	Yes	26.05	Mixed-breed	NA	0.5	1.0	Yes	No	NA	NA	NA	NA	NA	NA	NA	NA	NA	NA	Negative	No
0797	No	NA	No	Yes	7.00	Male	No	40.08	Mixed-breed	NA	1.0	NA	Yes	No	Lymphoma, Intermediate to Large Cell	T-Cell	NA	Disseminated/ Metastatic	<=5cm	NA	NA	NA	NA	NA	Positive	No
0798	No	NA	No	Yes	7.08	Female	Yes	13.53	Mixed-breed	NA	3.0	2.0	Yes	No	Nasal Cavity and Paranasal Sinuses	NA	Localized/Regional	NA	<=5cm	NA	NA	NA	NA	NA	Negative	No
0799	No	NA	No	Yes	6.50	Male	Yes	31.37	Purebred	Greyhound	4.0	2.0	Yes	No	Lymphoma, Intermediate to Large Cell	UNK	NA	Disseminated/ Metastatic	<=5cm	NA	NA	NA	NA	NA	Negative	No
0800	No	NA	Yes	No	10.58	Male	Yes	13.23	Purebred	Shetland Sheepdog	3.0	2.0	Yes	No	Lymphoma, Intermediate to Large Cell	UNK	NA	Disseminated/ Metastatic	>5cm	NA	NA	NA	NA	NA	Positive	No
0801	No	NA	No	Yes	8.00	Male	Yes	34.07	Mixed-breed	NA	2.0	2.0	Yes	No	Nasal Cavity and Paranasal Sinuses	NA	Localized/Regional	NA	<=5cm	NA	NA	NA	NA	NA	Negative	No
0802	No	NA	No	Yes	10.17	Female	Yes	34.47	Purebred	Labrador Retriever	0.0	2.0	Yes	No	Oral Cavity	NA	Localized/Regional	NA	>5cm	NA	NA	NA	NA	NA	Negative	No
0803	No	NA	No	Yes	11.50	Female	Yes	31.06	Mixed-breed	NA	3.0	2.0	Yes	No	Lymphoma, Intermediate to Large Cell	B-cell	NA	Disseminated/ Metastatic	<=5cm	NA	NA	NA	NA	NA	Positive	Yes
0804	No	NA	Yes	No	10.83	Female	Yes	18.94	Mixed-breed	NA	2.0	2.0	Yes	No	Lymphoma, Intermediate to Large Cell	UNK	NA	Disseminated/ Metastatic	>5cm	NA	NA	NA	NA	NA	Positive	No
0805	Yes	Laboratory Workflow Deviation	No	No	14.00	Female	Yes	7.22	Mixed-breed	NA	4.0	2.0	Yes	No	NA	NA	NA	NA	NA	NA	NA	NA	NA	NA	Positive	No
0806	No	NA	No	Yes	10.25	Female	Yes	17.34	Purebred	Dalmation	2.0	2.0	Yes	No	Nasal Cavity and Paranasal Sinuses	NA	Localized/Regional	NA	<=5cm	NA	NA	NA	NA	NA	Negative	No
0807	No	NA	No	Yes	5.50	Male	No	46.00	Purebred	German Shepherd	2.0	2.0	Yes	No	Lymphoma, Intermediate to Large Cell	B-cell	NA	Disseminated/ Metastatic	<=5cm	NA	NA	NA	NA	NA	Positive	No
0808	No	NA	No	Yes	12.00	Female	Yes	35.77	Mixed-breed	NA	3.5	2.0	Yes	No	Mammary Gland Carcinoma	NA	Localized/Regional	NA	>5cm	NA	NA	NA	NA	NA	Positive	No
0809	No	NA	Yes	No	2.00	Female	No	20.04	Mixed-breed	NA	0.0	1.0	Yes	No	Transmissible Venereal Tumor	NA	Localized/Regional	NA	<=5cm	NA	NA	NA	NA	NA	Positive	No
0810	No	NA	No	Yes	12.00	Male	Yes	20.24	Mixed-breed	NA	1.0	2.0	Yes	No	Skin	NA	Localized/Regional	NA	>5cm	NA	NA	NA	NA	NA	Positive	No
0811	No	NA	No	Yes	3.08	Female	Yes	20.04	Mixed-breed	NA	1.0	2.0	Yes	No	Transmissible Venereal Tumor	NA	Localized/Regional	NA	<=5cm	NA	NA	NA	NA	NA	Negative	No
0812	No	NA	No	Yes	14.08	Male	Yes	10.52	Purebred	Cocker Spaniel	3.0	2.0	Yes	No	Nasal Cavity and Paranasal Sinuses	NA	Undetermined	NA	>5cm	NA	NA	NA	NA	NA	Negative	No
0813	No	NA	No	Yes	9.00	Female	Yes	30.56	Mixed-breed	NA	3.0	2.0	Yes	No	Leukemia, Chronic Lymphoid (CLL)	B-cell	NA	Disseminated/ Metastatic	NA	NA	NA	NA	NA	NA	Positive	No
0814	No	NA	Yes	No	9.00	Male	Yes	52.41	Purebred	Bernese Mountain Dogs	1.0	2.0	Yes	No	Mast Cell Tumor	NA	Localized/Regional	NA	<=5cm	NA	NA	NA	NA	NA	Negative	No
0815	No	NA	No	Yes	6.67	Male	Yes	43.89	Mixed-breed	NA	2.5	NA	Yes	No	Lymphoma, Intermediate to Large Cell	B-cell	NA	Disseminated/ Metastatic	>5cm	NA	NA	NA	NA	NA	Positive	No
0816	No	NA	No	Yes	8.00	Female	Yes	31.87	Mixed-breed	NA	3.0	2.0	Yes	No	Lymphoma, Intermediate to Large Cell	UNK	NA	Disseminated/ Metastatic	<=5cm	NA	NA	NA	NA	NA	Positive	No

Subject	Excluded	Reason for Exclusion	Training Set	Testing Set	Age	Sex	Spay/ Neuter	Weight (kg)	Purebred or Mixed Breed	Breed (Purebred)	Hemolysis	Lipemia	Cancer Diagnosis at Enrollment	Cancer Diagnosed after Liquid Biopsy	Cancer Type	Immunophenotype (Lymphoid Cancer)	Extent of Disease: Non-Lymphoid Cancer	Extent of Disease: Lymphoid Cancer	Tumor Size	Cancer Type (Cancer #2)	Immunophenotype (Cancer #2 if Lymphoid)	Extent of Disease: Non-Lymphoid Cancer (Cancer #2)	Extent of Disease: Lymphoid Cancer (Cancer #2)	Tumor Size (Cancer #2)	Liquid Biopsy Result	CSO Prediction of Hematological Malignancy
0817	No	NA	No	Yes	8.83	Male	Yes	17.24	Purebred	Beagle	2.0	NA	Yes	No	Lymphoma, Intermediate to Large Cell	UNK	NA	Disseminated/ Metastatic	<=5cm	NA	NA	NA	NA	NA	Positive	Yes
0818	No	NA	No	Yes	14.00	Male	Yes	15.23	Mixed-breed	NA	4.0	2.0	Yes	No	Urinary Bladder/Urethra	NA	Localized/Regional	NA	<=5cm	NA	NA	NA	NA	NA	Negative	No
0819	Yes	Test failure	No	No	9.00	Male	No	33.37	Purebred	Bernese Mountain Dogs	NA	NA	Yes	No	Urinary Bladder/Urethra	NA	Localized/Regional	NA	<=5cm	NA	NA	NA	NA	NA	Negative	No
0820	No	NA	No	Yes	14.00	Female	Yes	23.25	Mixed-breed	NA	3.5	2.0	Yes	No	Lymphoma, Intermediate to Large Cell	UNK	NA	Disseminated/ Metastatic	<=5cm	NA	NA	NA	NA	NA	Positive	No
0821	No	NA	Yes	No	7.58	Male	Yes	35.07	Mixed-breed	NA	4.0	2.0	Yes	No	Lymphoma, Intermediate to Large Cell	UNK	NA	Disseminated/ Metastatic	>5cm	NA	NA	NA	NA	NA	Positive	No
0822	No	NA	No	Yes	6.42	Male	Yes	29.76	Mixed-breed	NA	3.0	2.0	Yes	No	Lymphoma, Intermediate to Large Cell	B-cell	NA	Disseminated/ Metastatic	>5cm	NA	NA	NA	NA	NA	Positive	No
0823	No	NA	No	Yes	12.92	Female	Yes	22.65	Mixed-breed	NA	4.0	2.0	Yes	No	Lymphoma, Intermediate to Large Cell	B-cell	NA	Disseminated/ Metastatic	>5cm	NA	NA	NA	NA	NA	Positive	Yes
0824	No	NA	No	Yes	6.42	Male	Yes	40.08	Purebred	Doberman Pinscher	4.0	2.0	Yes	No	Mast Cell Tumor	NA	Localized/Regional	NA	>5cm	NA	NA	NA	NA	NA	Positive	No
0825	No	NA	No	Yes	10.50	Male	Yes	67.34	Mixed-breed	NA	2.0	2.0	Yes	No	Lymphoma, Intermediate to Large Cell	T-Cell	NA	Disseminated/ Metastatic	>5cm	NA	NA	NA	NA	NA	Positive	No
0826	No	NA	No	Yes	10.00	Male	Yes	30.26	Mixed-breed	NA	1.5	1.0	Yes	No	Lymphoma, Intermediate to Large Cell	B-cell	NA	Disseminated/ Metastatic	>5cm	NA	NA	NA	NA	NA	Positive	Yes
0827	No	NA	Yes	No	13.00	Female	Yes	35.47	Mixed-breed	NA	4.0	2.0	Yes	No	Mast Cell Tumor	NA	Disseminated/Metastatic	NA	>5cm	NA	NA	NA	NA	NA	Negative	No
0828	No	NA	Yes	No	10.58	Female	Yes	22.25	Purebred	English Bulldog	1.0	2.0	Yes	No	Soft Tissue Sarcoma	NA	Undetermined	NA	<=5cm	NA	NA	NA	NA	NA	Positive	No
0829	No	NA	No	Yes	8.75	Male	Yes	22.15	Mixed-breed	NA	2.0	1.0	Yes	No	Hemangiosarcoma	NA	Disseminated/Metastatic	NA	>5cm	NA	NA	NA	NA	NA	Positive	No
0830	No	NA	No	Yes	11.25	Female	Yes	28.36	Mixed-breed	NA	2.5	2.0	Yes	No	Urinary Bladder/Urethra	NA	Undetermined	NA	<=5cm	NA	NA	NA	NA	NA	Negative	No
0831	No	NA	No	Yes	9.33	Female	Yes	19.34	Mixed-breed	NA	3.5	2.0	Yes	No	Urinary Bladder/Urethra	NA	Localized/Regional	NA	<=5cm	NA	NA	NA	NA	NA	Negative	No
0832	No	NA	No	Yes	7.75	Male	Yes	39.78	Mixed-breed	NA	3.0	2.0	Yes	No	Lymphoma, Intermediate to Large Cell	B-cell	NA	Disseminated/ Metastatic	<=5cm	NA	NA	NA	NA	NA	Positive	No
0833	No	NA	No	Yes	9.42	Male	Yes	30.56	Mixed-breed	NA	2.0	1.0	Yes	No	Mast Cell Tumor	NA	Disseminated/Metastatic	NA	<=5cm	NA	NA	NA	NA	NA	Positive	No
0834	No	NA	No	Yes	10.50	Male	Yes	19.54	Purebred	Basset Hound	4.0	2.0	Yes	No	Anal Sac	NA	Localized/Regional	NA	>5cm	NA	NA	NA	NA	NA	Positive	No
0835	No	NA	No	Yes	9.67	Male	Yes	25.85	Purebred	English Springer Spaniel	2.5	2.0	Yes	No	Anal Sac	NA	Disseminated/Metastatic	NA	>5cm	NA	NA	NA	NA	NA	Positive	No
0836	No	NA	No	Yes	13.83	Male	Yes	34.97	Purebred	Poodle, Standard	4.0	2.0	Yes	No	Nasal Cavity and Paranasal Sinuses	NA	Disseminated/Metastatic	NA	<=5cm	NA	NA	NA	NA	NA	Positive	No
0837	No	NA	No	Yes	4.08	Female	Yes	16.53	Mixed-breed	NA	4.0	2.0	Yes	No	Lymphoma, Intermediate to Large Cell	UNK	NA	Disseminated/ Metastatic	UNK	NA	NA	NA	NA	NA	Positive	Yes
0838	No	NA	No	Yes	6.42	Male	Yes	30.06	Mixed-breed	NA	0.5	1.0	Yes	No	Mast Cell Tumor	NA	Localized/Regional	NA	>5cm	NA	NA	NA	NA	NA	Negative	No
0839	No	NA	No	Yes	11.42	Female	Yes	23.35	Mixed-breed	NA	2.0	2.0	Yes	No	Lymphoma, Intermediate to Large Cell	B-cell	NA	Disseminated/ Metastatic	<=5cm	NA	NA	NA	NA	NA	Positive	Yes
0840	No	NA	No	Yes	9.67	Female	Yes	41.79	Purebred	Rottweiler	2.0	2.0	Yes	No	Anal Sac	NA	Localized/Regional	NA	<=5cm	NA	NA	NA	NA	NA	Negative	No
0841	No	NA	No	Yes	10.50	Male	Yes	36.88	Purebred	Golden Retriever	4.0	2.0	Yes	No	Soft Tissue Sarcoma	NA	Disseminated/Metastatic	NA	>5cm	NA	NA	NA	NA	NA	Positive	No
0842	No	NA	No	Yes	7.17	Male	Yes	51.01	Mixed-breed	NA	2.0	1.0	Yes	No	Lymphoma, Intermediate to Large Cell	UNK	NA	Disseminated/ Metastatic	<=5cm	NA	NA	NA	NA	NA	Positive	Yes
0843	Yes	Laboratory Workflow Deviation	No	No	NA	NA	NA	NA	NA	NA	3.0	1.0	Yes	No	NA	NA	NA	NA	NA	NA	NA	NA	NA	NA	Negative	No
0844	No	NA	Yes	No	10.42	Female	Yes	17.54	Mixed-breed	NA	2.5	2.0	Yes	No	Mast Cell Tumor	NA	Localized/Regional	NA	>5cm	NA	NA	NA	NA	NA	Positive	No
0845	No	NA	Yes	No	5.25	Male	Yes	13.63	Purebred	French Bulldog	2.5	2.0	Yes	No	Mast Cell Tumor	NA	Localized/Regional	NA	<=5cm	NA	NA	NA	NA	NA	Positive	No
0846	No	NA	No	Yes	11.08	Male	Yes	30.66	Mixed-breed	NA	2.0	NA	Yes	No	Lymphoma, Intermediate to Large Cell	B-cell	NA	Disseminated/ Metastatic	<=5cm	NA	NA	NA	NA	NA	Positive	Yes
0847	No	NA	No	Yes	8.00	Female	Yes	37.98	Purebred	German Shepherd	3.0	NA	Yes	No	Mast Cell Tumor	NA	Localized/Regional	NA	<=5cm	NA	NA	NA	NA	NA	Negative	No
0848	No	NA	Yes	No	4.00	Male	Yes	25.65	Mixed-breed	NA	1.0	2.0	Yes	No	Lymphoma, Intermediate to Large Cell	T-Cell	NA	Disseminated/ Metastatic	<=5cm	NA	NA	NA	NA	NA	Positive	No
0849	No	NA	Yes	No	4.92	Female	Yes	23.75	Mixed-breed	NA	2.0	2.0	Yes	No	Lymphoma, Intermediate to Large Cell	B-cell	NA	Disseminated/ Metastatic	<=5cm	NA	NA	NA	NA	NA	Positive	No
0850	No	NA	No	Yes	9.50	Female	Yes	27.36	Purebred	Golden Retriever	1.0	2.0	Yes	No	Lymphoma, Intermediate to Large Cell	B-cell	NA	Disseminated/ Metastatic	>5cm	NA	NA	NA	NA	NA	Positive	No
0851	No	NA	No	Yes	10.00	Female	Yes	13.73	Mixed-breed	NA	3.5	2.0	Yes	No	Soft Tissue Sarcoma	NA	Localized/Regional	NA	<=5cm	NA	NA	NA	NA	NA	Negative	No
0852	No	NA	No	Yes	6.00	Male	Yes	31.87	Purebred	Golden Retriever	4.0	2.0	Yes	No	Anal Sac	NA	Localized/Regional	NA	>5cm	NA	NA	NA	NA	NA	Negative	No
0853	No	NA	Yes	No	8.92	Female	Yes	22.35	Purebred	Labrador Retriever	4.0	2.0	Yes	No	Thymoma	NA	Undetermined	NA	<=5cm	NA	NA	NA	NA	NA	Negative	No
0854	No	NA	No	Yes	8.83	Male	No	38.88	Purebred	Golden Retriever	1.0	1.0	Yes	No	Nasal Cavity and Paranasal Sinuses	NA	Localized/Regional	NA	<=5cm	NA	NA	NA	NA	NA	Negative	No
0855	No	NA	No	Yes	7.00	Female	Yes	22.05	Purebred	Barbet	1.0	1.0	Yes	No	Brain	NA	Localized/Regional	NA	<=5cm	NA	NA	NA	NA	NA	Negative	No
0856	No	NA	Yes	No	6.92	Male	Yes	16.84	Mixed-breed	NA	2.0	2.0	Yes	No	Prostate	NA	Localized/Regional	NA	<=5cm	NA	NA	NA	NA	NA	Negative	No
0857	No	NA	Yes	No	5.67	Male	Yes	13.13	Purebred	Boston Terrier	4.0	2.0	Yes	No	Mast Cell Tumor	NA	Localized/Regional	NA	<=5cm	NA	NA	NA	NA	NA	Negative	No
0858	No	NA	No	Yes	8.25	Male	Yes	17.94	Purebred	Shetland Sheepdog	2.5	1.0	Yes	No	Brain	NA	Localized/Regional	NA	<=5cm	NA	NA	NA	NA	NA	Negative	No
0859	No	NA	No	Yes	9.00	Male	Yes	35.07	Mixed-breed	NA	3.0	2.0	Yes	No	Prostate	NA	Localized/Regional	NA	<=5cm	NA	NA	NA	NA	NA	Negative	No
0860	No	NA	No	Yes	4.50	Female	Yes	29.36	Purebred	German Shorthaired Pointer	2.0	1.0	Yes	No	Lymphoma, Intermediate to Large Cell	B-cell	NA	Disseminated/ Metastatic	>5cm	NA	NA	NA	NA	NA	Positive	Yes
0861	No	NA	No	Yes	11.17	Female	Yes	12.93	Purebred	Pembroke Welsh Corgi	2.5	1.0	Yes	No	Heart Base	NA	Localized/Regional	NA	>5cm	NA	NA	NA	NA	NA	Positive	No
0862	No	NA	Yes	No	2.42	Male	Yes	18.34	Purebred	English Bulldog	3.0	1.0	Yes	No	Leukemia, Chronic Lymphoid (CLL)	T-Cell	NA	Disseminated/ Metastatic	NA	NA	NA	NA	NA	NA	Positive	Yes
0863	No	NA	No	Yes	6.33	Male	Yes	41.59	Purebred	Bernese Mountain Dogs	1.0	2.0	Yes	No	Histiocytic Sarcoma	NA	Undetermined	NA	>5cm	NA	NA	NA	NA	NA	Positive	No
0864	No	NA	No	Yes	7.83	Male	Yes	46.20	Purebred	German Shepherd	2.0	2.0	Yes	No	Lymphoma, Intermediate to Large Cell	UNK	NA	Disseminated/ Metastatic	<=5cm	NA	NA	NA	NA	NA	Positive	No
0865	No	NA	No	Yes	8.00	Male	Yes	36.48	Mixed-breed	NA	2.0	1.0	Yes	No	Urinary Bladder/Urethra	NA	Localized/Regional	NA	<=5cm	NA	NA	NA	NA	NA	Negative	No
0866	No	NA	No	Yes	8.17	Female	Yes	26.25	Purebred	Boxer	3.0	2.0	Yes	No	Lymphoma, Intermediate to Large Cell	B-cell	NA	Disseminated/ Metastatic	UNK	NA	NA	NA	NA	NA	Positive	Yes
0867	No	NA	No	Yes	9.58	Female	Yes	23.85	Mixed-breed	NA	1.0	0.0	Yes	No	Anal Sac	NA	Localized/Regional	NA	<=5cm	NA	NA	NA	NA	NA	Negative	No
0868	No	NA	Yes	No	6.67	Female	Yes	17.84	Purebred	Vizslas	1.5	2.0	Yes	No	Spinal Cord	NA	Localized/Regional	NA	<=5cm	NA	NA	NA	NA	NA	Negative	No
0869	Yes	Enrollment or Clinical Deviation	No	No	6.83	Female	Yes	33.07	Mixed-breed	NA	1.0	2.0	Yes	No	NA	NA	NA	NA	NA	NA	NA	NA	NA	NA	Negative	No
0870	No	NA	No	Yes	11.00	Male	Yes	34.77	Mixed-breed	NA	1.0	2.0	Yes	No	Hemangiosarcoma	NA	Disseminated/Metastatic	NA	>5cm	NA	NA	NA	NA	NA	Positive	No
0871	Yes	Enrollment or Clinical Deviation	No	No	7.00	Male	Yes	33.97	Mixed-breed	NA	0.5	2.0	Yes	No	Malignant Melanoma	NA	Localized/Regional	NA	<=5cm	NA	NA	NA	NA	NA	Negative	No
0872	No	NA	No	Yes	6.92	Male	Yes	30.66	Purebred	Boxer	1.5	2.0	Yes	No	Lymphoma, Intermediate to Large Cell	T-Cell	NA	Disseminated/ Metastatic	>5cm	NA	NA	NA	NA	NA	Positive	No
0873	No	NA	No	Yes	9.50	Male	Yes	33.47	Purebred	Poodle, Standard	3.5	NA	Yes	No	Soft Tissue Sarcoma	NA	Disseminated/Metastatic	NA	>5cm	NA	NA	NA	NA	NA	Negative	No
0874	No	NA	No	Yes	8.08	Female	Yes	20.24	Mixed-breed	NA	2.0	1.0	Yes	No	Mast Cell Tumor	NA	Disseminated/Metastatic	NA	<=5cm	NA	NA	NA	NA	NA	Positive	No
0875	No	NA	Yes	No	10.00	Male	Yes	48.90	Purebred	Labrador Retriever	3.0	2.0	Yes	No	Bone, Osteosarcoma	NA	Localized/Regional	NA	>5cm	NA	NA	NA	NA	NA	Positive	No

S1 Table. Full subject level data for subjects enrolled in the CANDiD study (continued)

See legend on last page.

Subject	Excluded	Reason for Exclusion	Training Set	Testing Set	Age	Sex	Spay/ Neuter	Weight (kg)	Purebred or Mixed Breed	Breed (Purebred)	Hemolysis	Lipemia	Cancer Diagnosis at Enrollment	Cancer Diagnosed after Liquid Biopsy	Cancer Type	Immunophenotype (Lymphoid Cancer)	Extent of Disease: Non-Lymphoid Cancer	Extent of Disease: Lymphoid Cancer	Tumor Size	Cancer Type (Cancer #2)	Immunophenotype (Cancer #2 if Lymphoid)	Extent of Disease: Non-Lymphoid Cancer (Cancer #2)	Extent of Disease: Lymphoid Cancer (Cancer #2)	Tumor Size (Cancer #2)	Liquid Biopsy Result	CSO Prediction of Hematological Malignancy
0876	No	NA	No	Yes	7.08	Male	Yes	57.52	Purebred	German Shepherd	2.5	1.0	Yes	No	Lymphoma, Intermediate to Large Cell	B-cell	NA	Disseminated/ Metastatic	<=5cm	NA	NA	NA	NA	NA	Positive	No
0877	No	NA	Yes	No	15.58	Female	Yes	15.23	Purebred	Cocker Spaniel	2.0	2.0	Yes	No	Lung	NA	Localized/Regional	NA	<=5cm	NA	NA	NA	NA	NA	Negative	No
0878	No	NA	No	Yes	14.00	Male	Yes	13.63	Purebred	Beagle	0.0	1.0	Yes	No	Lymphoma, Intermediate to Large Cell	UNK	NA	Disseminated/ Metastatic	UNK	NA	NA	NA	NA	NA	Positive	No
0879	No	NA	No	Yes	7.08	Female	Yes	33.17	Mixed-breed	NA	2.0	2.0	Yes	No	Mast Cell Tumor	NA	Localized/Regional	NA	<=5cm	NA	NA	NA	NA	NA	Negative	No
0880	No	NA	No	Yes	5.00	Male	Yes	42.59	Purebred	English Mastiff	1.0	2.0	Yes	No	Lymphoma, Intermediate to Large Cell	T-Cell	NA	Disseminated/ Metastatic	<=5cm	NA	NA	NA	NA	NA	Positive	No
0881	No	NA	No	Yes	11.67	Female	Yes	22.15	Mixed-breed	NA	2.0	1.0	Yes	No	Lymphoma, Intermediate to Large Cell	B-cell	NA	Disseminated/ Metastatic	<=5cm	NA	NA	NA	NA	NA	Positive	Yes
0882	No	NA	No	Yes	11.50	Male	Yes	44.09	Purebred	Golden Retriever	3.5	2.0	Yes	No	Nasal Cavity and Paranasal Sinuses	NA	Localized/Regional	NA	>5cm	NA	NA	NA	NA	NA	Negative	No
0883	No	NA	No	Yes	11.92	Female	Yes	22.15	Mixed-breed	NA	2.0	2.0	Yes	No	Mast Cell Tumor	NA	Localized/Regional	NA	>5cm	NA	NA	NA	NA	NA	Positive	No
0884	No	NA	No	Yes	7.33	Female	Yes	24.25	Mixed-breed	NA	3.0	2.0	Yes	No	Lymphoma, Intermediate to Large Cell	B-cell	NA	Disseminated/ Metastatic	<=5cm	NA	NA	NA	NA	NA	Positive	Yes
0885	No	NA	Yes	No	4.00	Female	Yes	35.17	Purebred	Golden Retriever	3.0	2.0	Yes	No	Lymphoma, Intermediate to Large Cell	B-cell	NA	Disseminated/ Metastatic	<=5cm	NA	NA	NA	NA	NA	Positive	Yes
0886	No	NA	No	Yes	10.92	Male	Yes	28.26	Purebred	German Shepherd	1.0	2.0	Yes	No	Anal Sac	NA	Localized/Regional	NA	>5cm	Histiocytic Sarcoma	NA	Localized/Regional	NA	>5cm	Positive	Yes
0887	No	NA	No	Yes	10.08	Male	Yes	31.06	Mixed-breed	NA	2.0	2.0	Yes	No	Hemangiosarcoma	NA	Disseminated/Metastatic	NA	<=5cm	NA	NA	NA	NA	NA	Negative	No
0888	No	NA	No	Yes	4.00	Female	Yes	10.32	Mixed-breed	NA	2.0	2.0	Yes	No	Mast Cell Tumor	NA	Disseminated/Metastatic	NA	>5cm	NA	NA	NA	NA	NA	Positive	No
0889	No	NA	No	Yes	9.00	Male	Yes	14.13	Mixed-breed	NA	3.5	2.0	Yes	No	Lymphoma, Intermediate to Large Cell	T-Cell	NA	Disseminated/ Metastatic	NA	NA	NA	NA	NA	NA	Positive	No
0890	No	NA	No	Yes	12.00	Female	Yes	31.47	Purebred	Vizslas	3.0	2.0	Yes	No	Lymphoma, Intermediate to Large Cell	B-cell	NA	Disseminated/ Metastatic	<=5cm	NA	NA	NA	NA	NA	Positive	Yes
0891	No	NA	No	Yes	14.25	Female	Yes	26.96	Mixed-breed	NA	1.0	0.0	Yes	No	Lymphoma, Intermediate to Large Cell	UNK	NA	Disseminated/ Metastatic	NA	NA	NA	NA	NA	NA	Positive	No
0892	No	NA	No	Yes	7.00	Female	Yes	52.91	Purebred	Dogues de Bordeaux	1.0	1.0	Yes	No	Lymphoma, Intermediate to Large Cell	T-Cell	NA	Disseminated/ Metastatic	<=5cm	NA	NA	NA	NA	NA	Positive	No
0893	No	NA	No	Yes	6.00	Male	Yes	26.76	Mixed-breed	NA	2.5	2.0	Yes	No	Lymphoma, Intermediate to Large Cell	UNK	NA	Disseminated/ Metastatic	>5cm	NA	NA	NA	NA	NA	Positive	No
0894	No	NA	No	Yes	8.58	Female	Yes	31.77	Mixed-breed	NA	3.5	2.0	Yes	No	Lymphoma, Intermediate to Large Cell	B-cell	NA	Disseminated/ Metastatic	<=5cm	NA	NA	NA	NA	NA	Positive	No
0895	No	NA	No	Yes	11.33	Male	Yes	44.89	Purebred	Golden Retriever	NA	0.5	Yes	No	Lymphoma, Intermediate to Large Cell	B-cell	NA	Disseminated/ Metastatic	>5cm	NA	NA	NA	NA	NA	Positive	No
0896	No	NA	No	Yes	7.50	Male	Yes	30.26	Mixed-breed	NA	2.0	0.0	Yes	No	Bone, Osteosarcoma	NA	Localized/Regional	NA	>5cm	NA	NA	NA	NA	NA	Positive	No
0897	No	NA	No	Yes	12.33	Female	Yes	47.80	Purebred	Rhodesian Ridgeback	3.0	1.5	Yes	No	Skin	NA	Localized/Regional	NA	<=5cm	NA	NA	NA	NA	NA	Negative	No
0898	No	NA	Yes	No	11.00	Male	Yes	28.96	Purebred	Border Collie	3.5	1.0	Yes	No	Lymphoma, Intermediate to Large Cell	B-cell	NA	Disseminated/ Metastatic	>5cm	NA	NA	NA	NA	NA	Positive	Yes
0899	No	NA	No	Yes	10.42	Female	Yes	37.28	Purebred	German Shepherd	NA	2.0	Yes	No	Lymphoma, Intermediate to Large Cell	B-cell	NA	Disseminated/ Metastatic	UNK	NA	NA	NA	NA	NA	Positive	No
0900	No	NA	No	Yes	12.00	Male	Yes	14.83	Mixed-breed	NA	3.0	2.0	Yes	No	Lymphoma, Intermediate to Large Cell	UNK	NA	Disseminated/ Metastatic	>5cm	NA	NA	NA	NA	NA	Positive	No
0901	No	NA	No	Yes	10.83	Male	Yes	9.22	Purebred	Scottish Terrier	3.0	2.0	Yes	No	Mast Cell Tumor	NA	Localized/Regional	NA	<=5cm	NA	NA	NA	NA	NA	Negative	No
0902	No	NA	No	Yes	11.08	Male	Yes	20.74	Mixed-breed	NA	2.5	2.0	Yes	No	Lymphoma, Intermediate to Large Cell	B-cell	NA	Disseminated/ Metastatic	>5cm	NA	NA	NA	NA	NA	Positive	Yes
0903	No	NA	No	Yes	8.75	Female	Yes	27.26	Purebred	Doberman Pinscher	2.0	2.0	Yes	No	Lymphoma, Intermediate to Large Cell	B-cell	NA	Disseminated/ Metastatic	<=5cm	NA	NA	NA	NA	NA	Positive	No
0904	No	NA	Yes	No	7.75	Female	Yes	21.04	Purebred	English Bulldog	3.0	2.0	Yes	No	Lymphoma, Intermediate to Large Cell	PARR NEGATIVE	NA	Disseminated/ Metastatic	<=5cm	NA	NA	NA	NA	NA	Positive	Yes
0905	No	NA	No	Yes	7.42	Male	Yes	46.50	Purebred	Rottweiler	1.0	1.0	Yes	No	Lymphoma, Intermediate to Large Cell	B-cell	NA	Disseminated/ Metastatic	>5cm	NA	NA	NA	NA	NA	Positive	Yes
0906	No	NA	No	Yes	8.58	Female	Yes	30.76	Mixed-breed	NA	NA	0.0	Yes	No	Chondrosarcoma	NA	Localized/Regional	NA	>5cm	NA	NA	NA	NA	NA	Positive	No
0907	No	NA	No	Yes	12.42	Female	Yes	30.46	Mixed-breed	NA	3.0	2.0	Yes	No	Oral Cavity	NA	Localized/Regional	NA	<=5cm	NA	NA	NA	NA	NA	Negative	No
0908	No	NA	Yes	No	10.25	Female	Yes	21.75	Mixed-breed	NA	3.0	1.0	Yes	No	Thyroid	NA	Disseminated/Metastatic	NA	>5cm	NA	NA	NA	NA	NA	Positive	No
0909	No	NA	No	Yes	10.25	Female	Yes	25.85	Purebred	Poodle, Standard	2.5	2.0	Yes	No	Lymphoma, Intermediate to Large Cell	UNK	NA	Disseminated/ Metastatic	>5cm	NA	NA	NA	NA	NA	Positive	No
0910	No	NA	No	Yes	10.00	Female	Yes	33.67	Mixed-breed	NA	3.0	2.0	Yes	No	Mast Cell Tumor	NA	Localized/Regional	NA	<=5cm	NA	NA	NA	NA	NA	Negative	No
0911	No	NA	No	Yes	6.17	Female	Yes	24.75	Mixed-breed	NA	1.0	2.0	Yes	No	Lymphoma, Intermediate to Large Cell	B-cell	NA	Disseminated/ Metastatic	>5cm	NA	NA	NA	NA	NA	Positive	No
0912	No	NA	No	Yes	10.00	Female	Yes	31.67	Mixed-breed	NA	2.0	1.0	Yes	No	Bone, Osteosarcoma	NA	Localized/Regional	NA	>5cm	NA	NA	NA	NA	NA	Positive	No
0913	No	NA	No	Yes	6.00	Female	Yes	29.26	Purebred	Small Munsterlander	2.0	2.0	Yes	No	Lymphoma, Intermediate to Large Cell	B-cell	NA	Disseminated/ Metastatic	>5cm	NA	NA	NA	NA	NA	Positive	No
0914	No	NA	Yes	No	5.50	Male	Yes	21.44	Mixed-breed	NA	0.0	2.0	Yes	No	Leukemia, Acute Lymphoid (ALL)	T-Cell	NA	Disseminated/ Metastatic	NA	NA	NA	NA	NA	NA	Positive	Yes
0915	No	NA	No	Yes	8.67	Male	Yes	27.96	Mixed-breed	NA	3.0	2.0	Yes	No	Malignant Melanoma	NA	Localized/Regional	NA	<=5cm	NA	NA	NA	NA	NA	Negative	No
0916	No	NA	No	Yes	5.00	Female	No	28.86	Purebred	Boxer	3.0	2.0	Yes	No	Lymphoma, Intermediate to Large Cell	B-cell	NA	Disseminated/ Metastatic	>5cm	NA	NA	NA	NA	NA	Positive	Yes
0917	No	NA	Yes	No	9.17	Female	Yes	31.06	Purebred	Boxer	2.0	2.0	Yes	No	Lymphoma, Intermediate to Large Cell	B-cell	NA	Disseminated/ Metastatic	<=5cm	NA	NA	NA	NA	NA	Positive	Yes
0918	No	NA	No	Yes	8.42	Female	Yes	33.67	Purebred	Bernese Mountain Dogs	3.5	2.0	Yes	No	Histiocytic Sarcoma	NA	Undetermined	NA	>5cm	NA	NA	NA	NA	NA	Positive	No
0919	No	NA	Yes	No	6.58	Male	Yes	46.30	Purebred	Rhodesian Ridgeback	1.0	2.0	Yes	No	Lymphoma, Intermediate to Large Cell	T-Cell	NA	Disseminated/ Metastatic	<=5cm	NA	NA	NA	NA	NA	Positive	No
0920	No	NA	No	Yes	13.58	Male	Yes	30.16	Purebred	Golden Retriever	1.0	2.0	Yes	No	Lymphoma, Intermediate to Large Cell	B-cell	NA	Disseminated/ Metastatic	<=5cm	NA	NA	NA	NA	NA	Positive	Yes
0921	No	NA	No	Yes	14.92	Female	Yes	29.96	Mixed-breed	NA	1.0	2.0	Yes	No	Lymphoma, Intermediate to Large Cell	UNK	NA	Disseminated/ Metastatic	<=5cm	NA	NA	NA	NA	NA	Positive	Yes
0922	No	NA	No	Yes	8.42	Male	Yes	37.48	Purebred	Doberman Pinscher	2.5	2.0	Yes	No	Lymphoma, Intermediate to Large Cell	B-cell	NA	Disseminated/ Metastatic	>5cm	NA	NA	NA	NA	NA	Positive	No
0923	No	NA	No	Yes	11.08	Female	Yes	27.86	Mixed-breed	NA	3.5	2.0	Yes	No	Lymphoma, Intermediate to Large Cell	T-Cell	NA	Disseminated/ Metastatic	>5cm	NA	NA	NA	NA	NA	Positive	No
0924	No	NA	No	Yes	11.92	Male	Yes	29.36	Mixed-breed	NA	3.0	2.0	Yes	No	Leukemia, Acute Lymphoid (ALL)	T-Cell	NA	Disseminated/ Metastatic	NA	NA	NA	NA	NA	NA	Positive	No
0925	No	NA	No	Yes	10.33	Male	Yes	40.08	Purebred	Poodle, Standard	3.0	2.0	Yes	No	Lymphoma, Intermediate to Large Cell	B-cell	NA	Disseminated/ Metastatic	<=5cm	NA	NA	NA	NA	NA	Negative	No
0926	No	NA	No	Yes	10.25	Female	Yes	31.06	Purebred	German Shepherd	2.0	1.0	Yes	No	Brain	NA	Localized/Regional	NA	<=5cm	NA	NA	NA	NA	NA	Positive	No
0927	No	NA	No	Yes	11.08	Male	UNK	30.56	Mixed-breed	NA	4.0	NA	Yes	No	Chondrosarcoma	NA	Localized/Regional	NA	>5cm	NA	NA	NA	NA	NA	Negative	No
0928	No	NA	No	Yes	11.33	Male	Yes	23.05	Mixed-breed	NA	1.0	1.0	Yes	No	Nasal Cavity and Paranasal Sinuses	NA	Localized/Regional	NA	<=5cm	NA	NA	NA	NA	NA	Positive	No

S1 Table. Full subject level data for subjects enrolled in the CANDiD study (continued)

See legend on last page.

Subject	Excluded	Reason for Exclusion	Training Set	Testing Set	Age	Sex	Spay/ Neuter	Weight (kg)	Purebred or Mixed Breed	Breed (Purebred)	Hemolysis	Lipemia	Cancer Diagnosis at Enrollment	Cancer Diagnosed after Liquid Biopsy	Cancer Type	Immunophenotype (Lymphoid Cancer)	Extent of Disease: Non-Lymphoid Cancer	Extent of Disease: Lymphoid Cancer	Tumor Size	Cancer Type (Cancer #2)	Immunophenotype (Cancer #2 if Lymphoid)	Extent of Disease: Non-Lymphoid Cancer (Cancer #2)	Extent of Disease: Lymphoid Cancer (Cancer #2)	Tumor Size (Cancer #2)	Liquid Biopsy Result	CSO Prediction of Hematological Malignancy
0929	No	NA	No	Yes	8.08	Male	Yes	19.84	Mixed-breed	NA	2.0	2.0	Yes	No	Prostate	NA	Localized/Regional	NA	<=5cm	NA	NA	NA	NA	NA	Negative	No
0930	No	NA	No	Yes	10.00	Female	Yes	22.35	Mixed-breed	NA	3.0	NA	Yes	No	Lymphoma, Indolent	T ZONE	NA	Disseminated/ Metastatic	<=5cm	NA	NA	NA	NA	NA	Positive	Yes
0931	Yes	Laboratory Workflow Deviation	No	No	6.50	Male	Yes	29.86	Purebred	American Staffordshire Terrier	4.0	2.0	Yes	No	Pituitary	NA	Localized/Regional	NA	<=5cm	NA	NA	NA	NA	NA	Negative	No
0932	No	NA	Yes	No	9.83	Male	Yes	47.30	Mixed-breed	NA	4.0	2.0	Yes	No	Urinary Bladder/Urethra	NA	Localized/Regional	NA	<=5cm	NA	NA	NA	NA	NA	Negative	No
0933	No	NA	Yes	No	14.25	Female	Yes	22.05	Mixed-breed	NA	2.5	2.0	Yes	No	Soft Tissue Sarcoma	NA	Localized/Regional	NA	>5cm	NA	NA	NA	NA	NA	Negative	No
0934	No	NA	No	Yes	10.08	Female	Yes	21.95	Mixed-breed	NA	2.0	NA	Yes	No	Mast Cell Tumor	NA	Disseminated/Metastatic	NA	<=5cm	NA	NA	NA	NA	NA	Indetermi- nate	No
0935	No	NA	No	Yes	9.00	Male	Yes	32.47	Purebred	Boxer	4.0	1.0	Yes	No	Lymphoma, Intermediate to Large Cell	B-cell	NA	Disseminated/ Metastatic	<=5cm	NA	NA	NA	NA	NA	Positive	Yes
0936	Yes	Enrollment or Clinical Deviation	No	No	12.92	Male	Yes	22.85	Mixed-breed	NA	2.5	1.0	Yes	No	NA	NA	NA	NA	NA	NA	NA	NA	NA	NA	Negative	No
0937	No	NA	No	Yes	11.33	Female	Yes	25.65	Purebred	American Staffordshire Terrier	3.5	1.0	Yes	No	Urinary Bladder/Urethra	NA	Localized/Regional	NA	NA	NA	NA	NA	NA	NA	Negative	No
0938	No	NA	No	Yes	10.42	Male	Yes	36.98	Purebred	Labrador Retriever	2.5	1.5	Yes	No	Bone, Osteosarcoma	NA	Localized/Regional	NA	>5cm	NA	NA	NA	NA	NA	Positive	No
0939	No	NA	Yes	No	13.42	Female	Yes	33.07	Purebred	American Staffordshire Terrier	3.0	2.0	Yes	No	Lymphoma, Indolent	T ZONE	NA	Localized/ Regional	<=5cm	NA	NA	NA	NA	NA	Negative	No
0940	No	NA	No	Yes	4.50	Male	Yes	29.06	Purebred	Bulldog, American	2.0	2.0	Yes	No	Lymphoma, Intermediate to Large Cell	T-Cell	NA	Disseminated/ Metastatic	>5cm	NA	NA	NA	NA	NA	Positive	No
0941	No	NA	No	Yes	5.33	Female	Yes	35.57	Mixed-breed	NA	2.0	2.0	Yes	No	Bone, Osteosarcoma	NA	Localized/Regional	NA	<=5cm	NA	NA	NA	NA	NA	Negative	No
0942	No	NA	No	Yes	2.08	Male	Yes	31.77	Purebred	Golden Retriever	0.5	1.0	Yes	No	Lymphoma, Intermediate to Large Cell	INCONCLUSIVE	NA	Disseminated/ Metastatic	>5cm	NA	NA	NA	NA	NA	Positive	No
0943	No	NA	No	Yes	6.92	Female	Yes	35.47	Purebred	Labrador Retriever	2.5	2.5	Yes	No	Mast Cell Tumor	NA	Localized/Regional	NA	>5cm	NA	NA	NA	NA	NA	Positive	No
0944	No	NA	No	Yes	6.75	Male	Yes	26.05	Purebred	English Bulldog	2.5	1.5	Yes	No	Soft Tissue Sarcoma	NA	Disseminated/Metastatic	NA	>5cm	NA	NA	NA	NA	NA	Negative	No
0945	No	NA	Yes	No	9.25	Male	Yes	24.35	Mixed-breed	NA	3.0	2.0	Yes	No	Heart Base	NA	Localized/Regional	NA	UNK	NA	NA	NA	NA	NA	Positive	No
0946	Yes	Test failure	No	No	8.92	Female	Yes	19.34	Mixed-breed	NA	4.0	2.0	Yes	No	Lymphoma, Intermediate to Large Cell	T-Cell	NA	Disseminated/ Metastatic	NA	NA	NA	NA	NA	NA	Indetermi- nate	No
0947	No	NA	No	Yes	13.00	Male	Yes	6.91	Mixed-breed	NA	2.0	1.5	Yes	No	Lymphoma, Indolent	T ZONE	NA	Disseminated/ Metastatic	NA	NA	NA	NA	NA	NA	Negative	No
0948	No	NA	No	Yes	11.75	Female	Yes	13.03	Mixed-breed	NA	3.5	2.0	Yes	No	Mast Cell Tumor	NA	Localized/Regional	NA	>5cm	NA	NA	NA	NA	NA	Negative	No
0949	No	NA	No	Yes	9.92	Male	Yes	41.29	Mixed-breed	NA	3.0	2.0	Yes	No	Lymphoma, Intermediate to Large Cell	UNK	NA	Disseminated/ Metastatic	NA	NA	NA	NA	NA	NA	Positive	No
0950	No	NA	No	Yes	8.00	Female	Yes	24.85	Mixed-breed	NA	3.5	2.0	Yes	No	Mast Cell Tumor	NA	Localized/Regional	NA	>5cm	NA	NA	NA	NA	NA	Negative	No
0951	No	NA	Yes	No	10.33	Male	Yes	14.93	Purebred	Beagle	2.5	2.0	Yes	No	Urinary Bladder/Urethra	NA	Undetermined	NA	<=5cm	NA	NA	NA	NA	NA	Positive	No
0952	No	NA	Yes	No	9.75	Male	Yes	81.67	Mixed-breed	NA	3.0	2.0	Yes	No	Lymphoma, Intermediate to Large Cell	B-cell	NA	Disseminated/ Metastatic	>5cm	NA	NA	NA	NA	NA	Positive	Yes
0953	No	NA	No	Yes	8.00	Female	Yes	56.32	Purebred	Great Dane	3.0	2.0	Yes	No	Bone, Osteosarcoma	NA	Localized/Regional	NA	<=5cm	NA	NA	NA	NA	NA	Negative	No
0954	No	NA	No	Yes	11.00	Female	Yes	36.68	Mixed-breed	NA	3.0	1.0	Yes	No	Multiple Myeloma	NA	Disseminated/Metastatic	NA	<=5cm	NA	NA	NA	NA	NA	Negative	No
0955	No	NA	No	Yes	11.17	Male	Yes	9.42	Purebred	West Highland White Terrier	4.0	2.0	Yes	No	Skin	NA	Disseminated/Metastatic	NA	<=5cm	NA	NA	NA	NA	NA	Negative	No
0956	No	NA	No	Yes	14.83	Female	Yes	15.83	Purebred	Border Collie	3.0	2.0	Yes	No	Urinary Bladder/Urethra	NA	Localized/Regional	NA	<=5cm	NA	NA	NA	NA	NA	Negative	No
0957	No	NA	No	Yes	11.00	Male	Yes	33.17	Purebred	Labrador Retriever	3.5	2.0	Yes	No	Multiple Myeloma	NA	Undetermined	NA	NA	NA	NA	NA	NA	NA	Negative	No
0958	No	NA	No	Yes	5.00	Male	Yes	57.62	Purebred	German Shepherd	NA	1.0	Yes	No	Bone, Osteosarcoma	NA	Localized/Regional	NA	>5cm	NA	NA	NA	NA	NA	Positive	No
0959	No	NA	Yes	No	6.00	Male	Yes	48.30	Mixed-breed	NA	3.0	1.0	Yes	No	Lymphoma, Intermediate to Large Cell	B-cell	NA	Disseminated/ Metastatic	<=5cm	NA	NA	NA	NA	NA	Positive	Yes
0960	No	NA	Yes	No	7.67	Male	Yes	30.36	Purebred	Golden Retriever	2.0	1.0	Yes	No	Prostate	NA	Localized/Regional	NA	<=5cm	NA	NA	NA	NA	NA	Negative	No
0961	No	NA	No	Yes	12.92	Female	Yes	16.43	Purebred	Pembroke Welsh Corgi	3.0	2.0	Yes	No	Lymphoma, Intermediate to Large Cell	B-cell	NA	Disseminated/ Metastatic	<=5cm	NA	NA	NA	NA	NA	Negative	No
0962	No	NA	No	Yes	7.25	Female	Yes	30.16	Mixed-breed	NA	3.5	2.0	Yes	No	Lymphoma, Intermediate to Large Cell	B-cell	NA	Disseminated/ Metastatic	<=5cm	NA	NA	NA	NA	NA	Positive	Yes
0963	No	NA	No	Yes	11.00	Male	Yes	32.27	Mixed-breed	NA	3.5	1.0	Yes	No	Lymphoma, Intermediate to Large Cell	B-cell	NA	Disseminated/ Metastatic	<=5cm	NA	NA	NA	NA	NA	Positive	No
0964	No	NA	No	Yes	5.75	Male	Yes	43.09	Purebred	Golden Retriever	3.5	2.0	Yes	No	Lymphoma, Indolent	T ZONE	NA	Disseminated/ Metastatic	<=5cm	NA	NA	NA	NA	NA	Positive	No
0965	No	NA	No	Yes	7.67	Female	Yes	29.06	Purebred	Golden Retriever	2.0	0.0	Yes	No	Histiocytic Sarcoma	NA	Localized/Regional	NA	<=5cm	NA	NA	NA	NA	NA	Positive	Yes
0966	No	NA	No	Yes	5.33	Female	Yes	37.48	Mixed-breed	NA	3.5	2.0	Yes	No	Soft Tissue Sarcoma	NA	Localized/Regional	NA	<=5cm	NA	NA	NA	NA	NA	Negative	No
0967	Yes	Enrollment or Clinical Deviation	No	No	11.33	Male	Yes	24.55	Purebred	Vizslas	2.0	2.0	Yes	No	NA	NA	NA	NA	NA	NA	NA	NA	NA	NA	Negative	No
0968	No	NA	No	Yes	12.00	Male	Yes	11.82	Purebred	Boston Terrier	3.0	2.0	Yes	No	Hemangiosarcoma	NA	Disseminated/Metastatic	NA	<=5cm	NA	NA	NA	NA	NA	Negative	No
0969	No	NA	No	Yes	7.58	Female	No	32.07	Purebred	Siberian Husky	4.0	2.0	Yes	No	Lymphoma, Intermediate to Large Cell	B-cell	NA	Disseminated/ Metastatic	>5cm	NA	NA	NA	NA	NA	Positive	Yes
0970	No	NA	No	Yes	10.00	Male	Yes	19.94	Purebred	Siberian Husky	4.0	2.0	Yes	No	Lymphoma, Intermediate to Large Cell	UNK	NA	Disseminated/ Metastatic	>5cm	NA	NA	NA	NA	NA	Positive	No
0971	No	NA	Yes	No	9.08	Female	Yes	26.35	Purebred	Collie	4.0	NA	Yes	No	Urinary Bladder/Urethra	NA	Undetermined	NA	>5cm	NA	NA	NA	NA	NA	Negative	No
0972	No	NA	Yes	No	9.33	Male	Yes	36.58	Purebred	Labrador Retriever	1.0	0.0	Yes	No	Bone, Fibrosarcoma	NA	Localized/Regional	NA	<=5cm	NA	NA	NA	NA	NA	Negative	No
0973	No	NA	Yes	No	6.50	Female	Yes	32.87	Purebred	Labrador Retriever	3.5	2.0	Yes	No	Bone, Multilobular Osteochondrosarcoma	NA	Localized/Regional	NA	>5cm	NA	NA	NA	NA	NA	Negative	No
0974	No	NA	No	Yes	12.00	Female	No	22.15	Purebred	Siberian Husky	2.0	0.0	Yes	No	Lung	NA	Localized/Regional	NA	UNK	NA	NA	NA	NA	NA	Negative	No
0975	No	NA	No	Yes	11.25	Female	Yes	27.06	Purebred	Labrador Retriever	2.5	1.5	Yes	No	Nasal Cavity and Paranasal Sinuses	NA	Localized/Regional	NA	>5cm	NA	NA	NA	NA	NA	Positive	No
0976	No	NA	No	Yes	9.00	Female	Yes	50.10	Purebred	Golden Retriever	4.0	2.0	Yes	No	Soft Tissue Sarcoma	NA	Undetermined	NA	>5cm	NA	NA	NA	NA	NA	Positive	No
0977	No	NA	No	Yes	9.58	Female	Yes	33.57	Purebred	Labrador Retriever	2.0	0.0	Yes	No	Leukemia, Chronic Lymphoid (CLL)	T-Cell	NA	Disseminated/ Metastatic	NA	NA	NA	NA	NA	NA	Positive	No
0978	No	NA	No	Yes	6.92	Male	No	37.78	Purebred	Rottweiler	3.0	1.0	Yes	No	Lymphoma, Intermediate to Large Cell	UNK	NA	Disseminated/ Metastatic	<=5cm	NA	NA	NA	NA	NA	Positive	Yes
0979	No	NA	No	Yes	12.58	Male	Yes	33.57	Purebred	Golden Retriever	1.0	0.0	Yes	No	Lymphoma, Intermediate to Large Cell	B-cell	NA	Disseminated/ Metastatic	>5cm	Lymphoma, Indolent	T ZONE	NA	Disseminated/ Metastatic	>5cm	Positive	Yes
0980	No	NA	Yes	No	11.83	Female	Yes	14.83	Purebred	Beagle	4.0	2.0	Yes	No	Thyroid	NA	Disseminated/Metastatic	NA	<=5cm	NA	NA	NA	NA	NA	Positive	No
0981	Yes	Test failure	No	No	7.08	Male	Yes	32.07	Mixed-breed	NA	2.0	NA	Yes	No	NA	NA	NA	NA	NA	NA	NA	NA	NA	NA	Negative	No
0982	No	NA	No	Yes	15.00	Male	Yes	25.35	Mixed-breed	NA	NA	1.5	Yes	No	Lymphoma, Intermediate to Large Cell	B-cell	NA	Disseminated/ Metastatic	<=5cm	NA	NA	NA	NA	NA	Positive	Yes
0983	No	NA	Yes	No	3.50	Female	Yes	30.96	Purebred	German Shepherd	3.0	2.0	Yes	No	Lymphoma, Intermediate to Large Cell	B-cell	NA	Disseminated/ Metastatic	<=5cm	NA	NA	NA	NA	NA	Positive	No
0984	No	NA	No	Yes	10.67	Female	Yes	35.07	Mixed-breed	NA	2.5	NA	Yes	No	Peripheral Nerve Sheath Tumor	NA	Localized/Regional	NA	<=5cm	NA	NA	NA	NA	NA	Negative	No

S1 Table. Full subject level data for subjects enrolled in the CANDiD study (continued)

See legend on last page.

Subject	Excluded	Reason for Exclusion	Training Set	Testing Set	Age	Sex	Spay/ Neuter	Weight (kg)	Purebred or Mixed Breed	Breed (Purebred)	Hemolysis	Lipemia	Cancer Diagnosis at Enrollment	Cancer Diagnosed after Liquid Biopsy	Cancer Type	Immunophenotype (Lymphoid Cancer)	Extent of Disease: Non-Lymphoid Cancer	Extent of Disease: Lymphoid Cancer	Tumor Size	Cancer Type (Cancer #2)	Immunophenotype (Cancer #2 if Lymphoid)	Extent of Disease: Non-Lymphoid Cancer (Cancer #2)	Extent of Disease: Lymphoid Cancer (Cancer #2)	Tumor Size (Cancer #2)	Liquid Biopsy Result	CSO Prediction of Hematological Malignancy
0985	No	NA	Yes	No	12.42	Female	Yes	36.78	Purebred	Labrador Retriever	3.0	2.0	Yes	No	Anal Sac	NA	Localized/Regional	NA	<=5cm	NA	NA	NA	NA	NA	Negative	No
0986	No	NA	No	Yes	12.50	Male	Yes	15.73	Mixed-breed	NA	3.0	2.0	Yes	No	Lung	NA	Localized/Regional	NA	>5cm	NA	NA	NA	NA	NA	Positive	No
0987	No	NA	No	Yes	11.92	Male	Yes	26.25	Purebred	Australian Shepherd	3.0	2.0	Yes	No	Lymphoma, Intermediate to Large Cell	B-cell	NA	Disseminated/ Metastatic	UNK	NA	NA	NA	NA	NA	Positive	Yes
0988	No	NA	No	Yes	9.00	Male	No	37.08	Purebred	Golden Retriever	NA	0.0	Yes	No	Lymphoma, Indolent	T ZONE	NA	Localized/ Regional	>5cm	NA	NA	NA	NA	NA	Positive	No
0989	No	NA	No	Yes	11.58	Male	Yes	21.95	Purebred	Australian Shepherd	2.0	2.0	Yes	No	Lymphoma, Intermediate to Large Cell	T-Cell	NA	Disseminated/ Metastatic	<=5cm	NA	NA	NA	NA	NA	Positive	No
0990	No	NA	No	Yes	8.42	Male	Yes	40.08	Mixed-breed	NA	2.0	2.0	Yes	No	Thyroid	NA	Disseminated/Metastatic	NA	>5cm	NA	NA	NA	NA	NA	Positive	No
0991	No	NA	No	Yes	7.75	Male	Yes	39.48	Mixed-breed	NA	2.5	2.0	Yes	No	Lymphoma, Intermediate to Large Cell	B-cell	NA	Disseminated/ Metastatic	>5cm	NA	NA	NA	NA	NA	Positive	Yes
0992	No	NA	No	Yes	10.42	Female	Yes	25.85	Mixed-breed	NA	3.0	2.0	Yes	No	Lymphoma, Intermediate to Large Cell	B-cell	NA	Disseminated/ Metastatic	<=5cm	NA	NA	NA	NA	NA	Positive	No
0993	No	NA	Yes	No	7.25	Male	Yes	13.53	Purebred	Cavalier King Charles Spaniel	3.0	2.0	Yes	No	Lymphoma, Indolent	T ZONE	NA	Localized/ Regional	<=5cm	NA	NA	NA	NA	NA	Negative	No
0994	No	NA	No	Yes	5.00	Male	No	62.43	Purebred	Cane Corso	2.5	2.0	Yes	No	Lymphoma, Intermediate to Large Cell	UNK	NA	Disseminated/ Metastatic	>5cm	NA	NA	NA	NA	NA	Positive	No
0995	Yes	Laboratory Workflow Deviation	No	No	9.67	Male	Yes	7.42	Mixed-breed	NA	2.0	1.0	Yes	No	NA	NA	NA	NA	NA	NA	NA	NA	NA	NA	Positive	No
0996	No	NA	No	Yes	4.67	Male	Yes	15.93	Mixed-breed	NA	2.0	1.0	Yes	No	Lymphoma, Intermediate to Large Cell	B-cell	NA	Disseminated/ Metastatic	>5cm	NA	NA	NA	NA	NA	Positive	No
0997	Yes	Laboratory Workflow Deviation	No	No	11.00	Female	Yes	9.42	Mixed-breed	NA	2.0	2.0	Yes	No	NA	NA	NA	NA	NA	NA	NA	NA	NA	NA	Positive	No
0998	No	NA	Yes	No	5.00	Female	Yes	27.76	Mixed-breed	NA	4.0	2.0	Yes	No	Lymphoma, Intermediate to Large Cell	B-cell	NA	Disseminated/ Metastatic	>5cm	NA	NA	NA	NA	NA	Positive	Yes
0999	No	NA	No	Yes	11.50	Female	Yes	38.58	Purebred	German Shepherd	4.0	2.0	Yes	No	Soft Tissue Sarcoma	NA	Disseminated/Metastatic	NA	<=5cm	NA	NA	NA	NA	NA	Positive	No
1000	Yes	Laboratory Workflow Deviation	No	No	9.75	Female	UNK	32.07	Purebred	Golden Retriever	4.0	2.0	Yes	No	Lymphoma, Indolent	T ZONE	NA	Disseminated/ Metastatic	<=5cm	NA	NA	NA	NA	NA	Negative	No
1001	No	NA	No	Yes	3.67	Male	Yes	37.08	Purebred	Golden Retriever	1.0	0.0	Yes	No	Lymphoma, Intermediate to Large Cell	B-cell	NA	Localized/ Regional	>5cm	NA	NA	NA	NA	NA	Positive	Yes
1002	Yes	Laboratory Workflow Deviation	No	No	10.17	Female	Yes	7.42	Mixed-breed	NA	2.0	2.0	Yes	No	NA	NA	NA	NA	NA	NA	NA	NA	NA	NA	Negative	No
1003	No	NA	No	Yes	12.42	Female	Yes	23.05	Mixed-breed	NA	3.5	2.0	Yes	No	Lymphoma, Intermediate to Large Cell	NA	NA	Disseminated/ Metastatic	<=5cm	NA	NA	NA	NA	NA	Positive	No
1004	No	NA	No	Yes	12.00	Female	Yes	20.04	Mixed-breed	NA	3.0	0.0	Yes	No	Histiocytic Sarcoma	NA	Localized/Regional	NA	<=5cm	NA	NA	NA	NA	NA	Negative	No
1005	No	NA	No	Yes	9.00	Male	Yes	27.86	Purebred	Basset Hound	4.0	2.0	Yes	No	Lymphoma, Intermediate to Large Cell	T-Cell	NA	Disseminated/ Metastatic	<=5cm	NA	NA	NA	NA	NA	Positive	No
1006	No	NA	No	Yes	3.00	Male	Yes	23.75	Mixed-breed	NA	2.0	2.0	Yes	No	Lymphoma, Intermediate to Large Cell	B-cell	NA	Disseminated/ Metastatic	<=5cm	NA	NA	NA	NA	NA	Positive	No
1007	No	NA	No	Yes	4.83	Male	Yes	18.74	Purebred	Staffordshire Bull Terrier	2.0	2.0	Yes	No	Lymphoma, Intermediate to Large Cell	B-cell	NA	Disseminated/ Metastatic	>5cm	NA	NA	NA	NA	NA	Positive	No
1008	No	NA	No	Yes	2.92	Male	No	13.53	Purebred	French Bulldog	3.5	2.0	Yes	No	Lymphoma, Intermediate to Large Cell	B-cell	NA	Disseminated/ Metastatic	<=5cm	NA	NA	NA	NA	NA	Positive	Yes
1009	No	NA	No	Yes	10.50	Female	Yes	52.61	Mixed-breed	NA	3.0	1.0	Yes	No	Mast Cell Tumor	NA	Localized/Regional	NA	<=5cm	NA	NA	NA	NA	NA	Negative	No
1010	No	NA	No	Yes	14.50	Female	Yes	21.44	Purebred	Australian Cattle Dog	2.0	0.0	Yes	No	Lymphoma, Intermediate to Large Cell	B-cell	NA	Disseminated/ Metastatic	>5cm	NA	NA	NA	NA	NA	Positive	Yes
1011	Yes	Laboratory Workflow Deviation	No	No	9.17	Male	Yes	8.42	Mixed-breed	NA	3.0	1.0	Yes	No	NA	NA	NA	NA	NA	NA	NA	NA	NA	NA	Positive	Yes
1012	No	NA	No	Yes	5.00	Female	Yes	16.94	Purebred	French Bulldog	2.0	0.0	Yes	No	Lymphoma, Intermediate to Large Cell	B-cell	NA	Disseminated/ Metastatic	<=5cm	NA	NA	NA	NA	NA	Positive	No
1013	Yes	Laboratory Workflow Deviation	No	No	12.92	Male	Yes	5.51	Purebred	Maltese	0.0	1.0	Yes	No	NA	NA	NA	NA	NA	NA	NA	NA	NA	NA	Fail	No
1014	No	NA	No	Yes	14.17	Female	Yes	25.85	Mixed-breed	NA	1.0	1.0	Yes	No	Urinary Bladder/Urethra	NA	Localized/Regional	NA	<=5cm	Lymphoma, Intermediate to Large Cell	B-cell	NA	Disseminated/ Metastatic	<=5cm	Positive	Yes
1015	No	NA	No	Yes	7.08	Male	Yes	30.06	Mixed-breed	NA	2.5	0.5	Yes	No	Transmissible Venereal Tumor	NA	Localized/Regional	NA	>5cm	NA	NA	NA	NA	NA	Positive	No
1016	Yes	Enrollment or Clinical Deviation	No	No	7.25	Male	Yes	10.22	Purebred	Cavalier King Charles Spaniel	1.0	0.0	Yes	No	NA	NA	NA	NA	NA	NA	NA	NA	NA	NA	Positive	No
1017	No	NA	No	Yes	5.50	Female	Yes	16.43	Mixed-breed	NA	4.0	1.0	Yes	No	Lymphoma, Intermediate to Large Cell	T-Cell	NA	Disseminated/ Metastatic	<=5cm	NA	NA	NA	NA	NA	Negative	No
1018	No	NA	Yes	No	7.42	Male	Yes	33.27	Mixed-breed	NA	3.5	2.0	Yes	No	Mast Cell Tumor	NA	Localized/Regional	NA	<=5cm	NA	NA	NA	NA	NA	Negative	No
1019	No	NA	Yes	No	4.42	Male	Yes	24.35	Purebred	English Bulldog	3.0	2.0	Yes	No	Lymphoma, Intermediate to Large Cell	B-cell	NA	Disseminated/ Metastatic	>5cm	NA	NA	NA	NA	NA	Positive	Yes
1020	No	NA	No	Yes	8.33	Female	Yes	16.03	Mixed-breed	NA	4.0	2.0	Yes	No	Oral Cavity	NA	Localized/Regional	NA	>5cm	NA	NA	NA	NA	NA	Negative	No
1021	No	NA	No	Yes	9.17	Female	Yes	58.12	Purebred	Great Dane	2.0	1.0	Yes	No	Bone, Osteosarcoma	NA	Disseminated/Metastatic	NA	<=5cm	NA	NA	NA	NA	NA	Negative	No
1022	No	NA	No	Yes	7.92	Female	Yes	30.96	Mixed-breed	NA	2.0	1.0	Yes	No	Bone, Osteosarcoma	NA	Localized/Regional	NA	<=5cm	NA	NA	NA	NA	NA	Negative	No
1023	Yes	Enrollment or Clinical Deviation	No	No	9.50	Male	Yes	49.40	Purebred	Golden Retriever	2.0	1.0	Yes	No	NA	NA	NA	NA	NA	NA	NA	NA	NA	NA	Positive	No
1024	No	NA	No	Yes	11.00	Male	Yes	23.55	Mixed-breed	NA	3.5	2.0	Yes	No	Malignant Melanoma	NA	Localized/Regional	NA	<=5cm	NA	NA	NA	NA	NA	Positive	No
1025	No	NA	No	Yes	7.25	Male	Yes	32.07	Purebred	Boxer	4.0	2.0	Yes	No	Lymphoma, Intermediate to Large Cell	T-Cell	NA	Disseminated/ Metastatic	<=5cm	NA	NA	NA	NA	NA	Positive	No
1026	No	NA	No	Yes	11.83	Male	No	34.07	Purebred	Golden Retriever	3.5	2.0	Yes	No	Lymphoma, Intermediate to Large Cell	UNK	NA	Disseminated/ Metastatic	>5cm	NA	NA	NA	NA	NA	Positive	Yes
1027	No	NA	Yes	No	10.17	Male	Yes	10.02	Purebred	French Bulldog	1.0	2.0	Yes	No	Bone, Osteosarcoma	NA	Disseminated/Metastatic	NA	<=5cm	NA	NA	NA	NA	NA	Positive	No
1028	No	NA	No	Yes	10.17	Female	Yes	23.65	Purebred	English Bulldog	1.0	1.0	Yes	No	Lymphoma, Intermediate to Large Cell	UNK	NA	Disseminated/ Metastatic	>5cm	NA	NA	NA	NA	NA	Positive	Yes
1029	No	NA	Yes	No	5.00	Female	Yes	20.04	Mixed-breed	NA	4.0	2.0	Yes	No	Lymphoma, Intermediate to Large Cell	B-cell	NA	Disseminated/ Metastatic	>5cm	NA	NA	NA	NA	NA	Positive	No
1030	No	NA	No	Yes	7.00	Male	Yes	52.11	Purebred	Rottweiler	1.0	2.0	Yes	No	Malignant Melanoma	NA	Disseminated/Metastatic	NA	>5cm	NA	NA	NA	NA	NA	Positive	No
1031	No	NA	No	Yes	7.00	Male	No	35.27	Purebred	Labrador Retriever	4.0	2.0	Yes	No	Lymphoma, Intermediate to Large Cell	B-cell	NA	Disseminated/ Metastatic	<=5cm	NA	NA	NA	NA	NA	Positive	No
1032	No	NA	No	Yes	8.67	Male	Yes	38.18	Mixed-breed	NA	3.0	2.0	Yes	No	Lymphoma, Intermediate to Large Cell	T-Cell	NA	Disseminated/ Metastatic	>5cm	NA	NA	NA	NA	NA	Positive	No
1033	No	NA	No	Yes	13.42	Female	Yes	43.09	Purebred	German Shepherd	1.0	0.0	Yes	No	Hemangiosarcoma	NA	Localized/Regional	NA	>5cm	Thyroid	NA	Disseminated/ Metastatic	NA	>5cm	Positive	No
1034	Yes	Laboratory Workflow Deviation	No	No	12.58	Female	Yes	8.42	Purebred	Boston Terrier	2.0	0.5	Yes	No	NA	NA	NA	NA	NA	NA	NA	NA	NA	NA	Negative	No
1035	No	NA	Yes	No	10.00	Female	No	14.53	Purebred	English Setter	1.0	2.0	Yes	No	Lymphoma, Intermediate to Large Cell	UNK	NA	Disseminated/ Metastatic	>5cm	NA	NA	NA	NA	NA	Positive	No

Subject	Excluded	Reason for Exclusion	Training Set	Testing Set	Age	Sex	Spay/ Neuter	Weight (kg)	Purebred or Mixed Breed	Breed (Purebred)	Hemolysis	Lipemia	Cancer Diagnosis at Enrollment	Cancer Diagnosed after Liquid Biopsy	Cancer Type	Immunophenotype (Lymphoid Cancer)	Extent of Disease: Non-Lymphoid Cancer	Extent of Disease: Lymphoid Cancer	Tumor Size	Cancer Type (Cancer #2)	Immunophenotype (Cancer #2 if Lymphoid)	Extent of Disease: Non-Lymphoid Cancer (Cancer #2)	Extent of Disease: Lymphoid Cancer (Cancer #2)	Tumor Size (Cancer #2)	Liquid Biopsy Result	CSO Prediction of Hematological Malignancy
1036	No	NA	Yes	No	4.58	Female	No	11.82	Purebred	French Bulldog	4.0	NA	Yes	No	Lymphoma, Intermediate to Large Cell	UNK	NA	Disseminated/ Metastatic	<=5cm	NA	NA	NA	NA	NA	Positive	No
1037	No	NA	No	Yes	8.08	Male	Yes	26.56	Mixed-breed	NA	2.0	2.0	Yes	No	Lymphoma, Intermediate to Large Cell	B-cell	NA	Disseminated/ Metastatic	<=5cm	NA	NA	NA	NA	NA	Positive	No
1038	No	NA	No	Yes	9.58	Male	Yes	39.58	Purebred	Golden Retriever	2.0	1.0	Yes	No	Nasal Cavity and Paranasal Sinuses	NA	Localized/Regional	NA	>5cm	NA	NA	NA	NA	NA	Negative	No
1039	No	NA	No	Yes	5.50	Male	Yes	23.25	Purebred	Australian Shepherd	3.0	2.0	Yes	No	Lymphoma, Intermediate to Large Cell	UNK	NA	Disseminated/ Metastatic	>5cm	NA	NA	NA	NA	NA	Positive	No
1040	No	NA	No	Yes	9.83	Female	Yes	23.05	Purebred	Basset Hound	4.0	2.0	Yes	No	Nasal Cavity and Paranasal Sinuses	NA	Localized/Regional	NA	UNK	NA	NA	NA	NA	NA	Negative	No
1041	No	NA	No	Yes	5.92	Female	Yes	27.26	Mixed-breed	NA	3.5	1.0	Yes	No	Lymphoma, Intermediate to Large Cell	T-Cell	NA	Disseminated/ Metastatic	<=5cm	NA	NA	NA	NA	NA	Positive	No
1042	Yes	Enrollment or Clinical Deviation	No	No	10.50	Male	No	32.07	Purebred	Labrador Retriever	1.0	2.0	Yes	No	NA	NA	NA	NA	NA	NA	NA	NA	NA	NA	Negative	No
1043	No	NA	No	Yes	15.08	Female	Yes	19.84	Mixed-breed	NA	2.0	2.0	Yes	No	Lymphoma, Intermediate to Large Cell	B-cell	NA	Disseminated/ Metastatic	<=5cm	NA	NA	NA	NA	NA	Positive	Yes
1044	No	NA	No	Yes	11.75	Male	Yes	43.39	Mixed-breed	NA	2.0	2.0	Yes	No	Urinary Bladder/Urethra	NA	Localized/Regional	NA	<=5cm	Hemangio-sarcoma	NA	Localized/Regional	NA	>5cm	Positive	No
1045	No	NA	No	Yes	7.00	Male	Yes	37.08	Purebred	Golden Retriever	3.5	2.0	Yes	No	Lymphoma, Intermediate to Large Cell	T-Cell	NA	Localized/Re-gional	<=5cm	NA	NA	NA	NA	NA	Negative	No
1046	No	NA	No	Yes	4.00	Male	Yes	12.83	Purebred	Pembroke Welsh Corgi	3.0	1.0	Yes	No	Lymphoma, Intermediate to Large Cell	B-cell	NA	Disseminated/ Metastatic	<=5cm	NA	NA	NA	NA	NA	Positive	Yes
1047	No	NA	No	Yes	11.92	Female	Yes	27.06	Mixed-breed	NA	3.0	NA	Yes	No	Lymphoma, Intermediate to Large Cell	B-cell	NA	Disseminated/ Metastatic	<=5cm	NA	NA	NA	NA	NA	Positive	No
1048	Yes	Laboratory Workflow Deviation	No	No	12.83	Male	Yes	33.67	Purebred	American Staffordshire Terrier	3.5	2.0	Yes	No	Lymphoma, Indolent	T ZONE	NA	Disseminated/ Metastatic	<=5cm	NA	NA	NA	NA	NA	Negative	No
1049	No	NA	No	Yes	13.00	Male	No	23.15	Mixed-breed	NA	2.0	1.0	Yes	No	Pituitary	NA	Localized/Regional	NA	<=5cm	NA	NA	NA	NA	NA	Negative	No
1050	No	NA	No	Yes	10.00	Male	Yes	34.09	Mixed-breed	NA	4.0	2.0	Yes	No	Bone, Osteosarcoma	NA	Disseminated/Metastatic	NA	>5cm	NA	NA	NA	NA	NA	Positive	No
1051	No	NA	No	Yes	12.00	Female	Yes	25.55	Mixed-breed	NA	2.0	0.0	Yes	No	Skin	NA	Disseminated/Metastatic	NA	<=5cm	NA	NA	NA	NA	NA	Positive	No
1052	No	NA	No	Yes	7.50	Female	Yes	19.34	Purebred	Airedale Terrier	3.0	1.0	Yes	No	Lymphoma, Intermediate to Large Cell	B-cell	NA	Disseminated/ Metastatic	<=5cm	NA	NA	NA	NA	NA	Positive	Yes
1053	No	NA	No	Yes	12.17	Female	Yes	10.02	Purebred	Pembroke Welsh Corgi	1.5	2.0	Yes	No	Lymphoma, Intermediate to Large Cell	UNK	NA	Disseminated/ Metastatic	>5cm	NA	NA	NA	NA	NA	Negative	No
1054	No	NA	No	Yes	5.92	Female	Yes	27.06	Purebred	English Bulldog	2.0	1.0	Yes	No	Lymphoma, Intermediate to Large Cell	T-Cell	NA	Disseminated/ Metastatic	>5cm	NA	NA	NA	NA	NA	Positive	No
1055	No	NA	No	Yes	11.00	Male	Yes	30.76	Mixed-breed	NA	2.5	2.0	Yes	No	Soft Tissue Sarcoma	NA	Localized/Regional	NA	>5cm	NA	NA	NA	NA	NA	Negative	No
1056	Yes	Laboratory Workflow Deviation	No	No	6.33	Female	Yes	24.15	Mixed-breed	NA	4.0	2.0	Yes	No	Mast Cell Tumor	NA	Localized/Regional	NA	<=5cm	NA	NA	NA	NA	NA	Negative	No
1057	No	NA	No	Yes	8.00	Female	Yes	36.58	Mixed-breed	NA	2.0	2.0	Yes	No	Malignant Melanoma	NA	Localized/Regional	NA	<=5cm	NA	NA	NA	NA	NA	Negative	No
1058	No	NA	Yes	No	6.17	Female	Yes	13.13	Purebred	Cardigan Welsh Corgi	2.0	2.0	Yes	No	Lymphoma, Intermediate to Large Cell	T-Cell	NA	Localized/ Regional	>5cm	NA	NA	NA	NA	NA	Positive	No
1059	Yes	Laboratory Workflow Deviation	No	No	2.75	Female	Yes	35.57	Purebred	Golden Retriever	3.0	2.0	Yes	No	NA	NA	NA	NA	NA	NA	NA	NA	NA	NA	Negative	No
1060	Yes	Laboratory Workflow Deviation	No	No	4.33	Female	Yes	32.07	Purebred	Labrador Retriever	4.0	2.0	Yes	No	Soft Tissue Sarcoma	NA	Localized/Regional	NA	<=5cm	NA	NA	NA	NA	NA	Positive	No
1061	No	NA	Yes	No	12.00	Female	Yes	9.92	Mixed-breed	NA	3.0	2.0	Yes	No	Anal Sac	NA	Disseminated/Metastatic	NA	<=5cm	NA	NA	NA	NA	NA	Negative	No
1062	No	NA	No	Yes	2.58	Male	Yes	16.23	Purebred	Shetland Sheepdog	4.0	2.0	Yes	No	Lymphoma, Intermediate to Large Cell	T-Cell	NA	Localized/ Regional	>5cm	NA	NA	NA	NA	NA	Positive	No
1063	Yes	Laboratory Workflow Deviation	No	No	6.58	Male	Yes	51.01	Purebred	Great Pyrenees	3.0	2.0	Yes	No	NA	NA	NA	NA	NA	NA	NA	NA	NA	NA	Positive	No
1064	No	NA	No	Yes	8.42	Female	Yes	9.62	Mixed-breed	NA	3.0	2.0	Yes	No	Mast Cell Tumor	NA	Localized/Regional	NA	<=5cm	NA	NA	NA	NA	NA	Negative	No
1065	Yes	Test failure	No	No	7.00	Male	Yes	35.91	Mixed-breed	NA	NA	2.0	Yes	No	Mast Cell Tumor	NA	Localized/Regional	NA	<=5cm	NA	NA	NA	NA	NA	Negative	No
1066	Yes	Enrollment or Clinical Deviation	No	No	6.00	Male	Yes	30.86	Purebred	Labrador Retriever	0.0	1.0	Yes	No	NA	NA	NA	NA	NA	NA	NA	NA	NA	NA	Negative	No
1067	Yes	Laboratory Workflow Deviation	No	No	12.58	Female	Yes	16.94	Purebred	Australian Shepherd	2.0	2.0	Yes	No	NA	NA	NA	NA	NA	NA	NA	NA	NA	NA	Positive	No
1068	Yes	Enrollment or Clinical Deviation	No	No	9.00	Male	Yes	34.87	Purebred	Golden Retriever	0.0	2.0	Yes	No	NA	NA	NA	NA	NA	NA	NA	NA	NA	NA	Negative	No
1069	No	NA	No	Yes	9.92	Male	Yes	11.82	Purebred	Shih Tzu	3.0	1.0	Yes	No	Mast Cell Tumor	NA	Localized/Regional	NA	<=5cm	Leukemia, Chronic Lymphoid (CLL)	B-cell	NA	Undetermined	NA	Positive	Yes
1070	No	NA	No	Yes	8.58	Male	Yes	30.56	Mixed-breed	NA	4.0	2.0	Yes	No	Anal Sac	NA	Localized/Regional	NA	<=5cm	NA	NA	NA	NA	NA	Negative	No
1071	No	NA	No	Yes	8.92	Male	Yes	32.07	Mixed-breed	NA	NA	1.0	Yes	No	Oral Cavity	NA	Localized/Regional	NA	>5cm	NA	NA	NA	NA	NA	Positive	No
1072	No	NA	No	Yes	8.83	Male	Yes	44.39	Purebred	Dogo Argentino	3.0	2.0	Yes	No	Bone, Osteosarcoma	NA	Localized/Regional	NA	UNK	NA	NA	NA	NA	NA	Negative	No
1073	No	NA	No	Yes	12.83	Male	Yes	25.35	Mixed-breed	NA	3.0	NA	Yes	No	Oral Cavity	NA	Localized/Regional	NA	<=5cm	NA	NA	NA	NA	NA	Negative	No
1074	Yes	Enrollment or Clinical Deviation	No	No	10.50	Female	Yes	20.54	Mixed-breed	NA	0.0	2.0	Yes	No	NA	NA	NA	NA	NA	NA	NA	NA	NA	NA	Negative	No
1075	No	NA	Yes	No	11.08	Male	Yes	29.96	Mixed-breed	NA	3.0	1.0	Yes	No	Bone, Multilobular Osteochondrosarcoma	NA	Localized/Regional	NA	<=5cm	NA	NA	NA	NA	NA	Negative	No
1076	No	NA	No	Yes	15.75	Female	Yes	24.95	Purebred	Siberian Husky	3.0	2.0	Yes	No	Mammary Gland Carcinoma	NA	Disseminated/Metastatic	NA	>5cm	NA	NA	NA	NA	NA	Positive	No
1077	No	NA	Yes	No	8.75	Female	Yes	37.38	Purebred	Labrador Retriever	4.0	2.0	Yes	No	Oral Cavity	NA	Localized/Regional	NA	<=5cm	NA	NA	NA	NA	NA	Negative	No
1078	Yes	Laboratory Workflow Deviation	No	No	12.50	Male	Yes	38.08	Mixed-breed	NA	1.0	2.0	Yes	No	NA	NA	NA	NA	NA	NA	NA	NA	NA	NA	Positive	No
1079	Yes	Laboratory Workflow Deviation	No	No	3.75	Male	Yes	7.52	Purebred	Dachshund	4.0	1.0	Yes	No	NA	NA	NA	NA	NA	NA	NA	NA	NA	NA	Negative	No
1080	No	NA	Yes	No	11.00	Male	Yes	34.07	Purebred	Collie	4.0	2.0	Yes	No	Soft Tissue Sarcoma	NA	Localized/Regional	NA	>5cm	NA	NA	NA	NA	NA	Negative	No
1081	No	NA	No	Yes	11.50	Female	Yes	26.46	Mixed-breed	NA	1.0	1.0	Yes	No	Anal Sac	NA	Disseminated/Metastatic	NA	<=5cm	Mammary Gland Carcinoma	NA	Localized/Regional	NA	<=5cm	Negative	No
1082	Yes	Enrollment or Clinical Deviation	No	No	10.75	Female	Yes	22.05	Mixed-breed	NA	2.5	1.5	Yes	No	NA	NA	NA	NA	NA	NA	NA	NA	NA	NA	Negative	No
1083	No	NA	No	Yes	11.67	Male	Yes	26.05	Mixed-breed	NA	2.0	NA	Yes	No	Soft Tissue Sarcoma	NA	Localized/Regional	NA	>5cm	NA	NA	NA	NA	NA	Negative	No
1084	No	NA	Yes	No	10.08	Male	Yes	32.37	Mixed-breed	NA	2.0	NA	Yes	No	Soft Tissue Sarcoma	NA	Localized/Regional	NA	<=5cm	NA	NA	NA	NA	NA	Negative	No
1085	No	NA	No	Yes	6.00	Male	Yes	50.91	Purebred	St. Bernard	2.0	2.0	Yes	No	Bone, Osteosarcoma	NA	Localized/Regional	NA	<=5cm	NA	NA	NA	NA	NA	Positive	No
1086	Yes	Laboratory Workflow Deviation	No	No	8.17	Female	Yes	10.32	Purebred	Cocker Spaniel	2.0	1.0	Yes	No	NA	NA	NA	NA	NA	NA	NA	NA	NA	NA	Positive	No
1087	No	NA	No	Yes	6.92	Female	Yes	62.13	Purebred	Great Dane	4.0	2.0	Yes	No	Bone, Osteosarcoma	NA	Localized/Regional	NA	>5cm	NA	NA	NA	NA	NA	Negative	No

S1 Table. Full subject level data for subjects enrolled in the CANDiD study (continued)

See legend on last page.

Subject	Excluded	Reason for Exclusion	Training Set	Testing Set	Age	Sex	Spay/ Neuter	Weight (kg)	Purebred or Mixed Breed	Breed (Purebred)	Hemolysis	Lipemia	Cancer Diagnosis at Enrollment	Cancer Diagnosed after Liquid Biopsy	Cancer Type	Immunophenotype (Lymphoid Cancer)	Extent of Disease: Non-Lymphoid Cancer	Extent of Disease: Lymphoid Cancer	Tumor Size	Cancer Type (Cancer #2)	Immunophenotype (Cancer #2 if Lymphoid)	Extent of Disease: Non-Lymphoid Cancer (Cancer #2)	Extent of Disease: Lymphoid Cancer (Cancer #2)	Tumor Size (Cancer #2)	Liquid Biopsy Result	CSO Prediction of Hematological Malignancy
1088	Yes	Laboratory Workflow Deviation	No	No	12.67	Female	Yes	11.62	Purebred	Cocker Spaniel	2.0	1.0	Yes	No	NA	NA	NA	NA	NA	NA	NA	NA	NA	NA	Positive	No
1089	No	NA	No	Yes	11.33	Female	Yes	16.43	Mixed-breed	NA	4.0	2.0	Yes	No	Large intestine	NA	Localized/Regional	NA	<=5cm	NA	NA	NA	NA	NA	Negative	No
1090	No	NA	No	Yes	15.50	Male	Yes	20.04	Mixed-breed	NA	2.0	1.0	Yes	No	Malignant Melanoma	NA	Localized/Regional	NA	<=5cm	Soft Tissue Sarcoma	NA	Localized/Regional	NA	<=5cm	Negative	No
1091	Yes	Laboratory Workflow Deviation	No	No	13.58	Male	Yes	9.02	Purebred	Havanese	1.0	1.0	Yes	No	NA	NA	NA	NA	NA	NA	NA	NA	NA	NA	Negative	No
1092	Yes	Laboratory Workflow Deviation	No	No	10.75	Male	UNK	6.31	Mixed-breed	NA	4.0	2.0	Yes	No	NA	NA	NA	NA	NA	NA	NA	NA	NA	NA	Negative	No
1093	Yes	Enrollment or Clinical Deviation	No	No	2.33	Male	Yes	32.27	Purebred	Labrador Retriever	3.0	2.0	Yes	No	NA	NA	NA	NA	NA	NA	NA	NA	NA	NA	Negative	No
1094	Yes	Laboratory Workflow Deviation	No	No	11.67	Female	Yes	34.07	Purebred	Labrador Retriever	NA	1.0	Yes	No	NA	NA	NA	NA	NA	NA	NA	NA	NA	NA	Negative	No
1095	Yes	Laboratory Workflow Deviation	No	No	13.50	Female	Yes	34.07	Mixed-breed	NA	2.0	2.0	Yes	No	NA	NA	NA	NA	NA	NA	NA	NA	NA	NA	Positive	No
1096	No	NA	Yes	No	9.33	Male	Yes	46.10	Purebred	Boxer	3.5	2.0	Yes	No	Soft Tissue Sarcoma	NA	Localized/Regional	NA	>5cm	NA	NA	NA	NA	NA	Negative	No
1097	Yes	Enrollment or Clinical Deviation	No	No	13.08	Male	Yes	35.47	Purebred	Labrador Retriever	3.0	2.0	Yes	No	NA	NA	NA	NA	NA	NA	NA	NA	NA	NA	Negative	No
1098	Yes	Laboratory Workflow Deviation	No	No	13.58	Male	Yes	8.32	Purebred	Cavalier King Charles Spaniel	4.0	2.0	Yes	No	NA	NA	NA	NA	NA	NA	NA	NA	NA	NA	Negative	No
1099	Yes	Laboratory Workflow Deviation	No	No	7.33	Female	Yes	8.72	Purebred	Shih Tzu	3.0	2.0	Yes	No	NA	NA	NA	NA	NA	NA	NA	NA	NA	NA	Negative	No
1100	Yes	Laboratory Workflow Deviation	No	No	13.42	Male	Yes	9.32	Purebred	Russell Terrier	4.0	2.0	Yes	No	NA	NA	NA	NA	NA	NA	NA	NA	NA	NA	Negative	No
1101	Yes	Laboratory Workflow Deviation	No	No	8.58	Female	Yes	48.40	Purebred	Bernese Mountain Dogs	3.0	2.0	Yes	No	NA	NA	NA	NA	NA	NA	NA	NA	NA	NA	Negative	No
1102	No	NA	No	Yes	7.25	Male	Yes	32.47	Purebred	Chesapeake Bay Retriever	2.0	1.0	Yes	No	Oral Cavity	NA	Disseminated/Metastatic	NA	>5cm	NA	NA	NA	NA	NA	Negative	No
1103	Yes	Laboratory Workflow Deviation	No	No	13.75	Female	Yes	4.51	Purebred	Maltese	4.0	2.0	Yes	No	Spinal Cord	NA	Localized/Regional	NA	<=5cm	NA	NA	NA	NA	NA	Negative	No
1104	Yes	Laboratory Workflow Deviation	No	No	5.25	Male	Yes	23.55	Mixed-breed	NA	3.0	1.0	Yes	No	NA	NA	NA	NA	NA	NA	NA	NA	NA	NA	Positive	No
1105	Yes	Laboratory Workflow Deviation	No	No	8.92	Male	Yes	41.79	Purebred	Labrador Retriever	2.0	1.0	Yes	No	NA	NA	NA	NA	NA	NA	NA	NA	NA	NA	Negative	No
1106	No	NA	No	Yes	9.58	Female	Yes	14.23	Purebred	Kerry Blue Terrier	3.0	2.0	Yes	No	Urinary Bladder/Urethra	NA	Localized/Regional	NA	<=5cm	Oral Cavity	NA	Localized/Regional	NA	<=5cm	Negative	No
1107	No	NA	No	Yes	8.67	Female	No	28.36	Purebred	Labrador Retriever	2.0	2.0	Yes	No	Lung	NA	Localized/Regional	NA	>5cm	NA	NA	NA	NA	NA	Negative	No
1108	No	NA	No	Yes	12.42	Female	Yes	27.36	Mixed-breed	NA	4.0	2.0	Yes	No	Bone, Osteosarcoma	NA	Localized/Regional	NA	<=5cm	NA	NA	NA	NA	NA	Positive	No
1109	Yes	Test failure	No	No	7.00	Male	No	11.02	Purebred	Boston Terrier	4.0	2.0	Yes	No	Hemangiosarcoma	NA	Localized/Regional	NA	<=5cm	Mast Cell Tumor	NA	Localized/Regional	NA	<=5cm	Negative	No
1110	Yes	Laboratory Workflow Deviation	No	No	5.00	Female	Yes	36.08	Purebred	Rhodesian Ridgeback	3.0	NA	Yes	No	NA	NA	NA	NA	NA	NA	NA	NA	NA	NA	Negative	No
1111	Yes	Laboratory Workflow Deviation	No	No	12.00	Female	Yes	24.95	Mixed-breed	NA	3.0	2.0	Yes	No	NA	NA	NA	NA	NA	NA	NA	NA	NA	NA	Positive	No
1112	Yes	Laboratory Workflow Deviation	No	No	10.67	Male	Yes	16.63	Mixed-breed	NA	3.0	2.0	Yes	No	NA	NA	NA	NA	NA	NA	NA	NA	NA	NA	Negative	No
1113	No	NA	No	Yes	10.00	Female	Yes	38.38	Mixed-breed	NA	2.0	1.0	Yes	No	Bone, Osteosarcoma	NA	Localized/Regional	NA	UNK	NA	NA	NA	NA	NA	Positive	No
1114	Yes	Laboratory Workflow Deviation	No	No	10.08	Female	No	33.27	Mixed-breed	NA	3.0	NA	Yes	No	NA	NA	NA	NA	NA	NA	NA	NA	NA	NA	Negative	No
1115	Yes	Enrollment or Clinical Deviation	No	No	9.58	Male	Yes	30.26	Mixed-breed	NA	2.0	2.0	Yes	No	NA	NA	NA	NA	NA	NA	NA	NA	NA	NA	Negative	No
1116	No	NA	No	Yes	10.17	Male	Yes	35.27	Purebred	German Shepherd	2.0	0.0	Yes	No	Pancreas, Endocrine	NA	Disseminated/Metastatic	NA	>5cm	NA	NA	NA	NA	NA	Negative	No
1117	Yes	Laboratory Workflow Deviation	No	No	10.00	Male	Yes	9.12	Purebred	American Eskimo Dog	4.0	NA	Yes	No	NA	NA	NA	NA	NA	NA	NA	NA	NA	NA	Negative	No
1118	Yes	Laboratory Workflow Deviation	No	No	14.17	Male	Yes	6.31	Purebred	Poodle, Toy	3.0	2.0	Yes	No	NA	NA	NA	NA	NA	NA	NA	NA	NA	NA	Negative	No
1119	No	NA	No	Yes	7.83	Male	No	29.56	Purebred	Flat-Coated Retriever	2.0	1.0	Yes	No	Oral Cavity	NA	Undetermined	NA	<=5cm	NA	NA	NA	NA	NA	Negative	No
1120	Yes	Laboratory Workflow Deviation	No	No	14.67	Female	Yes	11.72	Mixed-breed	NA	3.0	2.0	Yes	No	NA	NA	NA	NA	NA	NA	NA	NA	NA	NA	Negative	No
1121	No	NA	No	Yes	4.17	Male	Yes	14.03	Purebred	Cocker Spaniel	2.0	1.5	Yes	No	Mast Cell Tumor	NA	Localized/Regional	NA	<=5cm	NA	NA	NA	NA	NA	Negative	No
1122	No	NA	Yes	No	8.08	Male	Yes	29.76	Purebred	Boxer	3.0	2.0	Yes	No	Mast Cell Tumor	NA	Localized/Regional	NA	<=5cm	NA	NA	NA	NA	NA	Negative	No
1123	No	NA	No	Yes	8.83	Female	Yes	26.76	Purebred	Labrador Retriever	2.5	2.0	Yes	No	Mast Cell Tumor	NA	Localized/Regional	NA	>5cm	NA	NA	NA	NA	NA	Negative	No
1124	Yes	Enrollment or Clinical Deviation	No	No	5.00	Male	Yes	19.04	Mixed-breed	NA	2.5	1.0	Yes	No	NA	NA	NA	NA	NA	NA	NA	NA	NA	NA	Negative	No
1125	No	NA	No	Yes	9.67	Male	Yes	27.46	Purebred	Labrador Retriever	2.0	1.0	Yes	No	Soft Tissue Sarcoma	NA	Localized/Regional	NA	<=5cm	NA	NA	NA	NA	NA	Negative	No
1126	No	NA	No	Yes	7.00	Female	Yes	27.36	Purebred	Labrador Retriever	3.5	1.5	Yes	No	Mast Cell Tumor	NA	Disseminated/Metastatic	NA	>5cm	NA	NA	NA	NA	NA	Negative	No
1127	Yes	Laboratory Workflow Deviation	No	No	7.83	Female	Yes	24.75	Purebred	Siberian Husky	3.0	2.0	Yes	No	NA	NA	NA	NA	NA	NA	NA	NA	NA	NA	Fail	No
1128	No	NA	No	Yes	10.17	Female	Yes	36.88	Mixed-breed	NA	2.0	2.0	Yes	No	Oral Cavity	NA	Localized/Regional	NA	>5cm	NA	NA	NA	NA	NA	Positive	No
1129	No	NA	No	Yes	10.42	Male	Yes	38.38	Mixed-breed	NA	1.5	2.0	Yes	No	Bone, Osteosarcoma	NA	Localized/Regional	NA	<=5cm	NA	NA	NA	NA	NA	Positive	No
1130	No	NA	No	Yes	11.00	Female	Yes	36.58	Mixed-breed	NA	2.0	2.0	Yes	No	Bone, Osteosarcoma	NA	Localized/Regional	NA	UNK	NA	NA	NA	NA	NA	Positive	No
1131	No	NA	No	Yes	10.83	Female	Yes	10.22	Purebred	West Highland White Terrier	2.5	2.0	Yes	No	Soft Tissue Sarcoma	NA	Localized/Regional	NA	<=5cm	NA	NA	NA	NA	NA	Negative	No
1132	No	NA	No	Yes	12.83	Female	Yes	19.04	Purebred	Brittany	3.0	2.0	Yes	No	Soft Tissue Sarcoma	NA	Localized/Regional	NA	>5cm	NA	NA	NA	NA	NA	Negative	No
1133	Yes	Laboratory Workflow Deviation	No	No	12.25	Female	Yes	5.11	Mixed-breed	NA	3.0	1.0	Yes	No	NA	NA	NA	NA	NA	NA	NA	NA	NA	NA	Negative	No
1134	No	NA	No	Yes	9.83	Male	Yes	22.35	Mixed-breed	NA	3.0	2.0	Yes	No	Oral Cavity	NA	Localized/Regional	NA	<=5cm	NA	NA	NA	NA	NA	Negative	No
1135	No	NA	No	Yes	8.75	Male	Yes	42.09	Purebred	Boxer	3.0	2.0	Yes	No	Mast Cell Tumor	NA	Localized/Regional	NA	<=5cm	Soft Tissue Sarcoma	NA	Localized/Regional	NA	<=5cm	Negative	No
1136	No	NA	No	Yes	11.33	Male	Yes	36.08	Mixed-breed	NA	2.0	1.0	Yes	No	Oral Cavity	NA	Localized/Regional	NA	<=5cm	NA	NA	NA	NA	NA	Positive	No
1137	No	NA	No	Yes	9.08	Male	Yes	29.46	Purebred	Flat-Coated Retriever	1.0	2.0	Yes	No	Soft Tissue Sarcoma	NA	Localized/Regional	NA	<=5cm	NA	NA	NA	NA	NA	Negative	No
1138	Yes	Enrollment or Clinical Deviation	No	No	13.17	Male	Yes	14.23	Mixed-breed	NA	3.0	2.0	Yes	No	NA	NA	NA	NA	NA	NA	NA	NA	NA	NA	Negative	No
1139	No	NA	No	Yes	7.33	Male	Yes	24.75	Mixed-breed	NA	1.0	1.0	Yes	No	Thyroid	NA	Localized/Regional	NA	<=5cm	NA	NA	NA	NA	NA	Negative	No
1140	No	NA	Yes	No	10.08	Male	No	33.97	Mixed-breed	NA	1.0	1.5	Yes	No	Bile Duct	NA	Disseminated/Metastatic	NA	>5cm	NA	NA	NA	NA	NA	Positive	No
1141	No	NA	No	Yes	14.67	Female	Yes	8.32	Purebred	Dachshund	4.0	2.0	Yes	No	Liver	NA	Localized/Regional	NA	>5cm	NA	NA	NA	NA	NA	Negative	No
1142	No	NA	No	Yes	14.50	Female	Yes	17.44	Mixed-breed	NA	4.0	2.0	Yes	No	Anal Sac	NA	Localized/Regional	NA	<=5cm	NA	NA	NA	NA	NA	Positive	No
1143	No	NA	No	Yes	9.42	Female	Yes	8.92	Purebred	Pug	2.0	2.0	Yes	No	Mast Cell Tumor	NA	Localized/Regional	NA	<=5cm	Mast Cell Tumor	NA	Localized/Regional	NA	<=5cm	Negative	No

S1 Table. Full subject level data for subjects enrolled in the CANDiD study (continued)

See legend on last page.

Subject	Excluded	Reason for Exclusion	Training Set	Testing Set	Age	Sex	Spay/ Neuter	Weight (kg)	Purebred or Mixed Breed	Breed (Purebred)	Hemolysis	Lipemia	Cancer Diagnosis at Enrollment	Cancer Diagnosed after Liquid Biopsy	Cancer Type	Immunophenotype (Lymphoid Cancer)	Extent of Disease: Non-Lymphoid Cancer	Extent of Disease: Lymphoid Cancer	Tumor Size	Cancer Type (Cancer #2)	Immunophenotype (Cancer #2 if Lymphoid)	Extent of Disease: Non-Lymphoid Cancer (Cancer #2)	Extent of Disease: Lymphoid Cancer (Cancer #2)	Tumor Size (Cancer #2)	Liquid Biopsy Result	CSO Prediction of Hematological Malignancy
1144	Yes	Laboratory Workflow Deviation	No	No	9.08	Male	Yes	38.08	Mixed-breed	NA	0.0	1.0	Yes	No	NA	NA	NA	NA	NA	NA	NA	NA	NA	NA	Positive	No
1145	No	NA	No	Yes	10.58	Female	Yes	39.38	Mixed-breed	NA	3.0	2.0	Yes	No	Soft Tissue Sarcoma	NA	Localized/Regional	NA	>5cm	NA	NA	NA	NA	NA	Negative	No
1146	No	NA	Yes	No	9.67	Male	Yes	72.15	Purebred	Great Dane	3.5	NA	Yes	No	Soft Tissue Sarcoma	NA	Localized/Regional	NA	>5cm	NA	NA	NA	NA	NA	Negative	No
1147	No	NA	No	Yes	5.00	Male	Yes	33.57	Mixed-breed	NA	1.0	1.0	Yes	No	Nasal Cavity and Paranasal Sinuses	NA	Localized/Regional	NA	>5cm	NA	NA	NA	NA	NA	Positive	No
1148	No	NA	No	Yes	8.83	Male	Yes	33.47	Mixed-breed	NA	2.0	1.0	Yes	No	Nasal Cavity and Paranasal Sinuses	NA	Localized/Regional	NA	>5cm	NA	NA	NA	NA	NA	Negative	No
1149	Yes	Laboratory Workflow Deviation	No	No	11.00	Male	Yes	20.84	Mixed-breed	NA	3.5	1.5	Yes	No	Mast Cell Tumor	NA	Localized/Regional	NA	<=5cm	NA	NA	NA	NA	NA	Negative	No
1150	Yes	Enrollment or Clinical Deviation	No	No	6.00	Male	Yes	35.37	Purebred	Labrador Retriever	2.5	2.0	Yes	No	NA	NA	NA	NA	NA	NA	NA	NA	NA	NA	Negative	No
1151	Yes	Laboratory Workflow Deviation	No	No	10.50	Female	Yes	20.44	Purebred	Australian Cattle Dog	4.0	1.0	Yes	No	NA	NA	NA	NA	NA	NA	NA	NA	NA	NA	Negative	No
1152	Yes	Enrollment or Clinical Deviation	No	No	6.83	Female	Yes	26.05	Purebred	Siberian Husky	3.0	2.0	Yes	No	NA	NA	NA	NA	NA	NA	NA	NA	NA	NA	Negative	No
1153	No	NA	No	Yes	2.67	Female	Yes	44.59	Purebred	Bernese Mountain Dogs	1.5	2.0	Yes	No	Hemangiosarcoma	NA	Localized/Regional	NA	>5cm	NA	NA	NA	NA	NA	Positive	No
1154	Yes	Laboratory Workflow Deviation	No	No	12.25	Female	Yes	23.35	Mixed-breed	NA	2.0	1.0	Yes	No	NA	NA	NA	NA	NA	NA	NA	NA	NA	NA	Negative	No
1155	No	NA	No	Yes	11.00	Female	Yes	49.90	Purebred	Great Dane	4.0	2.0	Yes	No	Bone, Osteosarcoma	NA	Localized/Regional	NA	UNK	NA	NA	NA	NA	NA	Positive	No
1156	Yes	Laboratory Workflow Deviation	No	No	8.50	Female	Yes	19.04	Mixed-breed	NA	2.0	1.0	Yes	No	NA	NA	NA	NA	NA	NA	NA	NA	NA	NA	Negative	No
1157	Yes	Laboratory Workflow Deviation	No	No	10.75	Male	Yes	37.78	Purebred	Golden Retriever	2.0	1.0	Yes	No	NA	NA	NA	NA	NA	NA	NA	NA	NA	NA	Negative	No
1158	Yes	Laboratory Workflow Deviation	No	No	7.58	Female	Yes	22.25	Mixed-breed	NA	1.0	1.0	Yes	No	NA	NA	NA	NA	NA	NA	NA	NA	NA	NA	Positive	No
1159	Yes	Laboratory Workflow Deviation	No	No	9.00	Female	Yes	22.95	Purebred	Brittany	NA	1.0	Yes	No	NA	NA	NA	NA	NA	NA	NA	NA	NA	NA	Negative	No
1160	No	NA	No	Yes	8.00	Female	Yes	28.86	Purebred	Labrador Retriever	1.0	2.0	Yes	No	Mast Cell Tumor	NA	Localized/Regional	NA	<=5cm	NA	NA	NA	NA	NA	Negative	No
1161	Yes	Laboratory Workflow Deviation	No	No	7.17	Male	Yes	46.30	Mixed-breed	NA	2.0	2.0	Yes	No	NA	NA	NA	NA	NA	NA	NA	NA	NA	NA	Negative	No
1162	Yes	Laboratory Workflow Deviation	No	No	8.67	Male	Yes	27.06	Purebred	Golden Retriever	0.0	2.0	Yes	No	NA	NA	NA	NA	NA	NA	NA	NA	NA	NA	Positive	No
1163	Yes	Laboratory Workflow Deviation	No	No	10.75	Male	Yes	12.03	Mixed-breed	NA	4.0	2.0	Yes	No	NA	NA	NA	NA	NA	NA	NA	NA	NA	NA	Negative	No
1164	No	NA	No	Yes	8.00	Male	Yes	27.86	Mixed-breed	NA	0.0	2.0	Yes	No	Liver	NA	Localized/Regional	NA	>5cm	NA	NA	NA	NA	NA	Negative	No
1165	No	NA	No	Yes	9.00	Male	Yes	36.88	Purebred	Labrador Retriever	3.5	2.0	Yes	No	Soft Tissue Sarcoma	NA	Localized/Regional	NA	>5cm	NA	NA	NA	NA	NA	Positive	No
1166	No	NA	No	Yes	9.00	Male	Yes	36.58	Mixed-breed	NA	3.0	2.0	Yes	No	Mast Cell Tumor	NA	Localized/Regional	NA	<=5cm	NA	NA	NA	NA	NA	Negative	No
1167	No	NA	No	Yes	11.67	Male	Yes	33.87	Purebred	Golden Retriever	1.5	1.0	Yes	No	Bone, Osteosarcoma	NA	Localized/Regional	NA	UNK	NA	NA	NA	NA	NA	Negative	No
1168	No	NA	No	Yes	13.08	Male	No	32.07	Mixed-breed	NA	4.0	2.0	Yes	No	Anal Sac	NA	Localized/Regional	NA	<=5cm	NA	NA	NA	NA	NA	Negative	No
1169	No	NA	No	Yes	6.83	Male	No	27.06	Purebred	Bull Terrier	2.0	2.0	Yes	No	Mast Cell Tumor	NA	Localized/Regional	NA	<=5cm	NA	NA	NA	NA	NA	Negative	No
1170	Yes	Enrollment or Clinical Deviation	No	No	5.08	Female	Yes	24.05	Mixed-breed	NA	4.0	2.0	Yes	No	NA	NA	NA	NA	NA	NA	NA	NA	NA	NA	Negative	No
1171	No	NA	No	Yes	4.25	Female	Yes	15.73	Purebred	Anatolian Shepherd	4.0	2.0	Yes	No	Skin	NA	Disseminated/Metastatic	NA	<=5cm	NA	NA	NA	NA	NA	Positive	No
1172	No	NA	No	Yes	1.92	Male	No	36.78	Mixed-breed	NA	0.0	1.0	Yes	No	Hemangiosarcoma	NA	Undetermined	NA	>5cm	NA	NA	NA	NA	NA	Positive	No
1173	Yes	Enrollment or Clinical Deviation	No	No	7.25	Male	Yes	33.47	Mixed-breed	NA	4.0	2.0	Yes	No	NA	NA	NA	NA	NA	NA	NA	NA	NA	NA	Negative	No
1174	No	NA	Yes	No	7.75	Male	Yes	24.55	Purebred	American Staffordshire Terrier	2.5	2.0	Yes	No	Bone, Osteosarcoma	NA	Localized/Regional	NA	>5cm	NA	NA	NA	NA	NA	Positive	No
1175	Yes	Laboratory Workflow Deviation	No	No	11.50	Male	Yes	31.37	Mixed-breed	NA	3.0	1.0	Yes	No	NA	NA	NA	NA	NA	NA	NA	NA	NA	NA	Negative	No
1176	Yes	Test failure	No	No	7.17	Male	Yes	25.45	Mixed-breed	NA	4.0	NA	Yes	No	Soft Tissue Sarcoma	NA	Localized/Regional	NA	UNK	NA	NA	NA	NA	NA	Negative	No
1177	Yes	Laboratory Workflow Deviation	No	No	10.08	Female	Yes	28.86	Mixed-breed	NA	4.0	1.0	Yes	No	NA	NA	NA	NA	NA	NA	NA	NA	NA	NA	Negative	No
1178	No	NA	No	Yes	10.58	Male	Yes	29.96	Mixed-breed	NA	4.0	2.0	Yes	No	Mast Cell Tumor	NA	Disseminated/Metastatic	NA	>5cm	NA	NA	NA	NA	NA	Negative	No
1179	No	NA	No	Yes	5.00	Female	No	23.45	Purebred	Boxer	3.0	2.0	Yes	No	Mast Cell Tumor	NA	Localized/Regional	NA	<=5cm	NA	NA	NA	NA	NA	Negative	No
1180	No	NA	Yes	No	7.17	Female	Yes	26.05	Mixed-breed	NA	1.5	2.0	Yes	No	Bone, Osteosarcoma	NA	Localized/Regional	NA	NA	NA	NA	NA	NA	NA	Positive	No
1181	No	NA	Yes	No	7.83	Female	Yes	30.06	Mixed-breed	NA	3.0	2.0	Yes	No	Soft Tissue Sarcoma	NA	Localized/Regional	NA	<=5cm	NA	NA	NA	NA	NA	Negative	No
1182	Yes	Laboratory Workflow Deviation	No	No	8.00	Male	Yes	28.86	Mixed-breed	NA	3.0	NA	Yes	No	NA	NA	NA	NA	NA	NA	NA	NA	NA	NA	Negative	No
1183	Yes	Laboratory Workflow Deviation	No	No	11.42	Male	Yes	21.54	Mixed-breed	NA	2.0	1.0	Yes	No	NA	NA	NA	NA	NA	NA	NA	NA	NA	NA	Negative	No
1184	Yes	Enrollment or Clinical Deviation	No	No	14.17	Female	Yes	12.53	Mixed-breed	NA	0.0	1.0	Yes	No	NA	NA	NA	NA	NA	NA	NA	NA	NA	NA	Positive	No
1185	No	NA	Yes	No	13.92	Male	Yes	8.72	Mixed-breed	NA	2.0	2.0	Yes	No	Anal Sac	NA	Localized/Regional	NA	<=5cm	NA	NA	NA	NA	NA	Negative	No
1186	No	NA	No	Yes	12.92	Female	Yes	22.95	Mixed-breed	NA	1.5	1.0	Yes	No	Soft Tissue Sarcoma	NA	Undetermined	NA	>5cm	NA	NA	NA	NA	NA	Positive	No
1187	No	NA	No	Yes	7.67	Male	No	15.43	Mixed-breed	NA	0.5	1.0	Yes	No	Mast Cell Tumor	NA	Localized/Regional	NA	<=5cm	NA	NA	NA	NA	NA	Positive	No
1188	No	NA	No	Yes	12.00	Female	Yes	38.08	Mixed-breed	NA	1.0	1.0	Yes	No	Lung	NA	Localized/Regional	NA	<=5cm	NA	NA	NA	NA	NA	Positive	No
1189	No	NA	Yes	No	11.25	Female	Yes	31.06	Purebred	Siberian Husky	1.5	1.5	Yes	No	Thyroid	NA	Localized/Regional	NA	<=5cm	NA	NA	NA	NA	NA	Negative	No
1190	No	NA	No	Yes	9.08	Female	Yes	26.56	Mixed-breed	NA	2.0	1.0	Yes	No	Skin	NA	Localized/Regional	NA	>5cm	NA	NA	NA	NA	NA	Negative	No
1191	No	NA	No	Yes	11.00	Female	Yes	34.07	Purebred	Boxer	3.5	2.0	Yes	No	Soft Tissue Sarcoma	NA	Localized/Regional	NA	<=5cm	NA	NA	NA	NA	NA	Negative	No
1192	No	NA	No	Yes	8.83	Female	No	42.09	Purebred	German Shepherd	2.0	2.0	Yes	No	Mammary Gland Carcinoma	NA	Localized/Regional	NA	>5cm	NA	NA	NA	NA	NA	Positive	No
1193	No	NA	No	Yes	11.92	Male	Yes	30.06	Mixed-breed	NA	1.0	2.0	Yes	No	Malignant Melanoma	NA	Localized/Regional	NA	>5cm	NA	NA	NA	NA	NA	Negative	No
1194	Yes	Enrollment or Clinical Deviation	No	No	8.00	Male	Yes	21.65	Mixed-breed	NA	1.0	1.0	Yes	No	NA	NA	NA	NA	NA	NA	NA	NA	NA	NA	Positive	No
1195	Yes	Test failure	No	No	4.08	Male	Yes	17.84	Mixed-breed	NA	3.0	2.0	Yes	No	Mast Cell Tumor	NA	Localized/Regional	NA	<=5cm	NA	NA	NA	NA	NA	Fail	No
1196	No	NA	No	Yes	13.58	Male	Yes	13.53	Mixed-breed	NA	0.5	1.5	Yes	No	Soft Tissue Sarcoma	NA	Localized/Regional	NA	>5cm	NA	NA	NA	NA	NA	Negative	No
1197	No	NA	No	Yes	8.17	Female	Yes	28.66	Mixed-breed	NA	1.0	2.0	Yes	No	Soft Tissue Sarcoma	NA	Localized/Regional	NA	>5cm	NA	NA	NA	NA	NA	Negative	No
1198	No	NA	Yes	No	11.00	Female	Yes	30.66	Mixed-breed	NA	2.0	2.0	Yes	No	Soft Tissue Sarcoma	NA	Localized/Regional	NA	>5cm	NA	NA	NA	NA	NA	Negative	No
1199	No	NA	No	Yes	4.83	Male	Yes	47.70	Purebred	Bernese Mountain Dogs	1.0	2.0	Yes	No	Histiocytic Sarcoma	NA	Localized/Regional	NA	>5cm	NA	NA	NA	NA	NA	Positive	No
1200	No	NA	No	Yes	12.17	Male	Yes	33.57	Purebred	Golden Retriever	1.0	1.0	Yes	No	Hemangiosarcoma	NA	Localized/Regional	NA	<=5cm	NA	NA	NA	NA	NA	Positive	No
1201	No	NA	No	Yes	10.00	Male	Yes	29.06	Purebred	American Staffordshire Terrier	3.0	2.0	Yes	No	Soft Tissue Sarcoma	NA	Disseminated/Metastatic	NA	>5cm	NA	NA	NA	NA	NA	Positive	No
1202	No	NA	No	Yes	6.50	Male	Yes	44.19	Purebred	Golden Retriever	3.0	NA	Yes	No	Hemangiosarcoma	NA	Localized/Regional	NA	>5cm	NA	NA	NA	NA	NA	Positive	No

S1 Table. Full subject level data for subjects enrolled in the CANDiD study (continued)

See legend on last page.

Subject	Excluded	Reason for Exclusion	Training Set	Testing Set	Age	Sex	Spay/ Neuter	Weight (kg)	Purebred or Mixed Breed	Breed (Purebred)	Hemolysis	Lipemia	Cancer Diagnosis at Enrollment	Cancer Diagnosed after Liquid Biopsy	Cancer Type	Immunophenotype (Lymphoid Cancer)	Extent of Disease: Non-Lymphoid Cancer	Extent of Disease: Lymphoid Cancer	Tumor Size	Cancer Type (Cancer #2)	Immunophenotype (Cancer #2 if Lymphoid)	Extent of Disease: Non-Lymphoid Cancer (Cancer #2)	Extent of Disease: Lymphoid Cancer (Cancer #2)	Tumor Size (Cancer #2)	Liquid Biopsy Result	CSO Prediction of Hematological Malignancy
1203	No	NA	Yes	No	9.58	Male	Yes	27.56	Purebred	Golden Retriever	3.0	1.0	Yes	No	Mast Cell Tumor	NA	Localized/Regional	NA	<=5cm	NA	NA	NA	NA	NA	Negative	No
1204	No	NA	No	Yes	12.08	Female	Yes	28.86	Mixed-breed	NA	1.0	2.0	Yes	No	Soft Tissue Sarcoma	NA	Localized/Regional	NA	>5cm	NA	NA	NA	NA	NA	Negative	No
1205	No	NA	No	Yes	14.08	Male	Yes	9.92	Mixed-breed	NA	2.5	2.0	Yes	No	Anal Sac	NA	Localized/Regional	NA	<=5cm	NA	NA	NA	NA	NA	Negative	No
1206	No	NA	No	Yes	12.50	Female	Yes	24.55	Purebred	Labrador Retriever	4.0	2.0	Yes	No	Mast Cell Tumor	NA	Localized/Regional	NA	<=5cm	NA	NA	NA	NA	NA	Negative	No
1207	No	NA	No	Yes	8.50	Female	Yes	23.55	Mixed-breed	NA	1.3	1.5	Yes	No	Soft Tissue Sarcoma	NA	Localized/Regional	NA	<=5cm	NA	NA	NA	NA	NA	Negative	No
1208	No	NA	No	Yes	14.50	Female	Yes	14.43	Mixed-breed	NA	3.0	1.0	Yes	No	Liver	NA	Localized/Regional	NA	>5cm	NA	NA	NA	NA	NA	Positive	No
1209	No	NA	Yes	No	15.08	Male	Yes	30.16	Purebred	Labrador Retriever	1.5	1.0	Yes	No	Oral Cavity	NA	Undetermined	NA	>5cm	NA	NA	NA	NA	NA	Positive	No
1210	No	NA	No	Yes	10.08	Female	Yes	27.73	Purebred	Doberman Pinscher	1.0	2.0	Yes	No	Malignant Melanoma	NA	Localized/Regional	NA	<=5cm	NA	NA	NA	NA	NA	Positive	No
1211	Yes	Enrollment or Clinical Deviation	No	No	8.25	Female	Yes	13.33	Purebred	Australian Cattle Dog	0.7	2.0	Yes	No	NA	NA	NA	NA	NA	NA	NA	NA	NA	NA	Negative	No
1212	No	NA	No	Yes	10.08	Female	Yes	30.66	Mixed-breed	NA	2.0	2.0	Yes	No	Soft Tissue Sarcoma	NA	Localized/Regional	NA	>5cm	NA	NA	NA	NA	NA	Negative	No
1213	No	NA	No	Yes	11.58	Male	Yes	31.17	Purebred	Labrador Retriever	0.5	2.0	Yes	No	Soft Tissue Sarcoma	NA	Localized/Regional	NA	>5cm	NA	NA	NA	NA	NA	Positive	No
1214	No	NA	No	Yes	12.50	Female	Yes	33.37	Purebred	Labrador Retriever	1.0	2.0	Yes	No	Soft Tissue Sarcoma	NA	Disseminated/Metastatic	NA	>5cm	NA	NA	NA	NA	NA	Positive	No
1215	Yes	Enrollment or Clinical Deviation	No	No	14.17	Male	Yes	11.42	Mixed-breed	NA	3.0	2.0	Yes	No	NA	NA	NA	NA	NA	NA	NA	NA	NA	NA	Negative	No
1216	Yes	Laboratory Workflow Deviation	No	No	14.08	Male	Yes	6.81	Mixed-breed	NA	1.0	2.0	Yes	No	NA	NA	NA	NA	NA	NA	NA	NA	NA	NA	Positive	No
1217	No	NA	Yes	No	7.58	Male	Yes	38.58	Mixed-breed	NA	1.0	1.0	Yes	No	Bone, Osteosarcoma	NA	Localized/Regional	NA	<=5cm	NA	NA	NA	NA	NA	Positive	No
1218	No	NA	No	Yes	9.00	Female	Yes	29.06	Mixed-breed	NA	2.0	1.0	Yes	No	Chondrosarcoma	NA	Localized/Regional	NA	<=5cm	NA	NA	NA	NA	NA	Negative	No
1219	No	NA	No	Yes	6.92	Female	Yes	24.25	Mixed-breed	NA	4.0	2.0	Yes	No	Bone, Osteosarcoma	NA	Localized/Regional	NA	<=5cm	NA	NA	NA	NA	NA	Positive	No
1220	No	NA	No	Yes	10.33	Male	Yes	30.86	Mixed-breed	NA	3.5	2.0	Yes	No	Histiocytic Sarcoma	NA	Localized/Regional	NA	<=5cm	NA	NA	NA	NA	NA	Negative	No
1221	No	NA	Yes	No	13.42	Female	Yes	10.02	Mixed-breed	NA	4.0	NA	Yes	No	Nasal Cavity and Paranasal Sinuses	NA	Localized/Regional	NA	<=5cm	Oral Cavity	NA	Localized/Regional	NA	<=5cm	Negative	No
1222	No	NA	No	Yes	15.08	Male	Yes	12.43	Mixed-breed	NA	2.0	1.0	Yes	No	Mast Cell Tumor	NA	Disseminated/Metastatic	NA	<=5cm	NA	NA	NA	NA	NA	Positive	No
1223	No	NA	No	Yes	8.33	Female	Yes	37.48	Purebred	Labrador Retriever	1.5	1.0	Yes	No	Mast Cell Tumor	NA	Localized/Regional	NA	<=5cm	NA	NA	NA	NA	NA	Negative	No
1224	No	NA	Yes	No	8.42	Male	No	19.04	Mixed-breed	NA	3.0	2.0	Yes	No	Soft Tissue Sarcoma	NA	Localized/Regional	NA	>5cm	NA	NA	NA	NA	NA	Negative	No
1225	No	NA	No	Yes	12.75	Male	No	35.37	Purebred	Labrador Retriever	4.0	2.0	Yes	No	Nasal Planum	NA	Localized/Regional	NA	<=5cm	NA	NA	NA	NA	NA	Negative	No
1226	No	NA	Yes	No	11.00	Male	Yes	24.05	Purebred	Greyhound	4.0	2.0	Yes	No	Bone, Osteosarcoma	NA	Localized/Regional	NA	>5cm	NA	NA	NA	NA	NA	Positive	No
1227	Yes	Laboratory Workflow Deviation	No	No	12.92	Male	Yes	7.82	Purebred	Cairn Terrier	2.0	0.0	Yes	No	NA	NA	NA	NA	NA	NA	NA	NA	NA	NA	Negative	No
1228	No	NA	No	Yes	9.42	Male	Yes	12.63	Mixed-breed	NA	3.0	2.0	Yes	No	Mast Cell Tumor	NA	Localized/Regional	NA	<=5cm	NA	NA	NA	NA	NA	Negative	No
1229	Yes	Laboratory Workflow Deviation	No	No	9.83	Male	Yes	5.61	Mixed-breed	NA	4.0	2.0	Yes	No	NA	NA	NA	NA	NA	NA	NA	NA	NA	NA	Positive	No
1230	No	NA	Yes	No	10.00	Female	Yes	24.45	Mixed-breed	NA	2.0	1.0	Yes	No	Malignant Melanoma	NA	Localized/Regional	NA	<=5cm	NA	NA	NA	NA	NA	Negative	No
1231	No	NA	No	Yes	8.33	Female	Yes	33.87	Mixed-breed	NA	0.5	1.5	Yes	No	Mast Cell Tumor	NA	Localized/Regional	NA	<=5cm	NA	NA	NA	NA	NA	Negative	No
1232	No	NA	No	Yes	8.67	Male	Yes	23.75	Mixed-breed	NA	3.0	2.0	Yes	No	Anal Sac	NA	Localized/Regional	NA	<=5cm	NA	NA	NA	NA	NA	Negative	No
1233	No	NA	No	Yes	8.25	Male	Yes	28.06	Mixed-breed	NA	2.0	1.0	Yes	No	Bone, Osteosarcoma	NA	Localized/Regional	NA	UNK	NA	NA	NA	NA	NA	Positive	No
1234	Yes	Laboratory Workflow Deviation	No	No	10.00	Female	Yes	9.52	Purebred	Pembroke Welsh Corgi	2.0	2.0	Yes	No	NA	NA	NA	NA	NA	NA	NA	NA	NA	NA	Positive	No
1235	No	NA	No	Yes	12.25	Female	Yes	32.07	Mixed-breed	NA	0.0	1.0	Yes	No	Mast Cell Tumor	NA	Localized/Regional	NA	>5cm	NA	NA	NA	NA	NA	Positive	No
1236	Yes	Enrollment or Clinical Deviation	No	No	10.83	Male	Yes	31.57	Purebred	Golden Retriever	3.0	2.0	Yes	No	NA	NA	NA	NA	NA	NA	NA	NA	NA	NA	Positive	No
1237	No	NA	No	Yes	7.83	Female	Yes	28.56	Mixed-breed	NA	3.0	2.0	Yes	No	Soft Tissue Sarcoma	NA	Localized/Regional	NA	>5cm	NA	NA	NA	NA	NA	Positive	No
1238	No	NA	No	Yes	7.67	Male	No	58.12	Purebred	Greater Swiss Mountain Dog	3.0	NA	Yes	No	Mast Cell Tumor	NA	Localized/Regional	NA	<=5cm	NA	NA	NA	NA	NA	Negative	No
1239	No	NA	No	Yes	8.17	Female	Yes	22.65	Mixed-breed	NA	2.0	1.0	Yes	No	Oral Cavity	NA	Localized/Regional	NA	<=5cm	NA	NA	NA	NA	NA	Negative	No
1240	Yes	Laboratory Workflow Deviation	No	No	12.08	Male	Yes	14.53	Purebred	Russell Terrier	2.0	NA	Yes	No	NA	NA	NA	NA	NA	NA	NA	NA	NA	NA	Negative	No
1241	Yes	Laboratory Workflow Deviation	No	No	11.08	Female	No	5.11	Purebred	Shih Tzu	2.0	2.0	Yes	No	NA	NA	NA	NA	NA	NA	NA	NA	NA	NA	Positive	No
1242	No	NA	No	Yes	9.08	Female	Yes	22.35	Mixed-breed	NA	1.5	2.0	Yes	No	Bone, Osteosarcoma	NA	Localized/Regional	NA	>5cm	NA	NA	NA	NA	NA	Negative	No
1243	No	NA	No	Yes	8.92	Female	Yes	28.46	Purebred	Labrador Retriever	3.0	2.0	Yes	No	Mast Cell Tumor	NA	Localized/Regional	NA	<=5cm	NA	NA	NA	NA	NA	Negative	No
1244	No	NA	No	Yes	6.42	Male	Yes	47.60	Purebred	Golden Retriever	2.0	2.0	Yes	No	Oral Cavity	NA	Localized/Regional	NA	>5cm	NA	NA	NA	NA	NA	Negative	No
1245	No	NA	No	Yes	11.42	Female	Yes	18.44	Mixed-breed	NA	3.0	1.0	Yes	No	Thyroid	NA	Localized/Regional	NA	>5cm	NA	NA	NA	NA	NA	Negative	No
1246	No	NA	No	Yes	9.08	Male	Yes	60.13	Purebred	Bullmastiff	2.5	1.0	Yes	No	Bone, Osteosarcoma	NA	Localized/Regional	NA	>5cm	NA	NA	NA	NA	NA	Positive	No
1247	No	NA	No	Yes	12.25	Male	Yes	34.57	Mixed-breed	NA	3.0	NA	Yes	No	Anal Sac	NA	Localized/Regional	NA	<=5cm	NA	NA	NA	NA	NA	Negative	No
1248	Yes	Laboratory Workflow Deviation	No	No	9.75	Female	Yes	26.66	Purebred	Boxer	4.0	2.0	Yes	No	Thyroid	NA	Localized/Regional	NA	<=5cm	Mast Cell Tumor	NA	Localized/Regional	NA	UNK	Negative	No
1249	Yes	Laboratory Workflow Deviation	No	No	15.08	Male	Yes	10.12	Purebred	Bichons Frise	4.0	2.0	Yes	No	NA	NA	NA	NA	NA	NA	NA	NA	NA	NA	Negative	No
1250	No	NA	No	Yes	11.42	Female	Yes	42.19	Purebred	Rottweiler	0.0	1.0	Yes	No	Malignant Melanoma	NA	Localized/Regional	NA	<=5cm	NA	NA	NA	NA	NA	Negative	No
1251	No	NA	No	Yes	4.08	Male	Yes	36.88	Purebred	Golden Retriever	1.5	2.0	Yes	No	Mast Cell Tumor	NA	Localized/Regional	NA	<=5cm	NA	NA	NA	NA	NA	Negative	No
1252	No	NA	Yes	No	13.67	Male	Yes	29.06	Mixed-breed	NA	3.0	2.0	Yes	No	Oral Cavity	NA	Localized/Regional	NA	<=5cm	NA	NA	NA	NA	NA	Negative	No
1253	No	NA	No	Yes	10.08	Male	Yes	31.67	Mixed-breed	NA	2.0	0.0	Yes	No	Bone, Osteosarcoma	NA	Localized/Regional	NA	>5cm	NA	NA	NA	NA	NA	Positive	No
1254	No	NA	No	Yes	10.42	Female	Yes	31.06	Mixed-breed	NA	2.0	2.0	Yes	No	Lymphoma, Intermediate to Large Cell	NA	NA	Disseminated/ Metastatic	<=5cm	NA	NA	NA	NA	NA	Negative	No
1255	No	NA	No	Yes	9.00	Female	Yes	19.04	Purebred	English Bulldog	1.0	1.0	Yes	No	Bone, Osteosarcoma	NA	Localized/Regional	NA	<=5cm	NA	NA	NA	NA	NA	Negative	No
1256	No	NA	No	Yes	13.00	Female	Yes	21.85	Mixed-breed	NA	3.0	2.0	Yes	No	Mast Cell Tumor	NA	Localized/Regional	NA	>5cm	NA	NA	NA	NA	NA	Negative	No
1257	No	NA	Yes	No	10.67	Female	Yes	32.07	Mixed-breed	NA	1.5	0.0	Yes	No	Mast Cell Tumor	NA	Localized/Regional	NA	<=5cm	NA	NA	NA	NA	NA	Negative	No
1258	No	NA	Yes	No	11.00	Male	Yes	33.07	Mixed-breed	NA	1.0	2.0	Yes	No	Nasal Cavity and Paranasal Sinuses	NA	Localized/Regional	NA	UNK	NA	NA	NA	NA	NA	Negative	No
1259	No	NA	No	Yes	5.42	Male	Yes	12.43	Purebred	French Bulldog	1.0	0.0	Yes	No	Mast Cell Tumor	NA	Localized/Regional	NA	<=5cm	NA	NA	NA	NA	NA	Negative	No
1260	Yes	Enrollment or Clinical Deviation	No	No	4.58	Male	No	64.84	Purebred	Great Dane	2.0	2.0	Yes	No	NA	NA	NA	NA	NA	NA	NA	NA	NA	NA	Negative	No
1261	Yes	Laboratory Workflow Deviation	No	No	13.42	Female	Yes	4.51	Mixed-breed	NA	2.0	NA	Yes	No	NA	NA	NA	NA	NA	NA	NA	NA	NA	NA	Negative	No
1262	No	NA	Yes	No	11.67	Female	Yes	30.26	Purebred	Golden Retriever	4.0	2.0	Yes	No	Mast Cell Tumor	NA	Localized/Regional	NA	<=5cm	NA	NA	NA	NA	NA	Negative	No
1263	No	NA	No	Yes	10.92	Male	Yes	34.37	Purebred	Weimaraner	2.0	0.0	Yes	No	Thyroid	NA	Localized/Regional	NA	>5cm	NA	NA	NA	NA	NA	Negative	No
1264	No	NA	No	Yes	9.00	Male	Yes	38.78	Mixed-breed	NA	1.0	NA	Yes	No	Hemangiosarcoma	NA	Disseminated/Metastatic	NA	<=5cm	Stomach	NA	Undetermined	NA	<=5cm	Positive	Yes
1265	No	NA	No	Yes	9.92	Male	Yes	25.05	Mixed-breed	NA	2.5	2.0	Yes	No	Hemangiosarcoma	NA	Disseminated/Metastatic	NA	<=5cm	Soft Tissue Sarcoma	NA	Localized/Regional	NA	<=5cm	Positive	No
1266	Yes	Enrollment or Clinical Deviation	No	No	10.92	Female	Yes	34.77	Mixed-breed	NA	1.0	0.0	Yes	No	NA	NA	NA	NA	NA	NA	NA	NA	NA	NA	Negative	No

S1 Table. Full subject level data for subjects enrolled in the CANDiD study (continued)

See legend on last page.

Subject	Excluded	Reason for Exclusion	Training Set	Testing Set	Age	Sex	Spay/ Neuter	Weight (kg)	Purebred or Mixed Breed	Breed (Purebred)	Hemolysis	Lipemia	Cancer Diagnosis at Enrollment	Cancer Diagnosed after Liquid Biopsy	Cancer Type	Immunophenotype (Lymphoid Cancer)	Extent of Disease: Non-Lymphoid Cancer	Extent of Disease: Lymphoid Cancer	Tumor Size	Cancer Type (Cancer #2)	Immunophenotype (Cancer #2 if Lymphoid)	Extent of Disease: Non-Lymphoid Cancer (Cancer #2)	Extent of Disease: Lymphoid Cancer (Cancer #2)	Tumor Size (Cancer #2)	Liquid Biopsy Result	CSO Prediction of Hematological Malignancy
1267	No	NA	No	Yes	10.42	Female	Yes	27.26	Mixed-breed	NA	3.0	NA	Yes	No	Anal Sac	NA	Localized/Regional	NA	<=5cm	NA	NA	NA	NA	NA	Negative	No
1268	Yes	Enrollment or Clinical Deviation	No	No	10.25	Female	Yes	27.46	Purebred	American Staffordshire Terrier	3.0	2.0	Yes	No	NA	NA	NA	NA	NA	NA	NA	NA	NA	NA	Negative	No
1269	No	NA	No	Yes	11.17	Male	No	17.04	Purebred	Beagle	3.5	2.0	Yes	No	Mast Cell Tumor	NA	Localized/Regional	NA	<=5cm	NA	NA	NA	NA	NA	Negative	No
1270	No	NA	No	Yes	9.75	Female	Yes	23.15	Mixed-breed	NA	4.0	NA	Yes	No	Mammary Gland Carcinoma	NA	Localized/Regional	NA	<=5cm	NA	NA	NA	NA	NA	Negative	No
1271	Yes	Enrollment or Clinical Deviation	No	No	0.92	Male	Yes	24.55	Purebred	Belgian Malinois	3.5	NA	Yes	No	Mast Cell Tumor	NA	Disseminated/Metastatic	NA	<=5cm	NA	NA	NA	NA	NA	Negative	No
1272	Yes	Test failure	No	No	8.00	Female	Yes	26.05	Mixed-breed	NA	4.0	2.0	Yes	No	NA	NA	NA	NA	NA	NA	NA	NA	NA	NA	Indetermi-nate	No
1273	Yes	Enrollment or Clinical Deviation	No	No	5.00	Female	Yes	29.06	Mixed-breed	NA	4.0	2.0	Yes	No	NA	NA	NA	NA	NA	NA	NA	NA	NA	NA	Negative	No
1274	Yes	Test failure	No	No	7.00	Male	Yes	24.05	Mixed-breed	NA	2.0	1.0	Yes	No	Soft Tissue Sarcoma	NA	Localized/Regional	NA	<=5cm	NA	NA	NA	NA	NA	Negative	No
1275	No	NA	Yes	No	11.00	Female	Yes	7.11	Purebred	Shih Tzu	4.0	2.0	Yes	No	Mammary Gland Carcinoma	NA	Localized/Regional	NA	<=5cm	NA	NA	NA	NA	NA	Positive	No
1276	No	NA	No	Yes	10.58	Male	Yes	26.56	Purebred	Border Collie	4.0	2.0	Yes	No	Anal Sac	NA	Localized/Regional	NA	<=5cm	NA	NA	NA	NA	NA	Negative	No
1277	Yes	Laboratory Workflow Deviation	No	No	7.00	Male	Yes	26.35	Mixed-breed	NA	3.0	1.0	Yes	No	NA	NA	NA	NA	NA	NA	NA	NA	NA	NA	Negative	No
1278	No	NA	No	Yes	7.67	Male	Yes	38.28	Purebred	Doberman Pinscher	1.5	NA	Yes	No	Bone, Osteosarcoma	NA	Localized/Regional	NA	UNK	NA	NA	NA	NA	NA	Positive	No
1279	No	NA	Yes	No	7.00	Male	No	27.46	Mixed-breed	NA	3.0	1.0	Yes	No	Oral Cavity	NA	Localized/Regional	NA	<=5cm	NA	NA	NA	NA	NA	Negative	No
1280	Yes	Laboratory Workflow Deviation	No	No	13.92	Male	Yes	7.22	Mixed-breed	NA	4.0	2.0	Yes	No	NA	NA	NA	NA	NA	NA	NA	NA	NA	NA	Negative	No
1281	No	NA	Yes	No	10.33	Female	Yes	29.56	Purebred	Golden Retriever	3.0	1.0	Yes	No	Bone, Osteosarcoma	NA	Localized/Regional	NA	<=5cm	NA	NA	NA	NA	NA	Positive	No
1282	No	NA	No	Yes	9.58	Male	Yes	30.16	Purebred	Siberian Husky	2.0	NA	Yes	No	Liver	NA	Localized/Regional	NA	>5cm	NA	NA	NA	NA	NA	Negative	No
1283	No	NA	No	Yes	7.83	Female	Yes	22.35	Mixed-breed	NA	2.5	1.0	Yes	No	Soft Tissue Sarcoma	NA	Disseminated/Metastatic	NA	>5cm	NA	NA	NA	NA	NA	Positive	No
1284	Yes	Laboratory Workflow Deviation	No	No	2.00	Male	Yes	47.10	Purebred	Akbash	2.0	1.0	Yes	No	NA	NA	NA	NA	NA	NA	NA	NA	NA	NA	Indetermi-nate	No
1285	No	NA	No	Yes	11.67	Male	Yes	36.28	Mixed-breed	NA	1.0	0.0	Yes	No	Soft Tissue Sarcoma	NA	Localized/Regional	NA	>5cm	NA	NA	NA	NA	NA	Negative	No
1286	Yes	Enrollment or Clinical Deviation	No	No	11.17	Female	Yes	12.43	Mixed-breed	NA	4.0	2.0	Yes	No	NA	NA	NA	NA	NA	NA	NA	NA	NA	NA	Positive	No
1287	No	NA	No	Yes	11.42	Female	Yes	42.29	Mixed-breed	NA	3.0	2.0	Yes	No	Lung	NA	Localized/Regional	NA	<=5cm	NA	NA	NA	NA	NA	Positive	No
1288	Yes	Laboratory Workflow Deviation	No	No	8.33	Male	No	10.42	Purebred	Scottish Terrier	1.0	0.0	Yes	No	NA	NA	NA	NA	NA	NA	NA	NA	NA	NA	Positive	No
1289	No	NA	No	Yes	9.33	Female	Yes	22.35	Mixed-breed	NA	4.0	2.0	Yes	No	Histiocytic Sarcoma	NA	Localized/Regional	NA	<=5cm	NA	NA	NA	NA	NA	Positive	No
1290	No	NA	No	Yes	9.17	Male	Yes	39.58	Purebred	German Shepherd	2.0	1.5	Yes	No	Anal Sac	NA	Localized/Regional	NA	<=5cm	NA	NA	NA	NA	NA	Negative	No
1291	No	NA	No	Yes	10.00	Male	Yes	34.47	Purebred	Golden Retriever	2.5	1.0	Yes	No	Hemangiosarcoma	NA	Localized/Regional	NA	>5cm	NA	NA	NA	NA	NA	Positive	No
1292	No	NA	No	Yes	10.08	Male	Yes	9.52	Purebred	Rat Terrier	3.0	1.5	Yes	No	Salivary Gland	NA	Localized/Regional	NA	>5cm	NA	NA	NA	NA	NA	Positive	No
1293	No	NA	No	Yes	11.08	Male	Yes	22.85	Mixed-breed	NA	1.0	0.0	Yes	No	Pancreas, Endocrine	NA	Localized/Regional	NA	<=5cm	NA	NA	NA	NA	NA	Negative	No
1294	No	NA	No	Yes	10.17	Male	Yes	43.39	Mixed-breed	NA	3.0	NA	Yes	No	Anal Sac	NA	Localized/Regional	NA	<=5cm	NA	NA	NA	NA	NA	Positive	No
1295	Yes	Test failure	No	No	9.00	Male	Yes	15.83	Mixed-breed	NA	4.0	2.0	Yes	No	Anal Sac	NA	Localized/Regional	NA	>5cm	NA	NA	NA	NA	NA	Positive	No
1296	Yes	Laboratory Workflow Deviation	No	No	6.08	Female	Yes	41.09	Mixed-breed	NA	4.0	2.0	Yes	No	NA	NA	NA	NA	NA	NA	NA	NA	NA	NA	Negative	No
1297	No	NA	No	Yes	10.00	Female	Yes	36.58	Mixed-breed	NA	2.0	2.0	Yes	No	Bone, Osteosarcoma	NA	Disseminated/Metastatic	NA	<=5cm	NA	NA	NA	NA	NA	Positive	No
1298	No	NA	No	Yes	9.33	Female	Yes	36.88	Purebred	Rhodesian Ridgeback	4.0	2.0	Yes	No	Soft Tissue Sarcoma	NA	Localized/Regional	NA	>5cm	NA	NA	NA	NA	NA	Negative	No
1299	No	NA	No	Yes	10.67	Male	Yes	28.06	Mixed-breed	NA	2.0	NA	Yes	No	Soft Tissue Sarcoma	NA	Localized/Regional	NA	<=5cm	NA	NA	NA	NA	NA	Negative	No
1300	Yes	Test failure	No	No	1.92	Male	No	32.47	Purebred	Rottweiler	3.0	0.0	Yes	No	Bone, Osteosarcoma	NA	Localized/Regional	NA	UNK	NA	NA	NA	NA	NA	Positive	No
1301	No	NA	No	Yes	9.67	Male	No	38.58	Purebred	German Shepherd	3.0	0.0	Yes	No	Thyroid	NA	Localized/Regional	NA	<=5cm	NA	NA	NA	NA	NA	Negative	No
1302	No	NA	No	Yes	4.92	Male	No	61.13	Purebred	Bullmastiff	3.0	2.0	Yes	No	Bone, Osteosarcoma	NA	Localized/Regional	NA	<=5cm	NA	NA	NA	NA	NA	Negative	No
1303	No	NA	No	Yes	5.58	Male	Yes	23.05	Purebred	Vizslas	2.0	1.0	Yes	No	Mast Cell Tumor	NA	Localized/Regional	NA	>5cm	NA	NA	NA	NA	NA	Negative	No
1304	No	NA	No	Yes	11.25	Male	Yes	32.07	Purebred	Labrador Retriever	4.0	2.0	Yes	No	Mast Cell Tumor	NA	Localized/Regional	NA	<=5cm	NA	NA	NA	NA	NA	Negative	No
1305	No	NA	No	Yes	10.83	Male	Yes	37.58	Mixed-breed	NA	1.0	0.0	Yes	No	Bone, Osteosarcoma	NA	Localized/Regional	NA	<=5cm	NA	NA	NA	NA	NA	Positive	No
1307	No	NA	No	Yes	11.83	Male	Yes	28.96	Purebred	German Shorthaired Pointer	3.0	1.0	Yes	No	Oral Cavity	NA	Localized/Regional	NA	>5cm	NA	NA	NA	NA	NA	Negative	No
1308	Yes	Test failure	No	No	11.83	Female	Yes	20.74	Mixed-breed	NA	4.0	2.0	Yes	No	Soft Tissue Sarcoma	NA	Localized/Regional	NA	<=5cm	NA	NA	NA	NA	NA	Negative	No
1309	No	NA	No	Yes	9.33	Female	Yes	30.66	Purebred	Siberian Husky	2.0	0.0	Yes	No	Mammary Gland Carcinoma	NA	Localized/Regional	NA	<=5cm	NA	NA	NA	NA	NA	Negative	No
1310	No	NA	No	Yes	6.50	Female	No	36.08	Purebred	Boxer	NA	1.0	Yes	No	Ear Canal	NA	Disseminated/Metastatic	NA	<=5cm	NA	NA	NA	NA	NA	Positive	No
1311	Yes	Laboratory Workflow Deviation	No	No	11.83	Female	Yes	6.01	Mixed-breed	NA	2.0	2.0	Yes	No	NA	NA	NA	NA	NA	NA	NA	NA	NA	NA	Positive	No
1312	No	NA	No	Yes	2.33	Female	No	22.35	Purebred	Boxer	4.0	2.0	Yes	No	Lymphoma, Indolent	NA	NA	Disseminated/ Metastatic	<=5cm	NA	NA	NA	NA	NA	Negative	No
1313	No	NA	Yes	No	3.00	Female	Yes	28.56	Purebred	Golden Retriever	3.5	1.5	Yes	No	Soft Tissue Sarcoma	NA	Localized/Regional	NA	<=5cm	NA	NA	NA	NA	NA	Negative	No
1314	No	NA	No	Yes	10.42	Female	Yes	24.25	Mixed-breed	NA	1.0	1.0	Yes	No	Hemangiosarcoma	NA	Disseminated/Metastatic	NA	>5cm	NA	NA	NA	NA	NA	Positive	No
1315	Yes	Enrollment or Clinical Deviation	No	No	10.92	Male	Yes	21.04	Purebred	Beagle	3.0	1.0	Yes	No	NA	NA	NA	NA	NA	NA	NA	NA	NA	NA	Negative	No
1316	No	NA	Yes	No	8.00	Female	Yes	28.16	Purebred	Siberian Husky	2.0	NA	Yes	No	Soft Tissue Sarcoma	NA	Localized/Regional	NA	>5cm	NA	NA	NA	NA	NA	Negative	No
1317	Yes	Laboratory Workflow Deviation	No	No	11.25	Female	Yes	43.19	Purebred	Labrador Retriever	3.0	2.0	Yes	No	NA	NA	NA	NA	NA	NA	NA	NA	NA	NA	Negative	No
1318	Yes	Laboratory Workflow Deviation	No	No	7.08	Male	Yes	29.36	Purebred	English Bulldog	3.5	2.0	Yes	No	Skin	NA	Localized/Regional	NA	<=5cm	NA	NA	NA	NA	NA	Negative	No
1319	No	NA	No	Yes	12.00	Female	Yes	19.44	Purebred	Soft Coated Wheaten Terrier	4.0	2.0	Yes	No	Urinary Bladder/Urethra	NA	Localized/Regional	NA	>5cm	NA	NA	NA	NA	NA	Negative	No
1320	No	NA	No	Yes	11.00	Male	Yes	39.58	Mixed-breed	NA	3.0	2.0	Yes	No	Anal Sac	NA	Localized/Regional	NA	<=5cm	Bone, Osteosarcoma	NA	Localized/Regional	NA	UNK	Negative	No
1321	No	NA	No	Yes	11.83	Male	Yes	20.04	Mixed-breed	NA	2.0	0.0	Yes	No	Thyroid	NA	Localized/Regional	NA	>5cm	NA	NA	NA	NA	NA	Negative	No
1322	No	NA	No	Yes	10.08	Male	Yes	47.90	Purebred	German Shepherd	2.0	1.0	Yes	No	Bone, Osteosarcoma	NA	Localized/Regional	NA	UNK	NA	NA	NA	NA	NA	Positive	No
1323	No	NA	No	Yes	11.92	Male	Yes	32.57	Mixed-breed	NA	3.0	2.0	Yes	No	Lung	NA	Localized/Regional	NA	<=5cm	NA	NA	NA	NA	NA	Negative	No
1324	Yes	Laboratory Workflow Deviation	No	No	12.08	Male	Yes	8.82	Purebred	Boston Terrier	3.0	2.0	Yes	No	NA	NA	NA	NA	NA	NA	NA	NA	NA	NA	Negative	No
1325	No	NA	No	Yes	10.17	Male	Yes	26.96	Purebred	Labrador Retriever	2.5	2.0	Yes	No	Peripheral Nerve Sheath Tumor	NA	Localized/Regional	NA	<=5cm	NA	NA	NA	NA	NA	Negative	No
1326	Yes	Laboratory Workflow Deviation	No	No	9.75	Male	Yes	7.11	Purebred	Dachshund	1.0	0.0	Yes	No	NA	NA	NA	NA	NA	NA	NA	NA	NA	NA	Positive	No
1327	No	NA	No	Yes	8.25	Female	Yes	37.58	Mixed-breed	NA	2.5	2.0	Yes	No	Soft Tissue Sarcoma	NA	Localized/Regional	NA	<=5cm	NA	NA	NA	NA	NA	Negative	No

S1 Table. Full subject level data for subjects enrolled in the CANDiD study

Subject	Excluded	Reason for Exclusion	Training Set	Testing Set	Age	Sex	Spay/ Neuter	Weight (kg)	Purebred or Mixed Breed	Breed (Purebred)	Hemolysis	Lipemia	Cancer Diagnosis at Enrollment	Cancer Diagnosed after Liquid Biopsy	Cancer Type	Immunophenotype (Lymphoid Cancer)	Extent of Disease: Non-Lymphoid Cancer	Extent of Disease: Lymphoid Cancer	Tumor Size	Cancer Type (Cancer #2)	Immunophenotype (Cancer #2 if Lymphoid)	Extent of Disease: Non-Lymphoid Cancer (Cancer #2)	Extent of Disease: Lymphoid Cancer (Cancer #2)	Tumor Size (Cancer #2)	Liquid Biopsy Result	CSO Prediction of Hematological Malignancy
1328	No	NA	No	Yes	1.92	Male	Yes	37.78	Purebred	Golden Retriever	1.0	1.0	Yes	No	Bone, Osteosarcoma	NA	Localized/Regional	NA	>5cm	NA	NA	NA	NA	NA	Positive	No
1329	No	NA	No	Yes	13.75	Female	Yes	22.35	Purebred	Siberian Husky	2.0	2.0	Yes	No	Adrenal Gland	NA	Localized/Regional	NA	<=5cm	NA	NA	NA	NA	NA	Negative	No
1330	No	NA	No	Yes	6.00	Male	Yes	53.81	Purebred	Golden Retriever	1.0	1.0	Yes	No	Kidney	NA	Localized/Regional	NA	>5cm	NA	NA	NA	NA	NA	Positive	No
1331	No	NA	No	Yes	10.00	Male	Yes	32.27	Mixed-breed	NA	2.5	1.0	Yes	No	Thyroid	NA	Localized/Regional	NA	<=5cm	NA	NA	NA	NA	NA	Negative	No
1332	No	NA	No	Yes	10.67	Male	Yes	12.03	Mixed-breed	NA	2.5	1.0	Yes	No	Liver	NA	Localized/Regional	NA	>5cm	NA	NA	NA	NA	NA	Positive	No
1333	Yes	Laboratory Workflow Deviation	No	No	5.08	Male	Yes	33.07	Mixed-breed	NA	4.0	2.0	Yes	No	Thyroid	NA	Localized/Regional	NA	>5cm	NA	NA	NA	NA	NA	Positive	No
1334	No	NA	No	Yes	7.25	Male	Yes	28.06	Purebred	Siberian Husky	2.5	NA	Yes	No	Lung	NA	Localized/Regional	NA	>5cm	NA	NA	NA	NA	NA	Positive	No
1335	Yes	Enrollment or Clinical Deviation	No	No	8.17	Female	Yes	24.25	Purebred	Siberian Husky	2.0	2.0	Yes	No	NA	NA	NA	NA	NA	NA	NA	NA	NA	NA	Negative	No
1336	No	NA	No	Yes	14.50	Male	Yes	42.69	Mixed-breed	NA	4.0	1.0	Yes	No	Soft Tissue Sarcoma	NA	Localized/Regional	NA	>5cm	NA	NA	NA	NA	NA	Negative	No
1337	No	NA	No	Yes	13.92	Male	Yes	14.73	Purebred	Pembroke Welsh Corgi	2.0	1.0	Yes	No	Hemangiosarcoma	NA	Localized/Regional	NA	>5cm	NA	NA	NA	NA	NA	Positive	No
1338	Yes	Laboratory Workflow Deviation	No	No	2.00	Female	Yes	9.32	Purebred	Boston Terrier	4.0	2.0	Yes	No	NA	NA	NA	NA	NA	NA	NA	NA	NA	NA	Negative	No
1339	No	NA	No	Yes	12.33	Female	No	26.56	Mixed-breed	NA	2.0	2.0	Yes	No	Mammary Gland Carcinoma	NA	Localized/Regional	NA	<=5cm	NA	NA	NA	NA	NA	Negative	No
1340	Yes	Laboratory Workflow Deviation	No	No	13.67	Male	Yes	37.78	Mixed-breed	NA	2.0	2.0	Yes	No	NA	NA	NA	NA	NA	NA	NA	NA	NA	NA	Positive	No
1341	Yes	Laboratory Workflow Deviation	No	No	15.92	Female	Yes	16.84	Mixed-breed	NA	1.0	1.0	Yes	No	NA	NA	NA	NA	NA	NA	NA	NA	NA	NA	Positive	No
1342	Yes	Laboratory Workflow Deviation	No	No	11.00	Female	Yes	10.92	Purebred	American Eskimo Dog	4.0	2.0	Yes	No	NA	NA	NA	NA	NA	NA	NA	NA	NA	NA	Negative	No
1343	Yes	Enrollment or Clinical Deviation	No	No	7.67	Male	Yes	29.06	Mixed-breed	NA	1.5	0.5	Yes	No	NA	NA	NA	NA	NA	NA	NA	NA	NA	NA	Positive	No
1344	No	NA	No	Yes	10.92	Female	No	15.13	Purebred	Beagle	2.0	1.0	Yes	No	Mammary Gland Carcinoma	NA	Localized/Regional	NA	>5cm	Ovary	NA	Localized/Regional	NA	<=5cm	Negative	No
1345	No	NA	Yes	No	11.17	Male	Yes	30.06	Purebred	German Shorthaired Pointer	0.0	0.0	Yes	No	Adrenal Gland	NA	Localized/Regional	NA	>5cm	NA	NA	NA	NA	NA	Negative	No
1346	Yes	Laboratory Workflow Deviation	No	No	8.00	Male	Yes	30.16	Mixed-breed	NA	0.0	0.0	Yes	No	NA	NA	NA	NA	NA	NA	NA	NA	NA	NA	Negative	No
1347	Yes	Laboratory Workflow Deviation	No	No	9.92	Female	Yes	38.28	Mixed-breed	NA	4.0	2.0	Yes	No	NA	NA	NA	NA	NA	NA	NA	NA	NA	NA	Negative	No
1348	Yes	Laboratory Workflow Deviation	No	No	14.42	Female	Yes	26.56	Purebred	Golden Retriever	3.0	2.0	Yes	No	NA	NA	NA	NA	NA	NA	NA	NA	NA	NA	Positive	No
1349	Yes	Laboratory Workflow Deviation	No	No	7.08	Female	Yes	46.80	Mixed-breed	NA	2.0	2.0	Yes	No	NA	NA	NA	NA	NA	NA	NA	NA	NA	NA	Positive	No
1350	Yes	Laboratory Workflow Deviation	No	No	7.75	Male	Yes	45.70	Purebred	Cane Corso	2.0	2.0	Yes	No	NA	NA	NA	NA	NA	NA	NA	NA	NA	NA	Positive	No
1351	Yes	Laboratory Workflow Deviation	No	No	NA	NA	NA	NA	NA	NA	2.0	1.0	Yes	No	NA	NA	NA	NA	NA	NA	NA	NA	NA	NA	Positive	No
1352	Yes	Laboratory Workflow Deviation	No	No	NA	NA	NA	NA	NA	NA	2.0	2.0	Yes	No	NA	NA	NA	NA	NA	NA	NA	NA	NA	NA	Negative	No
1353	Yes	Enrollment or Clinical Deviation	No	No	13.08	Female	Yes	18.84	Purebred	Brittany	2.0	1.5	Yes	No	NA	NA	NA	NA	NA	NA	NA	NA	NA	NA	Negative	No
1354	Yes	Laboratory Workflow Deviation	No	No	13.00	Female	Yes	19.54	Mixed-breed	NA	0.0	0.0	Yes	No	NA	NA	NA	NA	NA	NA	NA	NA	NA	NA	Positive	No
1355	Yes	Laboratory Workflow Deviation	No	No	10.00	Female	Yes	39.78	Mixed-breed	NA	3.0	2.0	Yes	No	NA	NA	NA	NA	NA	NA	NA	NA	NA	NA	Positive	No
1356	Yes	Laboratory Workflow Deviation	No	No	14.00	Female	Yes	19.44	Purebred	Australian Shepherd	3.0	2.0	Yes	No	NA	NA	NA	NA	NA	NA	NA	NA	NA	NA	Negative	No
1357	Yes	Laboratory Workflow Deviation	No	No	NA	NA	NA	NA	NA	NA	3.5	2.0	Yes	No	NA	NA	NA	NA	NA	NA	NA	NA	NA	NA	Negative	No
1358	Yes	Laboratory Workflow Deviation	No	No	NA	NA	NA	NA	NA	NA	1.0	1.0	Yes	No	NA	NA	NA	NA	NA	NA	NA	NA	NA	NA	Positive	No

LEGEND

**Subject**	Subject number (deidentified)
**Excluded**	Whether or not subject was excluded from the training and testing sets
**Reason for Exclusion**	Reason subject was excluded from the training and testing sets
**Training Set**	Whether or not subject was included in the training set
**Testing Set**	Whether or not subject was included in the testing set
**Age**	Subject age (years)
**Sex**	Subject sex
**Spay/Neuter**	Whether or not subject was spayed/neutered
**Weight (kg)**	Subject weight (kg)
**Purebred or Mixed-Breed**	Whether subject was purebred or mixed-breed
**Breed (Purebred)**	Subject breed (for purebred subjects)
**Hemolysis**	Extent of hemolysis of sample received at the central lab, based on visual inspection of color: low (<1), medium (1-2.5), high (>2.5); not available
**Lipemia**	Extent of lipemia of sample received at the central lab, based on visual inspection of extent of turbidity: low (0), medium (>0-1), high (>1), not available
**Cancer Diagnosis at Enrollment**	Whether or not subject was enrolled into the CANDiD study as "cancer-diagnosed"
**Cancer Diagnosed after Liquid Biopsy**	Whether subject was enrolled into the CANDiD study as "presumably cancer-free" but received a cancer diagnosis following a positive liquid biopsy result and subsequent workup
**Cancer Type**	For subjects enrolled as "cancer-diagnosed", type of cancer diagnosed
**Immunophenotype (Lymphoid Cancer)**	Immunophenotype for subjects diagnosed with lymphoid cancer
**Extent of Disease: Non-Lymphoid Cancer**	For subjects diagnosed with non-lymphoid cancer, extent of disease (Localized/Regional, Disseminated/Metastatic, Undetermined)
**Extent of Disease: Lymphoid Cancer**	For subjects diagnosed with lymphoid cancer, extent of disease (Localized/Regional, Disseminated/Metastatic, Undetermined)
**Tumor Size**	For cancer-diagnosed subjects, the longest diameter (cm) of the largest lesion
**Cancer Type (Cancer #2)**	For subjects with multiple concurrent primary cancers, the second cancer type
**Immunophenotype (Cancer #2 if Lymphoid)**	Immunophenotype for subjects with multiple concurrent primary cancers in which the second diagnosis was lymphoid cancer
**Extent of Disease: Non-Lymphoid Cancer (Cancer #2)**	For subjects with multiple concurrent primary cancers in which the second diagnosis was non-lymphoid cancer, extent of disease of the second cancer type (Localized/Regional, Disseminated/Metastatic, Undetermined)
**Extent of Disease: Lymphoid Cancer (Cancer #2)**	For subjects with multiple concurrent primary cancers in which the second diagnosis was lymphoid cancer, extent of disease of the second cancer type (Localized/Regional, Disseminated/Metastatic, Undetermined)
**Tumor Size (Cancer #2)**	For subjects with multiple concurrent primary cancers, the longest diameter (cm) of the largest lesion from the second cancer type
**Liquid Biopsy Result**	Result of liquid biopsy testing (Positive, Negative, Indeterminate, Fail)
**CSO Prediction Of Hematological Malignancy**	Whether the subject received a Cancer Signal Origin (CSO) prediction of hematological malignancy
**NA**	Not applicable
