# Supplementary material for: Clinical validation of a next-generation sequencing-based multi-cancer early detection “liquid biopsy” blood test in over 1,000 dogs using an independent testing set: The CANcer Detection in Dogs (CANDiD) study
Source: PLoS One. 2022 Apr 26;17(4):e0266623. doi: 10.1371/journal.pone.0266623 (PMC9041869; doi:10.1371/journal.pone.0266623)
Supplement: S2 Table — (PDF) [file pone.0266623.s003.pdf]

S2 Table. Cancer types and subtypes evaluated in the CANDiD study

Cancer Types: Simplified Classification	Cancer Subtypes: Detailed Classification
Abdominal Cavity	Abdominal cavity tumor malignant: carcinoma
Adrenal Gland	Adrenal tumor malignant: adrenocortical carcinoma
Anal Sac	Anal sac adenocarcinoma
Bile Duct	Bile duct tumor malignant: cholangiocellular carcinoma
Bone, Fibrosarcoma	Bone sarcoma: fibrosarcoma (appendicular)
Bone, Multilobular Osteochondrosarcoma	Bone sarcoma: multilobular osteochondrosarcoma of skull
Bone, Osteosarcoma	Bone sarcoma: osteosarcoma (appendicular)
	Bone sarcoma: osteosarcoma (axial)
Brain	Brain tumor unspecified type
Chondrosarcoma	Cartilage sarcoma: chondrosarcoma (axial)
Ear Canal	Ear canal tumor: ceruminous adenocarcinoma
Stomach	Gastric tumor malignant: leiomyosarcoma
Heart Base	Heart base tumor
Hemangiosarcoma	Hemangiosarcoma: cardiac hemangiosarcoma
	Hemangiosarcoma: abdominal visceral organ hemangiosarcoma
	Hemangiosarcoma: skin / subcutaneous / intramuscular hemangiosarcoma
Liver	Hepatic tumor malignant: hepatocellular carcinoma
Histiocytic Sarcoma	Histiocytic sarcoma: disseminated histiocytic sarcoma
	Histiocytic sarcoma: localized histiocytic sarcoma
Large intestine	Intestinal tumor malignant: gastrointestinal stromal tumor
Leukemia, Acute Lymphoid (ALL)	Lymphoid leukemia: acute lymphoid leukemia
Leukemia, Chronic Lymphoid (CLL)	Lymphoid leukemia: chronic lymphoid leukemia
Lymphoma, Indolent	Lymphoma, indolent: small cell lymphoma
	Lymphoma, indolent: T zone lymphoma
Lymphoma, Intermediate to Large Cell	Lymphoma: cutaneous lymphoma
	Lymphoma: gastrointestinal lymphoma
	Lymphoma: hepatosplenic lymphoma
	Lymphoma: multicentric lymphoma
	Lymphoma: nasal lymphoma
Malignant Melanoma	Malignant melanoma: anal sac melanoma
	Malignant melanoma: cutaneous melanoma
	Malignant melanoma: oral melanoma
	Malignant melanoma: ungual melanoma
Mammary Gland Carcinoma	Mammary gland tumor malignant: mammary gland carcinoma
Mast Cell Tumor	Mast cell tumor: abdominal visceral organ mast cell tumor
	Mast cell tumor: cutaneous mast cell tumor
	Mast cell tumor: intestinal mast cell tumor
	Mast cell tumor: intramuscular mast cell tumor
	Mast cell tumor: mucocutaneous mast cell tumor
	Mast cell tumor: nasal cavity mast cell tumor
	Mast cell tumor: nasal planum mast cell tumor
	Mast cell tumor: oral mast cell tumor
	Mast cell tumor: subcutaneous mast cell tumor
Multiple Myeloma	Multiple myeloma

S2 Table. Cancer types and subtypes evaluated in the CANDiD study (continued)

Cancer Types: Simplified Classification	Cancer Subtypes: Detailed Classification
Nasal Cavity and Paranasal Sinuses	Nasal cavity and paranasal sinus tumor: adenocarcinoma
	Nasal cavity and paranasal sinus tumor: carcinoma
	Nasal cavity and paranasal sinus tumor: chondrosarcoma
	Nasal cavity and paranasal sinus tumor: plasma cell tumor
	Nasal cavity and paranasal sinus tumor: sarcoma
	Nasal cavity and paranasal sinus tumor: squamous cell carcinoma
Nasal Planum	Nasal planum tumor: squamous cell carcinoma
Peripheral Nerve Sheath	Nervous system tumor: peripheral nerve sheath tumor
Oral Cavity	Oral tumor: acanthomatous ameloblastoma
	Oral tumor: fibrosarcoma
	Oral tumor: osteosarcoma
	Oral tumor: plasma cell tumor
	Oral tumor: sarcoma
	Oral tumor: squamous cell carcinoma
Ovary	Ovarian tumor: granulosa cell tumor
Pancreas, Endocrine	Pancreatic tumor: insulinoma
Pituitary	Pituitary tumor: macroadenoma
Prostate	Prostatic tumor: carcinoma
	Prostatic tumor: transitional cell carcinoma
Lung	Pulmonary tumor malignant: adenocarcinoma
	Pulmonary tumor malignant: carcinoma
	Pulmonary tumor malignant: sarcoma
Kidney	Renal tumor: papillary carcinoma
Salivary Gland	Salivary gland tumor: adenocarcinoma
Skin	Skin tumor malignant: adenocarcinoma
	Skin tumor malignant: apocrine sweat gland adenocarcinoma
	Skin tumor malignant: basal cell carcinoma
	Skin tumor malignant: carcinoma
	Skin tumor malignant: sebaceous adenocarcinoma
	Skin tumor malignant: squamous cell carcinoma
Soft Tissue Sarcoma	Soft tissue sarcoma of abdominal visceral organs
	Soft tissue sarcoma of the head and neck
	Soft tissue sarcoma of the trunk and extremities
Spinal Cord	Spinal cord tumor: meningioma
Thymoma	Thymoma
Thyroid	Thyroid tumor: carcinoma
Transmissible Venereal Tumor	Transmissible venereal tumor
Urinary Bladder / Urethra	Urinary bladder / urethral tumor: transitional cell carcinoma

Cancer-diagnosed subjects enrolled in the CANDiD Study were assigned to one of 42 cancer types listed in the left column, based primarily on anatomic location; this simplified classification was adapted from Withrow and MacEwen's Small Animal Clinical Oncology (Sixth Edition) and from the American Joint Committee on Cancer (AJCC) Manual (Eighth Edition). This list was used to derive detection rates by cancer type across the training and the testing sets.

This simplified list was derived from a more detailed list of 82 cancer subtypes listed in the right column, which were additionally defined based on anatomic sub-location and/or histology.

This table shows the correspondence between the simplified classification and the detailed classification.
