# Supplementary material for: Clinical validation of a next-generation sequencing-based multi-cancer early detection “liquid biopsy” blood test in over 1,000 dogs using an independent testing set: The CANcer Detection in Dogs (CANDiD) study
Source: PLoS One. 2022 Apr 26;17(4):e0266623. doi: 10.1371/journal.pone.0266623 (PMC9041869; doi:10.1371/journal.pone.0266623)
Supplement: S3 Table — (PDF) [file pone.0266623.s004.pdf]

S3 Table. Analysis of test sensitivity based on demographic characteristics of cancer-diagnosed subjects in the testing set

	Characteristic	Test sensitivity	p-value
**Age**	Median<br>9.7 years		0.6187
	≥9.7 years<br>n=177	53.1%	
	<9.7 years<br>n=174	56.3%	
**Weight**	Median<br>29.7kg		0.2624
	≥29.7kg<br>n=176	58.0%	
	<29.7kg<br>n=175	51.4%	
**Sex**	Male (Neutered)<br>n=167	59.9%	0.2424
	Male (Intact)<br>n=22	50.0%	
	Female (Spayed)<br>n=150	51.3%	
	Female (Intact)<br>n=11	36.3%	
**Breed**	Purebred<br>n=174	55.2%	0.9452
	Mixed-breed<br>n=177	54.2%	

Note: Neuter status not provided for one male.
Chi-squared test was used to evaluate significance across multiple groups.
