# Supplementary material for: Clinical validation of a next-generation sequencing-based multi-cancer early detection “liquid biopsy” blood test in over 1,000 dogs using an independent testing set: The CANcer Detection in Dogs (CANDiD) study
Source: PLoS One. 2022 Apr 26;17(4):e0266623. doi: 10.1371/journal.pone.0266623 (PMC9041869; doi:10.1371/journal.pone.0266623)
Supplement: S4 Table — (PDF) [file pone.0266623.s005.pdf]

S4 Table. Overview of the putative false positive subjects from the testing set of the CANDiD study

Subject	Age at blood collection (years)	Sex	Breed	Blood collection	Liquid biopsy result reported	Cancer workup initiated[#]	Cancer diagnosed	Clinical details
0374*	7	FS	Mixed	Dec 2020	Apr 2021	May 2021	May 2021	Diagnosed with metastatic cardiac hemangiosarcoma; no clinical signs at diagnosis. Euthanized Aug 2021 after developing clinical signs.
0148**	9	MN	Rough Collie	Jan 2020	Apr 2021	Aug 2021	Aug 2021	Diagnosed with B-cell lymphoma on a normal-sized popliteal lymph node, intra-abdominal lymph node, and spleen by PARR; died of unrelated causes (suspected aspiration pneumonia) Aug 2021.
0009	11	MN	Mixed	Nov 2019	Apr 2021	N/A	N/A	Patient was being managed for progressive neuropathy; euthanized May 2020. No known diagnosis of cancer.
0176	12	FS	Mixed	Feb 2020	April 2021	N/A	N/A	Patient was being managed for chronic kidney disease; died in her sleep Apr 2021. No known diagnosis of cancer.
0334	8	FS	Mixed	Feb 2020	Nov 2021	N/A	N/A	Patient was being managed for chronic kidney disease. No evidence of cancer on imaging Aug 2020 (abdominal ultrasound) and Sep 2021 (thoracic radiographs). Died Oct 2021. No known diagnosis of cancer.
0442	14	FS	Mixed	Feb 2020	Apr 2021	N/A	N/A	Patient was being managed for GI signs; died May 2021. No known diagnosis of cancer.
0153***	3	MI	German Shorthaired Pointer	Mar 2020	Nov 2021	Nov 2021	N/A	No evidence of cancer on workup. FNA cytology of two subcutaneous masses revealed inflammation and fibroplasia.
0326***	1	MN	Mixed	Mar 2020	Nov 2021	Nov 2021	N/A	No evidence of cancer on workup.
0383***	5	MN	Boxer	Mar 2020	Nov 2021	Nov 2021	N/A	No evidence of cancer on workup. Two masses noted Nov 2021; histopathology consistent with peripheral odontogenic fibroma, and cutaneous fibrolipoma.
0735***	2	FI	Labrador Retriever	Apr 2021	Nov 2021	Nov 2021	N/A	No evidence of cancer on workup.

[#] *Cancer workup included physical exam (including oral and rectal exam), routine lab work (CBC, chemistry, urinalysis), three-view thoracic radiographs, abdominal ultrasound, and FNA cytology of any observed masses or enlarged lymph nodes.*

* *Described as a case study in the manuscript, see Figure 4.*

** *Additional clinical details provided in the manuscript.*

*** *These subjects continue to be monitored for evidence of cancer.*
