# Supplementary material for: Clinical validation of a next-generation sequencing-based multi-cancer early detection “liquid biopsy” blood test in over 1,000 dogs using an independent testing set: The CANcer Detection in Dogs (CANDiD) study
Source: PLoS One. 2022 Apr 26;17(4):e0266623. doi: 10.1371/journal.pone.0266623 (PMC9041869; doi:10.1371/journal.pone.0266623)
Supplement: S5 Table — (PDF) [file pone.0266623.s006.pdf]

S5 Table. Subjects with confirmed diagnoses of multiple concurrent primary cancers in the training and testing sets (n=16)

Subject	Cancer types present in each subject	Test result
MP-01	Anal Sac<br>Histiocytic Sarcoma	*Cancer Signal Detected*
MP-02	Lymphoma, Indolent<br>Lymphoma, Intermediate to Large Cell	
MP-03	Lymphoma, Intermediate to Large Cell<br>Urinary Bladder/Urethra	
MP-04	Hemangiosarcoma<br>Thyroid	
MP-05	Hemangiosarcoma<br>Urinary Bladder/Urethra	
MP-06	Leukemia, Chronic Lymphoid (CLL)<br>Mast Cell Tumor	
MP-07	Hemangiosarcoma<br>Stomach	
MP-08	Hemangiosarcoma<br>Soft Tissue Sarcoma	
MP-09	Anal Sac<br>Mammary Gland Carcinoma	*Cancer Signal Not Detected*
MP-10	Malignant Melanoma<br>Soft Tissue Sarcoma	
MP-11	Oral Cavity<br>Urinary Bladder/Urethra	
MP-12	Mast Cell Tumor<br>Soft Tissue Sarcoma	
MP-13	Mast Cell Tumor (Skin)<br>Mast Cell Tumor (Subcutaneous)	
MP-14	Nasal Cavity and Paranasal Sinuses<br>Oral Cavity	
MP-15	Anal Sac<br>Bone, Osteosarcoma	
MP-16	Mammary Gland Carcinoma<br>Ovary	

MP = multiple primary
