# Supplementary material for: Clinical validation of a next-generation sequencing-based multi-cancer early detection “liquid biopsy” blood test in over 1,000 dogs using an independent testing set: The CANcer Detection in Dogs (CANDiD) study
Source: PLoS One. 2022 Apr 26;17(4):e0266623. doi: 10.1371/journal.pone.0266623 (PMC9041869; doi:10.1371/journal.pone.0266623)
Supplement: S6 Table — (PDF) [file pone.0266623.s007.pdf]

S6 Table. Analysis of test performance based on pre-analytical variables in the testing set

Pre-analytical variable	Value/Score	Test Sensitivity	Test Specificity
Time from collection to processing	0 – 1 days	53.8% (n=91)	95.7% (n=70)
	2 – 4 days	56.2% (n=233)	98.7% (n=383)
	5 – 7 days	44.4% (n=27)	100.0% (n=66)
		p = 0.4988	*p = 0.0979*
Extent of hemolysis	Low (<1)	50.0% (n=12)	95.5% (n=22)
	Medium (1-2.5)	58.9% (n=185)	97.3% (n=219)
	High (>2.5)	47.3% (n=146)	99.6% (n=277)
	Not available	100.0% (n=8)	100.0% (n=1)
		p = 0.1040	*p = 0.0520*
Extent of lipemia	Low (0)	53.8% (n=26)	96.3% (n=27)
	Medium (>0-1)	61.6% (n=86)	98.0% (n=245)
	High (>1)	52.4% (n=210)	99.1% (n=233)
	Not available	51.7% (n=29)	100.0% (n=14)
		p = 0.3461	*p = 0.3881*

Chi-squared test was used to evaluate significance across multiple groups.
